# Supplementary material for: Rational correction of pathogenic conformational defects in HTRA1
Source: Nat Commun. 2024 Jul 16;15:5944. doi: 10.1038/s41467-024-49982-8 (PMC11252331; doi:10.1038/s41467-024-49982-8)
Supplement: Supplementary file 1 — Supplementary Information [file 41467_2024_49982_MOESM1_ESM.pdf]

Rational correction of pathogenic conformational defects in HTRA1

Supplementary information

Content

Supplementary Figures 1-15

Supplementary Figure Legends

Supplementary Tables 1-4

Supplementary Methods

Supplementary References

Uncropped gels and blots

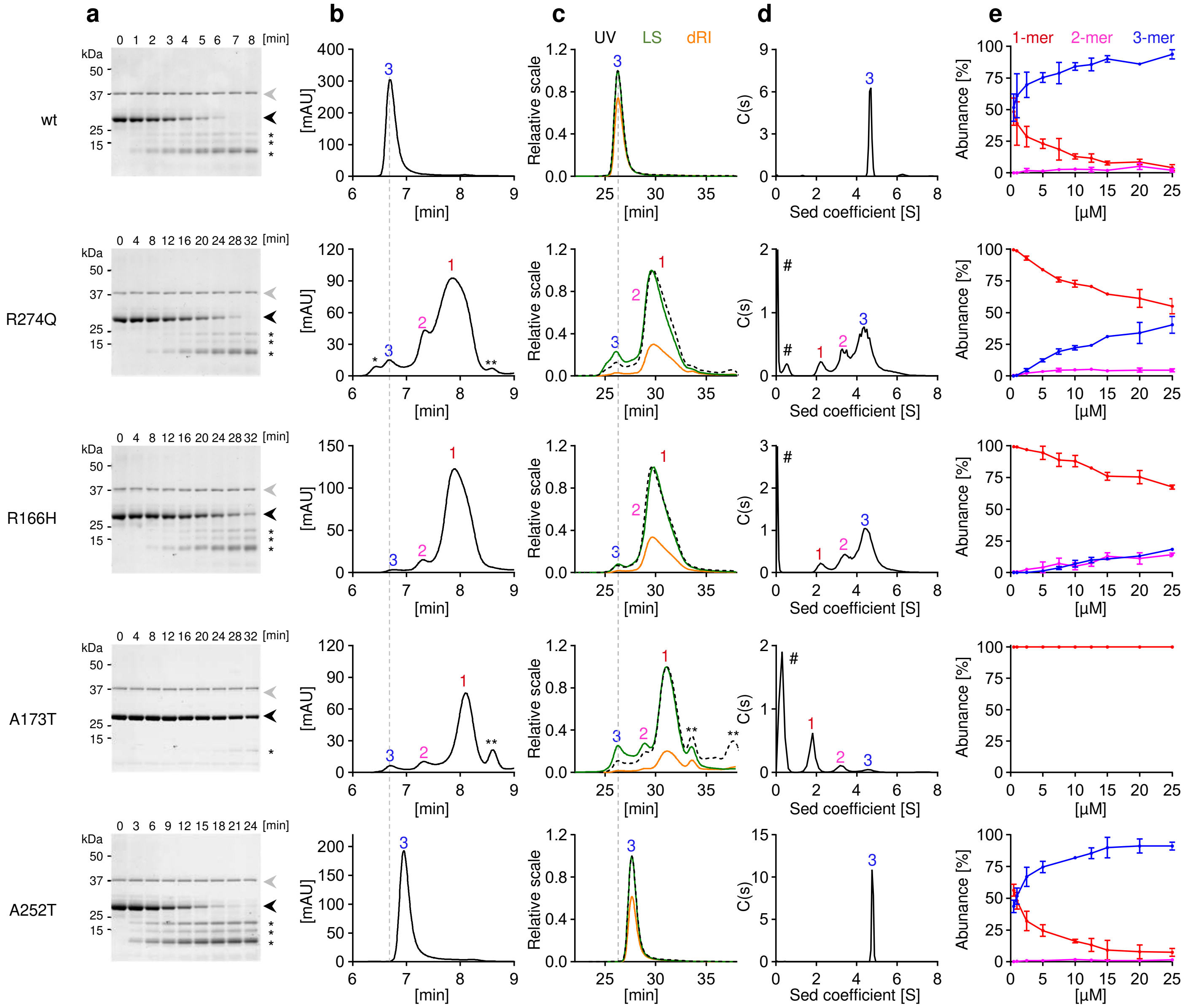

HTRA1	Relative activity [%] (β-casein)	b: Species [%]	c: Species [%]	c: MW [kDa]	d: Species [%]	d: Sed coef [S]	e: Species [%]	e: MW [kDa]
wt	100	3-mer [97]	3-mer [92]	114	3-mer [92]	5.153	3-mer [75]<br>2-mer [1]<br>1-mer [23]	110<br>73<br>37
R274Q	26	3-mer [3]<br>2-mer [11]<br>1-mer [80]	3-mer [4]<br>2-mer [57]<br>1-mer [25]	122<br>54<br>40	3-mer [48]<br>2-mer [19]<br>1-mer [7]	4.799<br>3.580<br>2.458	3-mer [12]<br>2-mer [4]<br>1-mer [84]	109<br>72<br>36
R166H	24	3-mer [1]<br>2-mer [5]<br>1-mer [93]	3-mer [2]<br>2-mer [56]<br>1-mer [28]	120<br>50<br>40	3-mer [61]<br>2-mer [17]<br>1-mer [8]	5.054<br>3.668<br>2.550	3-mer [1]<br>2-mer [4]<br>1-mer [94]	109<br>72<br>36
A173T	14	3-mer [1]<br>2-mer [1]<br>1-mer [70]	3-mer [3]<br>2-mer [5]<br>1-mer [51]	119<br>53<br>41	3-mer [4]<br>2-mer [7]<br>1-mer [24]	5.000<br>3.544<br>1.960	1-mer [100]	36
A252T	34	3-mer [96]	3-mer [93]	107	3-mer [98]	5.271	3-mer [75]<br>2-mer [1]<br>1-mer [25]	109<br>72<br>36

Supplementary Fig. 1 I Proteolytic activity and oligomeric states of wt and mutant HTRA1s. See legend on next page.

Supplementary Fig. 1 I Proteolytic activity and oligomeric states of wt and mutant HTRA1s. a, Representative images of β-casein (20 µM) digest by wt or mutant HTRA1s (1 µM) related to Fig. 1c. Grey arrowheads: HTRA1; black arrowheads: β-casein; *: β-casein degradation fragments. Table below summarizes the relative proteolytic activities of wt and mutant HTRA1s using β-casein as substrate. The activity of wt HTRA1 was set to 100%. **b,** SEC analysis of wt and mutant HTRA1s. 10 µl of 273 µM protein (except A173T: 178 µM) was applied to Agilent AdvanceBio SEC 300 Å column and 2xPBS as mobile phase (AU: absorbance units). Table below summarizes the relative distributions of oligomeric species. **c,** SEC-MALS analysis of wt and mutant HTRA1s. 56 µl of 273 µM protein (except A173T: 178 µM) was applied on Superdex 200 Increase column with 2xPBS as mobile phase. UV, LS and dRI spectra are depicted in overlay. Table below duplicates data depicted in Fig. 1d for comparison with other methods. **d,** Sedimentation velocity AUC analysis of wt and mutant HTRA1s at 80 µM in 100 mM NaH_2PO_4, 150 mM NaCl, pH 8.0. Absorbance data are provided in Source Data. Table below summarizes the sedimentation coefficients and abundance of oligomeric species. **b-d**: 1: 1-mer; 2: 2-mer; 3: 3-mer. **b-e**: protein concentrations are given as monomer-equivalent. **b-c,** Dashed lines indicate the retention time of wt HTRA1 trimers. *: aggregates; **: degradation products or impurities. **d**, # artefact due to fitting. **e,** Native MS analysis of wt and mutant HTRA1s. Shown is relative abundance of monomers, dimers and trimers at protein concentrations ranging from to 0.5 - 25 µM (mean ±SD of 3 datasets). Representative spectra at 5 µM are depicted in Fig. 1 or in Source Data. Table below summarizes the relative distributions and MW of oligomeric species at 5 µM. Source data are provided as a Source Data file.

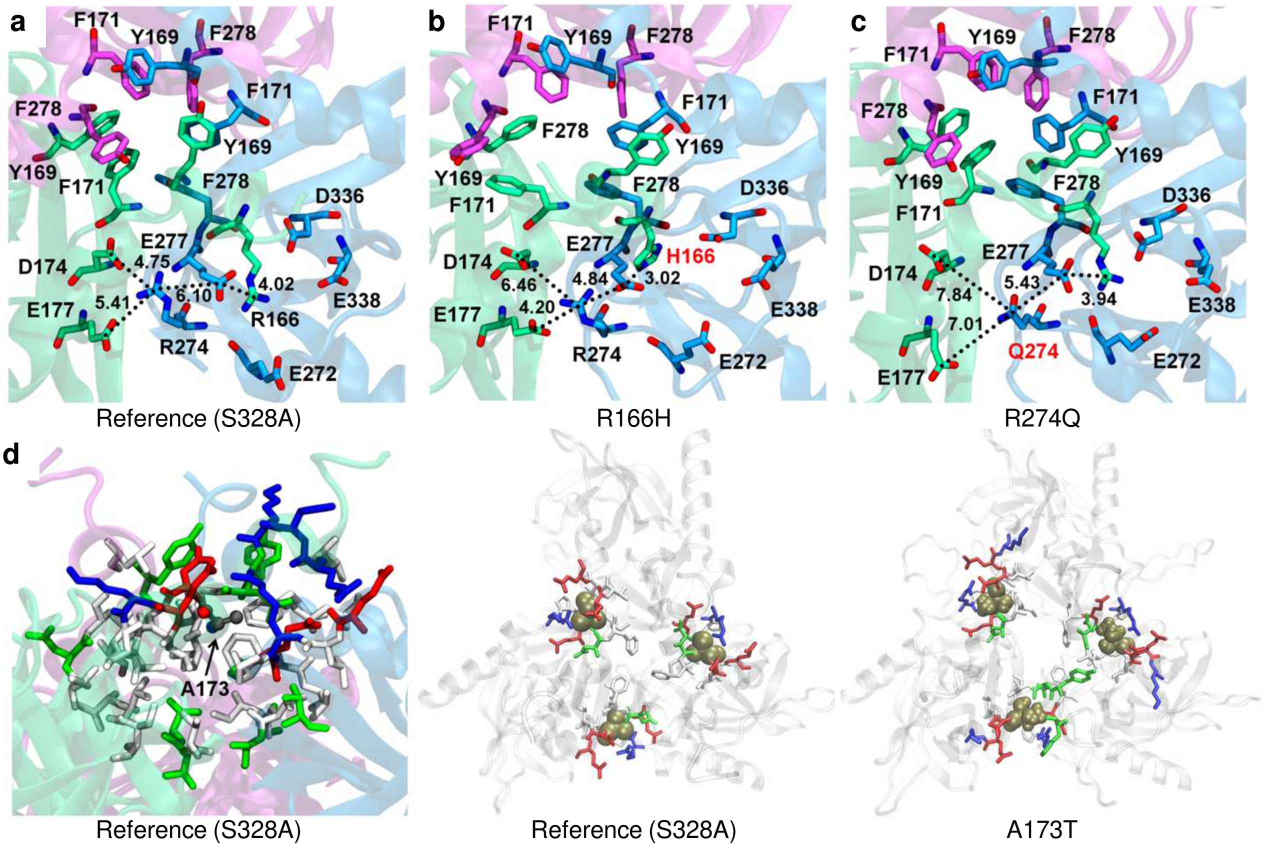

e

Mutation	ΔΔG (kcal/mol)
R166H	-38.8 ± 1.0
A173T	-12.1 ± 0.1
R274Q	-41.7 ± 1.1

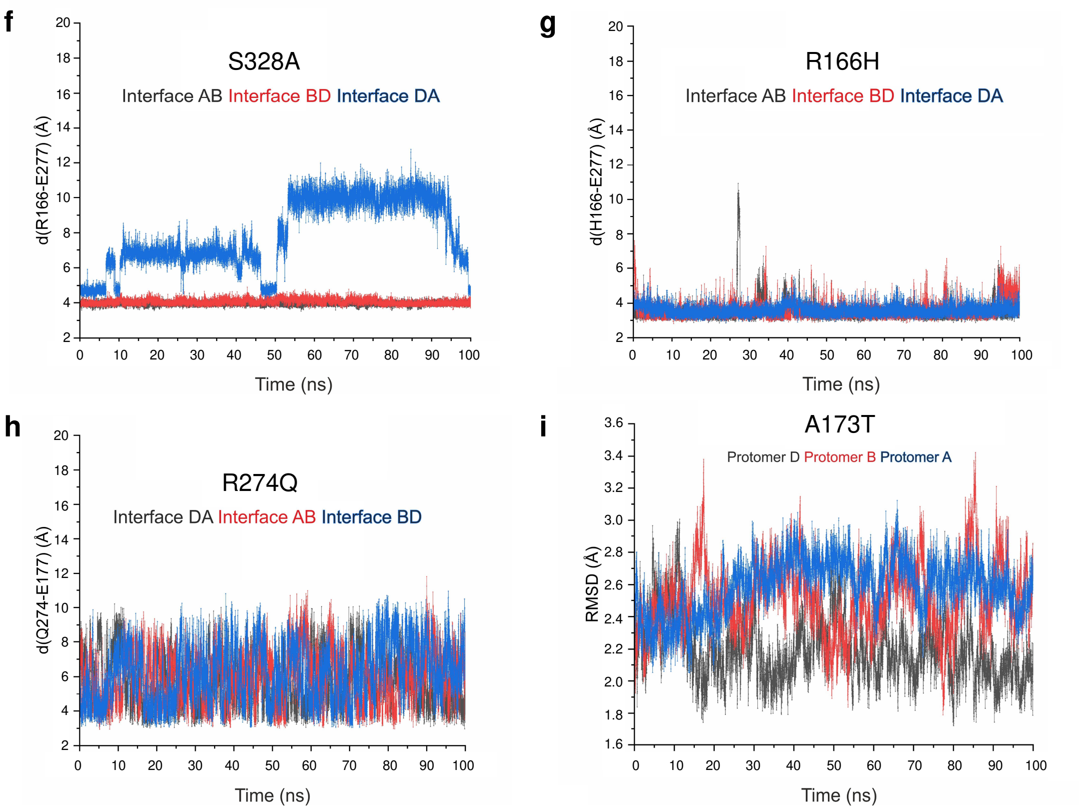

Supplementary Fig. 2 I Computational analysis of the effects of point mutations on HTRA1 trimer stability. See legend on next page.

Supplementary Fig. 2 I Computational analysis of the effects of point mutations on HTRA1 trimer stability. a, Reference trimer interface (PDB ID 3TJO). Protomers are in green, pink and blue; relevant residues as licorice with standard atom coloring code and hydrogen atoms omitted for clarity; distances in Å. **b-c**, Molecular dynamics simulations of mutants R166H and R274Q. R166 forms an inter-protomer salt bridge with E277 (a) which is replaced by the weaker H166-E277 interaction in mutant R166H (b). Accordingly, E277 moves closer to R274, enhancing this intra-protomer interaction. R274 forms inter-protomer salt bridges with D174 and E177 (a) that are abolished by the R274Q mutation (c). **d**, Localization of A173 (grey) in the vicinity of the hydrophobic cluster (a known determinant of trimer formation) comprising Y169, F171 and F278 (left). The hydrophobic contacts within 5 Å of the residue in position 173 become less structured in the mutated system. White: hydrophobic; green: hydrophilic; blue: positively charged; red: negatively charged; tan: residue in position 173. **e**, Free energy perturbation calculations. The values are presented per mole of trimer (corresponding to the contributions of 3 mutations). The thermodynamic cycle used to calculate the effect of point mutations on the stability of the HTRA1 trimer and free energy change as a function of couple parameter λ for the alchemical transformations are depicted in Source Data. **f-h**, Plots of selected relevant distances vs. time for S328A (f), R166H (g) and R274Q (h). **i**, Plot of the RMSD vs. time for A173T. Source data are provided as a Source Data file.

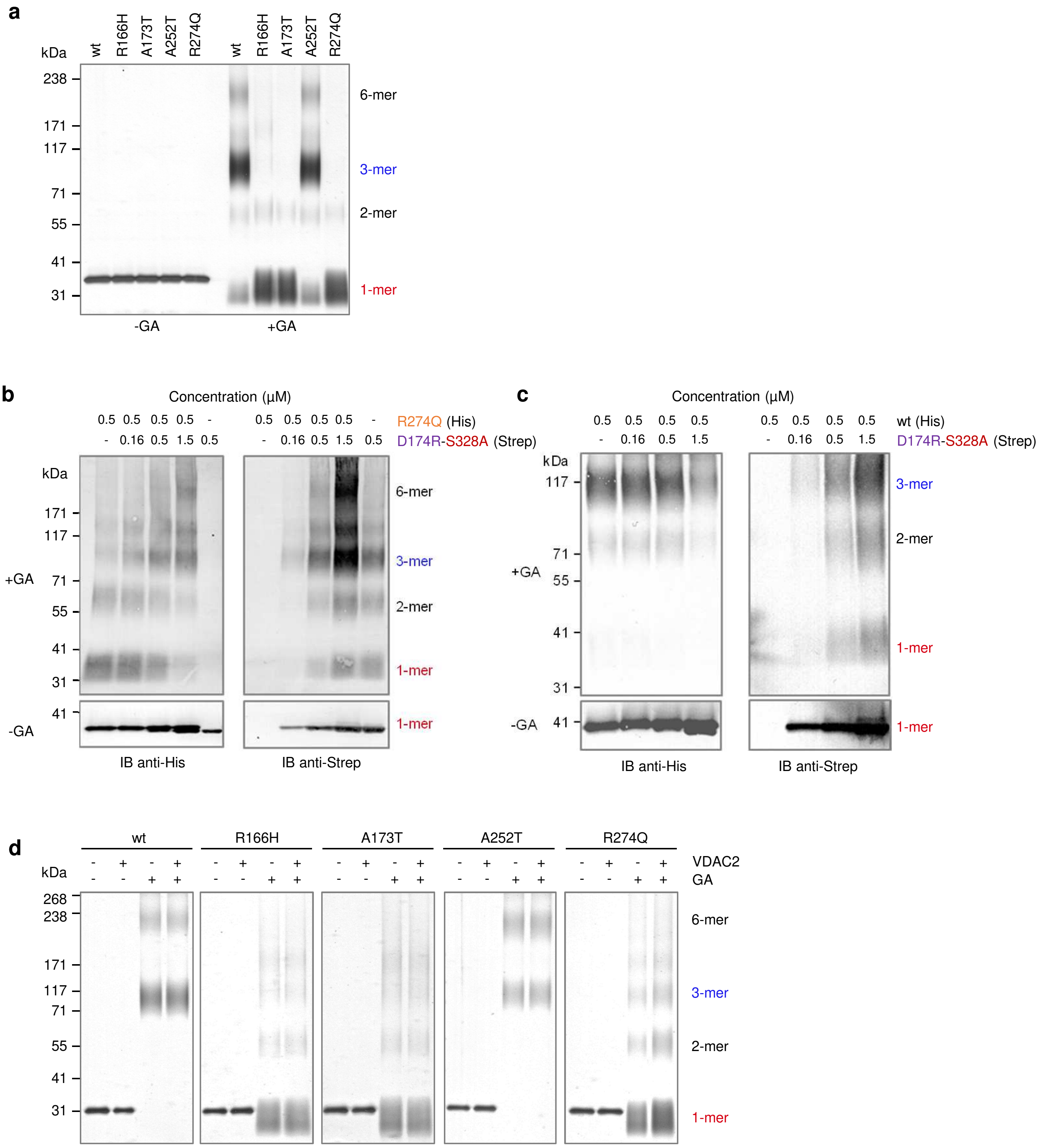

Supplementary Fig. 3 I Oligomeric states of wt and mutant HTRA1s evaluated by chemical crosslinking. a, wt or mutant HTRA1 (0.5 µM) was incubated in the absence or presence of glutaraldehyde (GA). The oligomeric states were analyzed by SDS-PAGE and silver staining. Note that GA increases the electrophoretic motility of HTRA1 monomers. **b-c**, Following incubation of R274Q (b) or wt HTRA1 (c) in the absence or presence of D174-S328A, the oligomeric states of each protein were determined via chemical crosslinking and subsequent immunoblot analysis using anti-His and Strep antibodies. Note that the anti-His antibody recognizes the Strep tag (with Strep-tagged HTRA1 running slightly lower compared to His-tagged HTRA1), however only in the uncrosslinked protein. **d**, Following incubation of HTRA1 in the absence or presence of VDAC2 peptide (50 µM), samples were analyzed as in (a).

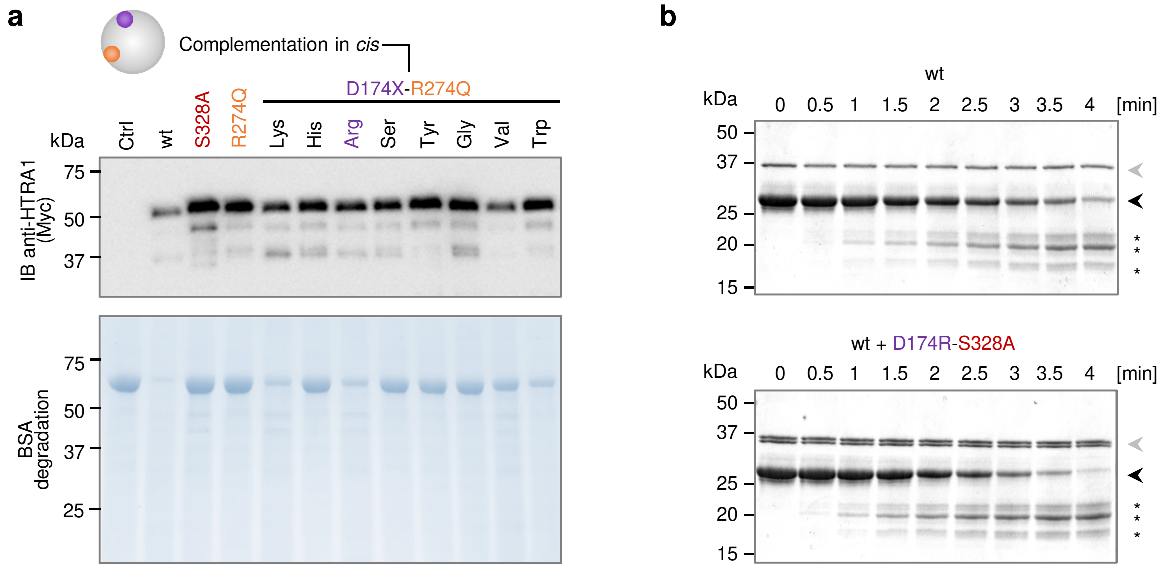

Supplementary Fig. 4 I Functional complementation of R274Q by various D174X mutations in *cis* and impact of D174R-S328A on the activity of wt HTRA1. a, Culture medium from HEK-293T cells transfected to overexpress full-length HTRA1 was collected to assess HTRA1 protein levels (anti-Myc immunoblot, upper panel) and proteolytic activity (BSA degradation assay, lower panel). **b**, Following incubation of wt ΔNHTRA1 (1 µM) in the absence or presence of ΔND174R-S328A (3 µM), proteolytic activity was measured using β-casein (20 µM) as a substrate. Grey arrowheads: HTRA1; black arrowheads: β-casein; *: β-casein degradation fragments.

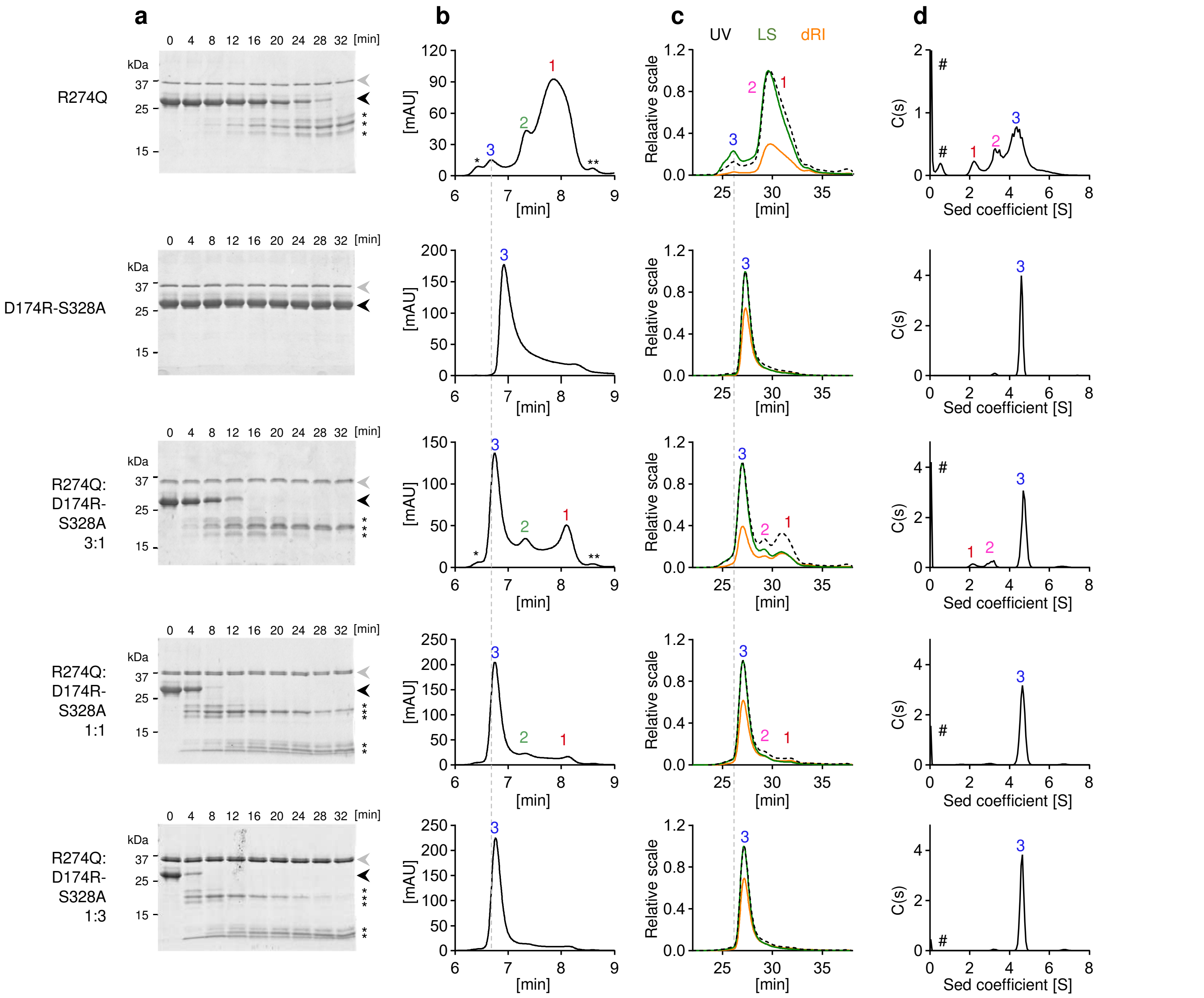

HTRA1	Relative activity [%] (β-casein)	Species [%]	Species [%]	MW [kDa]	Species [%]	Sed coef [S]
R274Q	100%	3-mer [3]<br>2-mer [11]<br>1-mer [80]	3-mer [4]<br>2-mer [57]<br>1-mer [25]	122<br>54<br>40	3-mer [48]<br>2-mer [19]<br>1-mer [7]	4.799<br>3.580<br>2.458
D174R-S327A	1%	3-mer [90]	3-mer [79]	106	3-mer [95]<br>2-mer [4]<br>1-mer [0.6]	5.057<br>3.577<br>1.472
R274Q:<br>D174R-S328A<br>3:1	197%	3-mer [54]<br>2-mer [17]<br>1-mer [28]	3-mer [55]<br>2-mer [12]<br>1-mer [26]	101<br>63<br>43	3-mer [63]<br>2-mer [7]<br>1-mer [5]	5.199<br>3.303<br>2.467
R274Q:<br>D174R-S328A<br>1:1	398%	3-mer [76]<br>2-mer [16]<br>1-mer [6.4]	3-mer [69]<br>2-mer [8]<br>1-mer [4]	103<br>69<br>44	3-mer [85]<br>2-mer [3]<br>1-mer [1]	5.121<br>3.306<br>1.889
R274Q:<br>D174R-S328A<br>1:3	659%	3-mer [95]	3-mer [82]	105	3-mer [90]<br>2-mer [2]<br>1-mer [0.4]	5.089<br>3.585<br>1.666

Supplementary Fig. 5 I In *trans* restoration of R274Q proteolytic activity and trimer assembly by D174R-S328A. See legend on next page.

Supplementary Fig. 5 I In *trans* restoration of R274Q proteolytic activity and trimer assembly by D174R-S328A. R274Q data which are already presented in Supplementary Fig. 1 are depicted for comparison with D174R-S328A and R274Q:D174R-S328A mixtures. **a,** Representative images of β-casein (20 µM) digest by R274Q (1 µM) mixed with varying amounts of D174R-S328A related to Fig. 2b. Grey arrowheads: HTRA1; Black arrowheads: β-casein; *: β-casein degradation fragments. Table below summarizes the relative proteolytic activities of HTRA1. The activity of R274Q alone was set to 100%. **b,** SEC analysis of R274Q, D174R-S328A and R274Q mixed with D174R-S328A. 10 µl of 273 µM protein solution (except A173T: 178 µM) were applied on Agilent AdvanceBio SEC 300 Å column and 2xPBS as mobile phase (AU: absorbance units). Table below summarizes the relative distributions of oligomeric species. **c,** SEC-MALS analysis of R274Q, D174R-S328A and R274Q mixed with D174R-S328A. 56 µl of 273 µM protein solution were applied on Superdex 200 Increase column with 2xPBS as mobile phase. UV, LS and dRI spectra are depicted in overlay. Table below summarizes the relative distributions and MW of oligomeric species (UV spectra). **d,** Sedimentation velocity AUC analysis of R274Q, D174R-S328A and R274Q mixed with D174R-S328A at 80 µM concentration in 100 mM NaH_2PO_4, 150 mM NaCl, pH 8.0. Table below summarizes the sedimentation coefficients and abundance of oligomeric species. **b-d**: 1: 1-mer; 2: 2-mer; 3: 3-mer. Protein concentrations are given as monomer-equivalent. **b-c,** Dashed lines indicate the retention time of wt HTRA1 trimers. *: aggregates; **: degradation products or impurities. **d**, # artefact due to fitting. Source data are provided as a Source Data file.

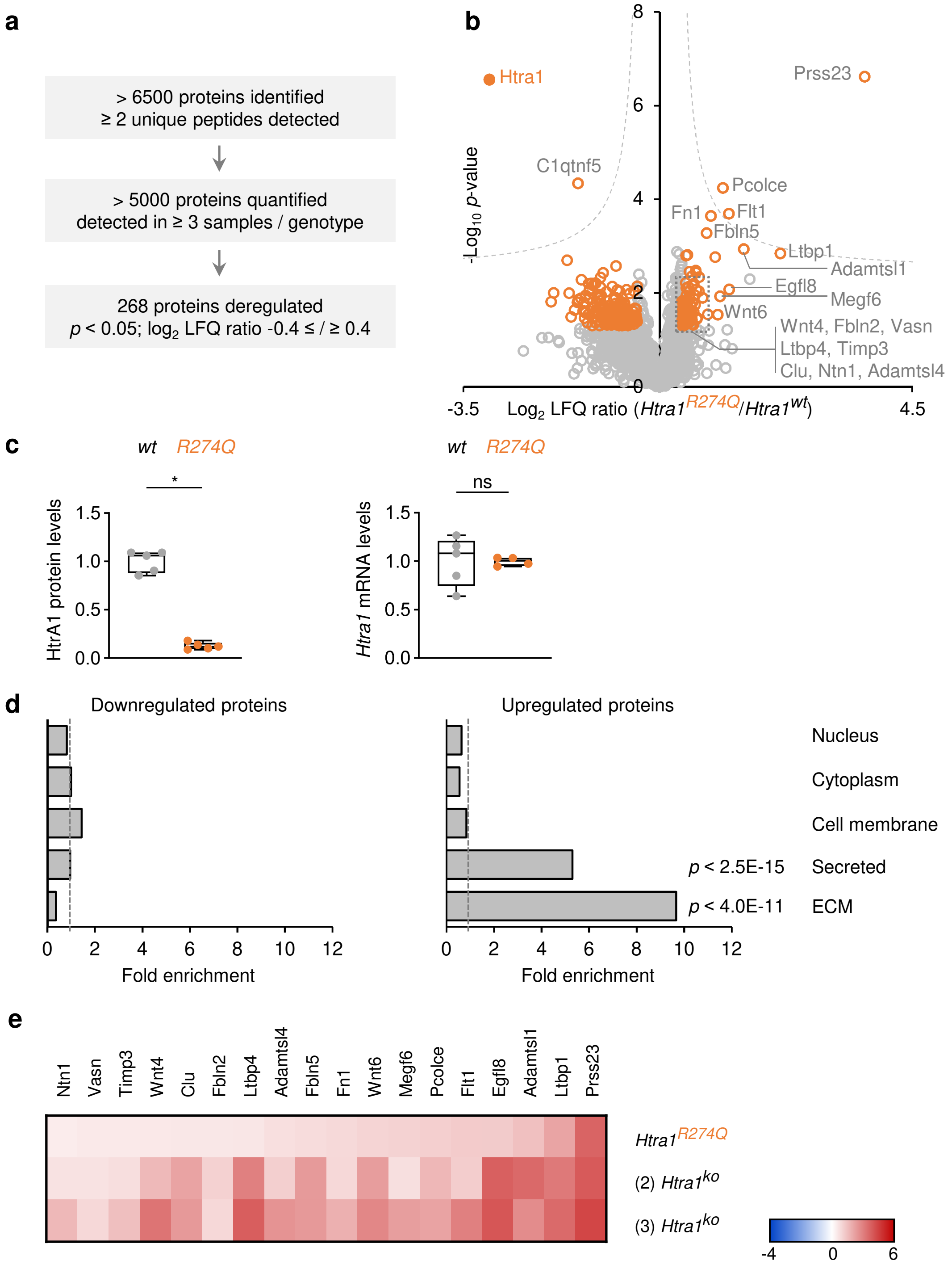

Supplementary Fig. 6 I Proteomic and RT-qPCR analyses of *Htra1*^{wt} and *Htra1*^{R274Q} brain vessels. a, Summary of LC–MS/MS and label-free quantification results. **b**, Volcano plot of all proteins quantified by MS. Orange circles: deregulated proteins; hyperbolic curve: permutation-based FDR estimation; proteins are labelled with gene names. **c**, HtrA1 protein levels determined by MS (left) and *htra1* mRNA levels determined by RT-qPCR (right; *Gapdh* was used as a housekeeping gene). The mean abundance in *Htra1*^{wt} vessels was set to 1; results as box-and-whisker plots (centerline: median; limits: 25th and 75th percentile; whiskers: minimum and maximum); data points represent individual mice; significance was tested by two-sided unpaired *t*-test. **d**, Subcellular location of deregulated proteins. Proteins were categorized based on the Uniprot database. For each protein category, the number of proteins down- (left) or upregulated (right) in *Htra1*^{R274Q} vessels was normalized to the total number of proteins quantified in this category. Extracellular matrix proteins (ECM) represent a subentity of secreted proteins. *p* values were calculated by Fisher´s exact test. **a-d**, n=5 mice per genotype, except htra1 mRNA levels in wt vessels (n=4). **e**, Overlap between *Htra1*^{R274Q} and *Htra1*^{KO} proteomic profiles**.** Log_2 LFQ ratio-based heatmap of proteins upregulated (p<0.05 and Log_2 LFQ ratio ≥0.4) in the cerebral vasculature of both *Htra1*^{R274Q} and *Htra1*^{KO} mice. Results are from the present study (1), Zellner et al., 2018[1] (2) and Kato et al., 2021[2] (3). Source data are provided as a Source Data file.

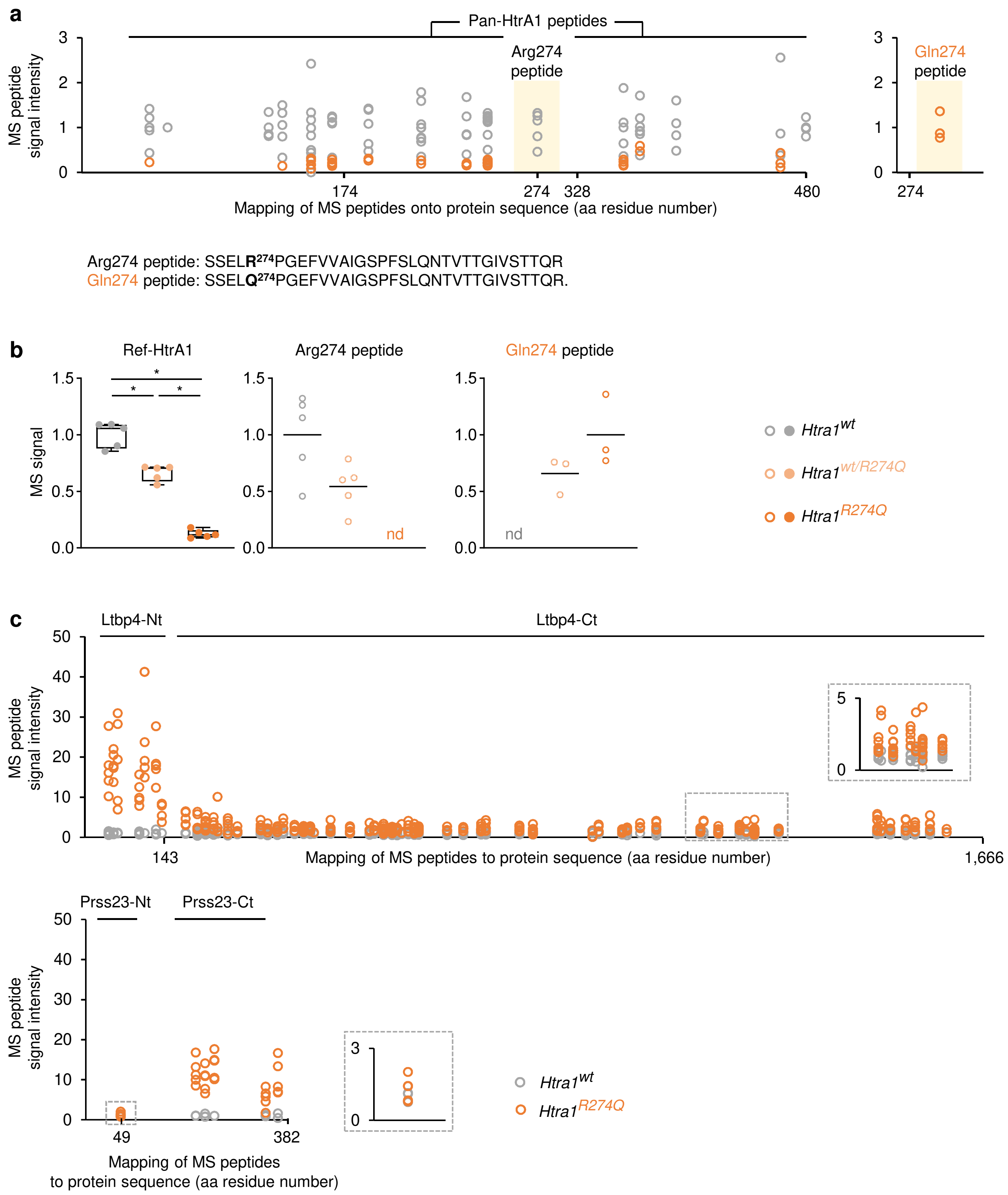

Supplementary Fig. 7 I Mass spectrometry-based analysis of distinct HtrA1 isoforms and of domain-specific changes in the abundance of Ltbp4 and Prss23. a, c, MS peptides mapped onto the respective protein sequences. The analysis of other key proteins is depicted in Source Data. **b**, MS signal of reference (Ref) HtrA1, Arg274-peptide or Gln274-peptide. LFQ values as box-and-whisker plots (centerline: median; limits: 25th and 75th percentile; whiskers: minimum and maximum) with filled circles; significance was tested by two-sided unpaired *t*-test. MS peptide intensities as scatter plots (black bars: mean) with empty circles (nd: not detected). **a-c**, n=5 mice per genotype. The mean MS signal intensity in *Htra1^{wt}* or *Htra1^{R274Q}* samples was set to 1 as appropriate. Source data are provided as a Source Data file.

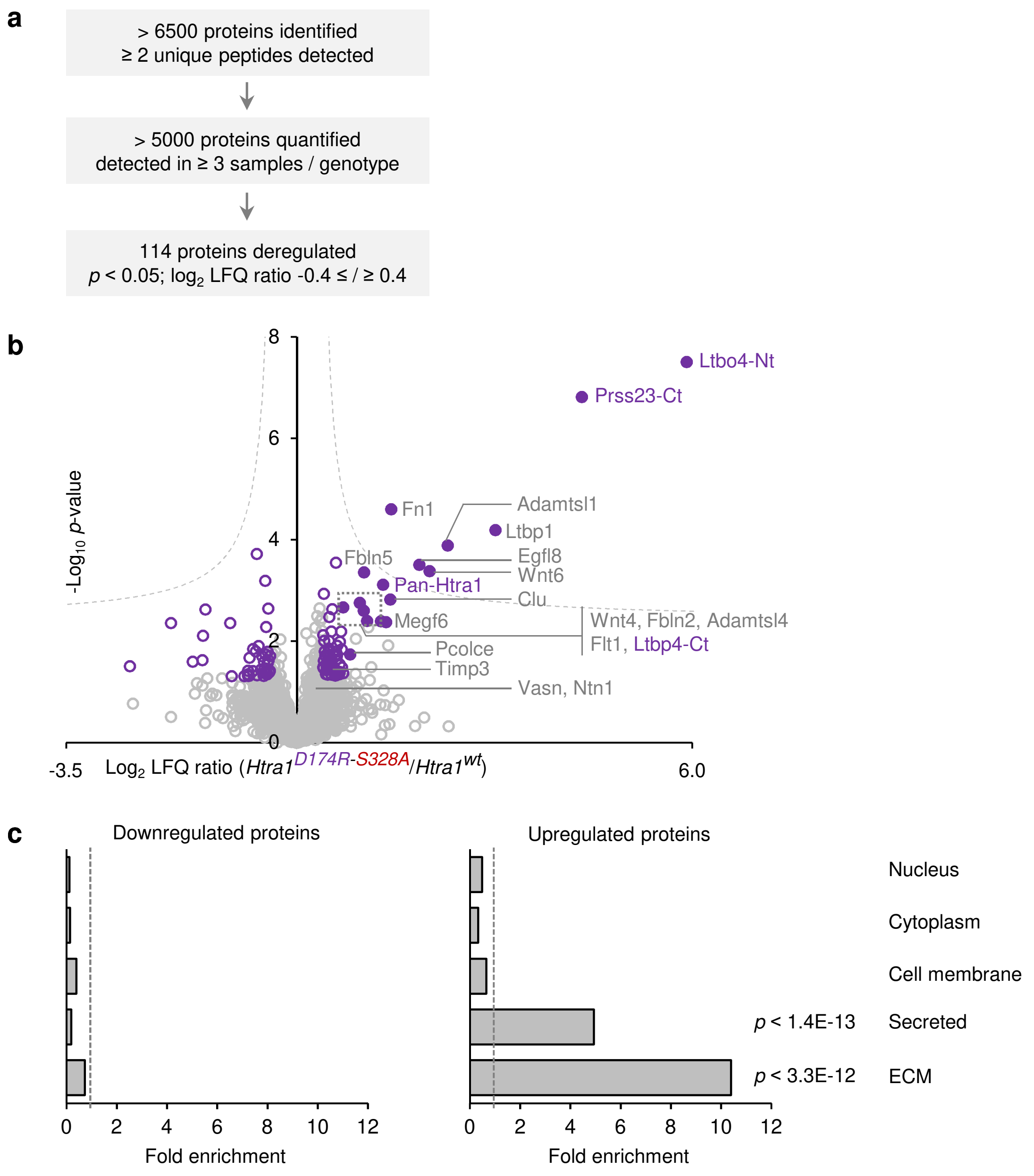

Supplementary Fig. 8 I Proteomic analysis of *Htra1^{wt}* and *Htra1$^{D174R\text{-}S328A}$* brain vessels. a, Summary of LC–MS/MS and label-free quantification results. **b**, Volcano plot of all proteins quantified by MS as in Fig. 3g. Purple circles: deregulated proteins. **c**, Subcellular location of deregulated proteins analyzed as in Supplementary Fig. 6d. *p* values were calculated by Fisher´s exact test. **a-c**, n=5 mice per genotype. Source Data are provided as a Source Data file.

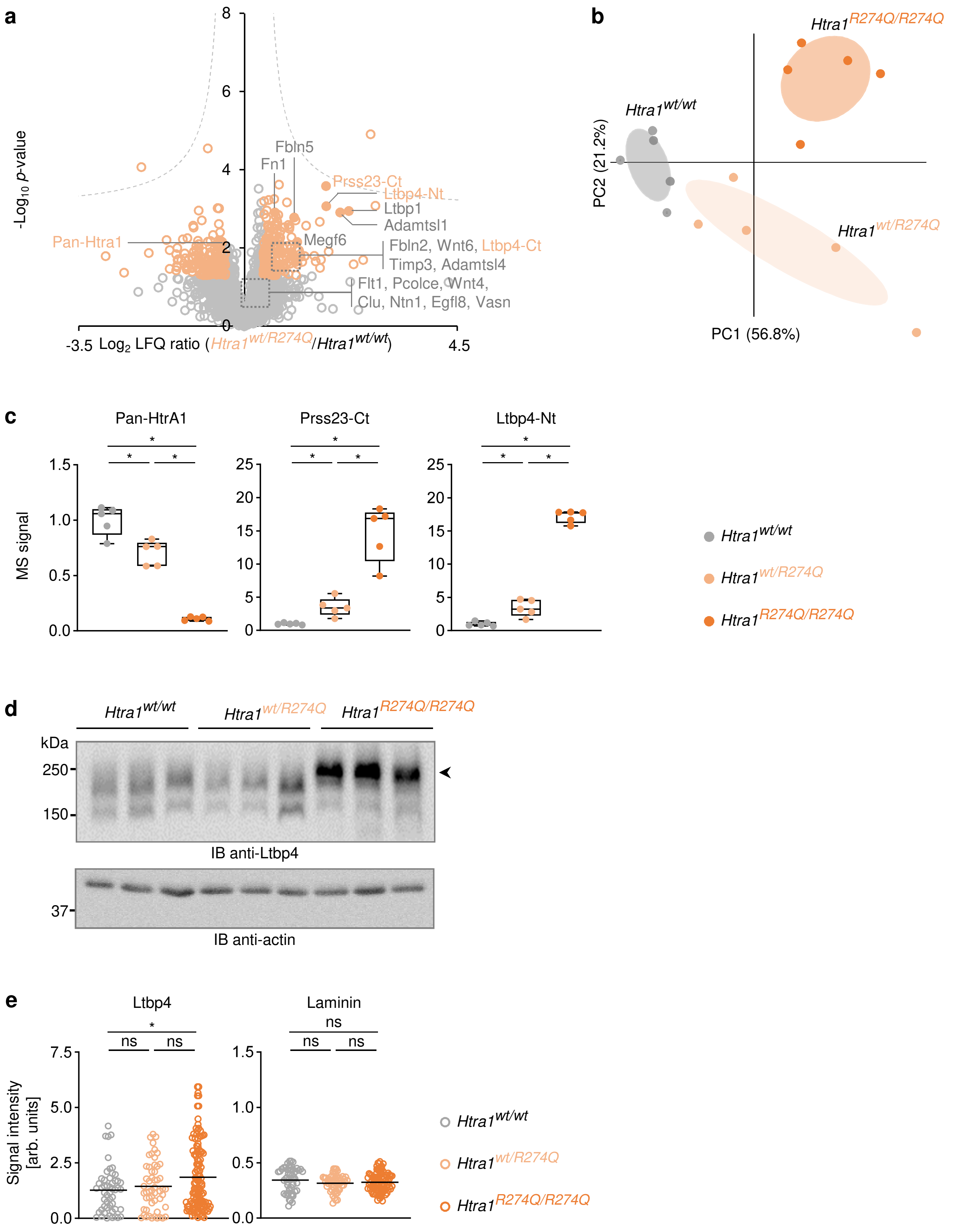

Supplementary Fig. 9 I Cerebrovascular proteome of *Htra1*$^{wt/R274Q}$ and *Htra1*$^{R274Q/R274Q}$ mice. Vessels from *Htra1*$^{wt/wt}$, *Htra1*$^{wt/R274Q}$ or *Htra1*$^{R274Q/R274Q}$ mice were analyzed by MS (**a-c,** n=5 mice per genotype; **c**, significance was tested by two-sided unpaired *t*-test), IB (**d,** vessels lysates from 3 mice per genotype), or IHC (**e,** wt: n=52 arteries from 3 mice; wt/R274Q: n=53 arteries from 4 mice; R274Q/R274Q: n=87 arteries from 4 mice; significance was tested by two-sided unpaired Mann-Whitney U-test) as in Fig. 3. **b**, Principal component analysis of protein abundance as in Fig. 4g. Filled circles represent individual mice (n=5 per genotype). Source data are provided as a Source Data file.

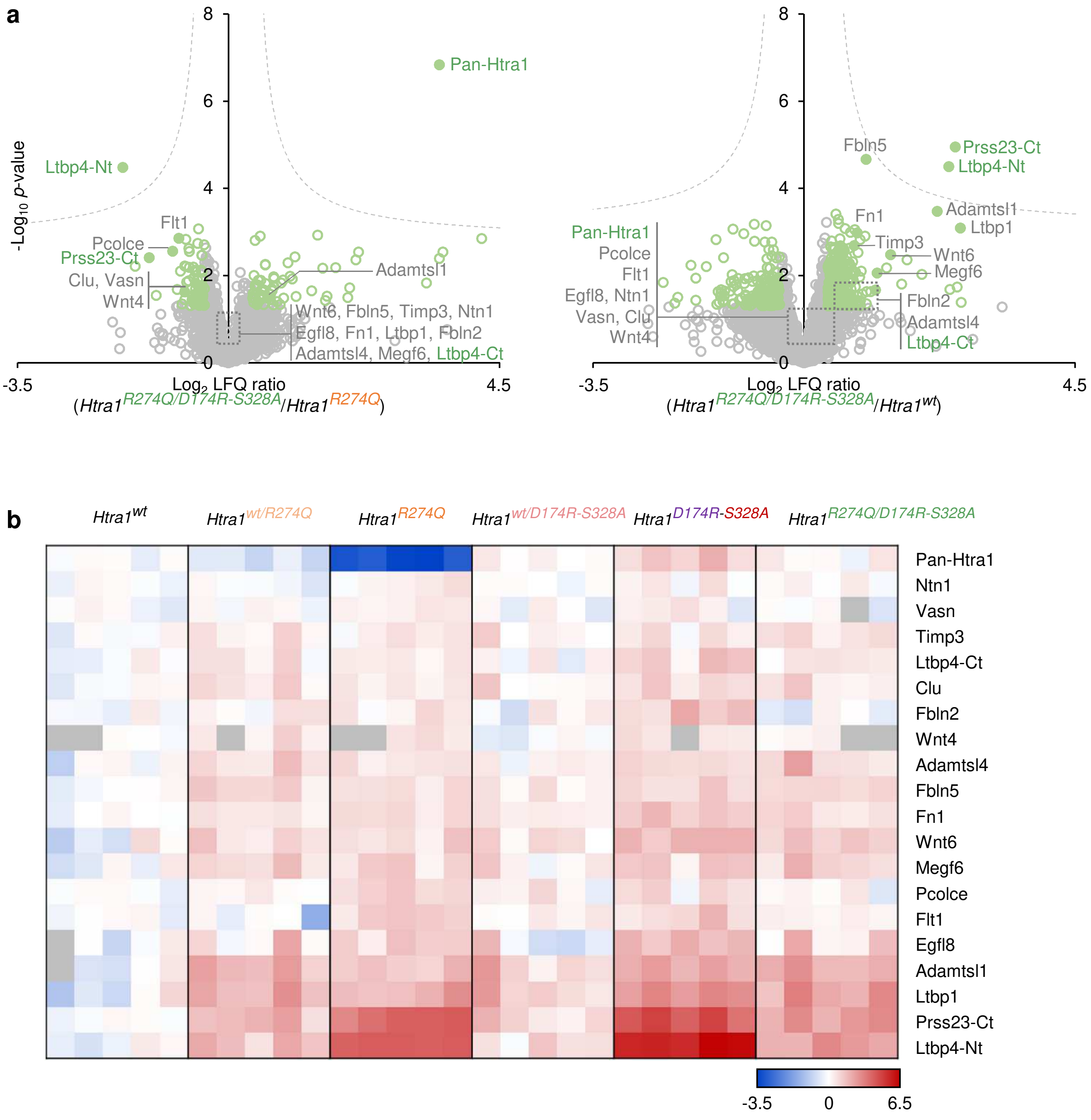

Supplementary Fig. 10 I Comparative analysis of *Htra1*$^{R274Q/D174R\text{-}S328A}$ brain vessels. a, Volcano plot of all proteins quantified by MS as in Fig. 3g. Green circles: deregulated proteins. **b**, Log_2 LFQ value-based heatmap of protein abundance changes in mouse brain vessels. The mean signal intensity in *Htra1*^{wt} samples was set to 0; horizontal lines represent individual mice; grey: not detected. **a-b**, n=5 mice per genotype. Source data are provided as a Source Data file.

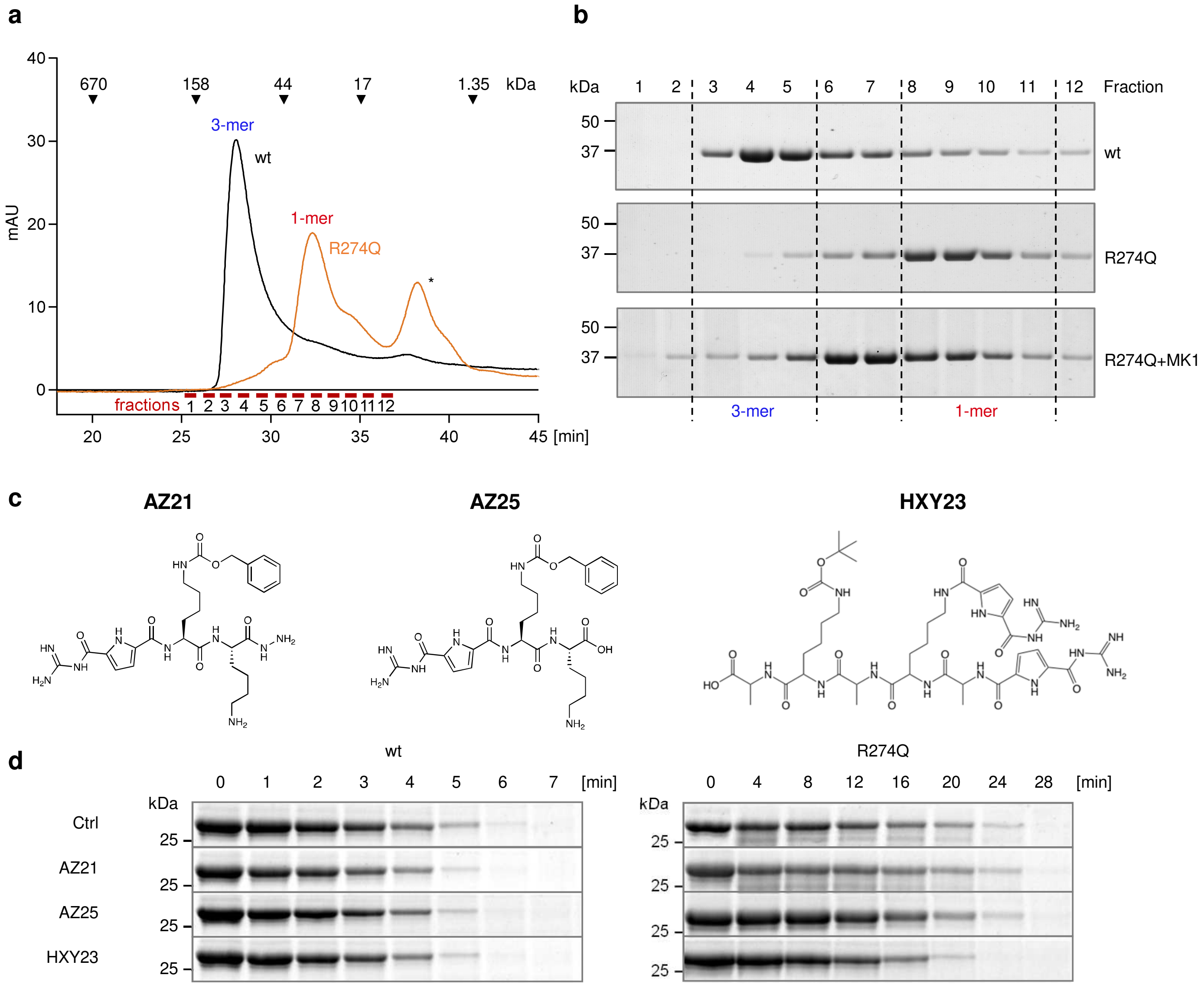

Supplementary Fig. 11 I Oligomeric states of HTRA1-R274Q in the presence of MK1 and impact of control GCP-containing compounds on the activity of HTRA1. a, HTRA1 wt and R274Q were analyzed by SEC. Overlay of UV chromatograms are depicted. The retention times of protein standards are marked by arrowheads. AU: absorbance units, *: degradation products or impurities. The chromatogram of R274Q in the presence of MK1 is not shown due to high UV absorbance of MK1 (>3000 mAU). **b**, HTRA1 wt and R274Q were analyzed by SEC followed by analysis of the eluted fractions (labelled in red in panel a) by SDS-PAGE and Coomassie staining. Where indicated, 2.5 mM MK1 was added both in the R274Q sample and to equilibrate the column. **c**, Structures of compounds. **d**, β-casein degradation by HTRA1 in the presence of control compounds. Following preincubation with the compounds indicated (2.5 mM), 1 μM wt HTRA1 or HTRA1-R274Q were incubated with 20 μM β-casein at 37°C. Samples were taken at indicated time points and subjected to SDS-PAGE followed by Coomassie staining. Source data are provided as a Source Data file.

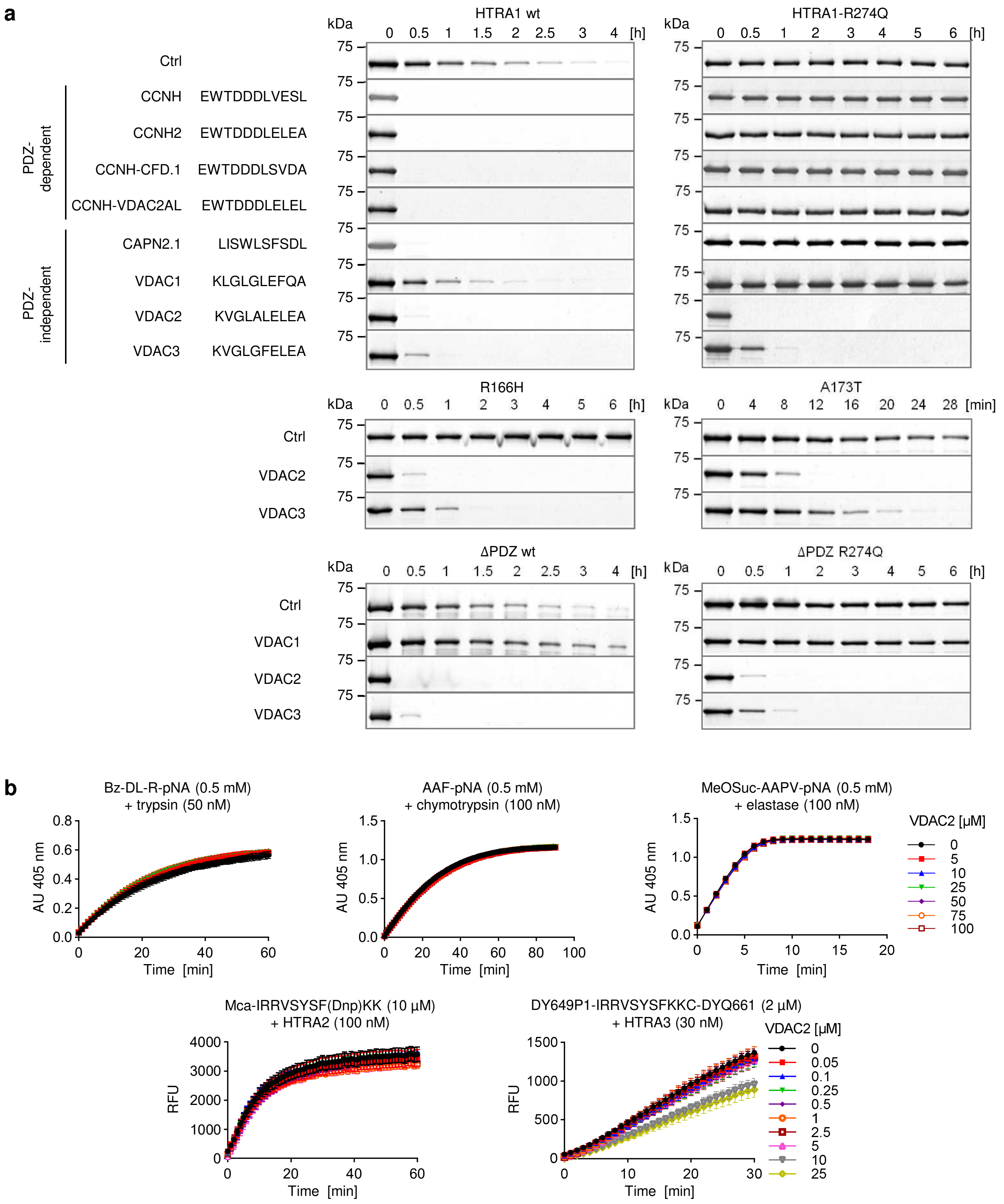

Supplementary Fig. 12 I Impact of peptidic modulators on the activity of HTRA1 and other proteases. a, Time-dependent degradation of Tau by the indicated HTRA1 variants in the absence or presence of the indicated peptides. Following preincubation with 50 µM of peptides, 1 µM HTRA1 was incubated with 3 µM Tau at 37°C. Samples were taken at time points indicated and subjected to SDS-PAGE followed by Coomassie staining. **b,** Selectivity of VDAC2 peptides analyzed by titration experiments. Proteases were preincubated with the peptide concentrations indicated, followed by addition of respective substrates at 37°C. Absorbance or fluorescence was recorded over time. AU: absorbance units, RFU: relative fluorescent units. Graphs depict the mean AU or RFU ±SD of 6 datapoints. The specific or relative activity estimates are provided in Source Data. Source data are provided as a Source Data file.

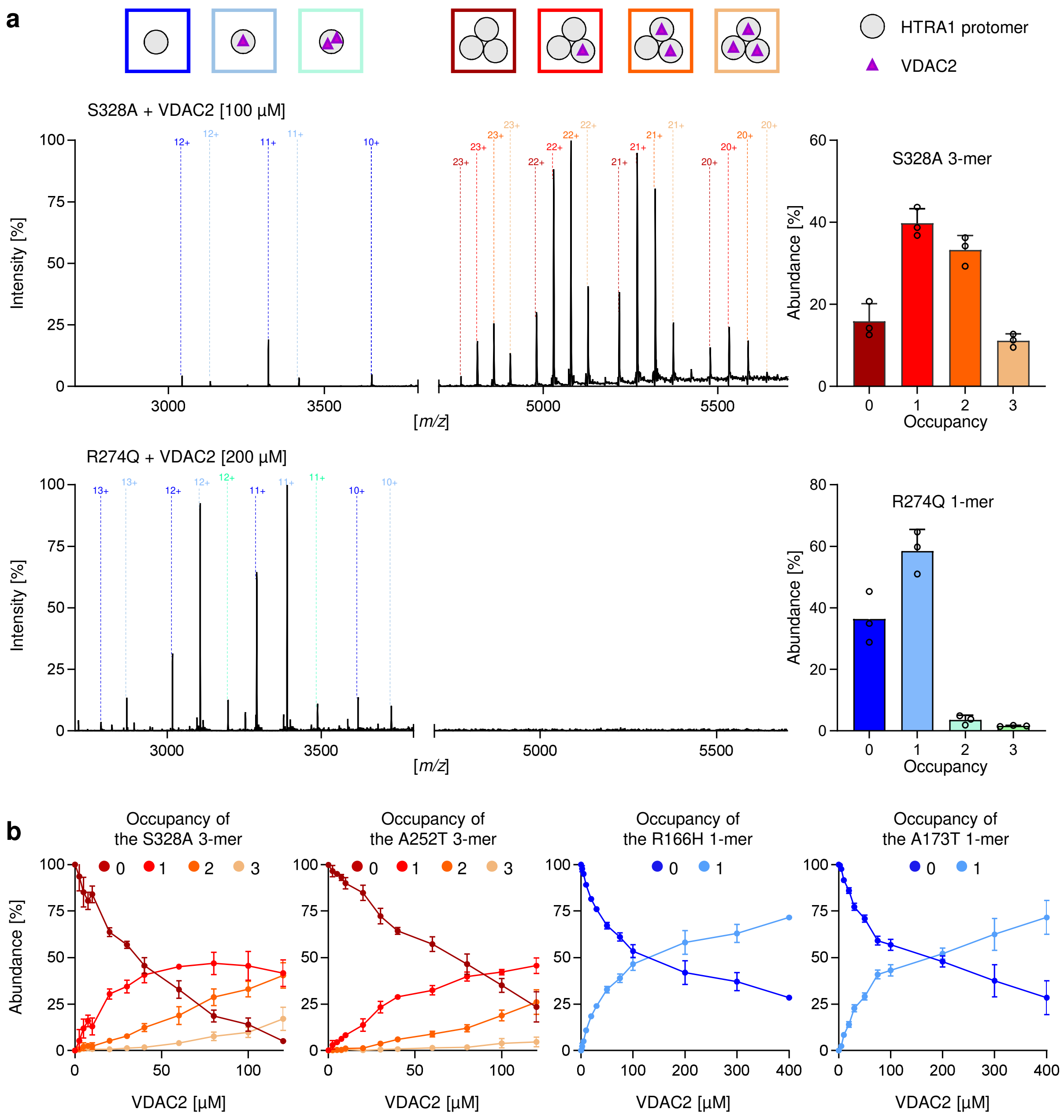

Supplementary Fig. 13 I Analysis of the interaction between HTRA1 and VDAC2 by native MS. a, Left panels: representative spectra of 5 µM HTRA1 S328A in the presence of 100 µM VDAC2 peptide or of 5 µM R274Q in the presence of 200 µM VDAC2 peptide. Right panels: Binding of VDAC2 peptide to HTRA1 wt trimers or to R274Q monomers. Histograms depict the average occupancy (0, 1, 2 or 3) ±SD of 3 datasets. Empty circles: individual datapoints. **b**, Titration of VDAC2 peptide to determine occupancy of HTRA1 trimers (S328A and A252T) or monomers (R166H and A173T). Graphs depict the average occupancy of peptide at HTRA1s (0, 1, 2 or 3) ±SD of 4 datasets. Source data are provided as a Source Data file.

a

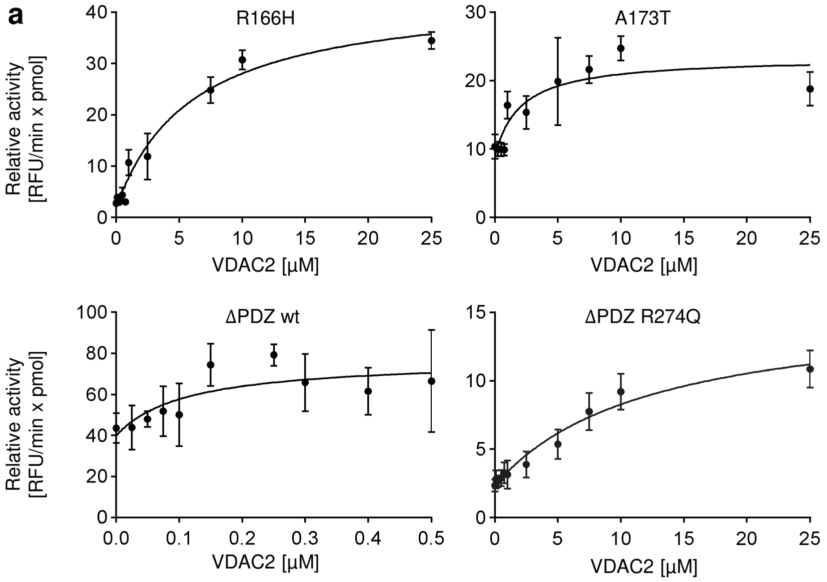

	V_{max} [RFU/min x pmol]	K_d [µM]	Offset
R166H	42.1 (±2.1)	6.1 (±1.0)	1.9 (±0.6)
A173T	14.5 (±1.3)	2.0 (±0.8)	8.9 (±0.9)
ΔPDZ wt	37.3 (±8.0)	0.1 (±0.1)	39.8 (±5.3)
ΔPDZ R274Q	13.2 (±1.4)	12.3 (±2.9)	2.3 (±0.2)

b

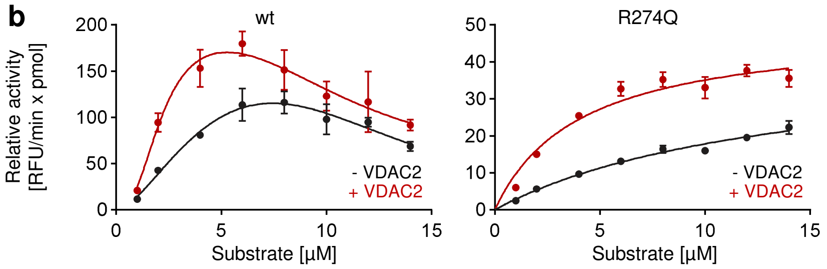

wt	- VDAC2	+ VDAC2 (0.5 µM)
V_{max} [RFU/min x pmol]	197.8 (±95.3)	204.3 (±34.6)
K_m [µM]	4.7 (±2.8)	2.2 (±0.4)
n	1.7 (±0.5)	2.5 (±0.7)
V_i [RFU/min x pmol]	8.0 (±60)	47.5 (±40.8)
K_i [µM]	11.5 (±5.7)	10.5 (±3.4)
z	= 3	= 3

R274Q	- VDAC2	+ VDAC2 (7.5 µM)
V_{max}	42.4 (±3.8)	49.4 (±2.5)
K_m	13.8 (±2.1)	4.1 (±0.6)

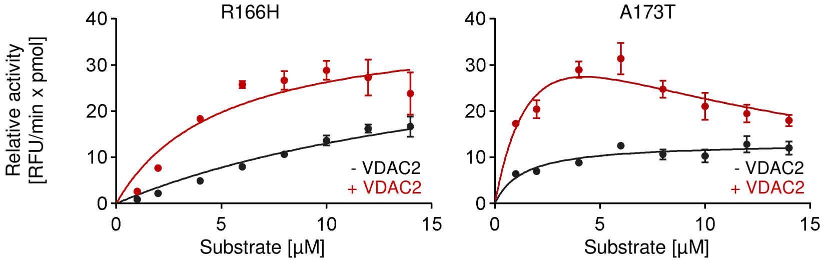

R166H	- VDAC2	+ VDAC2 (10 µM)
V_{max}	= 40	39.3 (±4.2)
K_m	20.7 (±0.7)	5.0 (±1.4)

A173T	- VDAC2	+ VDAC2 (10 µM)
V_{max}	13.2 (±0.6)	60.0 (±17.2)
K_m	1.4 (±0.3)	2.5 (±1.2)
K_{si} [µM]		7.2 (±3.4)

Supplementary Fig. 14 I Impact of VDAC2 peptide on the activity HTRA1. a, Normalized cleavage rates of peptidic fluorescence-quenched substrate DY649P-IRRVSYSFKKC-DYQ661 (2 µM) by the indicated HTRA1 variants plotted vs. concentration of VDAC2 peptide. (mean ±SD of 6 datasets). Data were fitted to the weak-binding equation to yield V_{max} and K_d (black line); brackets indicate SE of the fit. **b,** Normalized cleavage rates of peptidic fluorescence-quenched substrate DY649P-IRRVSYSFKKC-DYQ661 by wt and mutant HTRA1s in absence (black circles) and in presence of fixed VDAC2 peptide concentrations (red circles) plotted vs. substrate concentrations (mean ±SD of 4 experimental data). **a-b,** Data were fitted as described in Results and Methods; brackets indicate SE of the fit. Source data are provided as a Source Data file.

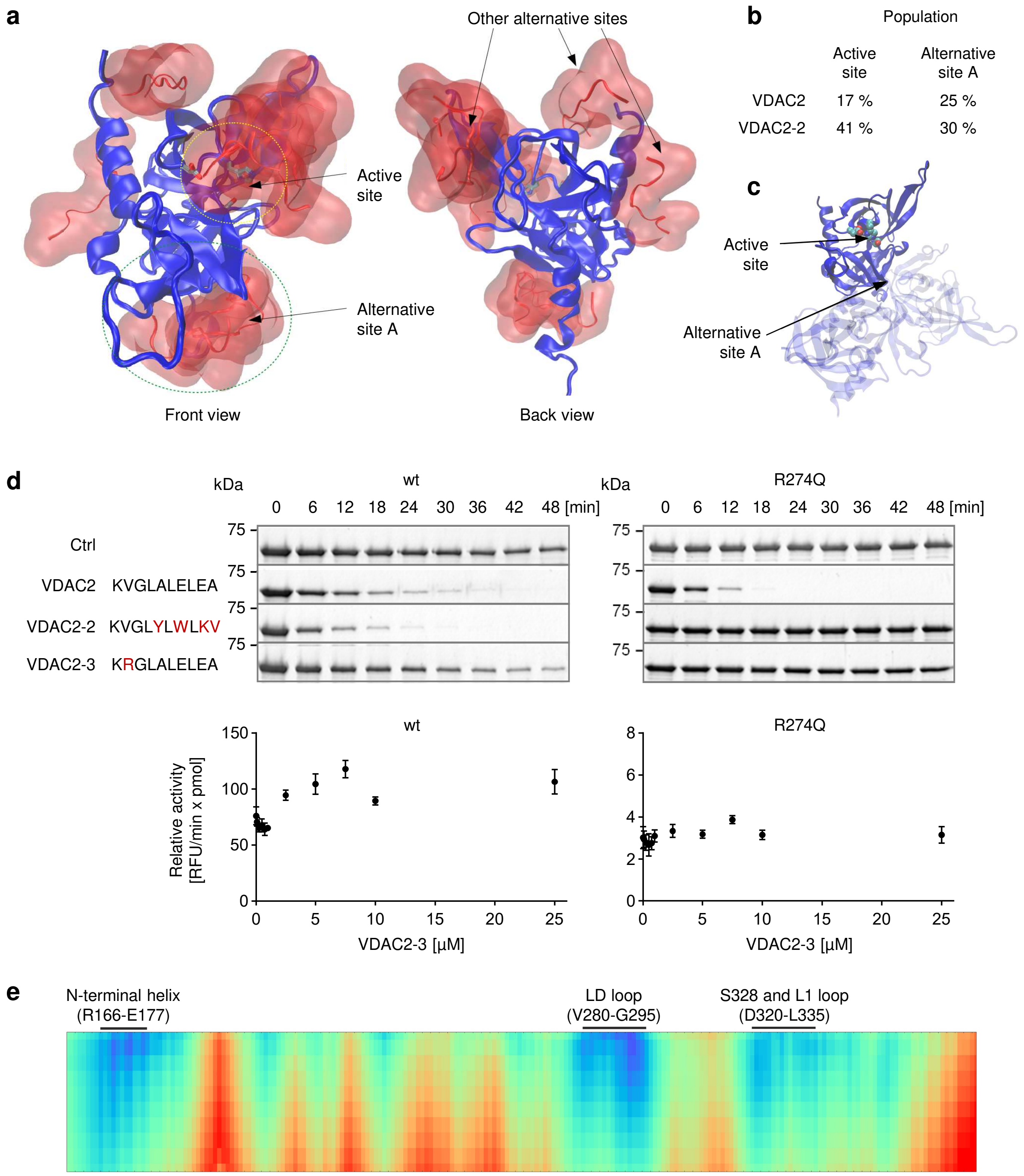

Supplementary Fig. 15 I Identification of VDAC2 peptide binding site to HTRA1-R274Q. See legend on next page

Supplementary Fig. 15 I Identification of VDAC2 peptide binding site to HTRA1-R274Q. a-b, Docking analysis of VDAC2 and VDAC2-2 (optimized to bind the active site) peptides on a monomeric model of HTRA1-R274Q. **a**, Representation of the most favored binding sites of VDAC2 peptide at HTRA1. Red clouds indicate the main clusters of docking poses, their centroid structure is represented with red ribbons. HTRA1-R274Q is shown in blue cartoon and the residues of the catalytic triad are highlighted with sticks (licorice style). **b**, Relative populations of peptide in the active site and in the alternative site A are shown for VDAC2 and VDAC2-2 peptides. **c**, Peptide binding at the alternative site A mimics the inter-protomer interface in the trimer form. **d**, Upper panels: Time-dependent degradation of Tau by HTRA1 in the presence of VDAC2, VDAC2-2 or VDAC2-3 peptides. Following preincubation with 50 µM of the respective peptides, 1 µM wt HTRA1 or R274Q was incubated with 3 µM Tau at 37°C. Samples were taken at indicated time points and subjected to SDS-PAGE followed by Coomassie staining. Lower panels: Following preincubation with the indicated concentration of VDAC2 peptide, HTRA1 wt (30 nM) or R274Q (300 nM) was incubated with peptidic fluorescence-quenched substrate DY649P-IRRVSYSFKKC-DYQ661 (2 µM) at 37°C and fluorescence was recorded over time. Relative activity was plotted vs concentration of VDAC2-3 peptide (mean ±SD of 6 datasets). VDAC2-2 peptide was not soluble in activity assay buffer. **e**, Distance map between the α-carbons of VDAC2 peptide and HTRA1-R274Q. The main contact regions between peptide and HTRA1-R274Q (involving the N-terminal helix motif and loop LD) are shown in dark blue. The binding poses presented in Fig. 6 show that the N-terminal residues of VDAC2 peptide are sandwiched between the helix motif and loop LD. A third, less prominent, contact region is found in a region involving residues D320 to L335, containing the catalytic S328. This suggests that binding of VDAC2 peptide at the trimer tip might also affect the conformation of S328 and consequently the configuration of the active site. Source data are provided as a Source Data file.

Supplementary Figure Legends

Supplementary Fig. 1 I Proteolytic activity and oligomeric states of wt and mutant HTRA1s. a, Representative images of β-casein (20 µM) digest by wt or mutant HTRA1s (1 µM) related to Fig. 1c. Grey arrowheads: HTRA1; black arrowheads: β-casein; *: β-casein degradation fragments. Table below summarizes the relative proteolytic activities of wt and mutant HTRA1s using β-casein as substrate. The activity of wt HTRA1 was set to 100%. **b,** SEC analysis of wt and mutant HTRA1s. 10 µl of 273 µM protein (except A173T: 178 µM) was applied to Agilent AdvanceBio SEC 300 Å column and 2xPBS as mobile phase (AU: absorbance units). Table below summarizes the relative distributions of oligomeric species. **c,** SEC-MALS analysis of wt and mutant HTRA1s. 56 µl of 273 µM protein (except A173T: 178 µM) was applied on Superdex 200 Increase column with 2xPBS as mobile phase. UV, LS and dRI spectra are depicted in overlay. Table below duplicates data depicted in Fig. 1d for comparison with other methods. **d,** Sedimentation velocity AUC analysis of wt and mutant HTRA1s at 80 µM in 100 mM NaH_2PO_4, 150 mM NaCl, pH 8.0. Absorbance data are provided in Source Data. Table below summarizes the sedimentation coefficients and abundance of oligomeric species. **b-d**: 1: 1-mer; 2: 2-mer; 3: 3-mer. **b-e**: protein concentrations are given as monomer-equivalent. **b-c,** Dashed lines indicate the retention time of wt HTRA1 trimers. *: aggregates; **: degradation products or impurities. **d**, # artefact due to fitting. **e,** Native MS analysis of wt and mutant HTRA1s. Shown is relative abundance of monomers, dimers and trimers at protein concentrations ranging from to 0.5 - 25 µM (mean ±SD of 3 datasets). Representative spectra at 5 µM are depicted in Fig. 1 or in Source Data. Table below summarizes the relative distributions and MW of oligomeric species at 5 µM. Source data are provided as a Source Data file.

Supplementary Fig. 2 I Computational analysis of the effects of point mutations on HTRA1 trimer stability. a, Reference trimer interface (PDB ID 3TJO). Protomers are in green, pink and blue; relevant residues as licorice with standard atom coloring code and hydrogen atoms omitted for clarity; distances in Å. **b-c**, Molecular dynamics simulations of mutants R166H and R274Q. R166 forms an inter-protomer salt bridge with E277 (a) which is replaced by the weaker H166-E277 interaction in mutant R166H (b). Accordingly, E277 moves closer to R274, enhancing this intra-protomer interaction. R274 forms inter-protomer salt bridges with D174 and E177 (a) that are abolished by the R274Q mutation (c). **d**, Localization of A173 (grey) in the vicinity of the hydrophobic cluster (a known determinant of trimer formation) comprising Y169, F171 and F278 (left). The hydrophobic contacts within 5 Å of the residue in position 173 become less structured in the mutated system. White: hydrophobic; green: hydrophilic; blue: positively charged; red: negatively charged; tan: residue in position 173. **e**,

Free energy perturbation calculations. The values are presented per mole of trimer (corresponding to the contributions of 3 mutations). The thermodynamic cycle used to calculate the effect of point mutations on the stability of the HTRA1 trimer and free energy change as a function of couple parameter λ for the alchemical transformations are depicted in Source Data. **f-h**, Plots of selected relevant distances vs. time for S328A (f), R166H (g) and R274Q (h). **i**, Plot of the RMSD vs. time for A173T. Source data are provided as a Source Data file.

Supplementary Fig. 3 I Oligomeric states of wt and mutant HTRA1s evaluated by chemical crosslinking. a, wt or mutant HTRA1 (0.5 µM) was incubated in the absence or presence of glutaraldehyde (GA). The oligomeric states were analyzed by SDS-PAGE and silver staining. Note that GA increases the electrophoretic motility of HTRA1 monomers. **b-c**, Following incubation of R274Q (b) or wt HTRA1 (c) in the absence or presence of D174-S328A, the oligomeric states of each protein were determined via chemical crosslinking and subsequent immunoblot analysis using anti-His and Strep antibodies. Note that the anti-His antibody recognizes the Strep tag (with Strep-tagged HTRA1 running slightly lower compared to His-tagged HTRA1), however only in the uncrosslinked protein. **d**, Following incubation of HTRA1 in the absence or presence of VDAC2 peptide (50 µM), samples were analyzed as in (a).

Supplementary Fig. 4 I Functional complementation of R274Q by various D174X mutations in *cis* and impact of D174R-S328A on the activity of wt HTRA1. a, Culture medium from HEK-293T cells transfected to overexpress full-length HTRA1 was collected to assess HTRA1 protein levels (anti-Myc immunoblot, upper panel) and proteolytic activity (BSA degradation assay, lower panel). **b**, Following incubation of wt ΔNHTRA1 (1 µM) in the absence or presence of ΔND174R-S328A (3 µM), proteolytic activity was measured using β-casein (20 µM) as a substrate. Grey arrowheads: HTRA1; black arrowheads: β-casein; *: β-casein degradation fragments.

Supplementary Fig. 5 I In *trans* restoration of R274Q proteolytic activity and trimer assembly by D174R-S328A. R274Q data which are already presented in Supplementary Fig. 1 are depicted for comparison with D174R-S328A and R274Q:D174R-S328A mixtures. **a,** Representative images of β-casein (20 µM) digest by R274Q (1 µM) mixed with varying amounts of D174R-S328A related to Fig. 2b. Grey arrowheads: HTRA1; Black arrowheads: β-casein; *: β-casein degradation fragments. Table below summarizes the relative proteolytic activities of HTRA1. The activity of R274Q alone was set to 100%. **b,** SEC analysis of R274Q, D174R-S328A and R274Q mixed with D174R-S328A. 10 µl of 273 µM protein solution (except A173T: 178 µM) were applied on Agilent AdvanceBio SEC 300 Å column and 2xPBS as mobile

phase (AU: absorbance units). Table below summarizes the relative distributions of oligomeric species. **c,** SEC-MALS analysis of R274Q, D174R-S328A and R274Q mixed with D174R-S328A. 56 µl of 273 µM protein solution were applied on Superdex 200 Increase column with 2xPBS as mobile phase. UV, LS and dRI spectra are depicted in overlay. Table below summarizes the relative distributions and MW of oligomeric species (UV spectra). **d,** Sedimentation velocity AUC analysis of R274Q, D174R-S328A and R274Q mixed with D174R-S328A at 80 µM concentration in 100 mM NaH_2PO_4, 150 mM NaCl, pH 8.0. Table below summarizes the sedimentation coefficients and abundance of oligomeric species. **b-d**: 1: 1-mer; 2: 2-mer; 3: 3-mer. Protein concentrations are given as monomer-equivalent. **b-c,** Dashed lines indicate the retention time of wt HTRA1 trimers. *: aggregates; **: degradation products or impurities. **d**, # artefact due to fitting. Source data are provided as a Source Data file.

Supplementary Fig. 6 I Proteomic and RT-qPCR analyses of $Htra1^{wt}$ and $Htra1^{R274Q}$ brain vessels. a, Summary of LC–MS/MS and label-free quantification results. **b**, Volcano plot of all proteins quantified by MS. Orange circles: deregulated proteins; hyperbolic curve: permutation-based FDR estimation; proteins are labelled with gene names. **c**, HtrA1 protein levels determined by MS (left) and *htra1* mRNA levels determined by RT-qPCR (right; *Gapdh* was used as a housekeeping gene). The mean abundance in $Htra1^{wt}$ vessels was set to 1; results as box-and-whisker plots (centerline: median; limits: 25th and 75th percentile; whiskers: minimum and maximum); data points represent individual mice; significance was tested by two-sided unpaired *t*-test. **d**, Subcellular location of deregulated proteins. Proteins were categorized based on the Uniprot database. For each protein category, the number of proteins down- (left) or upregulated (right) in $Htra1^{R274Q}$ vessels was normalized to the total number of proteins quantified in this category. Extracellular matrix proteins (ECM) represent a subentity of secreted proteins. *p* values were calculated by Fisher´s exact test. **a-d**, n=5 mice per genotype, except htra1 mRNA levels in wt vessels (n=4). **e**, Overlap between $Htra1^{R274Q}$ and $Htra1^{KO}$ proteomic profiles**.** Log_2 LFQ ratio-based heatmap of proteins upregulated (p<0.05 and Log_2 LFQ ratio ≥0.4) in the cerebral vasculature of both $Htra1^{R274Q}$ and $Htra1^{KO}$ mice. Results are from the present study (1), Zellner et al., 2018[1] (2) and Kato et al., 2021[2] (3). Source data are provided as a Source Data file.

Supplementary Fig. 7 I Mass spectrometry-based analysis of distinct HtrA1 isoforms and of domain-specific changes in the abundance of Ltbp4 and Prss23. a, c, MS peptides mapped onto the respective protein sequences. The analysis of other key proteins is depicted in Source Data. **b**, MS signal of reference (Ref) HtrA1, Arg274-peptide or Gln274-peptide. LFQ values as box-and-whisker plots (centerline: median; limits: 25th and 75th percentile; whiskers: minimum and maximum) with filled circles; significance was tested by two-sided unpaired *t*-

test. MS peptide intensities as scatter plots (black bars: mean) with empty circles (nd: not detected). **a-c**, n=5 mice per genotype. The mean MS signal intensity in $Htra1^{wt}$ or $Htra1^{R274Q}$ samples was set to 1 as appropriate. Source data are provided as a Source Data file.

Supplementary Fig. 8 I Proteomic analysis of $Htra1^{wt}$ and $Htra1^{D174R\text{-}S328A}$ brain vessels. **a**, Summary of LC–MS/MS and label-free quantification results. **b**, Volcano plot of all proteins quantified by MS as in Fig. 3g. Purple circles: deregulated proteins. **c**, Subcellular location of deregulated proteins analyzed as in Supplementary Fig. 6d. *p* values were calculated by Fisher´s exact test. **a-c**, n=5 mice per genotype. Source data are provided as a Source Data file.

Supplementary Fig. 9 I Cerebrovascular proteome of $Htra1^{wt/R274Q}$ and $Htra1^{R274Q/R274Q}$ mice. Vessels from $Htra1^{wt/wt}$, $Htra1^{wt/R274Q}$ or $Htra1^{R274Q/R274Q}$ mice were analyzed by MS (**a-c,** n=5 mice per genotype; **c**, significance was tested by two-sided unpaired *t*-test), IB (**d,** vessels lysates from 3 mice per genotype), or IHC (**e,** wt: n=52 arteries from 3 mice; wt/R274Q: n=53 arteries from 4 mice; R274Q/R274Q: n=87 arteries from 4 mice; significance was tested by two-sided unpaired Mann-Whitney U-test) as in Fig. 3. **b**, Principal component analysis of protein abundance as in Fig. 4g. Filled circles represent individual mice (n=5 per genotype). Source data are provided as a Source Data file.

Supplementary Fig. 10 I Comparative analysis of $Htra1^{R274Q/D174R\text{-}S328A}$ brain vessels. a, Volcano plot of all proteins quantified by MS as in Fig. 3g. Green circles: deregulated proteins. **b**, Log_2 LFQ value-based heatmap of protein abundance changes in mouse brain vessels. The mean signal intensity in $Htra1^{wt}$ samples was set to 0; horizontal lines represent individual mice; grey: not detected. **a-b**, n=5 mice per genotype. Source data are provided as a Source Data file.

Supplementary Fig. 11 I Oligomeric states of HTRA1-R274Q in the presence of MK1 and impact of control GCP-containing compounds on the activity of HTRA1. a, HTRA1 wt and R274Q were analyzed by SEC. Overlay of UV chromatograms are depicted. The retention times of protein standards are marked by arrowheads. AU: absorbance units, *: degradation products or impurities. The chromatogram of R274Q in the presence of MK1 is not shown due to high UV absorbance of MK1 (>3000 mAU). **b**, HTRA1 wt and R274Q were analyzed by SEC followed by analysis of the eluted fractions (labelled in red in panel a) by SDS-PAGE and Coomassie staining. Where indicated, 2.5 mM MK1 was added both in the R274Q sample and to equilibrate the column. **c**, Structures of compounds. **d**, β-casein degradation by HTRA1 in the presence of control compounds. Following preincubation with the compounds indicated

(2.5 mM), 1 µM wt HTRA1 or HTRA1-R274Q were incubated with 20 µM β-casein at 37°C. Samples were taken at indicated time points and subjected to SDS-PAGE followed by Coomassie staining. Source data are provided as a Source Data file.

Supplementary Fig. 12 I Impact of peptidic modulators on the activity of HTRA1 and other proteases. a, Time-dependent degradation of Tau by the indicated HTRA1 variants in the absence or presence of the indicated peptides. Following preincubation with 50 µM of peptides, 1 µM HTRA1 was incubated with 3 µM Tau at 37°C. Samples were taken at time points indicated and subjected to SDS-PAGE followed by Coomassie staining. **b,** Selectivity of VDAC2 peptides analyzed by titration experiments. Proteases were preincubated with the peptide concentrations indicated, followed by addition of respective substrates at 37°C. Absorbance or fluorescence was recorded over time. AU: absorbance units, RFU: relative fluorescent units. Graphs depict the mean AU or RFU ±SD of 6 datapoints. The specific or relative activity estimates are provided in Source Data. Source data are provided as a Source Data file.

Supplementary Fig. 13 I Analysis of the interaction between HTRA1 and VDAC2 by native MS. a, Left panels: representative spectra of 5 µM HTRA1 S328A in the presence of 100 µM VDAC2 peptide or of 5 µM R274Q in the presence of 200 µM VDAC2 peptide. Right panels: Binding of VDAC2 peptide to HTRA1 wt trimers or to R274Q monomers. Histograms depict the average occupancy (0, 1, 2 or 3) ±SD of 3 datasets. Empty circles: individual datapoints. **b**, Titration of VDAC2 peptide to determine occupancy of HTRA1 trimers (S328A and A252T) or monomers (R166H and A173T). Graphs depict the average occupancy of peptide at HTRA1s (0, 1, 2 or 3) ±SD of 4 datasets. Source data are provided as a Source Data file.

Supplementary Fig. 14 I Impact of VDAC2 peptide on the activity HTRA1. a, Normalized cleavage rates of peptidic fluorescence-quenched substrate DY649P-IRRVSYSFKKC-DYQ661 (2 µM) by the indicated HTRA1 variants plotted vs. concentration of VDAC2 peptide. (mean ±SD of 6 datasets). Data were fitted to the weak-binding equation to yield V_{max} and K_d (black line); brackets indicate SE of the fit. **b,** Normalized cleavage rates of peptidic fluorescence-quenched substrate DY649P-IRRVSYSFKKC-DYQ661 by wt and mutant HTRA1s in absence (black circles) and in presence of fixed VDAC2 peptide concentrations (red circles) plotted vs. substrate concentrations (mean ±SD of 4 experimental data). **a-b,** Data were fitted as described in Results and Methods; brackets indicate SE of the fit. Source data are provided as a Source Data file.

Supplementary Fig. 15 I Identification of VDAC2 peptide binding site to HTRA1-R274Q. **a-b,** Docking analysis of VDAC2 and VDAC2-2 (optimized to bind the active site) peptides on a monomeric model of HTRA1-R274Q. **a**, Representation of the most favored binding sites of VDAC2 peptide at HTRA1. Red clouds indicate the main clusters of docking poses, their centroid structure is represented with red ribbons. HTRA1-R274Q is shown in blue cartoon and the residues of the catalytic triad are highlighted with sticks (licorice style). **b**, Relative populations of peptide in the active site and in the alternative site A are shown for VDAC2 and VDAC2-2 peptides. **c**, Peptide binding at the alternative site A mimics the inter-protomer interface in the trimer form. **d**, Upper panels: Time-dependent degradation of Tau by HTRA1 in the presence of VDAC2, VDAC2-2 or VDAC2-3 peptides. Following preincubation with 50 µM of the respective peptides, 1 µM wt HTRA1 or R274Q was incubated with 3 µM Tau at 37°C. Samples were taken at indicated time points and subjected to SDS-PAGE followed by Coomassie staining. Lower panels: Following preincubation with the indicated concentration of VDAC2 peptide, HTRA1 wt (30 nM) or R274Q (300 nM) was incubated with peptidic fluorescence-quenched substrate DY649P-IRRVSYSFKKC-DYQ661 (2 µM) at 37°C and fluorescence was recorded over time. Relative activity was plotted vs concentration of VDAC2-3 peptide (mean ±SD of 6 datasets). VDAC2-2 peptide was not soluble in activity assay buffer. **e**, Distance map between the α-carbons of VDAC2 peptide and HTRA1-R274Q. The main contact regions between peptide and HTRA1-R274Q (involving the N-terminal helix motif and loop LD) are shown in dark blue. The binding poses presented in Fig. 6 show that the N-terminal residues of VDAC2 peptide are sandwiched between the helix motif and loop LD. A third, less prominent, contact region is found in a region involving residues D320 to L335, containing the catalytic S328. This suggests that binding of VDAC2 peptide at the trimer tip might also affect the conformation of S328 and consequently the configuration of the active site. Source data are provided as a Source Data file.

Supplementary Tables

Supplementary Table 1. Spectrum of CARASIL-related mutations. Position of the mutations, number of afflicted pedigrees and country of origin/ethnicity of the patients. The enzymatic and oligomeric properties of the corresponding mutant proteins (where reported) are indicated. (last update: December 22)

cDNA	amino acid	number of afflicted	country of origin/ethnicity	protease activity	oligomeric state	references
**Missense mutations**						
496C>T	R166C	1	Portugal	reduced	shifted towards monomers	[3,4]
497G>A	R166H	1	France	reduced	shifted towards monomers	[5], the present work
508A>C	N170H	1	not reported	not reported	not reported	[6]
517G>T	A173S	1	Mexico	not reported	not reported	[7]
517G>A	A173T	1	Pakistan	reduced	shifted towards monomers	[4,8], the present work
523G>A	V175M	1	India	not reported	not reported	[9]
616G>A	G206R	1	Puerto Rico	not reported	not reported	[10]
754G>A	A252T	1	Japan	reduced	trimeric	[11,12], the present work
821G>A	R274Q	2	Japan, India	reduced	shifted towards monomers	[12,13,14], the present work
824C>T	P275L	1	Turkey	reduced	not reported	[15,16]
854C>T	P285L	1	China	reduced	trimeric	[12,17]
883G>A	G295R	1	Spain	reduced	shifted towards monomers	[4,18]
889G>A	V297M	2	Japan	reduced	trimeric	[11,12]
958G>A	D320N	1	China	reduced	not reported	[16,19]
961G>A	A321T	1	Roumania	reduced	trimeric	[4,20]
971A>G	N324S	1	China	not reported	not reported	[21]
1021G>A	G341R	1	China	not reported	not reported	[19]
1091T>C	L364P	1	China	reduced	trimeric	[4,22]

Continued on next page

Continued from previous page

cDNA	amino acid	number of afflicted	country of origin/ethnicity	protease activity	oligomeric state	references
**Nonsense, frameshift and intronic mutations**						
126delG	E42fs	1	Roumania	*Apart from few exceptions, the biochemical properties of the nonsense, frameshift and intronic mutations have not been addressed experimentally. Most mutations cause the loss of at least one catalytic residue. R370X induces nonsense-mediated mRNA decay*[11]		[20]
161_162insAG	G56fs	1	China			[23]
472+2 T>C	not reported	1	China			[24]
502A>T	K168X	1	India			[25]
739delG	E247fs	1	India			[25]
805insG	S270fs	1	Iran			[26]
830delA	E277fs	1	France			[5], the present work
830_831delAG	E277fs	1	India			[25]
847G>T	G283X	1	China			[27]
904C>T	R302X	2	Japan, Caucasian			[11,28]
983C>A	S328X	1	Turkey			[29]
1005+1G>T	not reported	1	Turkey			[30]
1108C>T	R370X	2	Japan, Turkey			[11,31]

See also Source Data.

Supplementary Table 2. Mass spectrometer parameters used for native MS analysis of HTRA1 and software settings for the performed UniDec data analysis.

Mass spectrometer parameters used for native MS analysis of HTRA1 mutants.	
m/z range	2000-8000
Capillary temperature [°C]	150
Microscan count	10
FT resolution	17500
AGC target	3e6
S-lens RF level	200
HCD	0
isCID	0
UHV sensor [mbar]	5.96e-10 (2.85e-10)*
Source DC Offset [V]	40
Injection Flatapole DC [V]	14
Inter Flatapole Lens [V]	4
Bent Flatapole DC [V]	7
Transfer Multipole DC Tune Offset [V]	0.5
C-Trap Entrance Lense Tune Offset [V]	0.0
* binding studies with mostly monomeric HTRA1 mutants were performed with reduced pressure in the mass analyser to stabilize the protein-ligand complex.	

Software settings for the performed UniDec data analysis.	
Data Processing:	
m/z range	2000-7000 (2500-4000 or 4500-6000)
Normalization of data	Yes
Background subtraction	0 (0 or 20)
UniDec Parameters:	
Mass range [Da]	15000-150000 (15000-100000 or 50000-120000)
Sample mass every [Da]	0.5
Peak Selection:	
Peak detection range [Da]	800 (500)
Peak detection threshold	0.01-0.10
Peak normalization	Max (Total)
Extract normalization	Sum
Additional Filtering	
Filter peak scores (minimum DScore value)	30-50%
Values in parentheses were used to analyse the data from the binding studies. Settings were used depending on the predominant complex.	

Supplementary Table 3. Sequences of oligonucleotides and guideRNAs.

Oligonucleotides

Name	Seq (5'-3')
Exon2-F	AATGATTCCCCTTCCACAGA
Exon2-R	AAGGGAATGACTCTGCAAGA
Exon4-F	CACCTTTTCCCATTTTCCTTGG
Exon4-R	CTACCCACTCACTTCAGAACCC
Exon5-F	GAGGAGGTAAAAGTGGGTTT
Exon5-R	ATACTCTCTCAAACAACCCC
ss*Htra1*_R274Q	GCTGACGATCCCAGTGGTGACTGTGTTTTGAAGAGAAAAGGGGCTTCCAATGGCAACTACGAACTCGCCAGGCTGCAGCTCTGAGGAGCGGCCGAGCAGCAGGACTGGCAGCTTTCCCTGTAAGCACAAACGGAG
ss *Htra1*_D174R	CCTTTGTCCTCAGGGCAGGAAGATCCCAACAGTTTGCGTCATAAGTACAACTTTATTGCTCGAGTGGTGGAGAAGATCGCCCCGGCTGTGGTTCACATTGAACTATATCGCAAGTAAAGAGGC
ss*Htra1*_S328A	TTGGGCGGTGGTGTGATACAGAGCCAACCTTTCCCTTTCTGTTCCGTTTCAGTATGGAAACGCCGGCGGCCCGTTAGTAAACCTGGTAAGGCTTTCCAGTATCTGTGCTGGGTTTGTTTCGTCTGTG
Htra1-F	GGCGAGGTGATTGGGATTAA
Htra1-R	TCCGTTGATGCTGATGATG
Gapdh-F	ATTGTCAGCAATGCATCCTG
Gapdh-R	ATGGACTGTGGTCATGAGCC

guideRNAs

Name	Protospacer
crRNA_R274	ATGGCAACTACAAATTCTCC
crRNA_D174	CAACTTTATTGCTGATGTGG
crRNA_S328	TTTCAGTATGGAAATTCCGG

Supplementary Table 4. X-ray data collection and refinement statistics.

	HTRA1ΔPDZ R274Q	HTRA1ΔPDZ D174R R274Q „crystal 1“	HTRA1ΔPDZ D174R R274Q „crystal 2“
PDB codes	6Z0E	6Z0Y	6Z0X
Space group	R3	$P6_3$	$P6_3$
Wavelength	1.0	0.91953	0.91504
No. xtals	1	1	1
Synchrotron	SLS	SLS	SLS
Date	24 Nov 2018	04 Jun 2019	24 Sep 2018
Detector	Pilatus 6M	Pilatus 6M	Pilatus 6M
Mol/AU	2	3	3
a, b, c (Å)	109.9, 109.9, 114.4	101.4, 101.4, 144.9	103.5, 103.5, 147.3
α, β, γ (°)	90,90,120	90,90,120	90,90,120
Resolution (Å)	43.92-2.6 (2.693-2.6)	42.33-2.2 (2.279-2.2)	44.83-3.1(3.211-3.1)
R_{sym}	53.7(229.6)	10.95 (386.4)	12.23(224.1)
R_{pim}	17.5(75.3)	2.5(85.4)	2.8(49.2)
$I / \sigma I$	10.61 (1.15)	16.27(0.87)	17.5(1.10)
CC1/2	89.5 (42.8)	99.8(35.4)	99.9(67.3)
Completeness (%)	99.87 (99.81)	99.98(100.0)	99.75(99.94)
Redundancy	10.4 (10.2)	20.6(21.5)	20.6(21.6)
**Refinement:**			
Resolution (Å)	43.92-2.6 (2.693-2.6)	42.33-2.2 (2.279-2.2)	44.83-3.1(3.211-3.1)
No. reflections	15823	42810	16252
R_{work} / R_{free}(%)	23.7/26.4	24.7/25.3 (twin refine)	22.6/27.7
**No. atoms:**			
Protein/ Ligands	2926/2	4555/20	4611/45
Water	33	8	14
Aver. B (Å^2)	59.8	73.7	143.6
**R.m.s. deviations:**			
Bond lengths (Å)	0.007	0.005	0.014
Bond angles (°)	1.04	0.74	1.69
Clashscore	4.05	0.43	1.49
**Visible residues:**			
Chain A	161-301,315-369	160-300, 315-373	161-300,313-375
Chain B	161-191,200-301,315-369	163-189, 192-302, 313-371	164-302,313-370
Chain C	-	164-301,314-372	161-301,312-370

* Values in parentheses are for highest resolution shell.

Supplementary Methods

Computational modeling

For molecular dynamics simulations of HTRA1 mutants, the structure of trimeric HTRA1 (PDB code 3TJO) was used as starting point for all calculations involving the trimer, keeping the S328A mutation in all cases. The NAMD2.9 code was used together with the well-established for biomolecular simulations force field CHARMM22 (including CMAP corrections) and the TIP3P water model[32,33,34]. Electrostatic interactions were treated with the PME method[35]. The water box used had a minimum distance of 20 Å between the system and the walls of the box. Each system was equilibrated for 30 ns prior to the 100 ns of production runs. The data from the production runs from all mutants was used for the analyses. Since the HTRA1 structures used for the simulations of each of the mutants contain three protomers, the sampling of each interface is done simultaneously during the simulations starting from different initial velocities for the residues involved in the interfaces between the protomers. Simulations were performed in the NPT ensemble with the temperature set to 300 K. Plots of selected relevant distances vs. time for S328A, R166H and R274Q are shown in Supplementary Fig. 2f-h. In the case of A173T, the plot of the RMSD vs. time is shown in Supplementary Fig. 2i. VMD 1.92 was used for visualization and analysis. The input files for the simulations as well as the final frame are provided (**Supplementary Data 4-11**).

Mutants R166H, A173T and R274Q were studied using Free energy perturbation methods[36]. The alchemical transformation was performed using NAMD2.9[32]. The relative free energy changes (ΔΔG) were calculated using the thermodynamic cycle shown as Source data. The temperature was set to 300 K. The reference state was the crystal structure (S328A mutant)[37]. The alchemical transformation from the reference state to the selected mutant was accomplished using 60 windows. In each window, 400,000 time steps of molecular dynamics simulation (including 100,000 time steps of equilibration) were performed to generate a representative ensemble. The forward and backward transformations were carried out to estimate the error in the free energy values using the BAR estimator[38]. Since the alchemical transformation must be performed also in the monomer of HTRA1, a molecular dynamics simulation of the monomer was performed to relax the structure under the same conditions as the trimer. The alchemical transformation in the monomer was also performed as described for the trimer. In the case of mutants R166H and R274Q, the correction proposed by Morgan *et al.*[39] was applied to the calculated ΔΔG. This partially accounts for the effect of using the Ewald summation approach together with the change in the charged state of the system and the finite size simulation box.

For protein - peptide docking analyses, the monomeric structure of HTRA1 was obtained from the chain A of the crystallographic structure with PDB ID 3NUM (inactive HTRA1 S328A form).

The Modeller program[40] was used to model the R274Q and A328S mutations (the later to restore the catalytic triad) as well as the missing residues in the loop regions. Subsequently, 100 ns of molecular dynamic simulation were performed to relax the structure.
Docking simulations of HTRA1 R274Q with the peptides VDAC2 (KVGLALELEA) and VDAC2.2 (KVGLYLWLKV) were performed using CABSDock[41]. The search approach comprised 30 replicas of a simulated annealing simulation that combines 20 annealing cycles with 2500 Monte Carlo steps each. The structures for the analysis are obtained every 50 Monte Carlo steps, thus leading to 1,000 structures per replica. From each replica the 100 best scored structures are extracted and combined (3,000 structures) for a cluster analysis. 30 representative docking poses were extracted, one from each group formed during the cluster analysis.
For full-atom simulated annealing for binding mode optimization, simulations were performed with NAMD 2.12[32] using the CHARMM36m forcefield[42]. The model TIP3P was used for water[33]. Counter ions were added for neutralization. Long-range electrostatics contributions were evaluated using the PME method[35]. The solvent was initially equilibrated to the box dimensions along 150 ps of NVT simulation at 300K with the protein-peptide structure fixed in the initial geometry obtained from the docking analysis. Next, 1 ns of NPT simulation was performed with harmonic constraints on the protein and ligand's atoms. Subsequently, a production run of 50 ns molecular dynamics (MD) simulation was run in the NPT ensemble. This step was replicated three times starting from different docking poses at the alternative binding site. The data from the production runs was used for further cluster analysis. Next, the most representative structure (by clustering the trajectories) along the three replicas of the MD simulations was extracted.
Following equilibration, a local optimization was performed with short cycles of a simulated annealing procedure. The simulated annealing protocol was composed of 5 cycles involving a heating ramp between 300 K – 350 K for 1 ns, then a cooling ramp back to 300 K during 1 ns followed by 1 ns of re-equilibration at 300 K. In total, the protocol involved 15 ns of optimization, in the NVT ensemble, where the system was finally re-equilibrated at 300 K.
For enhanced sampling on the HTRA1-VDAC2 bound state, Gaussian accelerated MD simulations (GaMD, 100 ns production runs) were performed for an extended sampling of the conformations of VDAC2 in the binding site[43] using NAMD 2.14 and the CHARMM36m forcefield. The calculation was initiated on the binding geometry previously optimized with the simulated annealing protocol. The biasing potential was equilibrated in two steps: first 5 ns of conventional MD in which the potential energy distribution of the system was monitored. Subsequently, the biasing potential was equilibrated by fixing an energy threshold at the maximum value of the potential energy and successively updating this value after every step of the simulation. The constant of the harmonic bias was concurrently equilibrated by keeping

the maximum standard deviation of the biasing potential at 10 kT units. The 100 ns of production runs were used for the analysis. The biasing potential creates a smoothed potential energy surface, which allows sampling dynamic events that would need hundreds of nanoseconds of conventional molecular dynamic simulations. Here, this advanced sampling technique is used to obtain a robust picture of the conserved interactions in the binding model. The VDAC2 peptide is observed at the alternative binding site in the three replicas of the GaMD simulations regardless of the different starting conditions for the production simulations. VMD 1.94 was used for visualization and analysis. The input file as well as the final frame for the GaMD simulations are provided (**Supplementary Data 12-13**). The contact map plot was created using the protein contact map script from the group of Prof. Dr. Kaluda at the University of Maryland (Min-Kang Hsieh, Protein contact map, https://user.eng.umd.edu/~jbklauda/wiki/doku.php?id=protein_contact_map).

System´s setup:

**System**	**Box dimensions X Y Z (Å)**	**Tot. number of atoms**	**Tot. number of water molecules**	**Salt conc.**
A173T (monomer)	90 90 90	68699	21808	0
A173T (trimer)	120 120 120	164679	51618	0
R274Q (monomer)	90 90 90	68703	21808	0
R274Q (trimer)	120 120 120	164694	51619	0
R166H (monomer)	90 90 90	68703	21808	0
R166H (trimer)	120 120 120	164675	51612	0
R274Q GaMD	90.46 90.46 90.46	69492	21954	0.15 M

Chemistry

All starting materials and chemicals were used as obtained from commercial suppliers. Solvents were dried and distilled before use. Column chromatography was done on columns packed with silica gel of 60 Å with a spherical size of 32–63 mm (normal phase) or as reversed phase MPLC, performed on an *Armen Instrument* Liquid Chromatography Flash machine with C18 silica gel of 120 Å with a spherical size of 5 µm. HPLC was performed on a *Dionex* System as reversed phase. As column a *YMC-ODS-AQ* was used (RP18, 150 mm length, 3 mm diameter, 5 µm spherical size, 12 Å). As mobile phase, a gradient of MeOH and H_2O 10-100% (+ 0.05% TFA each) over 30 minutes was used. The IR spectra were recorded on a JASCO FT-IR 430 spectrometer. ^{1}H- and ^{13}C-NMR spectra were recorded on a Bruker DMX 300, an AV NEO 400, a Bruker Advance III HD 600 and a Bruker DRX 500 spectrometer at ambient

temperature. The chemical shifts are reported relative to the deuterated solvent DMSO-*d*6, [D1]-$CDCl_3$ or methanol-d_4. HR-ESI-mass spectra were received by using a Bruker maXis 4G. Microwave synthesis were performed with a *CEM Discover SPS* microwave system.

BocGCP was synthesized as reported before[44].
R_f = 0.64 (SiO_2, DCM/MeOH+NEt_3, 8:2+1%); m.p. >300°C. FT-IR: ṽ = 3393 (m), 2958 (w), 1650 (s), 1542 (s), 1319 (s) cm^{-1}. ^{1}H-NMR (400 MHz, DMSO-d_6): δ = 1.08 (t, $^3J_{H-H}$ = 7.20 Hz, 9H, NEt_3-CH_3), 1.45 (s, 9H, CH_3), 2.79 (q, $^3J_{H-H}$ = 7.20 Hz, 6H, NEt_3-CH_2), 6.47 (d, $^3J_{H-H}$ = 3.64 Hz, 1H, CH), 6.77 (d, $^3J_{H-H}$ = 3.68 Hz, 1H, CH), 8.58 (br. s, 1H, NH), 9.31 (br. s, 1, NH), 10.84 (br. s, 1H, NH) ppm. ^{13}C-NMR (100 MHz, DMSO-d_6): δ = 9.7 (NEt_3-CH_3), 27.8 (CH_3), 45.2 (NEt_3-CH_2), 80.2 (C_q), 112.1, 114.1 (both CH), 128.8, 133.0, 158.5, 160.6, 163.9, 167.2 (all C_q) ppm. HR-MS (neg. ESI): *m/z* calcd. for $[C_{12}H_{15}N_4O_5-H]^-$ 295.1048, found295.1048.

BocTREN was synthesized as reported previously[45].
R_f = 0.20 (SiO_2, MeCN : H_2O : NH_4OH = 10 : 2 : 1). FT-IR ṽ = 3322 (m), 2973 (m), 2932 (m), 2816 (m), 1685 (m), 1557 (s), 1541 (s), 1521 (s), 1508 (s), 1473 (m), 1456 (m), 1391 (m), 1364 (m), 1277 (m), 1251 (m), 1171 (s), 964 (w), 752 (m), 721 (m) cm^{-1}. ^{1}H-NMR (300 MHz, [D6]DMSO) δ = 1.48 (s, 9H, CH_3), 2.47 – 2.54 (m, 6H, CH_2), 2.64 (q, 4H, $^3J_{H-H}$ = 6.1 Hz, CH_2), 3.07 (q, 2H, $^3J_{H-H}$ = 6.2 Hz, CH_2), 6.82 – 6.90 (m, 1H, NH) ppm. ^{13}C-NMR (75 MHz, [D6]DMSO) δ = 28.2 (CH_3), 53.9, 57.8 (both CH_2), 77.3, 155.6 (both C_q) ppm. HR-MS (pos. ESI) *m/z* calcd. for $[C_{11}H_{26}N_4O_2+H]^+$ 247.2129 , found 247.2140.

BocGCP-OSu: BocGCP (1.00 equiv., 5.86 mmol, 2.33 g) and *N*-hydroxysuccinimide (1.60 equiv., 9.38 mmol, 1.08 g) were suspended in DMF (60 mL) and cooled down in an ice bath while stirring. 1-Ethyl-3-(3-dimethylaminopropyl)carbodiimide hydrochloride (1.60 equiv., 9.38 mmol, 1.80 g) was added. The mixture was stirred at 0°C for 2 h and afterward at room temperature overnight. The resulting solution was poured in cold water (200 mL). The precipitate was filtered and washed with cold water. The product was lyophilized, yielding the active ester as a white solid (2.28 g, 5.80 mmol, 99%). R_f = 0.41 (SiO_2, EA : Cyclohexane = 7 : 3); m. p. 212°C (decomp.). FT-IR: ṽ = 3346 (m), 2986 (w), 1759 (s), 1721 (s), 1625 (s), 1515 (s), 1205 (s), 1135 (s), 1073 (s), 1047 (s), 918 (s) cm^{-1}. ^{1}H-NMR (300 MHz, [D6]DMSO) δ = 1.48 (s, 9H, CH_3), 2.88 (s, 4H, CH_2), 6.88 (d, 1H, CH), 7.11 (d, 1H, CH), 8.60, 9.38 (bs, 1H, NH), 10.70 (bs, 1H, NH), 12.40 (bs, 1H, NH) ppm. ^{13}C-NMR (75 MHz, [D6]DMSO) δ = 25.5 (CH_2), 27.7 (CH_3), 81.7 (C_q), 114.1, 118.6 (both CH), 126.0, 127.3, 139.0, 155.3, 158.4, 170.5 (all C_q) ppm. HR-MS (pos. ESI) *m/z* calcd. for $[C_{16}H_{19}N_5O_7+H]^+$ 394.1357, found 394.1357.

$(BocGCP)_2BocTREN$: BocGCP-OSu (2.10 equiv., 0.89 mmol, 350 mg) and BocTREN (1.00 equiv., 0.42 mmol, 104 mg) were solved in DMF (20 mL) and stirred at room temperature overnight. The reaction mixture was poured in cold water (100 mL) and the resulting precipitate was filtered and washed with cold water. The remaining solid was solved in ethyl acetate and washed with brine three times. The organic phase was separated and dried over $MgSO_4$. The solvent was removed under reduced pressure. After adding water (10 mL) to the resulting oil, the resulting suspension was lyophilized yielding the product as a white solid (313 mg, 0.39 mmol, 92%).

R_f = 30.5 min (HPLC (RP18) 10–100% MeOH for 35 min; >90 %); m. p. 137°C (decomp.). FT-IR $\tilde{\nu}$ =3384 (w), 2978 (w), 1717 (m), 1625 (s), 1557 (s), 1472 (m), 1456 (m), 1292 (s), 1240 (s), 1146 (s), 1046 (w), 926 (w), 842 (m), 780 (m), 755 (m) cm^{-1}. ^{1}H-NMR (300 MHz, [D6]DMSO) δ = 1.32 (s, 9H, CH_3), 1.44 (s, 18H, CH_3), 2.53 (m, 2H, CH_2), 2.63 (m, 4H, CH_2), 2.96 (m, 2H, CH_2), 3.31 (m, 4H, CH_2), 6.57 (br, 1H, NH), 6.79 (d, 2H, CH), 8.31 (t, 2H, NH), 8.54 (bs, 2H, NH), 9.30 (bs, 2H, NH), 10.85 (bs, 2H, NH), 11.46 (bs, 2H, NH) ppm. ^{13}C-NMR (75 MHz, [D6]DMSO) δ = 27.7 (CH_3), 28.1 (CH_3), 37.2, 38.2, 53.6, 53.7 (all CH_2), 77.5 (C_q), 111.8, 113.7 (both CH), 129.5, 155.5, 158.3, 159.8 (all C_q) ppm. HR-MS (pos. ESI) *m/z* calcd. for $[C_{35}H_{54}N_{12}O_{10}+H]^+$ 803.4159, found 803.4161, calcd. For $[C_{35}H_{54}N_{12}O_{10}+Na]^+$ 825.3978, found 825.3974.

$(GCP)_2TREN$ · 3 TFA (MK1): (BocGCP)2BocTREN (1.00 equiv., 0.29 mmol, 234 mg) was suspended in DCM (5 mL). Under virtuous stirring, concentrated TFA (5 mL) was added stirring for 1 h at room temperature. The solvent and the acid were removed under reduced pressure. After adding water (15 mL) to the remaining oil, the crude was lyophilized overnight, yielding in a grey solid. The crude product was purified via MPLC (RP-18, 10-30% MeOH + 0.05% TFA for 30 min, 30-50% for 15 min, 50-100% for 2 min, 100% isocratic for 15 min). The fractions containing the product were united and the solvents were removed, yielding in MK1 as a white solid (145 mg, 0.17 mmol, 59%).

R_f = 8.8 min (HPLC (RP18) 10–100% MeOH+0.05 % TFA for 35 min; 96 %); m. p. 135°C (decomp.). FT-IR $\tilde{\nu}$ = 3328 (m), 1667 (s), 1558 (s), 1541 (s), 1435 (m), 1282 (m), 1256 (m), 1193 (s), 1129 (s), 1007 (w), 839 (m), 799 (m), 750 (m), 723 (s) cm^{-1}. ^{1}H-NMR (300 MHz, [D6]DMSO) δ = 2.72 (m, 6H, CH), 2.88 (m, 2H, CH_2), 3.34 (m, 4H, CH_2), 6.84 (d, 2H, CH), 7.09 (d, 2H, CH), 7.67 (bs, 3H, NH_3), 8.38 (br. m., 10H, 4xNH_2 and NH), 11.23 (bs, 2H, NH), 12.30 (bs, 2H, NH) ppm. ^{13}C-NMR (125 MHz, [D6]DMSO) δ = 36.7, 51.0, 53.1 (all CH_2), 112.4, 115.4 (both CH), 116.7 (CF_3, $^1J_{C\text{-}F}$ = 297.2 Hz), 125.5, 132.6, 155.1 (all C_q), 158.6 (COO^-, $^2J_{C\text{-}F}$ = 32.7 Hz), 159.5, 159.7 (both C_q) ppm. HR-MS (pos. ESI) *m/z* calcd. for $[C_{20}H_{30}N_{12}O_6+H]^+$ 503.25857, found 503.25860, calcd. for $[C_{20}H_{30}N_{12}O_6+2H]^{2+}$ 252.13293, found 252.13274.

$(Gly)_2BocTREN$: N_α-Cbz-Glycine (4.00 equiv., 2.39 mmol, 500 mg) and HATU (4.00 equiv., 2.39 mmol, 908 mg) were dissolved in DMF (10 mL) and was stirred virtuously. After DIPEA (16.5 equiv., 9.84 mmol, 1.68 mL) was added, the solution was stirred for 30 min. BocTREN (1.00 equiv., 0.60 mmol, 147 mg) was added and the reaction mixture was stirred in the microwave system at 20 W and 40°C for 90 min. The solution was stirred at room temperature for additional 48 h. Water (50 mL) was added the solution and the resulting precipitate was extracted with ethyl acetate (75 mL). The combined organic phase was washed with brine and dried over $MgSO_4$. The solvent was removed and the residue was solved in methanol. A catalytic amount of Pd/C was added and the reaction mixture was stirred under a hydrogen atmosphere overnight. The catalyst was filtered through a celite pad and washed with methanol. The solvent was removed and the resulting crude was purified via MPLC (RP-18, 30-70% MeOH for 35 min, 70-100% for 15 min, 100% isocratic for 10 min). The united fractions were lyophilized, yielding in the product as a yellow, sticky oil (121 mg, 0.34 mmol, 56%).
R_f = 12.8 min (HPLC (RP18) 10–100% MeOH for 35 min; 84 %); FT-IR $\tilde{v}$ = 3303 (m), 2974 (m), 2932 (m), 2825 (m), 1684 (s), 1651 (s), 1524 (s), 1456 (m), 1391 (m), 1364 (m), 1271 (m), 1251 (s), 1165 (s), 1065 (w), 970 (w), 862 (w) cm^{-1}. ^{1}H-NMR (300 MHz, [D6]DMSO) δ = 1.36 (s, 9H, CH_3), 2.44 – 2.51 (m, 6H, CH_2), 2.95 (m, 2H, CH_2), 3.08 – 3.13 (m, 8H, CH_2), 6.62 (br, 1H, NH), 7.80 (br, 2H, NH) ppm. ^{13}C-NMR (75 MHz, [D6]DMSO) δ = 28.2 (CH_3), 36.6, 38.3, 44.6, 53.3, 53.4 (all CH_2), 77.5, 155.6, 172.5 (all C_q) ppm. HR-MS (pos. ESI) *m/z* calcd. for $[C_{15}H_{32}N_6O_4+H]^+$ 361.2558, found 361.2561, calcd. for $[C_{15}H_{32}N_6O_4+Na]^+$ 383.2377, found 383.2378.

$(BocGCP\text{-}Gly)_2BocTREN$: GCP (2.05 equiv., 0.70 mmol, 278 mg) and HCTU (2.05 equiv., 0.70 mmol, 290 mg) were dissolved in DMF (10 mL) and was stirred virtuously. After NMM (4.38 equiv., 1.49 mmol, 166 µL) was added, the solution was stirred for 30 min. $(Gly)_2BocTREN$ (1.00 equiv., 0.34 mmol, 121 mg) was added and the reaction mixture was stirred for 24 h. The reaction mixture was poured in water (50 mL) and the resulting precipitate was filtered and washed with cold water. Drying yielded in a pale white solid (102 mg, 0.11 mmol, 32%).
R_f = 29.2 min (HPLC (RP18) 10–100% MeOH for 35 min; 89 %); m. p. 157°C (decomp.). FT-IR $\tilde{v}$ = 3837 (m), 3748 (m), 3673 (m), 3310 (m), 2974 (w), 2937 (w), 2163 (w), 2031 (w), 1631 (s), 1539 (s), 1459 (m), 1394 (m), 1368 (m), 1339 (m), 1289 (s), 1237 (s), 1144 (s), 1047 (w), 841 (m), 755 (m) cm^{-1}. ^{1}H-NMR (300 MHz, [D6]DMSO) δ = 1.36 (s, 9H, CH_3), 1.45 (s, 18H, CH_3), 2.43 – 2.50 (m, 6H, CH_2), 2.93 (q, 2H, $^3J_{H\text{-}H}$ = 6.0 Hz, CH_2), 3.11 (q, 4H, $^3J_{H\text{-}H}$ = 6.0 Hz, CH_2), 3.83 (d, 4H, $^2J_{H\text{-}H}$ = 5.7 Hz, CH_2), 6.67 (t, 1H, $^3J_{H\text{-}H}$ = 5.3 Hz, NH), 6.80 (s, 4H, CH), 7.85 (t, 2H, $^3J_{H\text{-}H}$ = 5.4 Hz, NH), 8.55 (bs, 2H, NH), 8.64 (t, 2H, $^3J_{H\text{-}H}$ = 5.7 Hz, NH), 9.31 (bs, 2H, NH), 10.82 (bs, 2H, NH), 11.31 (bs, 1H, NH) ppm. ^{13}C-NMR (125 MHz, [D6] DMSO) δ = 27.8

(CH_3), 28.2 (CH_3), 37.0, 38.3, 42.1, 53.3, 53.8 (all CH_2), 77.6 (C_q), 112.2 (CH), 155.7, 158.4, 159.9, 168.8 (all C_q) ppm. HR-MS (pos. ESI) *m/z* calcd. for $[C_{39}H_{60}N_{14}O_{12}+H]^+$ 917.4588, found 917.4610.

$(GCP\text{-}Gly)_2$TREN · 3TFA (MK2): Boc deprotection of ((BocGCP-Gly)$_2$BocTREN) was obtained similarly to MK1, yielding in MK2 as a white solid (102 mg, 0.11 mmol, 98%).
R_f = 10.9 min (HPLC (RP18) 10–100% MeOH+0.05 % TFA for 35 min; >94 %); m. p. 144°C (decomp.). FT-IR $\tilde{v}$ = 3312 (s), 1651 (s), 1557 (s), 1541 (s), 1473 (m), 1434 (m), 1286 (m), 1254 (m), 1195 (s), 1129 (s), 836 (m), 800 (m), 750 (m), 721 (s) cm^{-1}. ^{1}H-NMR (300 MHz, [D6] DMSO) δ = 2.58 (m, 4H, CH_2), 2.64 (m, 2H, CH_2), 2.84 (m, 2H, CH_2), 3.15 (m, 4H, CH_2), 3.86 (d, 4H, CH_2), 6.89 (d, 2H, CH), 7.10 (d, 2H, CH), 7.57 (bs, 3H, NH_3^+), 7.96 (t, 2H, NH), 8.40 (bs, 8H, NH_2), 8.81 (t, 2H, NH), 11.28 (bs, 2H, NH), 12.38 (bs, 2H, NH) ppm. ^{13}C-NMR (125 MHz, [D6] DMSO) δ = 36.8, 37.1, 42.3, 51.0, 53.2 (all CH_2), 112.8, 115.3 (both CH), 117.2 (CF_3, $^1J_{C\text{-}F}$ = 300.1 Hz), 125.6, 132.3, 155.0 (all C_q), 158.2 (COO^-, $^2J_{C\text{-}F}$ = 30.9 Hz), 159.6, 159.9 (both C_q) ppm. HR-MS (pos. ESI) *m/z* calcd. for $[C_{24}H_{36}N_{14}O_6+H]^+$ 617.30150, found 617.30197, calcd. for $[C_{20}H_{30}N_{12}O_6+2H]^{2+}$, found 309.15433, calcd. for $[C_{20}H_{30}N_{12}O_6+3H]^{3+}$ 206.43868, found 206.43845.

$(Cbz)_2$BocTREN: BocTREN (1.00 equiv., 1.34 mmol, 0.330 g) and Cbz-OSu (3.00 equiv., 4.01 mmol, 1.00 g) were dissolved in DCM (80 mL) and stirred for 4 h. Afterwards 50 mL of water were added and the aqueous phase was extracted three times with 60 mL DCM. The combined organic layer was dried over $MgSO_4$ and the solvent was removed under reduced pressure. The crude product was obtained as yellow oil (0.630 g, 1.22 mmol, 92%) and used without further purification.
^{1}H NMR (400 MHz, [D6] DMSO) δ = 7.35 – 7.32 (m, 10H), 7.15 (t, *J* = 5.7 Hz, 2H), 6.72 (t, *J* = 4.8 Hz, 1H), 5.00 (s, 4H), 3.02 (q, *J* = 6.2 Hz, 4H), 2.92 (d, *J* = 6.2 Hz, 2H), 2.47 – 2.45 (m, 4H), 1.35 (s, 9H) ppm. ^{13}C NMR (101 MHz, [D6] DMSO) δ = 156.43, 155.91, 128.50, 127.99, 127.89, 67.12, 65.62, 31.36, 28.27 ppm.

$(Cbz)_2$BocGCPTREN: $(Cbz)_2$BocTREN (1.00 equiv., 1.22 mmol, 0.63 g) were suspended in a mixture of MeOH and DCM (1:1). After addition of 5 mL concentrated HCl the reaction mixture was stirred for 3.5 h. Afterwards the solvent was removed under reduced pressure and the residue was dissolved in DMF (30 mL). Then triethylamine (1.4 eq, 1.71 mmol, 0.24 mL) and BocGCP-OSu (1.00 equiv., 1.22 mmol, 0.48 g) were added and the resulting mixture was stirred overnight. The reaction mixture was poured into water (150 mL) and the aqueous layer was extracted five times with 30 mL DCM. The combined organic layer was washed with a saturated aqueous solution of NaCl (60 mL). Afterwards the organic layer was dried over

$MgSO_4$ and the solvent was removed under reduced pressure. The product was obtained as yellow solid (0.46 g, 0.66 mmol, 54%) and used without further purification.
^{1}H NMR (400 MHz, [D6] DMSO) δ = 12.05 (s, 1H), 10.82 (s, 1H), 9.36 (s, 1H), 8.52 (s, 1H), 8.23 (t, J = 5.9 Hz, 1H), 7.38 – 7.24 (m, 10H), 7.14 (t, J = 5.6 Hz, 2H), 6.76 (s, 2H), 5.00 (s, 4H), 3.27 (q, J = 6.5 Hz, 2H), 3.06 (q, J = 6.4 Hz, 4H), 2.63 – 2.50 (m, 6H), 1.46 (s, 9H) ppmz. ^{13}C NMR (101 MHz, [D6] DMSO) δ = 156.22, 137.23, 128.32, 127.72, 127.68, 65.18, 54.92, 53.64, 53.23, 27.77 ppm. HR-MS (pos. ESI) *m/z* calcd. for $[C_{34}H_{44}N_8O_8+H]^+$ 693.3355, found 693.3356.

GCPTREN · 3 TFA (TNMK009): $(Cbz)_2$BocGCPTREN (1.00 equiv., 0.27 mmol, 0.19 g) was dissolved in MeOH (30 mL) and 10%wt Pd/C were added. The resulting suspension was stirred vigorously under H_2 atmosphere overnight. Then the suspension was filtered and the filtrate was treated with 5 mL concentrated TFA and stirred for 5 h. The solvent was removed under reduced pressure and the crude product was purified via MPLC (RP-18, 5% → 100% MeOH + 0.05% TFA for 90 min) yielding the product as white solid (0.11 g, 0.17 mmol, 36%).
R_f = 7.7 min (HPLC (RP18), 10-100% Meoh+0.05% TFA for 30 min). m. p. 118°C. FT-IR ṽ = 3334.32, 3315.03, 2994.91, 2898.49, 2838.70, 2360.44, 2342.12, 1772.26, 1737.55, 1716.34, 1697.05, 1680.66, 1636.30, 1556.27, 1523.49, 1508.06, 1486.85, 1436.71, 1372.10, 1339.32, 1287.25, 1257.36, 1132.97, 1005.70, 943.02, 890.95, 841.78, 812.85, 794.53, 765.60, 740.53, 722.21, 702.93, 669.18, 656.64, 600.72, 534.19, 514.90, 493.69, 482.12, 471.51, 461.87, 444.51, 432.94, 413.66, 404.98. ^{1}H NMR (400 MHz, [D6] DMSO) δ = 12.33 (s, 1H), 11.36 (s, 1H), 8.65 – 8.18 (s, 5H), 7.71 (s, 6H), 7.14 (d, J = 5.1, 2.6 Hz, 1H), 6.85 (dd, J = 4.1, 2.3 Hz, 1H), 3.35 (q, J = 6.5 Hz, 2H), 2.90 (s, 4H), 2.71 (m, 4H), 2.68 – 2.59 (m, 2H) ppm. HR-MS (pos. ESI) *m/z* calcd. for $[C_{13}H_{27}N_8O_2+H]^+$: 325.2095, found 325.2100.

$(Ac)_2$TREN: TREN (1.00 equiv., 9.80 mmol, 1.43 g, 1.47 mL) and triethylamine (1.8 equiv., 17.63 mmol, 2.44 mL) were dissolved in DCM (20 mL). Afterwards a solution of acetic anhydride (1.00 equiv., 9.80 mmol, 1.00 g, 0.92 mL) in 20 mL DCM was added dropwise over 1 h. After stirring for 1 h the solution was filtered and the filtrate was concentrated in vacuum. The crude product was purified by column chromatography (SiO_2, MeOH:NH_3, 10:1). The product was obtained as slightly yellow oil (0.64 g, 2.8 mmol, 28%).
R_f = 0.56 (SiO_2, MeOH:NH_3, 10:1). FT-IR ṽ = 3628.41, 3278.39, 3100.97, 2980.45, 2834.85, 2360.44, 2342.12, 1624.73, 1556.27, 1433.82, 1374.03, 1296.89, 1203.36, 1168.65, 1105.98, 1064.51, 1040.41, 997.02, 962.31, 669.18, 602.65, 557.33, 518.76, 508.15, 471.51, 443.55, 420.41. ^{1}H NMR (400 MHz, [D6] DMSO) δ = 7.80 (t, J = 5.5 Hz, 2H), 3.06 (q, J = 6.2 Hz, 4H), 2.46 – 2.36 (m, 6H), 1.80 (s, 6H) ppm (two protons missing caused by overlapping with DMSO signal). ^{13}C NMR (101 MHz, [D6] DMSO) δ = 169.18, 57.41, 53.70, 37.11, 22.63 ppm. HR-MS

(pos. ESI) *m/z* calcd. for $[C_{10}H_{22}N_4O_2+H]^+$ 231.1816, found 231.1838, calcd. for $[C_{10}H_{22}N_4O_2+Na]^+$ 253.1635, found 253.1649.

$(Ac)_2$GCPTREN · TFA (TNMK027): GCP-OSu (1.00 equiv., 0.39 mmol, 0.15 g) was dissolved in DCM (30 mL) and added to a solution of $(Ac)_2$TREN (2.00 equiv., 0.78 mmol, 0.18 g) in DMF (10 mL). The resulting solution was stirred overnight. Afterwards the reaction mixture was poured into water (160 mL). The aqueous phase was extracted three times with 20 mL DCM. The combined organic phases were washed with a saturated aqueous solution of NaCl (60 mL) and dried over $MgSO_4$. After filtration 20 mL of concentrated TFA were added to the filtrate and the resulting solution was stirred for 4 h. The solvent was removed under reduced pressure and the crude product was purified via MPLC (RP-18, 5% → 100% MeOH + 0.05% TFA for 90 min). The product was obtained as white solid (0.03 g, 0.06 mmol, 15%).
R_f = 8.9 min (HPLC (RP18), 10-100% MeOH+0.05% TFA for 30 min). m. p. 177°C (decomp.). FT-IR ṽ = 3344.93, 3233.07, 3127.01, 3080.73, 2361.41, 2342.12, 1725.98, 1682.59, 1644.98, 1628.59, 1569.77, 1541.81, 1480.10, 1448.28 ,1434.78, 1417.42, 1396.21, 1378.85, 1361.5, 1346.07, 1328.71, 1312.32, 1295.93, 1283.39, 1258.32, 1235.18, 1199.51, 1183.11, 1166.72, 1109.83, 1044.26, 1024.98, 1001.84, 953.63, 900.59, 888.06, 867.81, 826.35, 794.53, 769.46, 718.35, 631.57, 609.40, 576.61, 543.83, 531.29, 515.87, 495.62, 434.87, 404.98. ^{1}H NMR (400 MHz, [D6] DMSO) δ =z 12.39 (s, 1H), 11.47 (s, 1H), 9.77 (s, 1H), 8.84 – 8.37 (m, 5H), 8.23 (t, *J* = 5.8 Hz, 2H), 7.17 (dd, *J* = 4.2, 2.3 Hz, 1H), 6.88 (dd, *J* = 4.1, 2.3 Hz, 1H), 3.62 (q, *J* = 6.1 Hz, 2H), 3.47 – 3.21 (m, 10H), 1.84 (s, 6H). ^{13}C NMR (101 MHz, [D6] DMSO) δ = 170.75, 159.96, 159.72, 155.19, 132.05, 125.83, 115.37, 112.72, 52.21, 51.59, 33.74, 22.44 ppm. HR-MS (pos. ESI) *m/z* calcd. for $[C_{17}H_{28}N_8O_4+H]^+$: 409.2306, found 409.2303, calcd. for $[C_{17}H_{28}N_8O_4+Na]^+$ 431.2126, found 431.2123, calcd. for $[C_{17}H_{28}N_8O_4+2H]^{2+}$ 205.1190, found 205.1212.

N-Boc-Hydrazine was synthesized as described[46]. Instead of isopropanol, MeCN (200 mL) were used. Hydrazine monohydrate (5.06 equiv., 0.93 mol, 45 mL), NaOH (5.50 equiv., 1.01 mol, 40.32 g in 200 mL water) and Boc_2O (1.00 equiv., 0.18 mol, 40.00 g) were used, yielding in a white solid (20.00 g, 0.15 mol, 83%). R_f = 0.67 (9:1 DCM/MeOH); m. p. 42 – 45°C. FT-IR: ṽ = 3371, 3324, 2978, 2933, 1693 cm^{-1}. ^{1}H-NMR (300 MHz, $CDCl_3$) δ = 6.52 (s, 1H, NH), 3.69 (s, 2H, NH_2), 1.35 (s, 9H, CH_3) ppm. ^{13}C-NMR (75 MHz, $CDCl_3$) δ = 28.5 (CH_3), 77.2, 158.3 (both C_q) ppm.

GCP(Boc)-Lys(Cbz)-Lys(Boc)-OH synthesis was reported before[47]. The resin (2 g) was loaded with Fmoc-Lys(Boc)-OH (2.00 equiv., 1.45 mg, 3.1 mmol) and DIPEA (4.00 equiv., 1.08 mL, 6.2 mmol) as described above. Fmoc-Lys(Cbz)-OH (2.00 equiv., 1.2 g, 3.1 mmol), GCP (2.00

equiv, 1.23 g, 3.1 mmol), HCTU (2.00 equiv., 1.28 g, 3.1 mmol) and DIPEA (4.00 equiv., 1.08 mL, 6.2 mmol) were used. Purification via MPLC yielded in a white solid (2.63 mg, 3.34 mmol, 91%). R_f = 0.64 (DCM/MeOH 9:1); m. p. 90°C (decomp.), FT-IR: ṽ = 3311, 2938, 2843, 1664, 1637 cm^{-1}. ^{1}H-NMR (300 MHz, DMSO-d_6) δ = 1.53 – 1.18 (m, 26H, CH_2 and CH_3), 1.79 – 1.53 (m, 4H, CH_2), 2.92 – 2.78 (m, 2H, CH_2), 3.09 – 2.92 (m, 2H, CH_2), 4.18 – 4.07 (m, 1H, CH), 4.54 – 4.41 (m, 1H, CH), 4.98 (s, 2H, CH), 6.80 – 6.70 (m, 1H, NH), 6.89 (d, $^3J_{H-H}$ = 3.9 Hz, 1H, CH), 6.96 (d, $^3J_{H-H}$ = 3.9 Hz, 1H, CH), 7.21 (t, $^3J_{H-H}$ = 5.6 Hz, 1H, NH), 7.37 – 7.25 (m, 5H, CH), 8.23 (d, $^3J_{H-H}$ = 7.6 Hz, 1H, NH), 8.46 (d, $^3J_{H-H}$ = 8.0 Hz, 1H, NH), 12.19 (s, 1H, NH) ppm. ^{13}C-NMR (75 MHz, DMSO-d_6) δ = 22.8, 27.6 (both CH_2), 28.2, 29.1 (both CH_3), 29.1, 30.6, 31.7 (all CH_2), 51.8, 52.5 (both CH), 65.0, 77.3, 82.8 (all C_q), 113.3, 114.6, 127.6 (all CH), 128.3 (C_q), 131.2 (CH), 137.2, 155.5, 155.8, 156.0, 158.3, 158.8, 159.0, 171.8, 173.4 (all C_q) ppm. HR-MS (pos. ESI) *m/z* calcd. for $[C_{37}H_{54}N_8O_{11}+H]^+$: 787.3985, found 787.4093.

GCP-Lys(Cbz)-Lys-OH · 2 TFA (AZ25) synthesis was reported before[47]. In a 100 ml round bottom flask, GCP-Lys(Cbz)-Lys(Boc)-OH (1.00 equiv., 1.27 mmol, 0.38 g) was dissolved in TFA/DCM (10 mL 1:1). The reaction mixture was stirred for 1 h at room temperature. The solvents were removed. under reduced pressure. The crude product was purified via MPLC, yielding in a white solid (0.10 g, 0.13 mmol, 27%). R_f = 0.5 (DCM/MeOH 4:1); m. p. 82 – 85°C. FT-IR: ṽ = 3317, 2941, 2873, 1662 cm^{-1}. ^{1}H-NMR (600 MHz, DMSO-d_6) δ = 1.79 – 1.28 (m, 12H, CH_2), 2.81 – 2.72 (m, 2H, CH_2), 3.02 – 2.93 (m, 2H, CH_2), 4.20 – 4.14 (m, 1H, CH), 4.50 – 4.44 (m, 1H, CH), 4.98 (s, 2H, CH_2), 6.92 – 6.87 (m, 1H, CH), 7.18 – 7.14 (m, 1H, CH), 7.24 (t, $^3J_{H-H}$ = 5.6 Hz, 1H, NH), 7.37 – 7.27 (m, 5H, CH), 7.72 (s, 3H, NH), 8.31 (d, $^3J_{H-H}$ = 7.8 Hz, 1H, NH), 8.57 – 8.42 (m, 3H, NH), 8.66 (s, 2H, NH), 11.45 (s, 1H, NH), 12.55 (s, 1H, NH) ppm. ^{13}C-NMR (150 MHz, DMSO-d_6) δ = 22.4, 22.9, 26.5, 29.2, 30.3, 31.8 (all CH_2), 38.7 (CH), 40.1, 40.3 (both CH_2), 51.6, 52.6 (both CH), 65.1, 113.7, 115.0 (all C_q), 117.0 (CF_3, $^1J_{C-F}$ = 300.4 Hz), 125.6, 127.8, 128.4, 132.4, 137.3, 155.2, 156.1 (all C_q), 158.7 (COO^-, $^2J_{C-F}$ = 30.7 Hz), 158.8, 159.9, 171.8, 173.4 (all C_q) ppm. HR-MS (pos. ESI) *m/z* calcd. for $[C_{27}H_{26}N_8O_7+H]^+$: 587.2936, found 587.3138.

GCP-Lys(Cbz)-Lys-hydrazine (AZ21): Into a 100 ml round bottom flask, GCP-Lys(Z)-Lys(Boc)-OH (1.00 equiv., 1.27 mmol, 1.00 g) and PyBOP (1.10 equiv., 0.73 mmol, 0.73 g) were dissolved in DCM (60 mL). Subsequently, *N*-Boc-hydrazine (1.01 equiv., 1.29 mmol, 0.17 g) and DIPEA (2.94 equiv., 0.48 mmol, 0.65 mL) were added to the reaction solution. The reaction solution was stirred at room temperature overnight. After the reaction time, the turbid solution was filtered. The filter cake was washed with DCM and then dried. The solid was then dissolved in TFA / DCM (10 mL 1:1). The reaction mixture was stirred for 1 h at room temperature. The solvents were removed under reduced pressure. The crude product was purified via MPLC,

yielding in a white solid (0.31 g, 0.17 mmol, 25%). R_f = 0.17 (DCM/MeOH 9:1); m. p. 74 – 77°C. FT-IR: $\tilde{v}$ = 3319, 2947, 2877, 1664 cm^{-1}. ^{1}H-NMR (600 MHz, DMSO-d_6) δ = 1.75 – 1.23 (m, 12H, CH_2), 2.79 – 2.72 (m, 2H, CH_2), 3.02 – 2,94 (m, 2H, CH_2), 4.31 – 4.24 (m, $^3J_{H-H}$ = 8.4 Hz, 1H, CH), 4.47 – 4.40 (m, 1H, CH), 4.98 (s, 2H, CH_2), 6.92 – 6.88 (m, 1H, CH), 7.22 – 7.18 (m, 1H, CH), 7.26 (t, $^3J_{H-H}$ = 5.6 Hz, 1H, NH), 7.37 – 7.28 (m, 5H, CH), 8.39 (d, $^3J_{H-H}$ = 7.6 Hz, 1H), 8.64 – 8.49 (m, 3H, NH), 8.81 (s, 2H, NH), 10.74 (s, 1H, NH), 11.59 (s, 1H, NH), 12.51 (s, 1H, NH) ppm. ^{13}C-NMR (150 MHz, DMSO-d_6) δ = 22.2, 23.0, 26.5, 29.2, 31.0, 31.6 (all CH_2), 50.9, 52.9 (both CH), 65.1, 113.7, 115.0 (all C_q), 116.7(CF_3, $^1J_{C-F}$ = 300.4 Hz), 125.7, 127.7, 127.8, 128.4 (all CH), 132.2, 137.3, 155.3, 156.1(all C_q), 158.9 (COO^-, $^2J_{C-F}$ = 30.6 Hz), 159.0, 160.0, 171.0, 172.0 (all C_q) ppm. HR-MS (pos. ESI) *m/z* calcd. for $[C_{27}H_{40}N_{10}O_6+H]^+$: 601.3205, found 601.3312.

GCP(Boc)-Ala-Lys(GCP)-Lys-Ala-Lys(Boc)-Ala-OH (HXY23): The resin (750 mg) was loaded with Fmoc-Ala-OH (2.00 equiv., 750 mg, 2.4 mmol) and DIPEA (4.00 equiv., 0.85 mL, 4.8 mmol) as described above. In each of the following steps, HCTU (2.00 equiv., 994 mg, 2.4 mmol) and DIPEA (4.00 equiv., 0.85 mL, 4.8 mmol) were used. The peptide was built as follow: Fmoc-Lys(Boc)-OH (2.00 equiv., 1.12 g, 2.4 mmol), Fmoc-Ala-OH (2.00 equiv., 750 mg, 2.4 mmol), Fmoc-Lys(Alloc)-OH (2.00 equiv., 1.09 g, 2.4 mmol) and Fmoc-Ala-OH (2.00 equiv., 750 mg, 2.4 mmol). Alloc deprotection was performed by stirring the resin in DCM (5 mL) for 10 min with additional Pd(Ph3P)4 (0.1 equiv., 0.06 mmol, 64.5 mg) and PhSiH3 (12 equiv., 14.4 mmol, 1.8 mL). The solution was removed by suction. Finally, GCP(Boc) (2.67 equiv., 3.2 mmol, 1.28 g) was coupled with HCTU (2.67 equiv., 1.33 g, 3.2 mmol) and DIPEA (5.33 equiv., 1.13 mL, 6.4 mmol). Separation from the resin and purification via MPLC yielded in a white solid (0.50 g, 0.53 mmol, 44%). ^{1}H NMR (300 MHz, DMSO-d_6) δ 12.48 (s, 1H), 12.28 (s, 1H), 11.18 (brs, 2H), 8.56 (brd, *J* = 8.3 Hz, 1H), 8.51-8.17 (m, 8H), 8.10 (brd, *J* = 7.7 Hz, 2H), 7.95-7.75 (m, 2H), 7.06 (brs, 2H), 6.90-6.85 (m, 2H), 6.71 (brs, 1H), 4.57-4.40 (m, 1H), 4.34-4.10 (m, 4H), 3.24-3.19 (m, 2H), 2.90-2.83 (m, 2H), 1.80-1.09 (m, 30H). HR-MS (pos. ESI) m/z calcd. for [C40H61N15O12+H]+: 944.4697, found 944.4748, calcd. for [C40H61N15O12+Na]+: 966.4516, found 966.4508.

Supplementary References

1. Zellner A*, et al.* CADASIL brain vessels show a HTRA1 loss-of-function profile. *Acta Neuropathol* **136**, 111-125 (2018).
2. Kato T*, et al.* Candesartan prevents arteriopathy progression in cerebral autosomal recessive arteriopathy with subcortical infarcts and leukoencephalopathy model. *J Clin Invest* **131**, (2021).
3. Menezes Cordeiro I*, et al.* Shifting the CARASIL paradigm: report of a non-Asian family and literature review. *Stroke* **46**, 1110-1112 (2015).
4. Uemura M*, et al.* HTRA1 Mutations Identified in Symptomatic Carriers Have the Property of Interfering the Trimer-Dependent Activation Cascade. *Front Neurol* **10**, 693 (2019).
5. E. Tournier-Lasserve lab, University of Paris, INSERM U1141, AP-HP Saint-Louis Hospital, Paris, France.
6. Wen L*, et al.* Case Report: Diffuse Cerebral Microbleeds in Cerebral Autosomal Recessive Arteriopathy With Subcortical Infarcts and Leukoencephalopathy. *Front Neurol* **13**, 818332 (2022).
7. Shang T, Pinho M, Ray D, Khera A. Two Unique Mutations in HTRA1-Related Cerebral Small Vessel Disease in North America and Africa and Literature Review. *J Stroke Cerebrovasc Dis* **30**, 106029 (2021).
8. Khaleeli Z*, et al.* A novel HTRA1 exon 2 mutation causes loss of protease activity in a Pakistani CARASIL patient. *J Neurol* **262**, 1369-1372 (2015).
9. Khandelwal D, Mathur V, Vyas A, Ghunawat J, Bagaria AK. CARASIL - A Review of Patients from India. *Neurol India* **69**, 1359-1362 (2021).
10. Ibrahimi M*, et al.* A CARASIL Patient from Americas with Novel Mutation and Atypical Features: Case Presentation and Literature Review. *Cerebrovasc Dis* **44**, 135-140 (2017).
11. Hara K*, et al.* Association of HTRA1 mutations and familial ischemic cerebral small-vessel disease. *N Engl J Med* **360**, 1729-1739 (2009).
12. Nozaki H*, et al.* Distinct molecular mechanisms of HTRA1 mutants in manifesting heterozygotes with CARASIL. *Neurology* **86**, 1964-1974 (2016).
13. Nishimoto Y*, et al.* A novel mutation in the HTRA1 gene causes CARASIL without alopecia. *Neurology* **76**, 1353-1355 (2011).
14. Badachi S, John SK, Sekhar M, Mathew T. CARASIL; The Backache, Baldness, Brain Attack Syndrome: The Indian Scenario. *Ann Indian Acad Neurol* **23**, 559-561 (2020).
15. Bekircan-Kurt CE*, et al.* One Disease with two Faces: Semidominant Inheritance of a Novel HTRA1 Mutation in a Consanguineous Family. *J Stroke Cerebrovasc Dis* **30**, 105997 (2021).
16. Malik R*, et al.* Whole-exome sequencing reveals a role of HTRA1 and EGFL8 in brain white matter hyperintensities. *Brain* **144**, 2670-2682 (2021).
17. Chen Y, He Z, Meng S, Li L, Yang H, Zhang X. A novel mutation of the high-temperature requirement A serine peptidase 1 (HTRA1) gene in a Chinese family with cerebral autosomal recessive arteriopathy with subcortical infarcts and leukoencephalopathy (CARASIL). *J Int Med Res* **41**, 1445-1455 (2013).
18. Mendioroz M*, et al.* A missense HTRA1 mutation expands CARASIL syndrome to the Caucasian population. *Neurology* **75**, 2033-2035 (2010).

19. Xie F, Zhang LS. A Chinese CARASIL Patient Caused by Novel Compound Heterozygous Mutations in HTRA1. *J Stroke Cerebrovasc Dis* **27**, 2840-2842 (2018).

20. Bianchi S*, et al.* Two novel HTRA1 mutations in a European CARASIL patient. *Neurology* **82**, 898-900 (2014).

21. Hou MM, Mao XW, Liu XB, Liu YQ, Bi XY, Hou XJ. Novel c.971A>G mutation in the HTRA1 gene in a Chinese family with CARASIL. *J Biol Regul Homeost Agents* **34**, 1407-1410 (2020).

22. Wang XL, Li CF, Guo HW, Cao BZ. A novel mutation in the HTRA1 gene identified in Chinese CARASIL pedigree. *CNS Neurosci Ther* **18**, 867-869 (2012).

23. Cai B*, et al.* A frameshift mutation in HTRA1 expands CARASIL syndrome and peripheral small arterial disease to the Chinese population. *Neurol Sci* **36**, 1387-1391 (2015).

24. Sun D, Tian F, Zhang S, Zhang M. A new Chinese family with HTRA1 mutation associated with CARASIL. *Neurol Sci* **43**, 4577-4579 (2022).

25. Preethish-Kumar V*, et al.* CARASIL families from India with 3 novel null mutations in the HTRA1 gene. *Neurology* **89**, 2392-2394 (2017).

26. Ziaei A*, et al.* Novel mutation in HTRA1 in a family with diffuse white matter lesions and inflammatory features. *Neurol Genet* **5**, e345 (2019).

27. Yu Z*, et al.* Genetically Confirmed CARASIL: Case Report with Novel HTRA1 Mutation and Literature Review. *World Neurosurg* **143**, 121-128 (2020).

28. Tan RY*, et al.* Cerebral autosomal recessive arteriopathy with subcortical infarcts and leukoencephalopathy (CARASIL). *Pract Neurol* **21**, 448-451 (2021).

29. Gunduz T, Demirkol Y, Dogan O, Demir S, Akcakaya NH. A Case of Leukoencephalopathy and Small Vessels Disease Caused by a Novel HTRA1 Homozygous Mutation. *J Stroke Cerebrovasc Dis* **28**, 104354 (2019).

30. Roeben B, Uhrig S, Bender B, Synofzik M. Teaching NeuroImages: When alopecia and disk herniations meet vascular leukoencephalopathy: CARASIL. *Neurology* **86**, e166-e167 (2016).

31. Bayrakli F, Balaban H, Gurelik M, Hizmetli S, Topaktas S. Mutation in the HTRA1 gene in a patient with degenerated spine as a component of CARASIL syndrome. *Turk Neurosurg* **24**, 67-69 (2014).

32. Phillips JC*, et al.* Scalable molecular dynamics with NAMD. *J Comput Chem* **26**, 1781-1802 (2005).

33. Jorgensen WL, Chandrasekhar J, Madura JD, Impey RW, Klein ML. Comparison of simple potential functions for simulating liquid water. *J Chem Phys* **79**, 926-935 (1983).

34. Mackerell AD, Jr., Feig M, Brooks CL, 3rd. Extending the treatment of backbone energetics in protein force fields: limitations of gas-phase quantum mechanics in reproducing protein conformational distributions in molecular dynamics simulations. *J Comput Chem* **25**, 1400-1415 (2004).

35. Essmann U, Perera L, Berkowitz ML, Darden T, Lee H, Pedersen LG. A smooth particle mesh Ewald method. *Journal of Chemical Physics* **103**, 8577-8593 (1995).

36. Zwanzig RW. High-temperature equation of state by a perturbation method .1. Nonpolar gases. *J Chem Phys* **22**, 1420-1426 (1954).

37. Eigenbrot C*, et al.* Structural and functional analysis of HtrA1 and its subdomains. *Structure* **20**, 1040-1050 (2012).

38. Bennett CH. Efficient estimation of free-energy differences from Monte-Carlo data. *Journal of Computational Physics* **22**, 245-268 (1976).

39. Morgan BR, Massi F. Accurate Estimates of Free Energy Changes in Charge Mutations. *J Chem Theory Comput* **6**, 1884-1893 (2010).

40. Sali A, Blundell TL. Comparative protein modelling by satisfaction of spatial restraints. *J Mol Biol* **234**, 779-815 (1993).

41. Kurcinski M, Jamroz M, Blaszczyk M, Kolinski A, Kmiecik S. CABS-dock web server for the flexible docking of peptides to proteins without prior knowledge of the binding site. *Nucleic Acids Res* **43**, W419-424 (2015).

42. Huang J*, et al.* CHARMM36m: an improved force field for folded and intrinsically disordered proteins. *Nat Methods* **14**, 71-73 (2017).

43. Pang YT, Miao Y, Wang Y, McCammon JA. Gaussian Accelerated Molecular Dynamics in NAMD. *J Chem Theory Comput* **13**, 9-19 (2017).

44. Schmuck C*, et al.* A Facile and Efficient Multi-Gram Synthesis of N-Protected 5-(Guanidinocarbonyl)-1H-pyrrole-2-carboxylic Acids. *Eur J Org Chem*, 324-329 (2008).

45. DiVittorio KM, Hofmann FT, Johnson JR, Abu-Esba L, Smith BD. Facilitated phospholipid translocation in vesicles and nucleated cells using synthetic small molecule scramblases. *Bioorg Med Chem* **17**, 141-148 (2009).

46. Melendez RE, Lubell WD. Aza-amino acid scan for rapid identification of secondary structure based on the application of N-Boc-aza(1)-dipeptides in peptide synthesis. *J Am Chem Soc* **126**, 6759-6764 (2004).

47. Hoing A*, et al.* A Bivalent Supramolecular GCP Ligand Enables Blocking of the Taspase1/Importin alpha Interaction. *ChemMedChem* **17**, e202100640 (2022).

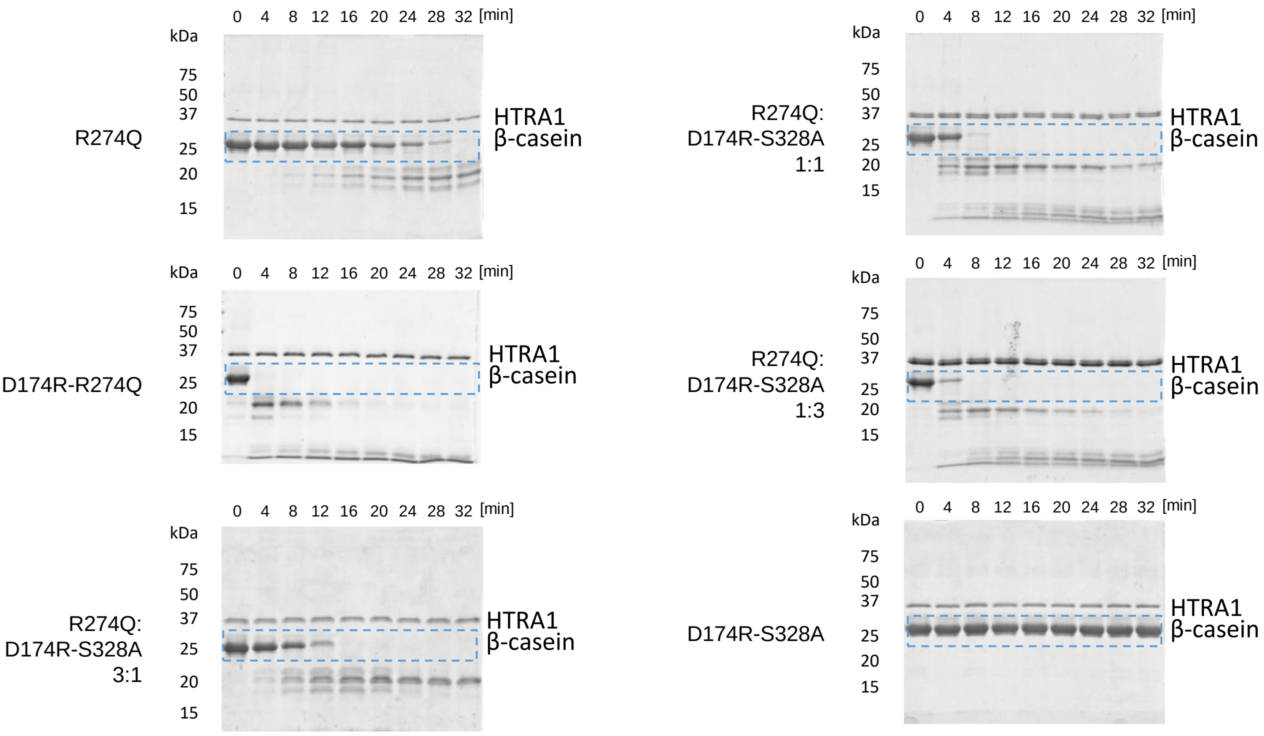

Uncropped gels related to Fig. 2b. Activity (β-casein degradation) of R274Q, D174R-R274Q, D174R-S328A and R274Q mixed at increasing molar ratios, and D174R-S328A. Uncropped gels are presented. The portions of the gels presented in Fig. 2b are marked by blue dashed rectangles.

IB anti-Ltbp4

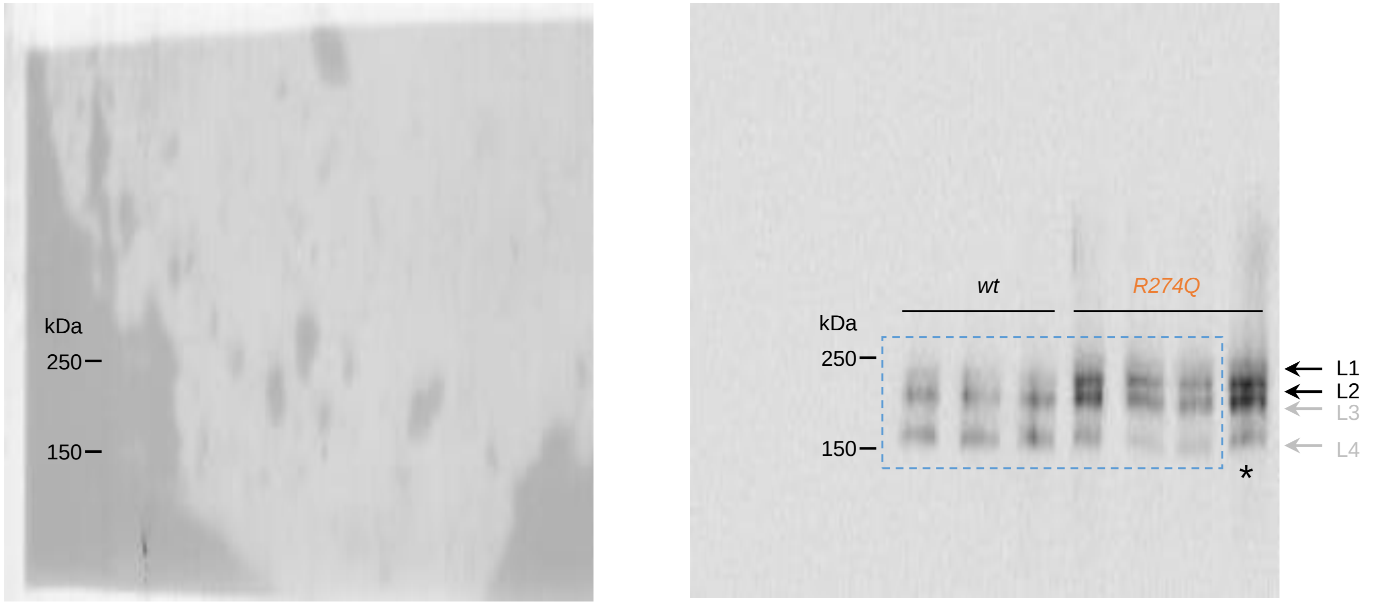

IB anti-actin (reblot after IB anti-Fbln5 – not depicted)

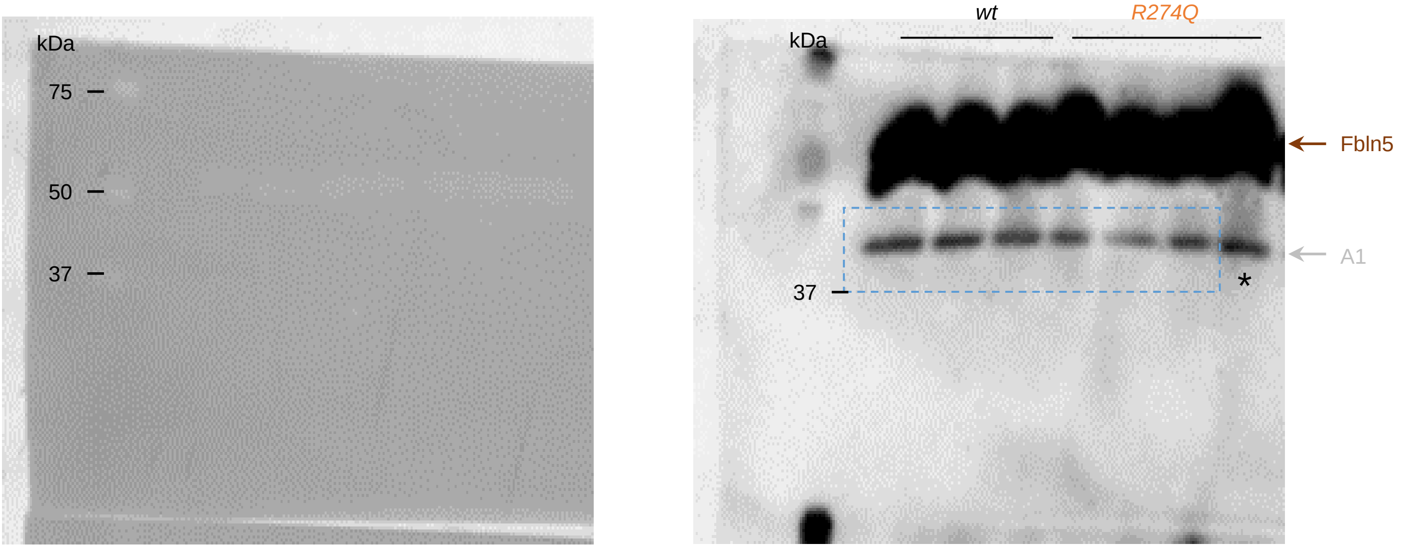

Uncropped blots related to Fig. 3d. Ltbp4 was detected by immunoblot in mouse brain vessels. Actin serves as loading control. *: sample not depicted in Fig. 3d. The portions of the blots presented in Fig. 3d are marked by blue dashed rectangles.

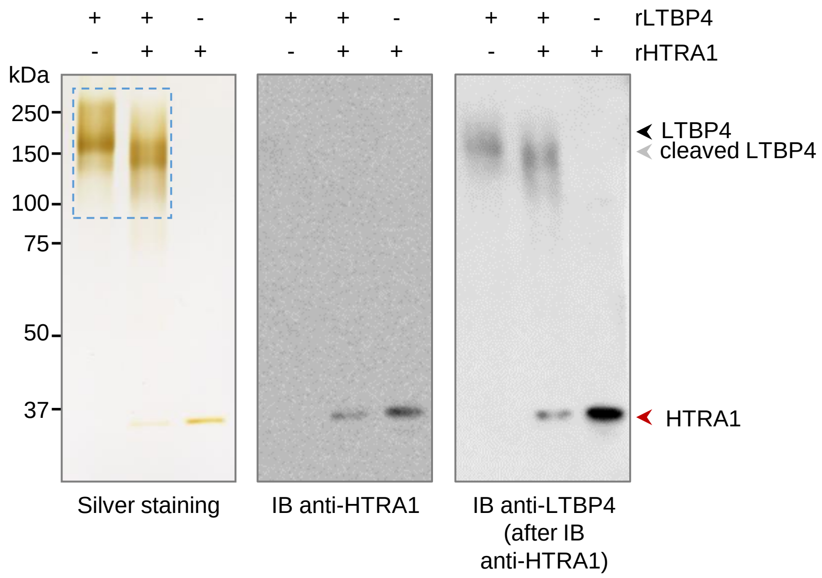

Uncropped gel related to Fig. 3e. Recombinant human LTBP4s was exposed to recombinant human HTRA1. Samples were analyzed by SDS-PAGE and silver staining or by anti-HTRA1 and anti-LTBP4 IB. The portion of the gel presented in Fig. 3e is marked by blue dashed rectangle.

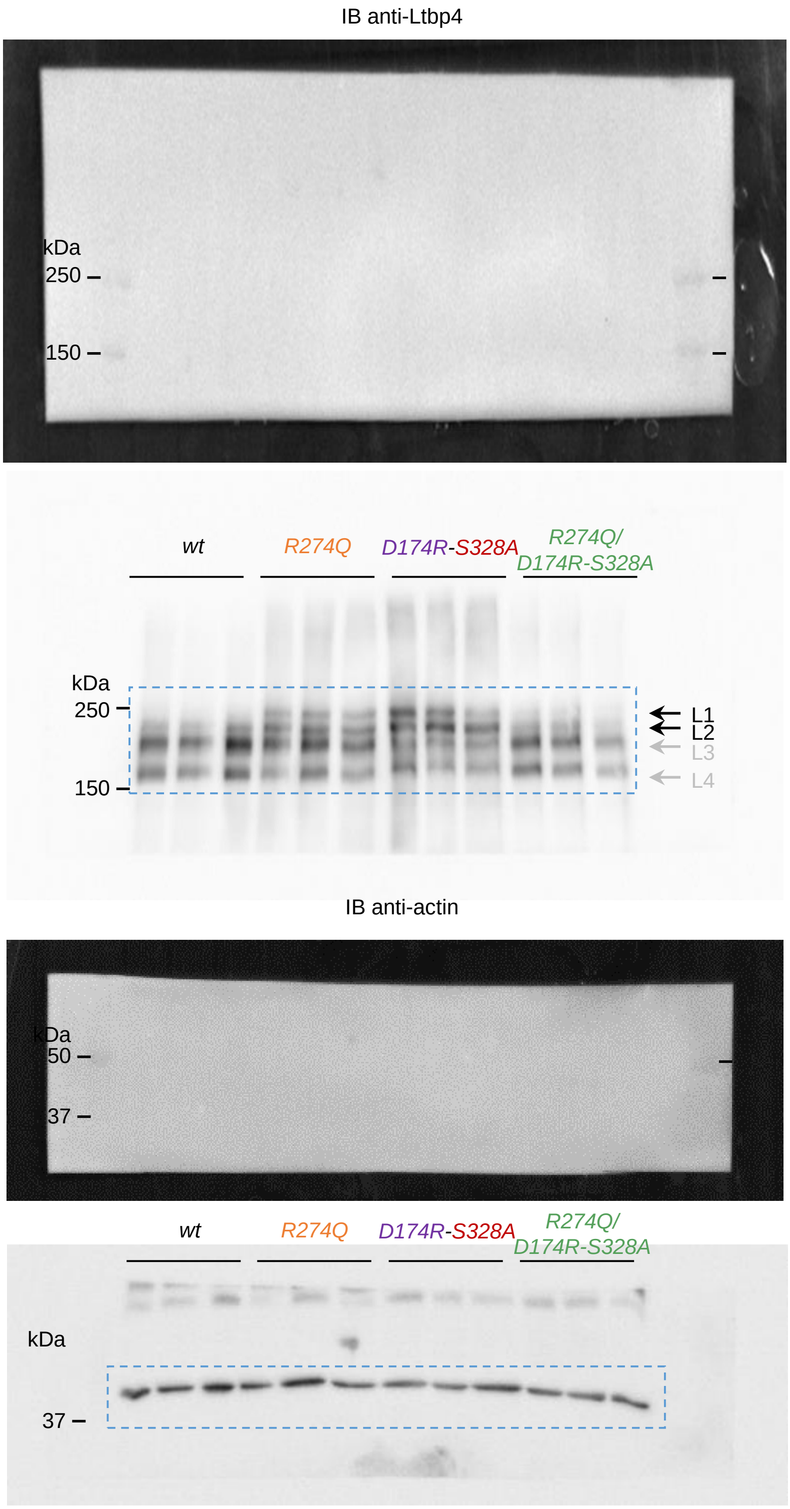

Uncropped blots related to Fig. 4e. Ltbp4 was detected by immunoblot in mouse brain vessels. Actin serves as loading control. The portions of the blots presented in Fig. 4e are marked by blue dashed rectangles.

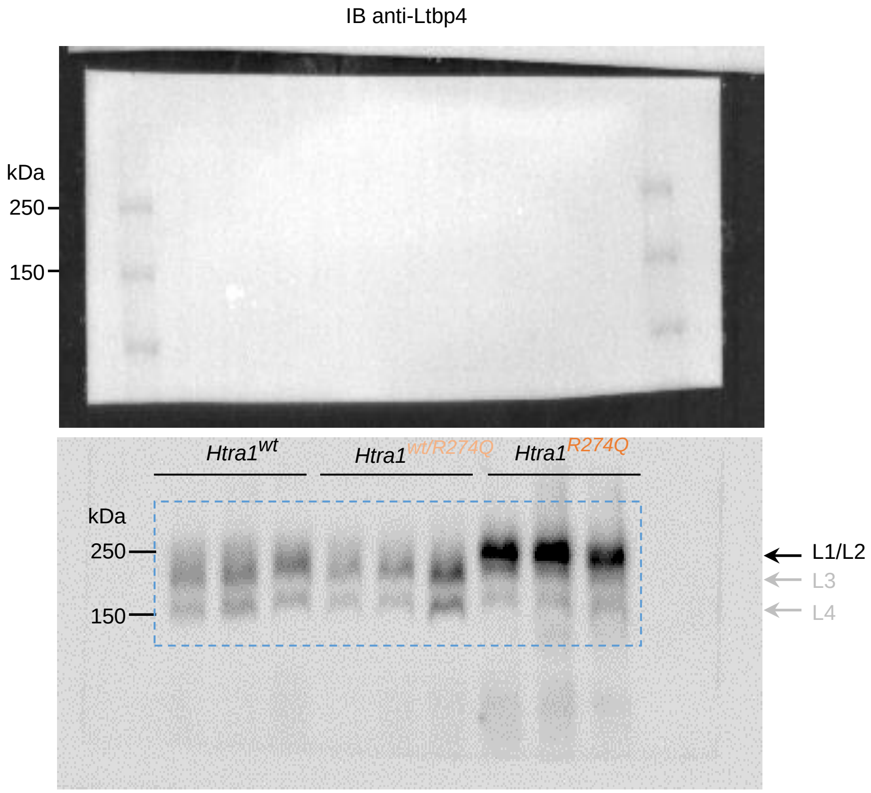

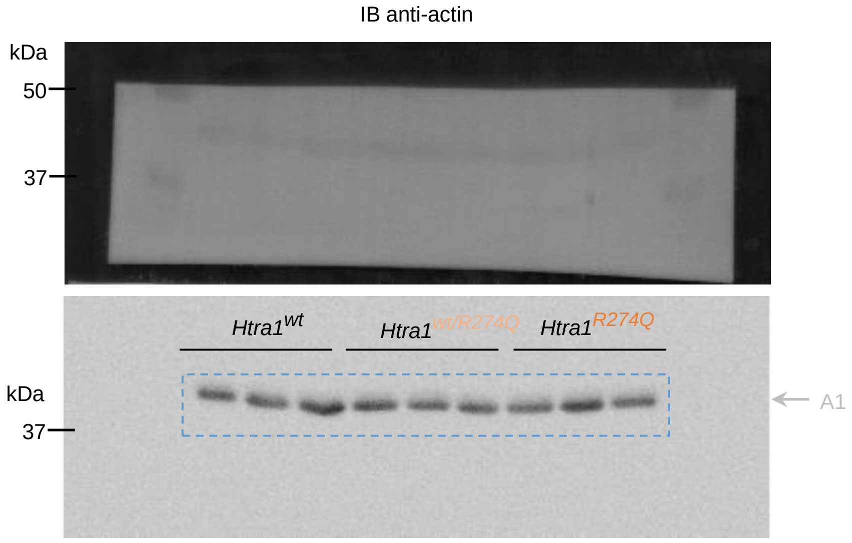

Uncropped blots related to Supplementary Fig. 9d. Ltbp4 was detected by immunoblot in mouse brain vessels. Actin served as a loading control. The portions of the blots presented in Supplementary Fig. 9d are marked by blue dashed rectangles.

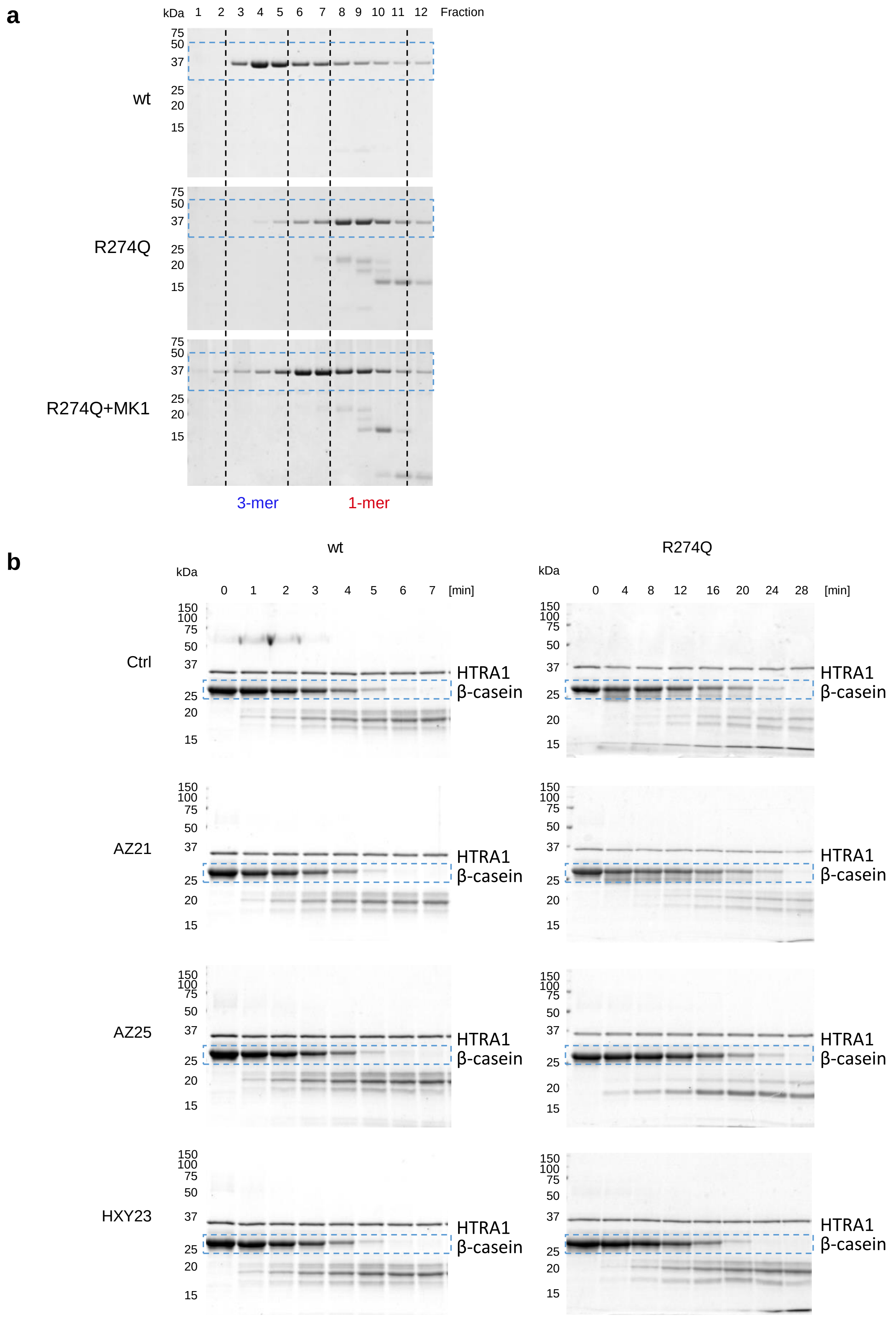

Uncropped gels related to Supplementary Fig. 11b and d. a, SEC elution fractions analyzed by SDS-PAGE and Coomassie staining. Uncropped gels are presented. The portions of the gels presented in Supplementary Fig. 11b are marked by blue dashed rectangles. **b,** Activity (β-casein degradation) of HTRA1 in the absence (Ctrl) or presence of control GCP compounds. Uncropped gels are presented. The portions of the gels presented in Supplementary Fig. 11d are marked by blue dashed rectangles.

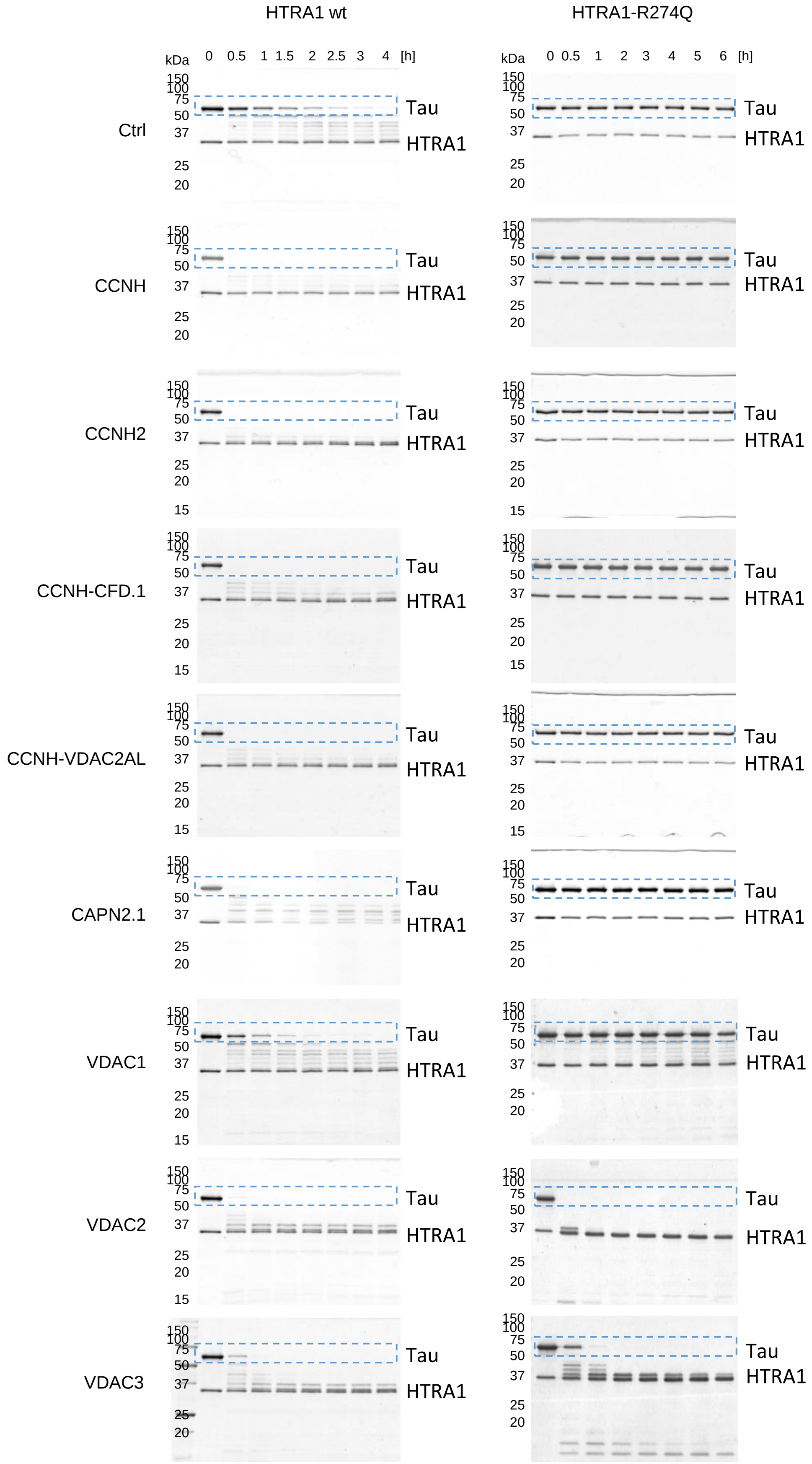

continued on next slide

Uncropped gels related to Supplementary Fig. 12a. Time-dependent degradation of Tau by the indicated HTRA1 species in the absence (Ctrl) or presence of the indicated peptides. Uncropped gels are presented. The portions of the gels presented in Supplementary Fig. 12a are marked by blue dashed rectangles.

continued from previous slide

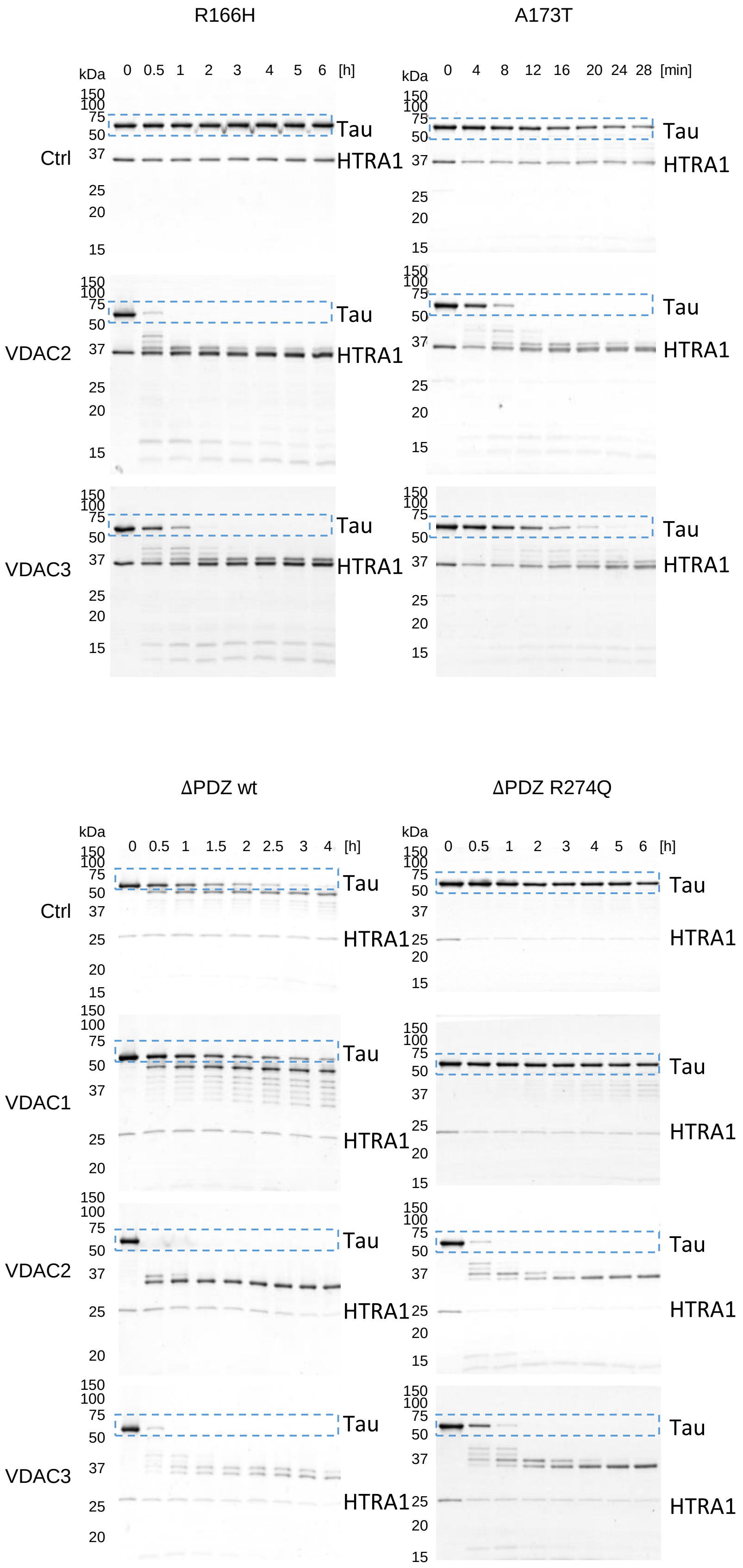

Uncropped gels related to Supplementary Fig. 12a.

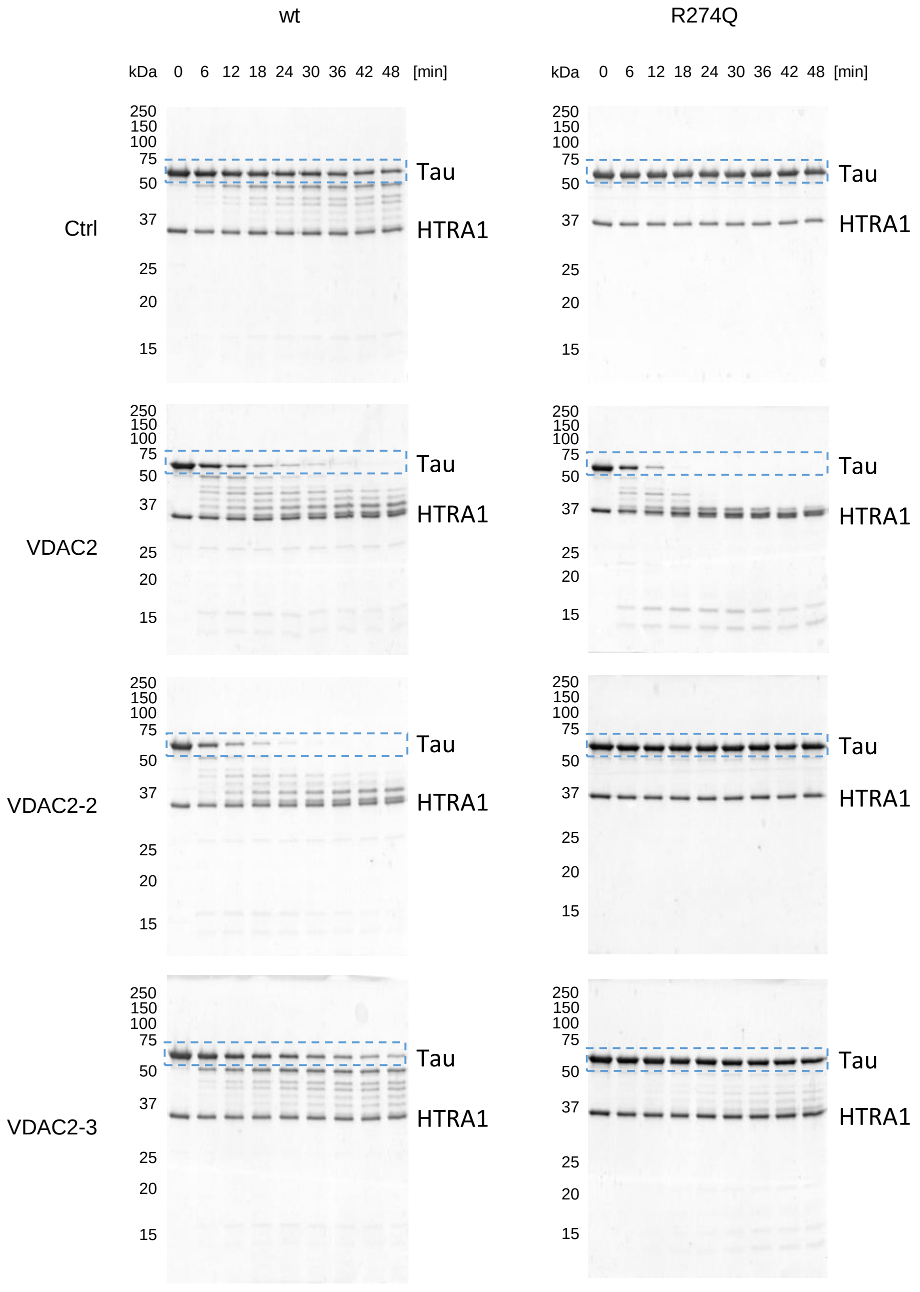

Uncropped gels related to Supplementary Fig. 15d. Time-dependent degradation of Tau by HTRA1 in the absence (Ctrl) or presence of the indicated peptides. The portions of the gels presented in Supplementary Fig. 15d are marked by blue dashed rectangles.
