# Supplementary material for: Rational correction of pathogenic conformational defects in HTRA1
Source: Nat Commun. 2024 Jul 16;15:5944. doi: 10.1038/s41467-024-49982-8 (PMC11252331; doi:10.1038/s41467-024-49982-8)
Supplement: Supplementary file 3 — Description of Additional Supplementary Files [file 41467_2024_49982_MOESM3_ESM.pdf]

Description of Additional Supplementary Files

Supplementary Data 1: Label-free quantification of mouse brain vessel proteome - reference protein sequences.

The raw data were searched against a modified, reviewed isoform FASTA database of *Mus musculus*.

Label free quantification (LFQ) required at least 2 unique or razor peptides. Generated LFQ values were log2 transformed and log2 ratios were calculated for inter genotype comparison. Corresponding p-values (two-sided unpaired *t*-test) were calculated and are additionally depicted as –log10 transformed values. n = 5 mice / genotype. HtrA1, Ltbp4 and Prss23 are highlighted in orange. The other proteins of interest are highlighted in yellow (see text for explanations).

Supplementary Data 2: Analysis of individual tryptic peptides.

The raw data were searched against a modified, reviewed isoform FASTA database of *Mus musculus* implemented with the sequences of HtrA1-R274Q and HtrA1-D174R-S328A. n = 5 mice / genotype.

Subtab "HtrA1": Unique peptides matching with the reference sequence of HtrA1 or with the sequences of HtrA1-R274Q or HtrA1-D174R-S328A are listed. Peptides containing Arg174, Arg274 or Gln274 are highlighted in yellow.

Subtabs "Ltbp4" and "Prss23": Unique peptides matching with the reference sequences of Ltbp4 or Prss23 are listed. Peptides matching with the aminoterminal region of Ltbp4 (aa 1-143) or with the carboxyterminal region of Prss23 (aa 50-382) are highlighted in yellow.

Subtab "Other POIs": Unique peptides matching with the reference sequences of the other proteins of interest (see text for explanations) are listed.

Supplementary Data 3: Label-free quantification of mouse brain vessel proteome - custom protein sequences.

The raw data were searched against a modified, reviewed isoform FASTA database of *Mus musculus*. The canonical HtrA1 sequence was replaced by the Pan-HtrA1 sequence (excluding aa 274); Ltbp4 was replaced with two sequences, which separately depict its N-

and C-terminal parts (aa 1-143 and aa 144-1,666, respectively); Prss23 sequence was shortened (aa 50-382) to only include its C-terminal part.
Label free quantification (LFQ) required at least 2 unique or razor peptides. Generated LFQ values were log2 transformed and log2 ratios were calculated for inter genotype comparison. Corresponding p-values (two-sided unpaired *t*-test) were calculated and are additionally depicted as –log10 transformed values. n = 5 mice / genotype. Pan-HtrA1, Ltbp4-Nt and Prss23-Ct are highlighted in orange. The other proteins of interest (including Ltbp4-Ct) are highlighted in yellow (see text for explanations).

Supplementary Data 4: Input file for the MD simulations of S328A.

Supplementary Data 5: Final frame for the MD simulations of S328A.

Supplementary Data 6: Input file for the MD simulations of R166H.

Supplementary Data 7: Final frame for the MD simulations of R166H.

Supplementary Data 8: Input file for the MD simulations of R274Q.

Supplementary Data 9: Final frame for the MD simulations of R274Q.

Supplementary Data 10: Input file for the MD simulations of A173T.

Supplementary Data 11: Final frame for the MD simulations of A173T.

Supplementary Data 12: Input file of the GaMD simulations of R274Q-VDAC2.

Supplementary Data 13: Final frame of the GaMD simulations of R274Q-VDAC2.
