# Supplementary material for: Rational correction of pathogenic conformational defects in HTRA1
Source: Nat Commun. 2024 Jul 16;15:5944. doi: 10.1038/s41467-024-49982-8 (PMC11252331; doi:10.1038/s41467-024-49982-8)
Supplement: Supplementary file 16 — Supplementary Data 13 [file 41467_2024_49982_MOESM16_ESM.pdf]

```
ATOM      1  N    GLY P   1       3.936 -19.380   8.669  0.00  0.00      PROA
ATOM      2  HT1  GLY P   1       3.290 -19.159   7.884  0.00  0.00      PROA
ATOM      3  HT2  GLY P   1       3.882 -20.401   8.863  0.00  0.00      PROA
ATOM      4  HT3  GLY P   1       4.891 -19.011   8.488  0.00  0.00      PROA
ATOM      5  CA   GLY P   1       3.433 -18.571   9.803  0.00  0.00      PROA
ATOM      6  HA1  GLY P   1       3.981 -18.870  10.684  0.00  0.00      PROA
ATOM      7  HA2  GLY P   1       2.400 -18.778  10.038  0.00  0.00      PROA
ATOM      8  C    GLY P   1       3.845 -17.012   9.630  0.00  0.00      PROA
ATOM      9  O    GLY P   1       3.548 -16.410   8.577  0.00  0.00      PROA
ATOM     10  N    GLN P   2       4.657 -16.445  10.601  0.00  0.00      PROA
ATOM     11  HN   GLN P   2       4.776 -16.885  11.487  0.00  0.00      PROA
ATOM     12  CA   GLN P   2       5.098 -15.034  10.507  0.00  0.00      PROA
ATOM     13  HA   GLN P   2       5.399 -14.895   9.479  0.00  0.00      PROA
ATOM     14  CB   GLN P   2       3.913 -14.060  10.714  0.00  0.00      PROA
ATOM     15  HB1  GLN P   2       3.276 -14.465   9.899  0.00  0.00      PROA
ATOM     16  HB2  GLN P   2       4.245 -13.052  10.384  0.00  0.00      PROA
ATOM     17  CG   GLN P   2       3.164 -14.019  12.085  0.00  0.00      PROA
ATOM     18  HG1  GLN P   2       3.780 -13.297  12.664  0.00  0.00      PROA
ATOM     19  HG2  GLN P   2       2.974 -15.011  12.547  0.00  0.00      PROA
ATOM     20  CD   GLN P   2       1.767 -13.351  11.985  0.00  0.00      PROA
ATOM     21  OE1  GLN P   2       0.848 -13.704  12.707  0.00  0.00      PROA
ATOM     22  NE2  GLN P   2       1.552 -12.353  11.147  0.00  0.00      PROA
ATOM     23 HE21 GLN P   2       0.650 -11.924  11.200  0.00  0.00      PROA
ATOM     24 HE22 GLN P   2       2.239 -11.793  10.685  0.00  0.00      PROA
ATOM     25  C    GLN P   2       6.303 -14.696  11.409  0.00  0.00      PROA
ATOM     26  O    GLN P   2       6.824 -13.554  11.400  0.00  0.00      PROA
ATOM     27  N    GLU P   3       6.922 -15.641  12.209  0.00  0.00      PROA
ATOM     28  HN   GLU P   3       6.680 -16.608  12.181  0.00  0.00      PROA
ATOM     29  CA   GLU P   3       8.044 -15.296  13.111  0.00  0.00      PROA
ATOM     30  HA   GLU P   3       7.823 -14.294  13.448  0.00  0.00      PROA
ATOM     31  CB   GLU P   3       8.092 -16.386  14.219  0.00  0.00      PROA
ATOM     32  HB1  GLU P   3       9.042 -16.401  14.794  0.00  0.00      PROA
ATOM     33  HB2  GLU P   3       8.150 -17.384  13.733  0.00  0.00      PROA
ATOM     34  CG   GLU P   3       6.927 -16.368  15.202  0.00  0.00      PROA
ATOM     35  HG1  GLU P   3       5.975 -16.305  14.633  0.00  0.00      PROA
ATOM     36  HG2  GLU P   3       6.739 -15.457  15.808  0.00  0.00      PROA
ATOM     37  CD   GLU P   3       6.803 -17.605  16.014  0.00  0.00      PROA
ATOM     38  OE1  GLU P   3       6.005 -18.451  15.588  0.00  0.00      PROA
ATOM     39  OE2  GLU P   3       7.400 -17.757  17.134  0.00  0.00      PROA
ATOM     40  C    GLU P   3       9.423 -15.236  12.405  0.00  0.00      PROA
ATOM     41  O    GLU P   3       9.626 -15.788  11.325  0.00  0.00      PROA
ATOM     42  N    ASP P   4      10.451 -14.508  12.966  0.00  0.00      PROA
ATOM     43  HN   ASP P   4      10.377 -13.998  13.819  0.00  0.00      PROA
ATOM     44  CA   ASP P   4      11.707 -14.246  12.447  0.00  0.00      PROA
ATOM     45  HA   ASP P   4      11.533 -13.953  11.422  0.00  0.00      PROA
ATOM     46  CB   ASP P   4      12.438 -13.071  13.302  0.00  0.00      PROA
ATOM     47  HB1  ASP P   4      13.541 -13.176  13.210  0.00  0.00      PROA
ATOM     48  HB2  ASP P   4      12.155 -13.185  14.370  0.00  0.00      PROA
ATOM     49  CG   ASP P   4      12.134 -11.644  12.953  0.00  0.00      PROA
ATOM     50  OD1  ASP P   4      12.555 -10.767  13.791  0.00  0.00      PROA
ATOM     51  OD2  ASP P   4      11.806 -11.403  11.761  0.00  0.00      PROA
ATOM     52  C    ASP P   4      12.677 -15.508  12.293  0.00  0.00      PROA
ATOM     53  O    ASP P   4      13.434 -15.471  11.358  0.00  0.00      PROA
ATOM     54  N    PRO P   5      12.626 -16.504  13.193  0.00  0.00      PROA
ATOM     55  CD   PRO P   5      13.234 -17.854  12.837  0.00  0.00      PROA
ATOM     56  HD1  PRO P   5      14.345 -17.851  12.859  0.00  0.00      PROA
ATOM     57  HD2  PRO P   5      12.744 -18.204  11.904  0.00  0.00      PROA
ATOM     58  CA   PRO P   5      12.299 -16.491  14.635  0.00  0.00      PROA
ATOM     59  HA   PRO P   5      11.229 -16.443  14.773  0.00  0.00      PROA
ATOM     60  CB   PRO P   5      12.640 -17.909  15.102  0.00  0.00      PROA
ATOM     61  HB1  PRO P   5      11.884 -18.349  15.787  0.00  0.00      PROA
ATOM     62  HB2  PRO P   5      13.618 -17.965  15.627  0.00  0.00      PROA
ATOM     63  CG   PRO P   5      12.761 -18.717  13.908  0.00  0.00      PROA
ATOM     64  HG1  PRO P   5      13.581 -19.453  14.045  0.00  0.00      PROA
ATOM     65  HG2  PRO P   5      11.868 -19.303  13.603  0.00  0.00      PROA
ATOM     66  C    PRO P   5      13.003 -15.422  15.556  0.00  0.00      PROA
ATOM     67  O    PRO P   5      14.188 -15.081  15.390  0.00  0.00      PROA
ATOM     68  N    ASN P   6      12.268 -14.865  16.526  0.00  0.00      PROA
ATOM     69  HN   ASN P   6      11.344 -15.160  16.758  0.00  0.00      PROA
ATOM     70  CA   ASN P   6      12.666 -13.756  17.400  0.00  0.00      PROA
ATOM     71  HA   ASN P   6      12.734 -12.854  16.810  0.00  0.00      PROA
ATOM     72  CB   ASN P   6      11.754 -13.509  18.571  0.00  0.00      PROA
ATOM     73  HB1  ASN P   6      12.102 -12.629  19.152  0.00  0.00      PROA
```

| | | | | | | | | | | | |
|---|---|---|---|---|---|---|---|---|---|---|---|
| ATOM | 74 | HB2 | ASN | P | 6 | 11.729 | -14.314 | 19.336 | 0.00 | 0.00 | PROA |
| ATOM | 75 | CG | ASN | P | 6 | 10.395 | -13.159 | 18.048 | 0.00 | 0.00 | PROA |
| ATOM | 76 | OD1 | ASN | P | 6 | 10.126 | -12.007 | 17.585 | 0.00 | 0.00 | PROA |
| ATOM | 77 | ND2 | ASN | P | 6 | 9.387 | -14.074 | 18.070 | 0.00 | 0.00 | PROA |
| ATOM | 78 | HD21 | ASN | P | 6 | 9.608 | -15.047 | 18.007 | 0.00 | 0.00 | PROA |
| ATOM | 79 | HD22 | ASN | P | 6 | 8.540 | -13.737 | 17.661 | 0.00 | 0.00 | PROA |
| ATOM | 80 | C | ASN | P | 6 | 14.033 | -13.815 | 17.951 | 0.00 | 0.00 | PROA |
| ATOM | 81 | O | ASN | P | 6 | 14.329 | -14.823 | 18.657 | 0.00 | 0.00 | PROA |
| ATOM | 82 | N | SER | P | 7 | 14.886 | -12.739 | 17.708 | 0.00 | 0.00 | PROA |
| ATOM | 83 | HN | SER | P | 7 | 14.432 | -11.922 | 17.360 | 0.00 | 0.00 | PROA |
| ATOM | 84 | CA | SER | P | 7 | 16.277 | -12.594 | 17.942 | 0.00 | 0.00 | PROA |
| ATOM | 85 | HA | SER | P | 7 | 16.534 | -11.596 | 17.621 | 0.00 | 0.00 | PROA |
| ATOM | 86 | CB | SER | P | 7 | 16.491 | -12.563 | 19.483 | 0.00 | 0.00 | PROA |
| ATOM | 87 | HB1 | SER | P | 7 | 16.670 | -13.569 | 19.920 | 0.00 | 0.00 | PROA |
| ATOM | 88 | HB2 | SER | P | 7 | 15.595 | -12.081 | 19.929 | 0.00 | 0.00 | PROA |
| ATOM | 89 | OG | SER | P | 7 | 17.743 | -11.947 | 19.717 | 0.00 | 0.00 | PROA |
| ATOM | 90 | HG1 | SER | P | 7 | 17.910 | -12.000 | 20.661 | 0.00 | 0.00 | PROA |
| ATOM | 91 | C | SER | P | 7 | 17.200 | -13.470 | 17.175 | 0.00 | 0.00 | PROA |
| ATOM | 92 | O | SER | P | 7 | 18.343 | -13.012 | 16.923 | 0.00 | 0.00 | PROA |
| ATOM | 93 | N | LEU | P | 8 | 16.874 | -14.707 | 16.860 | 0.00 | 0.00 | PROA |
| ATOM | 94 | HN | LEU | P | 8 | 16.036 | -15.130 | 17.195 | 0.00 | 0.00 | PROA |
| ATOM | 95 | CA | LEU | P | 8 | 17.642 | -15.544 | 15.920 | 0.00 | 0.00 | PROA |
| ATOM | 96 | HA | LEU | P | 8 | 18.665 | -15.637 | 16.254 | 0.00 | 0.00 | PROA |
| ATOM | 97 | CB | LEU | P | 8 | 16.965 | -16.951 | 16.078 | 0.00 | 0.00 | PROA |
| ATOM | 98 | HB1 | LEU | P | 8 | 15.980 | -17.017 | 15.569 | 0.00 | 0.00 | PROA |
| ATOM | 99 | HB2 | LEU | P | 8 | 16.789 | -17.084 | 17.167 | 0.00 | 0.00 | PROA |
| ATOM | 100 | CG | LEU | P | 8 | 17.748 | -18.150 | 15.567 | 0.00 | 0.00 | PROA |
| ATOM | 101 | HG | LEU | P | 8 | 17.927 | -18.098 | 14.472 | 0.00 | 0.00 | PROA |
| ATOM | 102 | CD1 | LEU | P | 8 | 19.144 | -18.369 | 16.194 | 0.00 | 0.00 | PROA |
| ATOM | 103 | HD11 | LEU | P | 8 | 19.046 | -18.701 | 17.250 | 0.00 | 0.00 | PROA |
| ATOM | 104 | HD12 | LEU | P | 8 | 19.740 | -17.440 | 16.072 | 0.00 | 0.00 | PROA |
| ATOM | 105 | HD13 | LEU | P | 8 | 19.621 | -19.243 | 15.701 | 0.00 | 0.00 | PROA |
| ATOM | 106 | CD2 | LEU | P | 8 | 16.764 | -19.346 | 15.768 | 0.00 | 0.00 | PROA |
| ATOM | 107 | HD21 | LEU | P | 8 | 16.298 | -19.250 | 16.772 | 0.00 | 0.00 | PROA |
| ATOM | 108 | HD22 | LEU | P | 8 | 17.303 | -20.299 | 15.577 | 0.00 | 0.00 | PROA |
| ATOM | 109 | HD23 | LEU | P | 8 | 16.005 | -19.184 | 14.973 | 0.00 | 0.00 | PROA |
| ATOM | 110 | C | LEU | P | 8 | 17.674 | -15.282 | 14.428 | 0.00 | 0.00 | PROA |
| ATOM | 111 | O | LEU | P | 8 | 18.745 | -15.164 | 13.860 | 0.00 | 0.00 | PROA |
| ATOM | 112 | N | ARG | P | 9 | 16.458 | -14.988 | 13.819 | 0.00 | 0.00 | PROA |
| ATOM | 113 | HN | ARG | P | 9 | 15.579 | -14.905 | 14.282 | 0.00 | 0.00 | PROA |
| ATOM | 114 | CA | ARG | P | 9 | 16.391 | -14.390 | 12.486 | 0.00 | 0.00 | PROA |
| ATOM | 115 | HA | ARG | P | 9 | 15.347 | -14.381 | 12.212 | 0.00 | 0.00 | PROA |
| ATOM | 116 | CB | ARG | P | 9 | 16.952 | -12.954 | 12.445 | 0.00 | 0.00 | PROA |
| ATOM | 117 | HB1 | ARG | P | 9 | 16.638 | -12.556 | 11.456 | 0.00 | 0.00 | PROA |
| ATOM | 118 | HB2 | ARG | P | 9 | 18.048 | -12.932 | 12.624 | 0.00 | 0.00 | PROA |
| ATOM | 119 | CG | ARG | P | 9 | 16.214 | -12.004 | 13.438 | 0.00 | 0.00 | PROA |
| ATOM | 120 | HG1 | ARG | P | 9 | 16.298 | -12.506 | 14.426 | 0.00 | 0.00 | PROA |
| ATOM | 121 | HG2 | ARG | P | 9 | 15.176 | -11.762 | 13.127 | 0.00 | 0.00 | PROA |
| ATOM | 122 | CD | ARG | P | 9 | 17.025 | -10.781 | 13.656 | 0.00 | 0.00 | PROA |
| ATOM | 123 | HD1 | ARG | P | 9 | 17.046 | -10.195 | 12.712 | 0.00 | 0.00 | PROA |
| ATOM | 124 | HD2 | ARG | P | 9 | 18.051 | -11.089 | 13.951 | 0.00 | 0.00 | PROA |
| ATOM | 125 | NE | ARG | P | 9 | 16.325 | -9.986 | 14.745 | 0.00 | 0.00 | PROA |
| ATOM | 126 | HE | ARG | P | 9 | 15.475 | -10.288 | 15.178 | 0.00 | 0.00 | PROA |
| ATOM | 127 | CZ | ARG | P | 9 | 16.548 | -8.695 | 14.891 | 0.00 | 0.00 | PROA |
| ATOM | 128 | NH1 | ARG | P | 9 | 17.317 | -7.978 | 14.130 | 0.00 | 0.00 | PROA |
| ATOM | 129 | HH11 | ARG | P | 9 | 17.566 | -7.039 | 14.369 | 0.00 | 0.00 | PROA |
| ATOM | 130 | HH12 | ARG | P | 9 | 17.717 | -8.419 | 13.326 | 0.00 | 0.00 | PROA |
| ATOM | 131 | NH2 | ARG | P | 9 | 16.080 | -8.069 | 15.960 | 0.00 | 0.00 | PROA |
| ATOM | 132 | HH21 | ARG | P | 9 | 15.876 | -8.644 | 16.753 | 0.00 | 0.00 | PROA |
| ATOM | 133 | HH22 | ARG | P | 9 | 16.267 | -7.091 | 16.044 | 0.00 | 0.00 | PROA |
| ATOM | 134 | C | ARG | P | 9 | 16.875 | -15.265 | 11.440 | 0.00 | 0.00 | PROA |
| ATOM | 135 | O | ARG | P | 9 | 18.034 | -15.074 | 10.989 | 0.00 | 0.00 | PROA |
| ATOM | 136 | N | HSD | P | 10 | 16.096 | -16.291 | 10.966 | 0.00 | 0.00 | PROA |
| ATOM | 137 | HN | HSD | P | 10 | 15.128 | -16.290 | 11.204 | 0.00 | 0.00 | PROA |
| ATOM | 138 | CA | HSD | P | 10 | 16.508 | -17.291 | 10.013 | 0.00 | 0.00 | PROA |
| ATOM | 139 | HA | HSD | P | 10 | 17.472 | -17.016 | 9.611 | 0.00 | 0.00 | PROA |
| ATOM | 140 | CB | HSD | P | 10 | 16.649 | -18.687 | 10.570 | 0.00 | 0.00 | PROA |
| ATOM | 141 | HB1 | HSD | P | 10 | 16.813 | -19.533 | 9.869 | 0.00 | 0.00 | PROA |
| ATOM | 142 | HB2 | HSD | P | 10 | 15.717 | -18.831 | 11.158 | 0.00 | 0.00 | PROA |
| ATOM | 143 | ND1 | HSD | P | 10 | 18.165 | -19.815 | 12.400 | 0.00 | 0.00 | PROA |
| ATOM | 144 | HD1 | HSD | P | 10 | 17.657 | -20.676 | 12.425 | 0.00 | 0.00 | PROA |
| ATOM | 145 | CG | HSD | P | 10 | 17.913 | -18.769 | 11.486 | 0.00 | 0.00 | PROA |
| ATOM | 146 | CE1 | HSD | P | 10 | 19.432 | -19.594 | 12.824 | 0.00 | 0.00 | PROA |

```
ATOM    147  HE1 HSD P  10      19.881 -20.327  13.495  0.00  0.00      PROA
ATOM    148  NE2 HSD P  10      19.966 -18.505  12.332  0.00  0.00      PROA
ATOM    149  CD2 HSD P  10      19.003 -17.985  11.530  0.00  0.00      PROA
ATOM    150  HD2 HSD P  10      19.145 -17.122  10.891  0.00  0.00      PROA
ATOM    151  C   HSD P  10      15.513 -17.269   8.812  0.00  0.00      PROA
ATOM    152  O   HSD P  10      15.481 -18.165   8.033  0.00  0.00      PROA
ATOM    153  N   LYS P  11      14.602 -16.260   8.687  0.00  0.00      PROA
ATOM    154  HN  LYS P  11      14.660 -15.609   9.440  0.00  0.00      PROA
ATOM    155  CA  LYS P  11      13.471 -16.250   7.775  0.00  0.00      PROA
ATOM    156  HA  LYS P  11      12.938 -17.182   7.894  0.00  0.00      PROA
ATOM    157  CB  LYS P  11      12.616 -15.001   8.078  0.00  0.00      PROA
ATOM    158  HB1 LYS P  11      13.242 -14.142   7.753  0.00  0.00      PROA
ATOM    159  HB2 LYS P  11      12.448 -14.857   9.167  0.00  0.00      PROA
ATOM    160  CG  LYS P  11      11.272 -14.996   7.335  0.00  0.00      PROA
ATOM    161  HG1 LYS P  11      10.835 -16.016   7.379  0.00  0.00      PROA
ATOM    162  HG2 LYS P  11      11.347 -14.659   6.279  0.00  0.00      PROA
ATOM    163  CD  LYS P  11      10.405 -13.950   8.078  0.00  0.00      PROA
ATOM    164  HD1 LYS P  11      10.391 -14.324   9.124  0.00  0.00      PROA
ATOM    165  HD2 LYS P  11       9.383 -14.196   7.717  0.00  0.00      PROA
ATOM    166  CE  LYS P  11      10.842 -12.587   7.805  0.00  0.00      PROA
ATOM    167  HE1 LYS P  11      10.433 -12.167   6.861  0.00  0.00      PROA
ATOM    168  HE2 LYS P  11      11.906 -12.268   7.828  0.00  0.00      PROA
ATOM    169  NZ  LYS P  11      10.135 -11.864   8.835  0.00  0.00      PROA
ATOM    170  HZ1 LYS P  11      10.335 -10.858   8.663  0.00  0.00      PROA
ATOM    171  HZ2 LYS P  11      10.435 -12.105   9.801  0.00  0.00      PROA
ATOM    172  HZ3 LYS P  11       9.096 -11.870   8.784  0.00  0.00      PROA
ATOM    173  C   LYS P  11      13.766 -16.215   6.269  0.00  0.00      PROA
ATOM    174  O   LYS P  11      13.101 -16.841   5.448  0.00  0.00      PROA
ATOM    175  N   TYR P  12      14.826 -15.548   5.789  0.00  0.00      PROA
ATOM    176  HN  TYR P  12      15.532 -15.165   6.379  0.00  0.00      PROA
ATOM    177  CA  TYR P  12      15.008 -15.265   4.356  0.00  0.00      PROA
ATOM    178  HA  TYR P  12      14.086 -15.453   3.825  0.00  0.00      PROA
ATOM    179  CB  TYR P  12      15.616 -13.817   4.050  0.00  0.00      PROA
ATOM    180  HB1 TYR P  12      15.702 -13.769   2.944  0.00  0.00      PROA
ATOM    181  HB2 TYR P  12      16.515 -13.684   4.689  0.00  0.00      PROA
ATOM    182  CG  TYR P  12      14.662 -12.741   4.417  0.00  0.00      PROA
ATOM    183  CD1 TYR P  12      14.691 -12.293   5.747  0.00  0.00      PROA
ATOM    184  HD1 TYR P  12      15.474 -12.626   6.412  0.00  0.00      PROA
ATOM    185  CE1 TYR P  12      13.820 -11.317   6.187  0.00  0.00      PROA
ATOM    186  HE1 TYR P  12      13.873 -11.000   7.218  0.00  0.00      PROA
ATOM    187  CZ  TYR P  12      12.816 -10.778   5.309  0.00  0.00      PROA
ATOM    188  OH  TYR P  12      11.828  -9.995   5.772  0.00  0.00      PROA
ATOM    189  HH  TYR P  12      11.368  -9.564   5.048  0.00  0.00      PROA
ATOM    190  CD2 TYR P  12      13.813 -12.106   3.529  0.00  0.00      PROA
ATOM    191  HD2 TYR P  12      13.740 -12.492   2.523  0.00  0.00      PROA
ATOM    192  CE2 TYR P  12      12.847 -11.169   3.998  0.00  0.00      PROA
ATOM    193  HE2 TYR P  12      12.023 -10.990   3.323  0.00  0.00      PROA
ATOM    194  C   TYR P  12      15.870 -16.369   3.781  0.00  0.00      PROA
ATOM    195  O   TYR P  12      15.918 -16.557   2.560  0.00  0.00      PROA
ATOM    196  N   ASN P  13      16.387 -17.240   4.688  0.00  0.00      PROA
ATOM    197  HN  ASN P  13      16.381 -17.075   5.671  0.00  0.00      PROA
ATOM    198  CA  ASN P  13      16.938 -18.529   4.296  0.00  0.00      PROA
ATOM    199  HA  ASN P  13      17.460 -18.549   3.350  0.00  0.00      PROA
ATOM    200  CB  ASN P  13      17.977 -18.930   5.265  0.00  0.00      PROA
ATOM    201  HB1 ASN P  13      18.217 -20.014   5.280  0.00  0.00      PROA
ATOM    202  HB2 ASN P  13      17.409 -18.553   6.143  0.00  0.00      PROA
ATOM    203  CG  ASN P  13      19.271 -18.158   5.089  0.00  0.00      PROA
ATOM    204  OD1 ASN P  13      19.550 -17.407   6.031  0.00  0.00      PROA
ATOM    205  ND2 ASN P  13      20.023 -18.385   3.988  0.00  0.00      PROA
ATOM    206 HD21 ASN P  13      19.849 -19.198   3.432  0.00  0.00      PROA
ATOM    207 HD22 ASN P  13      20.962 -18.044   3.944  0.00  0.00      PROA
ATOM    208  C   ASN P  13      15.846 -19.582   4.115  0.00  0.00      PROA
ATOM    209  O   ASN P  13      16.007 -20.405   3.177  0.00  0.00      PROA
ATOM    210  N   PHE P  14      14.700 -19.482   4.788  0.00  0.00      PROA
ATOM    211  HN  PHE P  14      14.766 -19.140   5.722  0.00  0.00      PROA
ATOM    212  CA  PHE P  14      13.368 -20.086   4.490  0.00  0.00      PROA
ATOM    213  HA  PHE P  14      13.481 -21.156   4.592  0.00  0.00      PROA
ATOM    214  CB  PHE P  14      12.234 -19.857   5.568  0.00  0.00      PROA
ATOM    215  HB1 PHE P  14      11.407 -20.571   5.369  0.00  0.00      PROA
ATOM    216  HB2 PHE P  14      11.829 -18.825   5.484  0.00  0.00      PROA
ATOM    217  CG  PHE P  14      12.620 -20.146   7.063  0.00  0.00      PROA
ATOM    218  CD1 PHE P  14      11.803 -19.544   8.059  0.00  0.00      PROA
ATOM    219  HD1 PHE P  14      10.923 -18.990   7.768  0.00  0.00      PROA
```

| | | | | | | | | | | | |
|---|---|---|---|---|---|---|---|---|---|---|---|
| ATOM | 220 | CE1 | PHE | P | 14 | 12.167 | -19.758 | 9.400 | 0.00 | 0.00 | PROA |
| ATOM | 221 | HE1 | PHE | P | 14 | 11.585 | -19.264 | 10.164 | 0.00 | 0.00 | PROA |
| ATOM | 222 | CZ | PHE | P | 14 | 13.328 | -20.568 | 9.737 | 0.00 | 0.00 | PROA |
| ATOM | 223 | HZ | PHE | P | 14 | 13.588 | -20.667 | 10.780 | 0.00 | 0.00 | PROA |
| ATOM | 224 | CD2 | PHE | P | 14 | 13.743 | -21.003 | 7.393 | 0.00 | 0.00 | PROA |
| ATOM | 225 | HD2 | PHE | P | 14 | 14.266 | -21.406 | 6.538 | 0.00 | 0.00 | PROA |
| ATOM | 226 | CE2 | PHE | P | 14 | 14.084 | -21.142 | 8.757 | 0.00 | 0.00 | PROA |
| ATOM | 227 | HE2 | PHE | P | 14 | 15.018 | -21.663 | 8.905 | 0.00 | 0.00 | PROA |
| ATOM | 228 | C | PHE | P | 14 | 12.823 | -19.771 | 3.058 | 0.00 | 0.00 | PROA |
| ATOM | 229 | O | PHE | P | 14 | 12.476 | -20.724 | 2.341 | 0.00 | 0.00 | PROA |
| ATOM | 230 | N | ILE | P | 15 | 12.854 | -18.483 | 2.682 | 0.00 | 0.00 | PROA |
| ATOM | 231 | HN | ILE | P | 15 | 13.081 | -17.779 | 3.350 | 0.00 | 0.00 | PROA |
| ATOM | 232 | CA | ILE | P | 15 | 12.656 | -18.053 | 1.368 | 0.00 | 0.00 | PROA |
| ATOM | 233 | HA | ILE | P | 15 | 11.654 | -18.400 | 1.161 | 0.00 | 0.00 | PROA |
| ATOM | 234 | CB | ILE | P | 15 | 12.591 | -16.542 | 1.182 | 0.00 | 0.00 | PROA |
| ATOM | 235 | HB | ILE | P | 15 | 13.528 | -16.110 | 1.592 | 0.00 | 0.00 | PROA |
| ATOM | 236 | CG2 | ILE | P | 15 | 12.414 | -16.242 | -0.288 | 0.00 | 0.00 | PROA |
| ATOM | 237 | HG21 | ILE | P | 15 | 11.536 | -16.680 | -0.808 | 0.00 | 0.00 | PROA |
| ATOM | 238 | HG22 | ILE | P | 15 | 13.313 | -16.555 | -0.862 | 0.00 | 0.00 | PROA |
| ATOM | 239 | HG23 | ILE | P | 15 | 12.356 | -15.139 | -0.409 | 0.00 | 0.00 | PROA |
| ATOM | 240 | CG1 | ILE | P | 15 | 11.408 | -16.038 | 2.035 | 0.00 | 0.00 | PROA |
| ATOM | 241 | HG11 | ILE | P | 15 | 11.870 | -16.148 | 3.040 | 0.00 | 0.00 | PROA |
| ATOM | 242 | HG12 | ILE | P | 15 | 10.569 | -16.767 | 2.018 | 0.00 | 0.00 | PROA |
| ATOM | 243 | CD | ILE | P | 15 | 10.863 | -14.659 | 1.691 | 0.00 | 0.00 | PROA |
| ATOM | 244 | HD1 | ILE | P | 15 | 10.110 | -14.292 | 2.420 | 0.00 | 0.00 | PROA |
| ATOM | 245 | HD2 | ILE | P | 15 | 10.338 | -14.704 | 0.713 | 0.00 | 0.00 | PROA |
| ATOM | 246 | HD3 | ILE | P | 15 | 11.695 | -13.925 | 1.636 | 0.00 | 0.00 | PROA |
| ATOM | 247 | C | ILE | P | 15 | 13.614 | -18.773 | 0.396 | 0.00 | 0.00 | PROA |
| ATOM | 248 | O | ILE | P | 15 | 13.189 | -19.087 | -0.669 | 0.00 | 0.00 | PROA |
| ATOM | 249 | N | ALA | P | 16 | 14.915 | -18.992 | 0.719 | 0.00 | 0.00 | PROA |
| ATOM | 250 | HN | ALA | P | 16 | 15.294 | -18.504 | 1.502 | 0.00 | 0.00 | PROA |
| ATOM | 251 | CA | ALA | P | 16 | 15.827 | -19.713 | -0.145 | 0.00 | 0.00 | PROA |
| ATOM | 252 | HA | ALA | P | 16 | 15.657 | -19.307 | -1.132 | 0.00 | 0.00 | PROA |
| ATOM | 253 | CB | ALA | P | 16 | 17.307 | -19.519 | 0.226 | 0.00 | 0.00 | PROA |
| ATOM | 254 | HB1 | ALA | P | 16 | 17.512 | -18.436 | 0.089 | 0.00 | 0.00 | PROA |
| ATOM | 255 | HB2 | ALA | P | 16 | 17.995 | -20.031 | -0.481 | 0.00 | 0.00 | PROA |
| ATOM | 256 | HB3 | ALA | P | 16 | 17.494 | -19.925 | 1.243 | 0.00 | 0.00 | PROA |
| ATOM | 257 | C | ALA | P | 16 | 15.512 | -21.186 | -0.213 | 0.00 | 0.00 | PROA |
| ATOM | 258 | O | ALA | P | 16 | 15.573 | -21.752 | -1.364 | 0.00 | 0.00 | PROA |
| ATOM | 259 | N | ASP | P | 17 | 15.061 | -21.934 | 0.848 | 0.00 | 0.00 | PROA |
| ATOM | 260 | HN | ASP | P | 17 | 15.267 | -21.571 | 1.753 | 0.00 | 0.00 | PROA |
| ATOM | 261 | CA | ASP | P | 17 | 14.435 | -23.309 | 0.724 | 0.00 | 0.00 | PROA |
| ATOM | 262 | HA | ASP | P | 17 | 15.131 | -23.955 | 0.209 | 0.00 | 0.00 | PROA |
| ATOM | 263 | CB | ASP | P | 17 | 14.144 | -24.003 | 2.015 | 0.00 | 0.00 | PROA |
| ATOM | 264 | HB1 | ASP | P | 17 | 13.631 | -24.965 | 1.801 | 0.00 | 0.00 | PROA |
| ATOM | 265 | HB2 | ASP | P | 17 | 13.560 | -23.332 | 2.681 | 0.00 | 0.00 | PROA |
| ATOM | 266 | CG | ASP | P | 17 | 15.420 | -24.296 | 2.724 | 0.00 | 0.00 | PROA |
| ATOM | 267 | OD1 | ASP | P | 17 | 16.473 | -24.594 | 2.170 | 0.00 | 0.00 | PROA |
| ATOM | 268 | OD2 | ASP | P | 17 | 15.359 | -24.369 | 3.983 | 0.00 | 0.00 | PROA |
| ATOM | 269 | C | ASP | P | 17 | 13.241 | -23.314 | -0.198 | 0.00 | 0.00 | PROA |
| ATOM | 270 | O | ASP | P | 17 | 13.108 | -24.233 | -1.025 | 0.00 | 0.00 | PROA |
| ATOM | 271 | N | VAL | P | 18 | 12.459 | -22.256 | -0.163 | 0.00 | 0.00 | PROA |
| ATOM | 272 | HN | VAL | P | 18 | 12.753 | -21.404 | 0.264 | 0.00 | 0.00 | PROA |
| ATOM | 273 | CA | VAL | P | 18 | 11.349 | -22.064 | -1.134 | 0.00 | 0.00 | PROA |
| ATOM | 274 | HA | VAL | P | 18 | 10.835 | -23.005 | -1.255 | 0.00 | 0.00 | PROA |
| ATOM | 275 | CB | VAL | P | 18 | 10.308 | -20.975 | -0.735 | 0.00 | 0.00 | PROA |
| ATOM | 276 | HB | VAL | P | 18 | 10.727 | -19.987 | -0.448 | 0.00 | 0.00 | PROA |
| ATOM | 277 | CG1 | VAL | P | 18 | 9.218 | -20.729 | -1.803 | 0.00 | 0.00 | PROA |
| ATOM | 278 | HG11 | VAL | P | 18 | 9.620 | -20.151 | -2.662 | 0.00 | 0.00 | PROA |
| ATOM | 279 | HG12 | VAL | P | 18 | 8.440 | -20.110 | -1.307 | 0.00 | 0.00 | PROA |
| ATOM | 280 | HG13 | VAL | P | 18 | 8.839 | -21.703 | -2.181 | 0.00 | 0.00 | PROA |
| ATOM | 281 | CG2 | VAL | P | 18 | 9.613 | -21.553 | 0.494 | 0.00 | 0.00 | PROA |
| ATOM | 282 | HG21 | VAL | P | 18 | 9.008 | -20.714 | 0.898 | 0.00 | 0.00 | PROA |
| ATOM | 283 | HG22 | VAL | P | 18 | 10.412 | -21.722 | 1.248 | 0.00 | 0.00 | PROA |
| ATOM | 284 | HG23 | VAL | P | 18 | 8.970 | -22.455 | 0.401 | 0.00 | 0.00 | PROA |
| ATOM | 285 | C | VAL | P | 18 | 11.663 | -21.751 | -2.567 | 0.00 | 0.00 | PROA |
| ATOM | 286 | O | VAL | P | 18 | 11.042 | -22.323 | -3.451 | 0.00 | 0.00 | PROA |
| ATOM | 287 | N | VAL | P | 19 | 12.725 | -20.886 | -2.781 | 0.00 | 0.00 | PROA |
| ATOM | 288 | HN | VAL | P | 19 | 13.195 | -20.278 | -2.146 | 0.00 | 0.00 | PROA |
| ATOM | 289 | CA | VAL | P | 19 | 13.299 | -20.716 | -4.116 | 0.00 | 0.00 | PROA |
| ATOM | 290 | HA | VAL | P | 19 | 12.501 | -20.395 | -4.769 | 0.00 | 0.00 | PROA |
| ATOM | 291 | CB | VAL | P | 19 | 14.198 | -19.436 | -4.074 | 0.00 | 0.00 | PROA |
| ATOM | 292 | HB | VAL | P | 19 | 14.947 | -19.511 | -3.257 | 0.00 | 0.00 | PROA |

```
ATOM    293  CG1 VAL P  19      14.873 -19.390  -5.495  0.00  0.00      PROA
ATOM    294 HG11 VAL P  19      15.659 -18.605  -5.470  0.00  0.00      PROA
ATOM    295 HG12 VAL P  19      14.151 -19.205  -6.319  0.00  0.00      PROA
ATOM    296 HG13 VAL P  19      15.422 -20.320  -5.754  0.00  0.00      PROA
ATOM    297  CG2 VAL P  19      13.341 -18.152  -3.853  0.00  0.00      PROA
ATOM    298 HG21 VAL P  19      14.023 -17.333  -3.538  0.00  0.00      PROA
ATOM    299 HG22 VAL P  19      12.603 -18.315  -3.039  0.00  0.00      PROA
ATOM    300 HG23 VAL P  19      12.818 -17.849  -4.786  0.00  0.00      PROA
ATOM    301  C   VAL P  19      13.912 -21.969  -4.748  0.00  0.00      PROA
ATOM    302  O   VAL P  19      13.622 -22.197  -5.956  0.00  0.00      PROA
ATOM    303  N   GLU P  20      14.601 -22.858  -4.001  0.00  0.00      PROA
ATOM    304  HN  GLU P  20      14.873 -22.561  -3.090  0.00  0.00      PROA
ATOM    305  CA  GLU P  20      14.998 -24.164  -4.600  0.00  0.00      PROA
ATOM    306  HA  GLU P  20      15.268 -23.975  -5.629  0.00  0.00      PROA
ATOM    307  CB  GLU P  20      16.124 -24.807  -3.740  0.00  0.00      PROA
ATOM    308  HB1 GLU P  20      15.742 -25.025  -2.720  0.00  0.00      PROA
ATOM    309  HB2 GLU P  20      16.904 -24.017  -3.693  0.00  0.00      PROA
ATOM    310  CG  GLU P  20      16.705 -26.006  -4.500  0.00  0.00      PROA
ATOM    311  HG1 GLU P  20      16.684 -25.781  -5.588  0.00  0.00      PROA
ATOM    312  HG2 GLU P  20      16.071 -26.903  -4.331  0.00  0.00      PROA
ATOM    313  CD  GLU P  20      18.075 -26.488  -4.014  0.00  0.00      PROA
ATOM    314  OE1 GLU P  20      19.085 -25.895  -4.395  0.00  0.00      PROA
ATOM    315  OE2 GLU P  20      18.142 -27.411  -3.198  0.00  0.00      PROA
ATOM    316  C   GLU P  20      13.765 -25.113  -4.732  0.00  0.00      PROA
ATOM    317  O   GLU P  20      13.649 -25.866  -5.697  0.00  0.00      PROA
ATOM    318  N   LYS P  21      12.717 -25.060  -3.919  0.00  0.00      PROA
ATOM    319  HN  LYS P  21      12.753 -24.363  -3.207  0.00  0.00      PROA
ATOM    320  CA  LYS P  21      11.621 -25.854  -4.084  0.00  0.00      PROA
ATOM    321  HA  LYS P  21      11.963 -26.867  -4.234  0.00  0.00      PROA
ATOM    322  CB  LYS P  21      10.687 -25.714  -2.896  0.00  0.00      PROA
ATOM    323  HB1 LYS P  21      10.383 -24.650  -2.802  0.00  0.00      PROA
ATOM    324  HB2 LYS P  21      11.171 -25.899  -1.912  0.00  0.00      PROA
ATOM    325  CG  LYS P  21       9.415 -26.629  -2.910  0.00  0.00      PROA
ATOM    326  HG1 LYS P  21       8.933 -26.670  -3.910  0.00  0.00      PROA
ATOM    327  HG2 LYS P  21       8.709 -26.235  -2.149  0.00  0.00      PROA
ATOM    328  CD  LYS P  21       9.656 -28.036  -2.340  0.00  0.00      PROA
ATOM    329  HD1 LYS P  21      10.016 -27.995  -1.289  0.00  0.00      PROA
ATOM    330  HD2 LYS P  21      10.462 -28.393  -3.016  0.00  0.00      PROA
ATOM    331  CE  LYS P  21       8.393 -28.987  -2.389  0.00  0.00      PROA
ATOM    332  HE1 LYS P  21       7.850 -28.775  -3.335  0.00  0.00      PROA
ATOM    333  HE2 LYS P  21       7.817 -28.863  -1.447  0.00  0.00      PROA
ATOM    334  NZ  LYS P  21       8.879 -30.324  -2.549  0.00  0.00      PROA
ATOM    335  HZ1 LYS P  21       9.685 -30.340  -3.205  0.00  0.00      PROA
ATOM    336  HZ2 LYS P  21       8.134 -30.978  -2.864  0.00  0.00      PROA
ATOM    337  HZ3 LYS P  21       9.190 -30.716  -1.637  0.00  0.00      PROA
ATOM    338  C   LYS P  21      10.877 -25.604  -5.408  0.00  0.00      PROA
ATOM    339  O   LYS P  21      10.454 -26.564  -6.050  0.00  0.00      PROA
ATOM    340  N   ILE P  22      10.880 -24.318  -5.962  0.00  0.00      PROA
ATOM    341  HN  ILE P  22      11.300 -23.509  -5.558  0.00  0.00      PROA
ATOM    342  CA  ILE P  22       9.936 -23.979  -7.054  0.00  0.00      PROA
ATOM    343  HA  ILE P  22       9.091 -24.640  -7.175  0.00  0.00      PROA
ATOM    344  CB  ILE P  22       9.175 -22.623  -6.685  0.00  0.00      PROA
ATOM    345  HB  ILE P  22       9.068 -22.628  -5.579  0.00  0.00      PROA
ATOM    346  CG2 ILE P  22      10.062 -21.446  -6.991  0.00  0.00      PROA
ATOM    347 HG21 ILE P  22      10.218 -21.238  -8.071  0.00  0.00      PROA
ATOM    348 HG22 ILE P  22      11.022 -21.537  -6.440  0.00  0.00      PROA
ATOM    349 HG23 ILE P  22       9.573 -20.509  -6.647  0.00  0.00      PROA
ATOM    350  CG1 ILE P  22       7.757 -22.448  -7.313  0.00  0.00      PROA
ATOM    351 HG11 ILE P  22       7.696 -22.713  -8.390  0.00  0.00      PROA
ATOM    352 HG12 ILE P  22       7.481 -21.379  -7.190  0.00  0.00      PROA
ATOM    353  CD  ILE P  22       6.667 -23.269  -6.498  0.00  0.00      PROA
ATOM    354  HD1 ILE P  22       5.797 -23.386  -7.178  0.00  0.00      PROA
ATOM    355  HD2 ILE P  22       6.384 -22.788  -5.537  0.00  0.00      PROA
ATOM    356  HD3 ILE P  22       7.091 -24.282  -6.329  0.00  0.00      PROA
ATOM    357  C   ILE P  22      10.752 -23.874  -8.300  0.00  0.00      PROA
ATOM    358  O   ILE P  22      10.198 -23.750  -9.438  0.00  0.00      PROA
ATOM    359  N   ALA P  23      12.084 -23.920  -8.252  0.00  0.00      PROA
ATOM    360  HN  ALA P  23      12.621 -23.936  -7.412  0.00  0.00      PROA
ATOM    361  CA  ALA P  23      12.905 -23.938  -9.466  0.00  0.00      PROA
ATOM    362  HA  ALA P  23      12.535 -23.227 -10.190  0.00  0.00      PROA
ATOM    363  CB  ALA P  23      14.188 -23.390  -8.925  0.00  0.00      PROA
ATOM    364  HB1 ALA P  23      13.996 -22.452  -8.361  0.00  0.00      PROA
ATOM    365  HB2 ALA P  23      14.940 -23.225  -9.726  0.00  0.00      PROA
```

| | | | | | | | | | | | |
|---|---|---|---|---|---|---|---|---|---|---|---|
| ATOM | 366 | HB3 | ALA | P | 23 | 14.658 | -24.024 | -8.142 | 0.00 | 0.00 | PROA |
| ATOM | 367 | C | ALA | P | 23 | 12.948 | -25.367 | -10.064 | 0.00 | 0.00 | PROA |
| ATOM | 368 | O | ALA | P | 23 | 12.903 | -26.357 | -9.247 | 0.00 | 0.00 | PROA |
| ATOM | 369 | N | PRO | P | 24 | 13.131 | -25.544 | -11.370 | 0.00 | 0.00 | PROA |
| ATOM | 370 | CD | PRO | P | 24 | 13.436 | -26.838 | -12.002 | 0.00 | 0.00 | PROA |
| ATOM | 371 | HD1 | PRO | P | 24 | 12.406 | -27.219 | -12.167 | 0.00 | 0.00 | PROA |
| ATOM | 372 | HD2 | PRO | P | 24 | 14.098 | -27.422 | -11.326 | 0.00 | 0.00 | PROA |
| ATOM | 373 | CA | PRO | P | 24 | 13.059 | -24.553 | -12.408 | 0.00 | 0.00 | PROA |
| ATOM | 374 | HA | PRO | P | 24 | 13.300 | -23.550 | -12.088 | 0.00 | 0.00 | PROA |
| ATOM | 375 | CB | PRO | P | 24 | 14.099 | -25.010 | -13.415 | 0.00 | 0.00 | PROA |
| ATOM | 376 | HB1 | PRO | P | 24 | 15.077 | -24.711 | -12.982 | 0.00 | 0.00 | PROA |
| ATOM | 377 | HB2 | PRO | P | 24 | 13.929 | -24.717 | -14.473 | 0.00 | 0.00 | PROA |
| ATOM | 378 | CG | PRO | P | 24 | 14.163 | -26.589 | -13.294 | 0.00 | 0.00 | PROA |
| ATOM | 379 | HG1 | PRO | P | 24 | 13.591 | -26.967 | -14.169 | 0.00 | 0.00 | PROA |
| ATOM | 380 | HG2 | PRO | P | 24 | 15.219 | -26.935 | -13.282 | 0.00 | 0.00 | PROA |
| ATOM | 381 | C | PRO | P | 24 | 11.694 | -24.545 | -13.058 | 0.00 | 0.00 | PROA |
| ATOM | 382 | O | PRO | P | 24 | 11.542 | -24.322 | -14.268 | 0.00 | 0.00 | PROA |
| ATOM | 383 | N | ALA | P | 25 | 10.588 | -24.736 | -12.340 | 0.00 | 0.00 | PROA |
| ATOM | 384 | HN | ALA | P | 25 | 10.540 | -24.692 | -11.345 | 0.00 | 0.00 | PROA |
| ATOM | 385 | CA | ALA | P | 25 | 9.235 | -24.723 | -12.893 | 0.00 | 0.00 | PROA |
| ATOM | 386 | HA | ALA | P | 25 | 9.180 | -25.126 | -13.894 | 0.00 | 0.00 | PROA |
| ATOM | 387 | CB | ALA | P | 25 | 8.438 | -25.623 | -12.041 | 0.00 | 0.00 | PROA |
| ATOM | 388 | HB1 | ALA | P | 25 | 7.412 | -25.737 | -12.453 | 0.00 | 0.00 | PROA |
| ATOM | 389 | HB2 | ALA | P | 25 | 8.480 | -25.487 | -10.939 | 0.00 | 0.00 | PROA |
| ATOM | 390 | HB3 | ALA | P | 25 | 8.855 | -26.648 | -12.139 | 0.00 | 0.00 | PROA |
| ATOM | 391 | C | ALA | P | 25 | 8.538 | -23.392 | -13.108 | 0.00 | 0.00 | PROA |
| ATOM | 392 | O | ALA | P | 25 | 7.349 | -23.291 | -13.471 | 0.00 | 0.00 | PROA |
| ATOM | 393 | N | VAL | P | 26 | 9.298 | -22.256 | -12.902 | 0.00 | 0.00 | PROA |
| ATOM | 394 | HN | VAL | P | 26 | 10.285 | -22.380 | -12.831 | 0.00 | 0.00 | PROA |
| ATOM | 395 | CA | VAL | P | 26 | 8.749 | -20.889 | -12.953 | 0.00 | 0.00 | PROA |
| ATOM | 396 | HA | VAL | P | 26 | 7.689 | -20.816 | -13.147 | 0.00 | 0.00 | PROA |
| ATOM | 397 | CB | VAL | P | 26 | 8.933 | -20.188 | -11.688 | 0.00 | 0.00 | PROA |
| ATOM | 398 | HB | VAL | P | 26 | 10.036 | -20.252 | -11.567 | 0.00 | 0.00 | PROA |
| ATOM | 399 | CG1 | VAL | P | 26 | 8.407 | -18.734 | -11.715 | 0.00 | 0.00 | PROA |
| ATOM | 400 | HG11 | VAL | P | 26 | 9.187 | -18.207 | -12.304 | 0.00 | 0.00 | PROA |
| ATOM | 401 | HG12 | VAL | P | 26 | 8.361 | -18.424 | -10.649 | 0.00 | 0.00 | PROA |
| ATOM | 402 | HG13 | VAL | P | 26 | 7.421 | -18.622 | -12.213 | 0.00 | 0.00 | PROA |
| ATOM | 403 | CG2 | VAL | P | 26 | 8.223 | -21.009 | -10.607 | 0.00 | 0.00 | PROA |
| ATOM | 404 | HG21 | VAL | P | 26 | 8.635 | -22.034 | -10.495 | 0.00 | 0.00 | PROA |
| ATOM | 405 | HG22 | VAL | P | 26 | 7.133 | -21.139 | -10.779 | 0.00 | 0.00 | PROA |
| ATOM | 406 | HG23 | VAL | P | 26 | 8.170 | -20.473 | -9.636 | 0.00 | 0.00 | PROA |
| ATOM | 407 | C | VAL | P | 26 | 9.412 | -20.106 | -14.125 | 0.00 | 0.00 | PROA |
| ATOM | 408 | O | VAL | P | 26 | 10.617 | -19.703 | -13.985 | 0.00 | 0.00 | PROA |
| ATOM | 409 | N | VAL | P | 27 | 8.697 | -20.012 | -15.264 | 0.00 | 0.00 | PROA |
| ATOM | 410 | HN | VAL | P | 27 | 7.704 | -19.919 | -15.246 | 0.00 | 0.00 | PROA |
| ATOM | 411 | CA | VAL | P | 27 | 9.269 | -19.856 | -16.573 | 0.00 | 0.00 | PROA |
| ATOM | 412 | HA | VAL | P | 27 | 10.343 | -19.895 | -16.469 | 0.00 | 0.00 | PROA |
| ATOM | 413 | CB | VAL | P | 27 | 8.921 | -21.055 | -17.491 | 0.00 | 0.00 | PROA |
| ATOM | 414 | HB | VAL | P | 27 | 9.374 | -20.919 | -18.496 | 0.00 | 0.00 | PROA |
| ATOM | 415 | CG1 | VAL | P | 27 | 9.335 | -22.304 | -16.799 | 0.00 | 0.00 | PROA |
| ATOM | 416 | HG11 | VAL | P | 27 | 9.286 | -23.227 | -17.415 | 0.00 | 0.00 | PROA |
| ATOM | 417 | HG12 | VAL | P | 27 | 9.034 | -22.521 | -15.751 | 0.00 | 0.00 | PROA |
| ATOM | 418 | HG13 | VAL | P | 27 | 10.426 | -22.286 | -16.590 | 0.00 | 0.00 | PROA |
| ATOM | 419 | CG2 | VAL | P | 27 | 7.406 | -21.190 | -17.801 | 0.00 | 0.00 | PROA |
| ATOM | 420 | HG21 | VAL | P | 27 | 6.846 | -21.267 | -16.844 | 0.00 | 0.00 | PROA |
| ATOM | 421 | HG22 | VAL | P | 27 | 7.227 | -22.080 | -18.441 | 0.00 | 0.00 | PROA |
| ATOM | 422 | HG23 | VAL | P | 27 | 7.092 | -20.209 | -18.219 | 0.00 | 0.00 | PROA |
| ATOM | 423 | C | VAL | P | 27 | 8.834 | -18.590 | -17.267 | 0.00 | 0.00 | PROA |
| ATOM | 424 | O | VAL | P | 27 | 7.870 | -17.868 | -16.906 | 0.00 | 0.00 | PROA |
| ATOM | 425 | N | HSD | P | 28 | 9.586 | -18.142 | -18.267 | 0.00 | 0.00 | PROA |
| ATOM | 426 | HN | HSD | P | 28 | 10.288 | -18.666 | -18.742 | 0.00 | 0.00 | PROA |
| ATOM | 427 | CA | HSD | P | 28 | 9.308 | -16.827 | -18.905 | 0.00 | 0.00 | PROA |
| ATOM | 428 | HA | HSD | P | 28 | 8.546 | -16.355 | -18.304 | 0.00 | 0.00 | PROA |
| ATOM | 429 | CB | HSD | P | 28 | 10.567 | -15.926 | -18.995 | 0.00 | 0.00 | PROA |
| ATOM | 430 | HB1 | HSD | P | 28 | 11.279 | -16.448 | -19.669 | 0.00 | 0.00 | PROA |
| ATOM | 431 | HB2 | HSD | P | 28 | 11.068 | -15.924 | -18.003 | 0.00 | 0.00 | PROA |
| ATOM | 432 | ND1 | HSD | P | 28 | 10.593 | -13.908 | -20.499 | 0.00 | 0.00 | PROA |
| ATOM | 433 | HD1 | HSD | P | 28 | 10.721 | -14.447 | -21.332 | 0.00 | 0.00 | PROA |
| ATOM | 434 | CG | HSD | P | 28 | 10.347 | -14.479 | -19.301 | 0.00 | 0.00 | PROA |
| ATOM | 435 | CE1 | HSD | P | 28 | 10.450 | -12.568 | -20.319 | 0.00 | 0.00 | PROA |
| ATOM | 436 | HE1 | HSD | P | 28 | 10.366 | -11.897 | -21.174 | 0.00 | 0.00 | PROA |
| ATOM | 437 | NE2 | HSD | P | 28 | 10.245 | -12.232 | -19.022 | 0.00 | 0.00 | PROA |
| ATOM | 438 | CD2 | HSD | P | 28 | 10.082 | -13.470 | -18.426 | 0.00 | 0.00 | PROA |

| | | | | | | | | | | | |
|---|---|---|---|---|---|---|---|---|---|---|---|
| ATOM | 439 | HD2 | HSD | P | 28 | 10.178 | -13.486 | -17.347 | 0.00 | 0.00 | PROA |
| ATOM | 440 | C | HSD | P | 28 | 8.739 | -17.039 | -20.249 | 0.00 | 0.00 | PROA |
| ATOM | 441 | O | HSD | P | 28 | 8.700 | -18.128 | -20.828 | 0.00 | 0.00 | PROA |
| ATOM | 442 | N | ILE | P | 29 | 8.031 | -16.000 | -20.751 | 0.00 | 0.00 | PROA |
| ATOM | 443 | HN | ILE | P | 29 | 8.122 | -15.128 | -20.277 | 0.00 | 0.00 | PROA |
| ATOM | 444 | CA | ILE | P | 29 | 7.182 | -16.093 | -21.953 | 0.00 | 0.00 | PROA |
| ATOM | 445 | HA | ILE | P | 29 | 7.430 | -17.000 | -22.483 | 0.00 | 0.00 | PROA |
| ATOM | 446 | CB | ILE | P | 29 | 5.668 | -16.118 | -21.590 | 0.00 | 0.00 | PROA |
| ATOM | 447 | HB | ILE | P | 29 | 5.469 | -15.338 | -20.825 | 0.00 | 0.00 | PROA |
| ATOM | 448 | CG2 | ILE | P | 29 | 4.842 | -16.196 | -22.898 | 0.00 | 0.00 | PROA |
| ATOM | 449 | HG21 | ILE | P | 29 | 5.076 | -15.307 | -23.521 | 0.00 | 0.00 | PROA |
| ATOM | 450 | HG22 | ILE | P | 29 | 3.778 | -16.153 | -22.581 | 0.00 | 0.00 | PROA |
| ATOM | 451 | HG23 | ILE | P | 29 | 5.101 | -17.160 | -23.386 | 0.00 | 0.00 | PROA |
| ATOM | 452 | CG1 | ILE | P | 29 | 5.394 | -17.421 | -20.821 | 0.00 | 0.00 | PROA |
| ATOM | 453 | HG11 | ILE | P | 29 | 6.046 | -17.399 | -19.922 | 0.00 | 0.00 | PROA |
| ATOM | 454 | HG12 | ILE | P | 29 | 5.819 | -18.230 | -21.452 | 0.00 | 0.00 | PROA |
| ATOM | 455 | CD | ILE | P | 29 | 3.877 | -17.666 | -20.505 | 0.00 | 0.00 | PROA |
| ATOM | 456 | HD1 | ILE | P | 29 | 3.508 | -18.189 | -21.413 | 0.00 | 0.00 | PROA |
| ATOM | 457 | HD2 | ILE | P | 29 | 3.231 | -16.781 | -20.323 | 0.00 | 0.00 | PROA |
| ATOM | 458 | HD3 | ILE | P | 29 | 3.778 | -18.344 | -19.630 | 0.00 | 0.00 | PROA |
| ATOM | 459 | C | ILE | P | 29 | 7.564 | -14.892 | -22.866 | 0.00 | 0.00 | PROA |
| ATOM | 460 | O | ILE | P | 29 | 7.686 | -13.757 | -22.408 | 0.00 | 0.00 | PROA |
| ATOM | 461 | N | GLU | P | 30 | 7.528 | -15.104 | -24.177 | 0.00 | 0.00 | PROA |
| ATOM | 462 | HN | GLU | P | 30 | 7.276 | -15.996 | -24.546 | 0.00 | 0.00 | PROA |
| ATOM | 463 | CA | GLU | P | 30 | 7.900 | -13.969 | -25.014 | 0.00 | 0.00 | PROA |
| ATOM | 464 | HA | GLU | P | 30 | 7.701 | -13.054 | -24.475 | 0.00 | 0.00 | PROA |
| ATOM | 465 | CB | GLU | P | 30 | 9.397 | -14.004 | -25.486 | 0.00 | 0.00 | PROA |
| ATOM | 466 | HB1 | GLU | P | 30 | 9.562 | -15.022 | -25.900 | 0.00 | 0.00 | PROA |
| ATOM | 467 | HB2 | GLU | P | 30 | 9.946 | -13.926 | -24.523 | 0.00 | 0.00 | PROA |
| ATOM | 468 | CG | GLU | P | 30 | 9.713 | -12.907 | -26.433 | 0.00 | 0.00 | PROA |
| ATOM | 469 | HG1 | GLU | P | 30 | 9.185 | -11.944 | -26.261 | 0.00 | 0.00 | PROA |
| ATOM | 470 | HG2 | GLU | P | 30 | 9.352 | -13.256 | -27.424 | 0.00 | 0.00 | PROA |
| ATOM | 471 | CD | GLU | P | 30 | 11.175 | -12.534 | -26.511 | 0.00 | 0.00 | PROA |
| ATOM | 472 | OE1 | GLU | P | 30 | 11.403 | -11.422 | -25.964 | 0.00 | 0.00 | PROA |
| ATOM | 473 | OE2 | GLU | P | 30 | 12.010 | -13.187 | -27.127 | 0.00 | 0.00 | PROA |
| ATOM | 474 | C | GLU | P | 30 | 7.011 | -13.995 | -26.246 | 0.00 | 0.00 | PROA |
| ATOM | 475 | O | GLU | P | 30 | 6.874 | -15.000 | -26.978 | 0.00 | 0.00 | PROA |
| ATOM | 476 | N | LEU | P | 31 | 6.400 | -12.809 | -26.480 | 0.00 | 0.00 | PROA |
| ATOM | 477 | HN | LEU | P | 31 | 6.604 | -11.958 | -26.003 | 0.00 | 0.00 | PROA |
| ATOM | 478 | CA | LEU | P | 31 | 5.499 | -12.661 | -27.592 | 0.00 | 0.00 | PROA |
| ATOM | 479 | HA | LEU | P | 31 | 5.133 | -13.600 | -27.978 | 0.00 | 0.00 | PROA |
| ATOM | 480 | CB | LEU | P | 31 | 4.158 | -12.092 | -27.081 | 0.00 | 0.00 | PROA |
| ATOM | 481 | HB1 | LEU | P | 31 | 3.632 | -11.885 | -28.038 | 0.00 | 0.00 | PROA |
| ATOM | 482 | HB2 | LEU | P | 31 | 4.378 | -11.109 | -26.613 | 0.00 | 0.00 | PROA |
| ATOM | 483 | CG | LEU | P | 31 | 3.329 | -13.053 | -26.204 | 0.00 | 0.00 | PROA |
| ATOM | 484 | HG | LEU | P | 31 | 3.555 | -14.020 | -26.704 | 0.00 | 0.00 | PROA |
| ATOM | 485 | CD1 | LEU | P | 31 | 3.876 | -12.999 | -24.725 | 0.00 | 0.00 | PROA |
| ATOM | 486 | HD11 | LEU | P | 31 | 3.808 | -11.975 | -24.301 | 0.00 | 0.00 | PROA |
| ATOM | 487 | HD12 | LEU | P | 31 | 4.925 | -13.359 | -24.657 | 0.00 | 0.00 | PROA |
| ATOM | 488 | HD13 | LEU | P | 31 | 3.321 | -13.754 | -24.127 | 0.00 | 0.00 | PROA |
| ATOM | 489 | CD2 | LEU | P | 31 | 1.825 | -12.784 | -26.327 | 0.00 | 0.00 | PROA |
| ATOM | 490 | HD21 | LEU | P | 31 | 1.220 | -13.570 | -25.826 | 0.00 | 0.00 | PROA |
| ATOM | 491 | HD22 | LEU | P | 31 | 1.451 | -12.653 | -27.364 | 0.00 | 0.00 | PROA |
| ATOM | 492 | HD23 | LEU | P | 31 | 1.561 | -11.833 | -25.818 | 0.00 | 0.00 | PROA |
| ATOM | 493 | C | LEU | P | 31 | 6.177 | -11.795 | -28.671 | 0.00 | 0.00 | PROA |
| ATOM | 494 | O | LEU | P | 31 | 6.705 | -10.690 | -28.442 | 0.00 | 0.00 | PROA |
| ATOM | 495 | N | PHE | P | 32 | 5.989 | -12.209 | -29.902 | 0.00 | 0.00 | PROA |
| ATOM | 496 | HN | PHE | P | 32 | 5.369 | -12.963 | -30.104 | 0.00 | 0.00 | PROA |
| ATOM | 497 | CA | PHE | P | 32 | 7.034 | -11.848 | -30.937 | 0.00 | 0.00 | PROA |
| ATOM | 498 | HA | PHE | P | 32 | 7.369 | -10.825 | -30.850 | 0.00 | 0.00 | PROA |
| ATOM | 499 | CB | PHE | P | 32 | 8.325 | -12.710 | -30.909 | 0.00 | 0.00 | PROA |
| ATOM | 500 | HB1 | PHE | P | 32 | 8.704 | -12.762 | -29.866 | 0.00 | 0.00 | PROA |
| ATOM | 501 | HB2 | PHE | P | 32 | 9.189 | -12.283 | -31.461 | 0.00 | 0.00 | PROA |
| ATOM | 502 | CG | PHE | P | 32 | 8.129 | -14.086 | -31.381 | 0.00 | 0.00 | PROA |
| ATOM | 503 | CD1 | PHE | P | 32 | 8.653 | -14.518 | -32.644 | 0.00 | 0.00 | PROA |
| ATOM | 504 | HD1 | PHE | P | 32 | 8.946 | -13.811 | -33.406 | 0.00 | 0.00 | PROA |
| ATOM | 505 | CE1 | PHE | P | 32 | 8.665 | -15.851 | -33.009 | 0.00 | 0.00 | PROA |
| ATOM | 506 | HE1 | PHE | P | 32 | 8.933 | -16.131 | -34.017 | 0.00 | 0.00 | PROA |
| ATOM | 507 | CZ | PHE | P | 32 | 8.119 | -16.824 | -32.158 | 0.00 | 0.00 | PROA |
| ATOM | 508 | HZ | PHE | P | 32 | 8.094 | -17.869 | -32.429 | 0.00 | 0.00 | PROA |
| ATOM | 509 | CD2 | PHE | P | 32 | 7.617 | -15.113 | -30.527 | 0.00 | 0.00 | PROA |
| ATOM | 510 | HD2 | PHE | P | 32 | 7.257 | -14.781 | -29.565 | 0.00 | 0.00 | PROA |
| ATOM | 511 | CE2 | PHE | P | 32 | 7.612 | -16.445 | -30.915 | 0.00 | 0.00 | PROA |

```
ATOM    512  HE2 PHE P  32       6.988 -17.064 -30.288  0.00  0.00      PROA
ATOM    513  C   PHE P  32       6.316 -11.773 -32.269  0.00  0.00      PROA
ATOM    514  O   PHE P  32       5.319 -12.560 -32.484  0.00  0.00      PROA
ATOM    515  N   ARG P  33       6.677 -10.885 -33.107  0.00  0.00      PROA
ATOM    516  HN  ARG P  33       7.492 -10.368 -32.855  0.00  0.00      PROA
ATOM    517  CA  ARG P  33       6.345 -10.753 -34.497  0.00  0.00      PROA
ATOM    518  HA  ARG P  33       5.590 -11.487 -34.739  0.00  0.00      PROA
ATOM    519  CB  ARG P  33       5.771  -9.321 -34.755  0.00  0.00      PROA
ATOM    520  HB1 ARG P  33       5.182  -9.048 -33.854  0.00  0.00      PROA
ATOM    521  HB2 ARG P  33       5.122  -9.376 -35.655  0.00  0.00      PROA
ATOM    522  CG  ARG P  33       6.759  -8.140 -35.095  0.00  0.00      PROA
ATOM    523  HG1 ARG P  33       7.300  -8.356 -36.041  0.00  0.00      PROA
ATOM    524  HG2 ARG P  33       7.336  -7.971 -34.161  0.00  0.00      PROA
ATOM    525  CD  ARG P  33       6.036  -6.873 -35.263  0.00  0.00      PROA
ATOM    526  HD1 ARG P  33       5.713  -6.483 -34.274  0.00  0.00      PROA
ATOM    527  HD2 ARG P  33       5.233  -6.904 -36.031  0.00  0.00      PROA
ATOM    528  NE  ARG P  33       7.112  -5.904 -35.767  0.00  0.00      PROA
ATOM    529  HE  ARG P  33       7.559  -5.354 -35.061  0.00  0.00      PROA
ATOM    530  CZ  ARG P  33       7.146  -5.320 -36.949  0.00  0.00      PROA
ATOM    531  NH1 ARG P  33       6.652  -5.852 -38.068  0.00  0.00      PROA
ATOM    532 HH11 ARG P  33       6.221  -6.753 -38.022  0.00  0.00      PROA
ATOM    533 HH12 ARG P  33       6.616  -5.288 -38.893  0.00  0.00      PROA
ATOM    534  NH2 ARG P  33       7.868  -4.203 -37.089  0.00  0.00      PROA
ATOM    535 HH21 ARG P  33       8.600  -3.996 -36.441  0.00  0.00      PROA
ATOM    536 HH22 ARG P  33       7.945  -3.787 -37.995  0.00  0.00      PROA
ATOM    537  C   ARG P  33       7.518 -11.094 -35.352  0.00  0.00      PROA
ATOM    538  O   ARG P  33       8.660 -10.983 -34.880  0.00  0.00      PROA
ATOM    539  N   LYS P  34       7.397 -11.580 -36.566  0.00  0.00      PROA
ATOM    540  HN  LYS P  34       6.459 -11.693 -36.886  0.00  0.00      PROA
ATOM    541  CA  LYS P  34       8.456 -11.701 -37.452  0.00  0.00      PROA
ATOM    542  HA  LYS P  34       9.397 -11.790 -36.929  0.00  0.00      PROA
ATOM    543  CB  LYS P  34       8.313 -12.976 -38.338  0.00  0.00      PROA
ATOM    544  HB1 LYS P  34       9.010 -12.940 -39.202  0.00  0.00      PROA
ATOM    545  HB2 LYS P  34       7.290 -13.028 -38.768  0.00  0.00      PROA
ATOM    546  CG  LYS P  34       8.420 -14.303 -37.577  0.00  0.00      PROA
ATOM    547  HG1 LYS P  34       7.770 -15.068 -38.052  0.00  0.00      PROA
ATOM    548  HG2 LYS P  34       7.982 -14.175 -36.564  0.00  0.00      PROA
ATOM    549  CD  LYS P  34       9.851 -14.872 -37.457  0.00  0.00      PROA
ATOM    550  HD1 LYS P  34       9.914 -15.897 -37.032  0.00  0.00      PROA
ATOM    551  HD2 LYS P  34      10.504 -14.159 -36.910  0.00  0.00      PROA
ATOM    552  CE  LYS P  34      10.589 -15.118 -38.898  0.00  0.00      PROA
ATOM    553  HE1 LYS P  34      11.549 -15.604 -38.622  0.00  0.00      PROA
ATOM    554  HE2 LYS P  34      10.758 -14.154 -39.425  0.00  0.00      PROA
ATOM    555  NZ  LYS P  34       9.882 -16.117 -39.717  0.00  0.00      PROA
ATOM    556  HZ1 LYS P  34       9.776 -17.073 -39.321  0.00  0.00      PROA
ATOM    557  HZ2 LYS P  34      10.276 -16.147 -40.679  0.00  0.00      PROA
ATOM    558  HZ3 LYS P  34       8.892 -15.826 -39.850  0.00  0.00      PROA
ATOM    559  C   LYS P  34       8.521 -10.435 -38.216  0.00  0.00      PROA
ATOM    560  O   LYS P  34       7.520  -9.881 -38.729  0.00  0.00      PROA
ATOM    561  N   LEU P  35       9.722  -9.888 -38.369  0.00  0.00      PROA
ATOM    562  HN  LEU P  35      10.524 -10.255 -37.904  0.00  0.00      PROA
ATOM    563  CA  LEU P  35       9.933  -8.758 -39.228  0.00  0.00      PROA
ATOM    564  HA  LEU P  35       9.327  -7.945 -38.858  0.00  0.00      PROA
ATOM    565  CB  LEU P  35      11.430  -8.346 -39.183  0.00  0.00      PROA
ATOM    566  HB1 LEU P  35      12.018  -9.278 -39.322  0.00  0.00      PROA
ATOM    567  HB2 LEU P  35      11.772  -7.873 -38.237  0.00  0.00      PROA
ATOM    568  CG  LEU P  35      11.802  -7.224 -40.139  0.00  0.00      PROA
ATOM    569  HG  LEU P  35      11.425  -7.468 -41.155  0.00  0.00      PROA
ATOM    570  CD1 LEU P  35      11.263  -5.793 -39.721  0.00  0.00      PROA
ATOM    571 HD11 LEU P  35      10.154  -5.832 -39.680  0.00  0.00      PROA
ATOM    572 HD12 LEU P  35      11.517  -4.967 -40.419  0.00  0.00      PROA
ATOM    573 HD13 LEU P  35      11.638  -5.638 -38.687  0.00  0.00      PROA
ATOM    574  CD2 LEU P  35      13.320  -7.002 -40.264  0.00  0.00      PROA
ATOM    575 HD21 LEU P  35      13.871  -7.902 -40.611  0.00  0.00      PROA
ATOM    576 HD22 LEU P  35      13.673  -6.757 -39.239  0.00  0.00      PROA
ATOM    577 HD23 LEU P  35      13.468  -6.177 -40.993  0.00  0.00      PROA
ATOM    578  C   LEU P  35       9.575  -9.078 -40.722  0.00  0.00      PROA
ATOM    579  O   LEU P  35      10.007 -10.184 -41.139  0.00  0.00      PROA
ATOM    580  N   PRO P  36       8.806  -8.425 -41.575  0.00  0.00      PROA
ATOM    581  CD  PRO P  36       7.801  -7.443 -41.062  0.00  0.00      PROA
ATOM    582  HD1 PRO P  36       8.262  -6.438 -40.959  0.00  0.00      PROA
ATOM    583  HD2 PRO P  36       7.329  -7.694 -40.088  0.00  0.00      PROA
ATOM    584  CA  PRO P  36       8.622  -8.728 -42.956  0.00  0.00      PROA
```

| | | | | | | | | | | | |
|---|---|---|---|---|---|---|---|---|---|---|---|
| ATOM | 585 | HA | PRO | P | 36 | 8.165 | -9.668 | -43.227 | 0.00 | 0.00 | PROA |
| ATOM | 586 | CB | PRO | P | 36 | 7.698 | -7.576 | -43.375 | 0.00 | 0.00 | PROA |
| ATOM | 587 | HB1 | PRO | P | 36 | 7.321 | -7.823 | -44.390 | 0.00 | 0.00 | PROA |
| ATOM | 588 | HB2 | PRO | P | 36 | 8.233 | -6.630 | -43.605 | 0.00 | 0.00 | PROA |
| ATOM | 589 | CG | PRO | P | 36 | 6.810 | -7.340 | -42.167 | 0.00 | 0.00 | PROA |
| ATOM | 590 | HG1 | PRO | P | 36 | 6.332 | -6.338 | -42.141 | 0.00 | 0.00 | PROA |
| ATOM | 591 | HG2 | PRO | P | 36 | 6.099 | -8.193 | -42.200 | 0.00 | 0.00 | PROA |
| ATOM | 592 | C | PRO | P | 36 | 9.893 | -8.847 | -43.792 | 0.00 | 0.00 | PROA |
| ATOM | 593 | O | PRO | P | 36 | 10.800 | -8.020 | -43.626 | 0.00 | 0.00 | PROA |
| ATOM | 594 | N | PHE | P | 37 | 10.009 | -9.877 | -44.629 | 0.00 | 0.00 | PROA |
| ATOM | 595 | HN | PHE | P | 37 | 9.236 | -10.481 | -44.810 | 0.00 | 0.00 | PROA |
| ATOM | 596 | CA | PHE | P | 37 | 11.054 | -10.035 | -45.641 | 0.00 | 0.00 | PROA |
| ATOM | 597 | HA | PHE | P | 37 | 10.732 | -10.829 | -46.298 | 0.00 | 0.00 | PROA |
| ATOM | 598 | CB | PHE | P | 37 | 11.427 | -8.699 | -46.400 | 0.00 | 0.00 | PROA |
| ATOM | 599 | HB1 | PHE | P | 37 | 11.978 | -8.082 | -45.659 | 0.00 | 0.00 | PROA |
| ATOM | 600 | HB2 | PHE | P | 37 | 10.549 | -8.068 | -46.656 | 0.00 | 0.00 | PROA |
| ATOM | 601 | CG | PHE | P | 37 | 12.140 | -8.791 | -47.717 | 0.00 | 0.00 | PROA |
| ATOM | 602 | CD1 | PHE | P | 37 | 11.720 | -9.706 | -48.639 | 0.00 | 0.00 | PROA |
| ATOM | 603 | HD1 | PHE | P | 37 | 11.102 | -10.584 | -48.529 | 0.00 | 0.00 | PROA |
| ATOM | 604 | CE1 | PHE | P | 37 | 12.318 | -9.645 | -49.965 | 0.00 | 0.00 | PROA |
| ATOM | 605 | HE1 | PHE | P | 37 | 12.033 | -10.444 | -50.634 | 0.00 | 0.00 | PROA |
| ATOM | 606 | CZ | PHE | P | 37 | 13.248 | -8.659 | -50.335 | 0.00 | 0.00 | PROA |
| ATOM | 607 | HZ | PHE | P | 37 | 13.638 | -8.704 | -51.341 | 0.00 | 0.00 | PROA |
| ATOM | 608 | CD2 | PHE | P | 37 | 13.095 | -7.823 | -48.044 | 0.00 | 0.00 | PROA |
| ATOM | 609 | HD2 | PHE | P | 37 | 13.302 | -7.132 | -47.240 | 0.00 | 0.00 | PROA |
| ATOM | 610 | CE2 | PHE | P | 37 | 13.566 | -7.727 | -49.366 | 0.00 | 0.00 | PROA |
| ATOM | 611 | HE2 | PHE | P | 37 | 14.229 | -6.888 | -49.517 | 0.00 | 0.00 | PROA |
| ATOM | 612 | C | PHE | P | 37 | 12.267 | -10.581 | -45.116 | 0.00 | 0.00 | PROA |
| ATOM | 613 | O | PHE | P | 37 | 13.278 | -10.772 | -45.795 | 0.00 | 0.00 | PROA |
| ATOM | 614 | N | SER | P | 38 | 12.338 | -10.826 | -43.783 | 0.00 | 0.00 | PROA |
| ATOM | 615 | HN | SER | P | 38 | 11.489 | -10.680 | -43.280 | 0.00 | 0.00 | PROA |
| ATOM | 616 | CA | SER | P | 38 | 13.505 | -11.320 | -43.083 | 0.00 | 0.00 | PROA |
| ATOM | 617 | HA | SER | P | 38 | 14.360 | -11.471 | -43.725 | 0.00 | 0.00 | PROA |
| ATOM | 618 | CB | SER | P | 38 | 13.888 | -10.304 | -42.013 | 0.00 | 0.00 | PROA |
| ATOM | 619 | HB1 | SER | P | 38 | 13.126 | -10.111 | -41.229 | 0.00 | 0.00 | PROA |
| ATOM | 620 | HB2 | SER | P | 38 | 14.014 | -9.296 | -42.462 | 0.00 | 0.00 | PROA |
| ATOM | 621 | OG | SER | P | 38 | 15.190 | -10.559 | -41.372 | 0.00 | 0.00 | PROA |
| ATOM | 622 | HG1 | SER | P | 38 | 15.770 | -10.122 | -42.000 | 0.00 | 0.00 | PROA |
| ATOM | 623 | C | SER | P | 38 | 13.191 | -12.625 | -42.358 | 0.00 | 0.00 | PROA |
| ATOM | 624 | O | SER | P | 38 | 12.017 | -12.990 | -42.396 | 0.00 | 0.00 | PROA |
| ATOM | 625 | N | LYS | P | 39 | 14.217 | -13.276 | -41.772 | 0.00 | 0.00 | PROA |
| ATOM | 626 | HN | LYS | P | 39 | 15.177 | -13.020 | -41.853 | 0.00 | 0.00 | PROA |
| ATOM | 627 | CA | LYS | P | 39 | 13.932 | -14.286 | -40.798 | 0.00 | 0.00 | PROA |
| ATOM | 628 | HA | LYS | P | 39 | 12.977 | -14.751 | -40.995 | 0.00 | 0.00 | PROA |
| ATOM | 629 | CB | LYS | P | 39 | 15.012 | -15.410 | -40.975 | 0.00 | 0.00 | PROA |
| ATOM | 630 | HB1 | LYS | P | 39 | 14.704 | -16.292 | -40.374 | 0.00 | 0.00 | PROA |
| ATOM | 631 | HB2 | LYS | P | 39 | 15.949 | -15.045 | -40.503 | 0.00 | 0.00 | PROA |
| ATOM | 632 | CG | LYS | P | 39 | 15.260 | -15.912 | -42.421 | 0.00 | 0.00 | PROA |
| ATOM | 633 | HG1 | LYS | P | 39 | 16.127 | -16.606 | -42.421 | 0.00 | 0.00 | PROA |
| ATOM | 634 | HG2 | LYS | P | 39 | 15.518 | -15.063 | -43.090 | 0.00 | 0.00 | PROA |
| ATOM | 635 | CD | LYS | P | 39 | 13.954 | -16.573 | -42.981 | 0.00 | 0.00 | PROA |
| ATOM | 636 | HD1 | LYS | P | 39 | 13.215 | -15.745 | -42.927 | 0.00 | 0.00 | PROA |
| ATOM | 637 | HD2 | LYS | P | 39 | 13.516 | -17.338 | -42.305 | 0.00 | 0.00 | PROA |
| ATOM | 638 | CE | LYS | P | 39 | 14.084 | -17.227 | -44.320 | 0.00 | 0.00 | PROA |
| ATOM | 639 | HE1 | LYS | P | 39 | 14.264 | -16.451 | -45.095 | 0.00 | 0.00 | PROA |
| ATOM | 640 | HE2 | LYS | P | 39 | 13.186 | -17.741 | -44.723 | 0.00 | 0.00 | PROA |
| ATOM | 641 | NZ | LYS | P | 39 | 15.143 | -18.235 | -44.362 | 0.00 | 0.00 | PROA |
| ATOM | 642 | HZ1 | LYS | P | 39 | 15.305 | -18.703 | -45.276 | 0.00 | 0.00 | PROA |
| ATOM | 643 | HZ2 | LYS | P | 39 | 14.976 | -18.953 | -43.629 | 0.00 | 0.00 | PROA |
| ATOM | 644 | HZ3 | LYS | P | 39 | 16.068 | -17.799 | -44.170 | 0.00 | 0.00 | PROA |
| ATOM | 645 | C | LYS | P | 39 | 14.015 | -13.680 | -39.381 | 0.00 | 0.00 | PROA |
| ATOM | 646 | O | LYS | P | 39 | 13.892 | -14.446 | -38.478 | 0.00 | 0.00 | PROA |
| ATOM | 647 | N | ARG | P | 40 | 14.365 | -12.424 | -39.110 | 0.00 | 0.00 | PROA |
| ATOM | 648 | HN | ARG | P | 40 | 14.801 | -11.861 | -39.809 | 0.00 | 0.00 | PROA |
| ATOM | 649 | CA | ARG | P | 40 | 14.369 | -11.942 | -37.746 | 0.00 | 0.00 | PROA |
| ATOM | 650 | HA | ARG | P | 40 | 14.968 | -12.561 | -37.095 | 0.00 | 0.00 | PROA |
| ATOM | 651 | CB | ARG | P | 40 | 14.877 | -10.488 | -37.596 | 0.00 | 0.00 | PROA |
| ATOM | 652 | HB1 | ARG | P | 40 | 14.900 | -10.060 | -36.571 | 0.00 | 0.00 | PROA |
| ATOM | 653 | HB2 | ARG | P | 40 | 14.197 | -9.828 | -38.176 | 0.00 | 0.00 | PROA |
| ATOM | 654 | CG | ARG | P | 40 | 16.267 | -10.412 | -38.124 | 0.00 | 0.00 | PROA |
| ATOM | 655 | HG1 | ARG | P | 40 | 16.530 | -9.352 | -38.328 | 0.00 | 0.00 | PROA |
| ATOM | 656 | HG2 | ARG | P | 40 | 16.390 | -10.943 | -39.091 | 0.00 | 0.00 | PROA |
| ATOM | 657 | CD | ARG | P | 40 | 17.366 | -10.788 | -37.065 | 0.00 | 0.00 | PROA |

```
ATOM    658  HD1 ARG P  40      17.492 -11.874 -36.867  0.00  0.00      PROA
ATOM    659  HD2 ARG P  40      17.158 -10.336 -36.071  0.00  0.00      PROA
ATOM    660  NE  ARG P  40      18.756 -10.345 -37.535  0.00  0.00      PROA
ATOM    661  HE  ARG P  40      18.914 -10.071 -38.484  0.00  0.00      PROA
ATOM    662  CZ  ARG P  40      19.831 -10.198 -36.726  0.00  0.00      PROA
ATOM    663  NH1 ARG P  40      19.895 -10.742 -35.550  0.00  0.00      PROA
ATOM    664 HH11 ARG P  40      19.022 -11.094 -35.212  0.00  0.00      PROA
ATOM    665 HH12 ARG P  40      20.607 -10.419 -34.927  0.00  0.00      PROA
ATOM    666  NH2 ARG P  40      20.935  -9.775 -37.263  0.00  0.00      PROA
ATOM    667 HH21 ARG P  40      20.858  -9.518 -38.226  0.00  0.00      PROA
ATOM    668 HH22 ARG P  40      21.865 -10.081 -37.057  0.00  0.00      PROA
ATOM    669  C   ARG P  40      13.065 -11.953 -36.990  0.00  0.00      PROA
ATOM    670  O   ARG P  40      11.934 -11.736 -37.444  0.00  0.00      PROA
ATOM    671  N   GLU P  41      13.157 -12.277 -35.649  0.00  0.00      PROA
ATOM    672  HN  GLU P  41      14.013 -12.602 -35.256  0.00  0.00      PROA
ATOM    673  CA  GLU P  41      12.014 -12.344 -34.710  0.00  0.00      PROA
ATOM    674  HA  GLU P  41      11.119 -12.333 -35.315  0.00  0.00      PROA
ATOM    675  CB  GLU P  41      12.151 -13.572 -33.782  0.00  0.00      PROA
ATOM    676  HB1 GLU P  41      11.567 -13.574 -32.837  0.00  0.00      PROA
ATOM    677  HB2 GLU P  41      13.210 -13.528 -33.449  0.00  0.00      PROA
ATOM    678  CG  GLU P  41      11.847 -14.919 -34.539  0.00  0.00      PROA
ATOM    679  HG1 GLU P  41      12.423 -15.024 -35.483  0.00  0.00      PROA
ATOM    680  HG2 GLU P  41      10.760 -15.065 -34.713  0.00  0.00      PROA
ATOM    681  CD  GLU P  41      12.308 -16.097 -33.667  0.00  0.00      PROA
ATOM    682  OE1 GLU P  41      12.006 -17.245 -33.975  0.00  0.00      PROA
ATOM    683  OE2 GLU P  41      13.011 -15.838 -32.640  0.00  0.00      PROA
ATOM    684  C   GLU P  41      12.047 -11.172 -33.835  0.00  0.00      PROA
ATOM    685  O   GLU P  41      13.063 -10.886 -33.157  0.00  0.00      PROA
ATOM    686  N   VAL P  42      11.072 -10.316 -33.840  0.00  0.00      PROA
ATOM    687  HN  VAL P  42      10.180 -10.506 -34.243  0.00  0.00      PROA
ATOM    688  CA  VAL P  42      11.079  -9.052 -33.128  0.00  0.00      PROA
ATOM    689  HA  VAL P  42      12.099  -8.888 -32.812  0.00  0.00      PROA
ATOM    690  CB  VAL P  42      10.900  -7.945 -34.111  0.00  0.00      PROA
ATOM    691  HB  VAL P  42      10.088  -8.195 -34.827  0.00  0.00      PROA
ATOM    692  CG1 VAL P  42      10.597  -6.636 -33.394  0.00  0.00      PROA
ATOM    693 HG11 VAL P  42      11.372  -6.243 -32.702  0.00  0.00      PROA
ATOM    694 HG12 VAL P  42       9.572  -6.667 -32.966  0.00  0.00      PROA
ATOM    695 HG13 VAL P  42      10.445  -5.881 -34.194  0.00  0.00      PROA
ATOM    696  CG2 VAL P  42      12.246  -7.887 -35.014  0.00  0.00      PROA
ATOM    697 HG21 VAL P  42      12.636  -8.857 -35.390  0.00  0.00      PROA
ATOM    698 HG22 VAL P  42      13.060  -7.587 -34.320  0.00  0.00      PROA
ATOM    699 HG23 VAL P  42      11.955  -7.212 -35.847  0.00  0.00      PROA
ATOM    700  C   VAL P  42      10.027  -9.079 -32.017  0.00  0.00      PROA
ATOM    701  O   VAL P  42       8.842  -9.289 -32.346  0.00  0.00      PROA
ATOM    702  N   PRO P  43      10.377  -8.897 -30.661  0.00  0.00      PROA
ATOM    703  CD  PRO P  43      11.769  -9.108 -30.217  0.00  0.00      PROA
ATOM    704  HD1 PRO P  43      12.428  -8.246 -30.457  0.00  0.00      PROA
ATOM    705  HD2 PRO P  43      12.291  -9.981 -30.664  0.00  0.00      PROA
ATOM    706  CA  PRO P  43       9.460  -8.765 -29.548  0.00  0.00      PROA
ATOM    707  HA  PRO P  43       8.846  -9.637 -29.382  0.00  0.00      PROA
ATOM    708  CB  PRO P  43      10.362  -8.451 -28.316  0.00  0.00      PROA
ATOM    709  HB1 PRO P  43       9.942  -8.937 -27.410  0.00  0.00      PROA
ATOM    710  HB2 PRO P  43      10.588  -7.390 -28.074  0.00  0.00      PROA
ATOM    711  CG  PRO P  43      11.612  -9.192 -28.655  0.00  0.00      PROA
ATOM    712  HG1 PRO P  43      12.395  -8.688 -28.050  0.00  0.00      PROA
ATOM    713  HG2 PRO P  43      11.495 -10.264 -28.388  0.00  0.00      PROA
ATOM    714  C   PRO P  43       8.416  -7.651 -29.587  0.00  0.00      PROA
ATOM    715  O   PRO P  43       8.682  -6.520 -30.032  0.00  0.00      PROA
ATOM    716  N   VAL P  44       7.213  -7.981 -29.149  0.00  0.00      PROA
ATOM    717  HN  VAL P  44       7.009  -8.898 -28.817  0.00  0.00      PROA
ATOM    718  CA  VAL P  44       6.098  -7.048 -29.012  0.00  0.00      PROA
ATOM    719  HA  VAL P  44       6.534  -6.063 -28.927  0.00  0.00      PROA
ATOM    720  CB  VAL P  44       5.124  -7.122 -30.182  0.00  0.00      PROA
ATOM    721  HB  VAL P  44       4.108  -6.776 -29.896  0.00  0.00      PROA
ATOM    722  CG1 VAL P  44       5.641  -6.221 -31.386  0.00  0.00      PROA
ATOM    723 HG11 VAL P  44       5.960  -5.211 -31.051  0.00  0.00      PROA
ATOM    724 HG12 VAL P  44       4.796  -6.059 -32.089  0.00  0.00      PROA
ATOM    725 HG13 VAL P  44       6.493  -6.577 -32.002  0.00  0.00      PROA
ATOM    726  CG2 VAL P  44       4.718  -8.556 -30.595  0.00  0.00      PROA
ATOM    727 HG21 VAL P  44       4.211  -9.099 -29.768  0.00  0.00      PROA
ATOM    728 HG22 VAL P  44       5.644  -9.101 -30.880  0.00  0.00      PROA
ATOM    729 HG23 VAL P  44       4.113  -8.647 -31.522  0.00  0.00      PROA
ATOM    730  C   VAL P  44       5.429  -7.244 -27.621  0.00  0.00      PROA
```

| | | | | | | | | | | | |
|---|---|---|---|---|---|---|---|---|---|---|---|
| ATOM | 731 | O | VAL | P | 44 | 4.822 | -6.359 | -27.108 | 0.00 | 0.00 | PROA |
| ATOM | 732 | N | ALA | P | 45 | 5.735 | -8.338 | -26.919 | 0.00 | 0.00 | PROA |
| ATOM | 733 | HN | ALA | P | 45 | 6.274 | -9.087 | -27.295 | 0.00 | 0.00 | PROA |
| ATOM | 734 | CA | ALA | P | 45 | 5.377 | -8.420 | -25.513 | 0.00 | 0.00 | PROA |
| ATOM | 735 | HA | ALA | P | 45 | 5.589 | -7.585 | -24.861 | 0.00 | 0.00 | PROA |
| ATOM | 736 | CB | ALA | P | 45 | 3.898 | -8.797 | -25.351 | 0.00 | 0.00 | PROA |
| ATOM | 737 | HB1 | ALA | P | 45 | 3.658 | -8.474 | -24.316 | 0.00 | 0.00 | PROA |
| ATOM | 738 | HB2 | ALA | P | 45 | 3.612 | -9.865 | -25.235 | 0.00 | 0.00 | PROA |
| ATOM | 739 | HB3 | ALA | P | 45 | 3.298 | -8.232 | -26.095 | 0.00 | 0.00 | PROA |
| ATOM | 740 | C | ALA | P | 45 | 6.188 | -9.546 | -24.855 | 0.00 | 0.00 | PROA |
| ATOM | 741 | O | ALA | P | 45 | 6.933 | -10.338 | -25.439 | 0.00 | 0.00 | PROA |
| ATOM | 742 | N | SER | P | 46 | 6.082 | -9.601 | -23.562 | 0.00 | 0.00 | PROA |
| ATOM | 743 | HN | SER | P | 46 | 5.691 | -8.795 | -23.123 | 0.00 | 0.00 | PROA |
| ATOM | 744 | CA | SER | P | 46 | 6.604 | -10.733 | -22.680 | 0.00 | 0.00 | PROA |
| ATOM | 745 | HA | SER | P | 46 | 6.318 | -11.706 | -23.052 | 0.00 | 0.00 | PROA |
| ATOM | 746 | CB | SER | P | 46 | 8.113 | -10.734 | -22.522 | 0.00 | 0.00 | PROA |
| ATOM | 747 | HB1 | SER | P | 46 | 8.644 | -10.937 | -23.477 | 0.00 | 0.00 | PROA |
| ATOM | 748 | HB2 | SER | P | 46 | 8.359 | -11.530 | -21.787 | 0.00 | 0.00 | PROA |
| ATOM | 749 | OG | SER | P | 46 | 8.662 | -9.505 | -21.964 | 0.00 | 0.00 | PROA |
| ATOM | 750 | HG1 | SER | P | 46 | 8.205 | -8.844 | -22.489 | 0.00 | 0.00 | PROA |
| ATOM | 751 | C | SER | P | 46 | 5.892 | -10.636 | -21.327 | 0.00 | 0.00 | PROA |
| ATOM | 752 | O | SER | P | 46 | 5.410 | -9.557 | -20.990 | 0.00 | 0.00 | PROA |
| ATOM | 753 | N | GLY | P | 47 | 5.919 | -11.801 | -20.591 | 0.00 | 0.00 | PROA |
| ATOM | 754 | HN | GLY | P | 47 | 6.252 | -12.658 | -20.977 | 0.00 | 0.00 | PROA |
| ATOM | 755 | CA | GLY | P | 47 | 5.460 | -11.949 | -19.274 | 0.00 | 0.00 | PROA |
| ATOM | 756 | HA1 | GLY | P | 47 | 4.382 | -11.918 | -19.205 | 0.00 | 0.00 | PROA |
| ATOM | 757 | HA2 | GLY | P | 47 | 5.874 | -11.118 | -18.722 | 0.00 | 0.00 | PROA |
| ATOM | 758 | C | GLY | P | 47 | 6.015 | -13.308 | -18.776 | 0.00 | 0.00 | PROA |
| ATOM | 759 | O | GLY | P | 47 | 7.000 | -13.745 | -19.273 | 0.00 | 0.00 | PROA |
| ATOM | 760 | N | SER | P | 48 | 5.434 | -13.954 | -17.726 | 0.00 | 0.00 | PROA |
| ATOM | 761 | HN | SER | P | 48 | 4.603 | -13.667 | -17.257 | 0.00 | 0.00 | PROA |
| ATOM | 762 | CA | SER | P | 48 | 5.976 | -15.170 | -17.145 | 0.00 | 0.00 | PROA |
| ATOM | 763 | HA | SER | P | 48 | 6.587 | -15.699 | -17.861 | 0.00 | 0.00 | PROA |
| ATOM | 764 | CB | SER | P | 48 | 6.865 | -14.915 | -15.842 | 0.00 | 0.00 | PROA |
| ATOM | 765 | HB1 | SER | P | 48 | 7.261 | -15.876 | -15.450 | 0.00 | 0.00 | PROA |
| ATOM | 766 | HB2 | SER | P | 48 | 6.164 | -14.456 | -15.113 | 0.00 | 0.00 | PROA |
| ATOM | 767 | OG | SER | P | 48 | 8.079 | -14.234 | -16.098 | 0.00 | 0.00 | PROA |
| ATOM | 768 | HG1 | SER | P | 48 | 7.852 | -13.317 | -16.266 | 0.00 | 0.00 | PROA |
| ATOM | 769 | C | SER | P | 48 | 4.793 | -16.130 | -16.822 | 0.00 | 0.00 | PROA |
| ATOM | 770 | O | SER | P | 48 | 3.591 | -15.789 | -16.877 | 0.00 | 0.00 | PROA |
| ATOM | 771 | N | GLY | P | 49 | 5.104 | -17.344 | -16.378 | 0.00 | 0.00 | PROA |
| ATOM | 772 | HN | GLY | P | 49 | 5.999 | -17.784 | -16.376 | 0.00 | 0.00 | PROA |
| ATOM | 773 | CA | GLY | P | 49 | 4.051 | -18.214 | -15.901 | 0.00 | 0.00 | PROA |
| ATOM | 774 | HA1 | GLY | P | 49 | 3.515 | -18.715 | -16.694 | 0.00 | 0.00 | PROA |
| ATOM | 775 | HA2 | GLY | P | 49 | 3.441 | -17.531 | -15.330 | 0.00 | 0.00 | PROA |
| ATOM | 776 | C | GLY | P | 49 | 4.656 | -19.273 | -15.128 | 0.00 | 0.00 | PROA |
| ATOM | 777 | O | GLY | P | 49 | 5.768 | -19.278 | -14.610 | 0.00 | 0.00 | PROA |
| ATOM | 778 | N | PHE | P | 50 | 3.890 | -20.375 | -14.950 | 0.00 | 0.00 | PROA |
| ATOM | 779 | HN | PHE | P | 50 | 2.939 | -20.339 | -15.246 | 0.00 | 0.00 | PROA |
| ATOM | 780 | CA | PHE | P | 50 | 4.382 | -21.598 | -14.386 | 0.00 | 0.00 | PROA |
| ATOM | 781 | HA | PHE | P | 50 | 5.431 | -21.570 | -14.640 | 0.00 | 0.00 | PROA |
| ATOM | 782 | CB | PHE | P | 50 | 4.260 | -21.562 | -12.837 | 0.00 | 0.00 | PROA |
| ATOM | 783 | HB1 | PHE | P | 50 | 4.772 | -20.751 | -12.276 | 0.00 | 0.00 | PROA |
| ATOM | 784 | HB2 | PHE | P | 50 | 4.774 | -22.498 | -12.532 | 0.00 | 0.00 | PROA |
| ATOM | 785 | CG | PHE | P | 50 | 2.845 | -21.491 | -12.325 | 0.00 | 0.00 | PROA |
| ATOM | 786 | CD1 | PHE | P | 50 | 2.178 | -20.342 | -12.004 | 0.00 | 0.00 | PROA |
| ATOM | 787 | HD1 | PHE | P | 50 | 2.665 | -19.398 | -12.199 | 0.00 | 0.00 | PROA |
| ATOM | 788 | CE1 | PHE | P | 50 | 0.937 | -20.393 | -11.395 | 0.00 | 0.00 | PROA |
| ATOM | 789 | HE1 | PHE | P | 50 | 0.448 | -19.472 | -11.114 | 0.00 | 0.00 | PROA |
| ATOM | 790 | CZ | PHE | P | 50 | 0.262 | -21.600 | -11.115 | 0.00 | 0.00 | PROA |
| ATOM | 791 | HZ | PHE | P | 50 | -0.634 | -21.642 | -10.514 | 0.00 | 0.00 | PROA |
| ATOM | 792 | CD2 | PHE | P | 50 | 2.167 | -22.735 | -12.103 | 0.00 | 0.00 | PROA |
| ATOM | 793 | HD2 | PHE | P | 50 | 2.611 | -23.654 | -12.454 | 0.00 | 0.00 | PROA |
| ATOM | 794 | CE2 | PHE | P | 50 | 0.883 | -22.768 | -11.528 | 0.00 | 0.00 | PROA |
| ATOM | 795 | HE2 | PHE | P | 50 | 0.466 | -23.720 | -11.236 | 0.00 | 0.00 | PROA |
| ATOM | 796 | C | PHE | P | 50 | 3.798 | -22.832 | -15.095 | 0.00 | 0.00 | PROA |
| ATOM | 797 | O | PHE | P | 50 | 2.732 | -22.814 | -15.726 | 0.00 | 0.00 | PROA |
| ATOM | 798 | N | ILE | P | 51 | 4.525 | -23.941 | -15.046 | 0.00 | 0.00 | PROA |
| ATOM | 799 | HN | ILE | P | 51 | 5.453 | -24.019 | -14.689 | 0.00 | 0.00 | PROA |
| ATOM | 800 | CA | ILE | P | 51 | 4.212 | -25.240 | -15.434 | 0.00 | 0.00 | PROA |
| ATOM | 801 | HA | ILE | P | 51 | 3.848 | -25.274 | -16.450 | 0.00 | 0.00 | PROA |
| ATOM | 802 | CB | ILE | P | 51 | 5.431 | -26.167 | -15.363 | 0.00 | 0.00 | PROA |
| ATOM | 803 | HB | ILE | P | 51 | 5.935 | -25.991 | -14.388 | 0.00 | 0.00 | PROA |

```
ATOM    804  CG2 ILE P  51       5.100 -27.625 -15.662  0.00  0.00      PROA
ATOM    805 HG21 ILE P  51       4.433 -27.669 -16.550  0.00  0.00      PROA
ATOM    806 HG22 ILE P  51       4.559 -27.976 -14.758  0.00  0.00      PROA
ATOM    807 HG23 ILE P  51       6.061 -28.124 -15.907  0.00  0.00      PROA
ATOM    808  CG1 ILE P  51       6.587 -25.612 -16.266  0.00  0.00      PROA
ATOM    809 HG11 ILE P  51       6.960 -24.758 -15.661  0.00  0.00      PROA
ATOM    810 HG12 ILE P  51       6.141 -25.195 -17.194  0.00  0.00      PROA
ATOM    811  CD  ILE P  51       7.769 -26.556 -16.663  0.00  0.00      PROA
ATOM    812  HD1 ILE P  51       7.447 -27.585 -16.931  0.00  0.00      PROA
ATOM    813  HD2 ILE P  51       8.413 -26.663 -15.763  0.00  0.00      PROA
ATOM    814  HD3 ILE P  51       8.197 -26.109 -17.585  0.00  0.00      PROA
ATOM    815  C   ILE P  51       3.136 -25.839 -14.567  0.00  0.00      PROA
ATOM    816  O   ILE P  51       3.454 -26.208 -13.427  0.00  0.00      PROA
ATOM    817  N   VAL P  52       1.942 -25.932 -15.149  0.00  0.00      PROA
ATOM    818  HN  VAL P  52       1.771 -25.654 -16.091  0.00  0.00      PROA
ATOM    819  CA  VAL P  52       0.875 -26.437 -14.248  0.00  0.00      PROA
ATOM    820  HA  VAL P  52       1.215 -26.610 -13.237  0.00  0.00      PROA
ATOM    821  CB  VAL P  52      -0.321 -25.425 -13.984  0.00  0.00      PROA
ATOM    822  HB  VAL P  52       0.192 -24.453 -13.822  0.00  0.00      PROA
ATOM    823  CG1 VAL P  52      -1.189 -25.441 -15.190  0.00  0.00      PROA
ATOM    824 HG11 VAL P  52      -1.918 -24.624 -15.000  0.00  0.00      PROA
ATOM    825 HG12 VAL P  52      -1.817 -26.357 -15.164  0.00  0.00      PROA
ATOM    826 HG13 VAL P  52      -0.725 -25.236 -16.179  0.00  0.00      PROA
ATOM    827  CG2 VAL P  52      -1.054 -25.672 -12.631  0.00  0.00      PROA
ATOM    828 HG21 VAL P  52      -1.650 -26.604 -12.737  0.00  0.00      PROA
ATOM    829 HG22 VAL P  52      -1.751 -24.817 -12.495  0.00  0.00      PROA
ATOM    830 HG23 VAL P  52      -0.374 -25.973 -11.805  0.00  0.00      PROA
ATOM    831  C   VAL P  52       0.423 -27.846 -14.613  0.00  0.00      PROA
ATOM    832  O   VAL P  52      -0.515 -28.402 -14.041  0.00  0.00      PROA
ATOM    833  N   SER P  53       1.082 -28.471 -15.529  0.00  0.00      PROA
ATOM    834  HN  SER P  53       1.851 -28.130 -16.064  0.00  0.00      PROA
ATOM    835  CA  SER P  53       0.813 -29.850 -15.732  0.00  0.00      PROA
ATOM    836  HA  SER P  53       0.550 -30.303 -14.788  0.00  0.00      PROA
ATOM    837  CB  SER P  53      -0.454 -30.115 -16.641  0.00  0.00      PROA
ATOM    838  HB1 SER P  53      -1.308 -29.412 -16.534  0.00  0.00      PROA
ATOM    839  HB2 SER P  53      -0.828 -31.143 -16.451  0.00  0.00      PROA
ATOM    840  OG  SER P  53      -0.177 -30.186 -18.054  0.00  0.00      PROA
ATOM    841  HG1 SER P  53      -1.026 -30.144 -18.500  0.00  0.00      PROA
ATOM    842  C   SER P  53       2.004 -30.598 -16.237  0.00  0.00      PROA
ATOM    843  O   SER P  53       3.035 -30.120 -16.704  0.00  0.00      PROA
ATOM    844  N   GLU P  54       2.010 -31.910 -15.989  0.00  0.00      PROA
ATOM    845  HN  GLU P  54       1.283 -32.389 -15.503  0.00  0.00      PROA
ATOM    846  CA  GLU P  54       3.017 -32.807 -16.427  0.00  0.00      PROA
ATOM    847  HA  GLU P  54       3.904 -32.278 -16.114  0.00  0.00      PROA
ATOM    848  CB  GLU P  54       2.863 -34.197 -15.740  0.00  0.00      PROA
ATOM    849  HB1 GLU P  54       3.707 -34.891 -15.945  0.00  0.00      PROA
ATOM    850  HB2 GLU P  54       1.926 -34.712 -16.041  0.00  0.00      PROA
ATOM    851  CG  GLU P  54       2.850 -34.071 -14.183  0.00  0.00      PROA
ATOM    852  HG1 GLU P  54       1.990 -33.436 -13.881  0.00  0.00      PROA
ATOM    853  HG2 GLU P  54       3.812 -33.621 -13.857  0.00  0.00      PROA
ATOM    854  CD  GLU P  54       2.493 -35.370 -13.483  0.00  0.00      PROA
ATOM    855  OE1 GLU P  54       1.462 -35.388 -12.789  0.00  0.00      PROA
ATOM    856  OE2 GLU P  54       3.264 -36.301 -13.539  0.00  0.00      PROA
ATOM    857  C   GLU P  54       3.135 -33.053 -17.945  0.00  0.00      PROA
ATOM    858  O   GLU P  54       4.218 -33.393 -18.469  0.00  0.00      PROA
ATOM    859  N   ASP P  55       2.011 -32.801 -18.664  0.00  0.00      PROA
ATOM    860  HN  ASP P  55       1.208 -32.635 -18.097  0.00  0.00      PROA
ATOM    861  CA  ASP P  55       1.974 -32.866 -20.102  0.00  0.00      PROA
ATOM    862  HA  ASP P  55       2.829 -33.412 -20.474  0.00  0.00      PROA
ATOM    863  CB  ASP P  55       0.716 -33.520 -20.719  0.00  0.00      PROA
ATOM    864  HB1 ASP P  55       0.658 -33.231 -21.790  0.00  0.00      PROA
ATOM    865  HB2 ASP P  55      -0.095 -33.202 -20.030  0.00  0.00      PROA
ATOM    866  CG  ASP P  55       0.869 -34.991 -20.692  0.00  0.00      PROA
ATOM    867  OD1 ASP P  55      -0.228 -35.675 -20.845  0.00  0.00      PROA
ATOM    868  OD2 ASP P  55       1.963 -35.486 -20.817  0.00  0.00      PROA
ATOM    869  C   ASP P  55       2.283 -31.519 -20.682  0.00  0.00      PROA
ATOM    870  O   ASP P  55       2.202 -31.363 -21.862  0.00  0.00      PROA
ATOM    871  N   GLY P  56       2.731 -30.521 -19.901  0.00  0.00      PROA
ATOM    872  HN  GLY P  56       2.807 -30.614 -18.911  0.00  0.00      PROA
ATOM    873  CA  GLY P  56       3.274 -29.270 -20.438  0.00  0.00      PROA
ATOM    874  HA1 GLY P  56       3.726 -29.336 -21.417  0.00  0.00      PROA
ATOM    875  HA2 GLY P  56       3.996 -28.912 -19.719  0.00  0.00      PROA
ATOM    876  C   GLY P  56       2.281 -28.117 -20.623  0.00  0.00      PROA
```

| | | | | | | | | | | | |
|---|---|---|---|---|---|---|---|---|---|---|---|
| ATOM | 877 | O | GLY | P | 56 | 2.413 | -27.339 | -21.598 | 0.00 | 0.00 | PROA |
| ATOM | 878 | N | LEU | P | 57 | 1.247 | -27.961 | -19.719 | 0.00 | 0.00 | PROA |
| ATOM | 879 | HN | LEU | P | 57 | 1.122 | -28.646 | -19.005 | 0.00 | 0.00 | PROA |
| ATOM | 880 | CA | LEU | P | 57 | 0.360 | -26.775 | -19.689 | 0.00 | 0.00 | PROA |
| ATOM | 881 | HA | LEU | P | 57 | 0.169 | -26.523 | -20.722 | 0.00 | 0.00 | PROA |
| ATOM | 882 | CB | LEU | P | 57 | -1.022 | -27.013 | -19.046 | 0.00 | 0.00 | PROA |
| ATOM | 883 | HB1 | LEU | P | 57 | -1.635 | -26.127 | -18.777 | 0.00 | 0.00 | PROA |
| ATOM | 884 | HB2 | LEU | P | 57 | -0.783 | -27.518 | -18.086 | 0.00 | 0.00 | PROA |
| ATOM | 885 | CG | LEU | P | 57 | -1.982 | -27.965 | -19.881 | 0.00 | 0.00 | PROA |
| ATOM | 886 | HG | LEU | P | 57 | -1.380 | -28.870 | -20.109 | 0.00 | 0.00 | PROA |
| ATOM | 887 | CD1 | LEU | P | 57 | -3.255 | -28.235 | -18.998 | 0.00 | 0.00 | PROA |
| ATOM | 888 | HD11 | LEU | P | 57 | -3.797 | -27.282 | -18.817 | 0.00 | 0.00 | PROA |
| ATOM | 889 | HD12 | LEU | P | 57 | -3.179 | -28.728 | -18.005 | 0.00 | 0.00 | PROA |
| ATOM | 890 | HD13 | LEU | P | 57 | -3.907 | -28.906 | -19.597 | 0.00 | 0.00 | PROA |
| ATOM | 891 | CD2 | LEU | P | 57 | -2.448 | -27.280 | -21.191 | 0.00 | 0.00 | PROA |
| ATOM | 892 | HD21 | LEU | P | 57 | -3.010 | -27.978 | -21.848 | 0.00 | 0.00 | PROA |
| ATOM | 893 | HD22 | LEU | P | 57 | -1.530 | -27.014 | -21.758 | 0.00 | 0.00 | PROA |
| ATOM | 894 | HD23 | LEU | P | 57 | -2.951 | -26.344 | -20.867 | 0.00 | 0.00 | PROA |
| ATOM | 895 | C | LEU | P | 57 | 1.100 | -25.708 | -19.069 | 0.00 | 0.00 | PROA |
| ATOM | 896 | O | LEU | P | 57 | 1.624 | -25.835 | -17.983 | 0.00 | 0.00 | PROA |
| ATOM | 897 | N | ILE | P | 58 | 1.169 | -24.540 | -19.757 | 0.00 | 0.00 | PROA |
| ATOM | 898 | HN | ILE | P | 58 | 1.049 | -24.598 | -20.745 | 0.00 | 0.00 | PROA |
| ATOM | 899 | CA | ILE | P | 58 | 1.718 | -23.343 | -19.138 | 0.00 | 0.00 | PROA |
| ATOM | 900 | HA | ILE | P | 58 | 2.360 | -23.596 | -18.307 | 0.00 | 0.00 | PROA |
| ATOM | 901 | CB | ILE | P | 58 | 2.708 | -22.670 | -20.050 | 0.00 | 0.00 | PROA |
| ATOM | 902 | HB | ILE | P | 58 | 2.258 | -22.430 | -21.037 | 0.00 | 0.00 | PROA |
| ATOM | 903 | CG2 | ILE | P | 58 | 3.356 | -21.325 | -19.420 | 0.00 | 0.00 | PROA |
| ATOM | 904 | HG21 | ILE | P | 58 | 3.838 | -21.495 | -18.434 | 0.00 | 0.00 | PROA |
| ATOM | 905 | HG22 | ILE | P | 58 | 2.516 | -20.599 | -19.450 | 0.00 | 0.00 | PROA |
| ATOM | 906 | HG23 | ILE | P | 58 | 4.050 | -20.900 | -20.177 | 0.00 | 0.00 | PROA |
| ATOM | 907 | CG1 | ILE | P | 58 | 3.846 | -23.725 | -20.429 | 0.00 | 0.00 | PROA |
| ATOM | 908 | HG11 | ILE | P | 58 | 4.522 | -23.200 | -21.137 | 0.00 | 0.00 | PROA |
| ATOM | 909 | HG12 | ILE | P | 58 | 3.435 | -24.532 | -21.071 | 0.00 | 0.00 | PROA |
| ATOM | 910 | CD | ILE | P | 58 | 4.749 | -24.190 | -19.251 | 0.00 | 0.00 | PROA |
| ATOM | 911 | HD1 | ILE | P | 58 | 5.212 | -23.323 | -18.733 | 0.00 | 0.00 | PROA |
| ATOM | 912 | HD2 | ILE | P | 58 | 5.673 | -24.743 | -19.522 | 0.00 | 0.00 | PROA |
| ATOM | 913 | HD3 | ILE | P | 58 | 4.124 | -24.734 | -18.510 | 0.00 | 0.00 | PROA |
| ATOM | 914 | C | ILE | P | 58 | 0.525 | -22.471 | -18.792 | 0.00 | 0.00 | PROA |
| ATOM | 915 | O | ILE | P | 58 | -0.354 | -22.226 | -19.639 | 0.00 | 0.00 | PROA |
| ATOM | 916 | N | VAL | P | 59 | 0.521 | -21.808 | -17.610 | 0.00 | 0.00 | PROA |
| ATOM | 917 | HN | VAL | P | 59 | 1.295 | -21.902 | -16.989 | 0.00 | 0.00 | PROA |
| ATOM | 918 | CA | VAL | P | 59 | -0.565 | -21.020 | -17.107 | 0.00 | 0.00 | PROA |
| ATOM | 919 | HA | VAL | P | 59 | -1.262 | -20.814 | -17.906 | 0.00 | 0.00 | PROA |
| ATOM | 920 | CB | VAL | P | 59 | -1.267 | -21.620 | -15.941 | 0.00 | 0.00 | PROA |
| ATOM | 921 | HB | VAL | P | 59 | -1.636 | -22.651 | -16.130 | 0.00 | 0.00 | PROA |
| ATOM | 922 | CG1 | VAL | P | 59 | -0.484 | -21.551 | -14.582 | 0.00 | 0.00 | PROA |
| ATOM | 923 | HG11 | VAL | P | 59 | -0.185 | -20.541 | -14.227 | 0.00 | 0.00 | PROA |
| ATOM | 924 | HG12 | VAL | P | 59 | 0.464 | -22.053 | -14.871 | 0.00 | 0.00 | PROA |
| ATOM | 925 | HG13 | VAL | P | 59 | -0.926 | -22.043 | -13.689 | 0.00 | 0.00 | PROA |
| ATOM | 926 | CG2 | VAL | P | 59 | -2.669 | -20.907 | -15.865 | 0.00 | 0.00 | PROA |
| ATOM | 927 | HG21 | VAL | P | 59 | -2.678 | -19.847 | -15.531 | 0.00 | 0.00 | PROA |
| ATOM | 928 | HG22 | VAL | P | 59 | -3.279 | -21.502 | -15.153 | 0.00 | 0.00 | PROA |
| ATOM | 929 | HG23 | VAL | P | 59 | -3.283 | -20.872 | -16.791 | 0.00 | 0.00 | PROA |
| ATOM | 930 | C | VAL | P | 59 | -0.011 | -19.656 | -16.837 | 0.00 | 0.00 | PROA |
| ATOM | 931 | O | VAL | P | 59 | 1.102 | -19.437 | -16.396 | 0.00 | 0.00 | PROA |
| ATOM | 932 | N | THR | P | 60 | -0.768 | -18.620 | -17.294 | 0.00 | 0.00 | PROA |
| ATOM | 933 | HN | THR | P | 60 | -1.633 | -18.797 | -17.757 | 0.00 | 0.00 | PROA |
| ATOM | 934 | CA | THR | P | 60 | -0.413 | -17.218 | -17.244 | 0.00 | 0.00 | PROA |
| ATOM | 935 | HA | THR | P | 60 | 0.012 | -17.011 | -16.274 | 0.00 | 0.00 | PROA |
| ATOM | 936 | CB | THR | P | 60 | 0.565 | -16.789 | -18.370 | 0.00 | 0.00 | PROA |
| ATOM | 937 | HB | THR | P | 60 | 1.395 | -17.520 | -18.465 | 0.00 | 0.00 | PROA |
| ATOM | 938 | OG1 | THR | P | 60 | 1.112 | -15.491 | -18.161 | 0.00 | 0.00 | PROA |
| ATOM | 939 | HG1 | THR | P | 60 | 2.008 | -15.639 | -17.851 | 0.00 | 0.00 | PROA |
| ATOM | 940 | CG2 | THR | P | 60 | -0.135 | -16.755 | -19.670 | 0.00 | 0.00 | PROA |
| ATOM | 941 | HG21 | THR | P | 60 | -0.592 | -17.722 | -19.969 | 0.00 | 0.00 | PROA |
| ATOM | 942 | HG22 | THR | P | 60 | 0.668 | -16.437 | -20.369 | 0.00 | 0.00 | PROA |
| ATOM | 943 | HG23 | THR | P | 60 | -0.929 | -15.978 | -19.683 | 0.00 | 0.00 | PROA |
| ATOM | 944 | C | THR | P | 60 | -1.562 | -16.352 | -17.070 | 0.00 | 0.00 | PROA |
| ATOM | 945 | O | THR | P | 60 | -2.644 | -16.859 | -16.803 | 0.00 | 0.00 | PROA |
| ATOM | 946 | N | ASN | P | 61 | -1.410 | -14.996 | -17.234 | 0.00 | 0.00 | PROA |
| ATOM | 947 | HN | ASN | P | 61 | -0.536 | -14.614 | -17.523 | 0.00 | 0.00 | PROA |
| ATOM | 948 | CA | ASN | P | 61 | -2.479 | -14.055 | -17.084 | 0.00 | 0.00 | PROA |
| ATOM | 949 | HA | ASN | P | 61 | -3.259 | -14.585 | -16.558 | 0.00 | 0.00 | PROA |

```
ATOM    950  CB  ASN P  61      -2.094 -12.770 -16.264  0.00  0.00      PROA
ATOM    951  HB1 ASN P  61      -2.997 -12.137 -16.135  0.00  0.00      PROA
ATOM    952  HB2 ASN P  61      -1.485 -12.081 -16.887  0.00  0.00      PROA
ATOM    953  CG  ASN P  61      -1.369 -13.054 -14.911  0.00  0.00      PROA
ATOM    954  OD1 ASN P  61      -0.359 -12.412 -14.802  0.00  0.00      PROA
ATOM    955  ND2 ASN P  61      -1.935 -13.889 -14.075  0.00  0.00      PROA
ATOM    956 HD21 ASN P  61      -1.648 -13.834 -13.119  0.00  0.00      PROA
ATOM    957 HD22 ASN P  61      -2.766 -14.374 -14.349  0.00  0.00      PROA
ATOM    958  C   ASN P  61      -3.159 -13.461 -18.329  0.00  0.00      PROA
ATOM    959  O   ASN P  61      -2.574 -13.403 -19.412  0.00  0.00      PROA
ATOM    960  N   ALA P  62      -4.386 -13.050 -18.280  0.00  0.00      PROA
ATOM    961  HN  ALA P  62      -4.839 -12.775 -17.435  0.00  0.00      PROA
ATOM    962  CA  ALA P  62      -5.187 -12.860 -19.479  0.00  0.00      PROA
ATOM    963  HA  ALA P  62      -4.985 -13.687 -20.144  0.00  0.00      PROA
ATOM    964  CB  ALA P  62      -6.635 -12.861 -18.990  0.00  0.00      PROA
ATOM    965  HB1 ALA P  62      -7.248 -12.614 -19.883  0.00  0.00      PROA
ATOM    966  HB2 ALA P  62      -6.905 -11.942 -18.426  0.00  0.00      PROA
ATOM    967  HB3 ALA P  62      -6.982 -13.776 -18.464  0.00  0.00      PROA
ATOM    968  C   ALA P  62      -4.822 -11.569 -20.226  0.00  0.00      PROA
ATOM    969  O   ALA P  62      -5.202 -11.480 -21.400  0.00  0.00      PROA
ATOM    970  N   HSD P  63      -4.113 -10.607 -19.563  0.00  0.00      PROA
ATOM    971  HN  HSD P  63      -4.031 -10.855 -18.600  0.00  0.00      PROA
ATOM    972  CA  HSD P  63      -3.688  -9.308 -20.075  0.00  0.00      PROA
ATOM    973  HA  HSD P  63      -4.384  -8.882 -20.783  0.00  0.00      PROA
ATOM    974  CB  HSD P  63      -3.616  -8.277 -18.868  0.00  0.00      PROA
ATOM    975  HB1 HSD P  63      -4.636  -8.217 -18.431  0.00  0.00      PROA
ATOM    976  HB2 HSD P  63      -3.446  -7.278 -19.322  0.00  0.00      PROA
ATOM    977  ND1 HSD P  63      -2.754  -9.251 -16.517  0.00  0.00      PROA
ATOM    978  HD1 HSD P  63      -3.646  -9.614 -16.250  0.00  0.00      PROA
ATOM    979  CG  HSD P  63      -2.521  -8.699 -17.778  0.00  0.00      PROA
ATOM    980  CE1 HSD P  63      -1.603  -9.330 -15.839  0.00  0.00      PROA
ATOM    981  HE1 HSD P  63      -1.409  -9.816 -14.883  0.00  0.00      PROA
ATOM    982  NE2 HSD P  63      -0.621  -8.793 -16.574  0.00  0.00      PROA
ATOM    983  CD2 HSD P  63      -1.166  -8.404 -17.795  0.00  0.00      PROA
ATOM    984  HD2 HSD P  63      -0.712  -8.007 -18.695  0.00  0.00      PROA
ATOM    985  C   HSD P  63      -2.365  -9.394 -20.809  0.00  0.00      PROA
ATOM    986  O   HSD P  63      -1.865  -8.345 -21.223  0.00  0.00      PROA
ATOM    987  N   VAL P  64      -1.781 -10.593 -21.070  0.00  0.00      PROA
ATOM    988  HN  VAL P  64      -2.227 -11.390 -20.671  0.00  0.00      PROA
ATOM    989  CA  VAL P  64      -0.532 -10.794 -21.688  0.00  0.00      PROA
ATOM    990  HA  VAL P  64      -0.095  -9.839 -21.937  0.00  0.00      PROA
ATOM    991  CB  VAL P  64       0.401 -11.687 -20.827  0.00  0.00      PROA
ATOM    992  HB  VAL P  64      -0.056 -12.613 -20.419  0.00  0.00      PROA
ATOM    993  CG1 VAL P  64       1.615 -12.159 -21.609  0.00  0.00      PROA
ATOM    994 HG11 VAL P  64       2.047 -11.259 -22.097  0.00  0.00      PROA
ATOM    995 HG12 VAL P  64       1.376 -12.894 -22.407  0.00  0.00      PROA
ATOM    996 HG13 VAL P  64       2.397 -12.586 -20.946  0.00  0.00      PROA
ATOM    997  CG2 VAL P  64       0.941 -10.920 -19.665  0.00  0.00      PROA
ATOM    998 HG21 VAL P  64       0.139 -10.518 -19.009  0.00  0.00      PROA
ATOM    999 HG22 VAL P  64       1.370  -9.980 -20.073  0.00  0.00      PROA
ATOM   1000 HG23 VAL P  64       1.686 -11.495 -19.074  0.00  0.00      PROA
ATOM   1001  C   VAL P  64      -0.691 -11.371 -23.031  0.00  0.00      PROA
ATOM   1002  O   VAL P  64      -0.004 -11.160 -24.009  0.00  0.00      PROA
ATOM   1003  N   VAL P  65      -1.853 -11.953 -23.186  0.00  0.00      PROA
ATOM   1004  HN  VAL P  65      -2.386 -11.816 -22.354  0.00  0.00      PROA
ATOM   1005  CA  VAL P  65      -2.510 -12.512 -24.377  0.00  0.00      PROA
ATOM   1006  HA  VAL P  65      -1.928 -13.360 -24.707  0.00  0.00      PROA
ATOM   1007  CB  VAL P  65      -3.854 -13.281 -24.066  0.00  0.00      PROA
ATOM   1008  HB  VAL P  65      -4.562 -12.498 -23.718  0.00  0.00      PROA
ATOM   1009  CG1 VAL P  65      -4.444 -13.852 -25.359  0.00  0.00      PROA
ATOM   1010 HG11 VAL P  65      -3.830 -14.525 -25.994  0.00  0.00      PROA
ATOM   1011 HG12 VAL P  65      -4.811 -13.050 -26.035  0.00  0.00      PROA
ATOM   1012 HG13 VAL P  65      -5.431 -14.266 -25.060  0.00  0.00      PROA
ATOM   1013  CG2 VAL P  65      -3.725 -14.346 -22.989  0.00  0.00      PROA
ATOM   1014 HG21 VAL P  65      -3.605 -13.866 -21.995  0.00  0.00      PROA
ATOM   1015 HG22 VAL P  65      -2.903 -15.040 -23.268  0.00  0.00      PROA
ATOM   1016 HG23 VAL P  65      -4.630 -14.952 -22.770  0.00  0.00      PROA
ATOM   1017  C   VAL P  65      -2.478 -11.540 -25.610  0.00  0.00      PROA
ATOM   1018  O   VAL P  65      -2.673 -10.324 -25.518  0.00  0.00      PROA
ATOM   1019  N   THR P  66      -2.291 -12.056 -26.813  0.00  0.00      PROA
ATOM   1020  HN  THR P  66      -2.143 -13.030 -26.966  0.00  0.00      PROA
ATOM   1021  CA  THR P  66      -2.646 -11.328 -27.972  0.00  0.00      PROA
ATOM   1022  HA  THR P  66      -3.632 -10.897 -27.878  0.00  0.00      PROA
```

```
ATOM   1023  CB  THR P  66      -1.650 -10.138 -28.415  0.00  0.00      PROA
ATOM   1024  HB  THR P  66      -1.936  -9.271 -27.781  0.00  0.00      PROA
ATOM   1025  OG1 THR P  66      -1.814  -9.814 -29.817  0.00  0.00      PROA
ATOM   1026  HG1 THR P  66      -2.585  -9.267 -29.980  0.00  0.00      PROA
ATOM   1027  CG2 THR P  66      -0.168 -10.561 -28.188  0.00  0.00      PROA
ATOM   1028 HG21 THR P  66       0.020 -10.665 -27.098  0.00  0.00      PROA
ATOM   1029 HG22 THR P  66       0.549  -9.810 -28.583  0.00  0.00      PROA
ATOM   1030 HG23 THR P  66      -0.019 -11.497 -28.768  0.00  0.00      PROA
ATOM   1031  C   THR P  66      -2.843 -12.426 -29.043  0.00  0.00      PROA
ATOM   1032  O   THR P  66      -2.175 -13.462 -29.057  0.00  0.00      PROA
ATOM   1033  N   ASN P  67      -3.816 -12.215 -29.954  0.00  0.00      PROA
ATOM   1034  HN  ASN P  67      -4.298 -11.344 -30.007  0.00  0.00      PROA
ATOM   1035  CA  ASN P  67      -4.103 -13.135 -31.048  0.00  0.00      PROA
ATOM   1036  HA  ASN P  67      -3.784 -14.127 -30.766  0.00  0.00      PROA
ATOM   1037  CB  ASN P  67      -5.605 -13.132 -31.434  0.00  0.00      PROA
ATOM   1038  HB1 ASN P  67      -5.791 -13.809 -32.295  0.00  0.00      PROA
ATOM   1039  HB2 ASN P  67      -5.961 -12.125 -31.736  0.00  0.00      PROA
ATOM   1040  CG  ASN P  67      -6.570 -13.518 -30.287  0.00  0.00      PROA
ATOM   1041  OD1 ASN P  67      -7.495 -12.829 -30.055  0.00  0.00      PROA
ATOM   1042  ND2 ASN P  67      -6.342 -14.688 -29.545  0.00  0.00      PROA
ATOM   1043 HD21 ASN P  67      -6.867 -14.794 -28.700  0.00  0.00      PROA
ATOM   1044 HD22 ASN P  67      -5.822 -15.437 -29.956  0.00  0.00      PROA
ATOM   1045  C   ASN P  67      -3.298 -12.711 -32.265  0.00  0.00      PROA
ATOM   1046  O   ASN P  67      -3.328 -13.564 -33.170  0.00  0.00      PROA
ATOM   1047  N   LYS P  68      -2.523 -11.656 -32.232  0.00  0.00      PROA
ATOM   1048  HN  LYS P  68      -2.535 -11.052 -31.439  0.00  0.00      PROA
ATOM   1049  CA  LYS P  68      -1.948 -11.074 -33.443  0.00  0.00      PROA
ATOM   1050  HA  LYS P  68      -2.263 -11.526 -34.372  0.00  0.00      PROA
ATOM   1051  CB  LYS P  68      -2.306  -9.518 -33.520  0.00  0.00      PROA
ATOM   1052  HB1 LYS P  68      -1.921  -9.020 -34.435  0.00  0.00      PROA
ATOM   1053  HB2 LYS P  68      -1.932  -8.957 -32.637  0.00  0.00      PROA
ATOM   1054  CG  LYS P  68      -3.884  -9.366 -33.572  0.00  0.00      PROA
ATOM   1055  HG1 LYS P  68      -4.439  -9.613 -32.642  0.00  0.00      PROA
ATOM   1056  HG2 LYS P  68      -4.326 -10.065 -34.314  0.00  0.00      PROA
ATOM   1057  CD  LYS P  68      -4.224  -7.832 -33.761  0.00  0.00      PROA
ATOM   1058  HD1 LYS P  68      -4.113  -7.524 -34.822  0.00  0.00      PROA
ATOM   1059  HD2 LYS P  68      -3.526  -7.238 -33.133  0.00  0.00      PROA
ATOM   1060  CE  LYS P  68      -5.706  -7.408 -33.386  0.00  0.00      PROA
ATOM   1061  HE1 LYS P  68      -5.833  -6.325 -33.173  0.00  0.00      PROA
ATOM   1062  HE2 LYS P  68      -5.994  -8.053 -32.529  0.00  0.00      PROA
ATOM   1063  NZ  LYS P  68      -6.653  -7.836 -34.409  0.00  0.00      PROA
ATOM   1064  HZ1 LYS P  68      -7.015  -6.959 -34.836  0.00  0.00      PROA
ATOM   1065  HZ2 LYS P  68      -7.353  -8.510 -34.038  0.00  0.00      PROA
ATOM   1066  HZ3 LYS P  68      -6.122  -8.230 -35.211  0.00  0.00      PROA
ATOM   1067  C   LYS P  68      -0.460 -11.308 -33.589  0.00  0.00      PROA
ATOM   1068  O   LYS P  68       0.084 -10.845 -34.624  0.00  0.00      PROA
ATOM   1069  N   HSD P  69       0.220 -11.931 -32.639  0.00  0.00      PROA
ATOM   1070  HN  HSD P  69      -0.142 -12.346 -31.808  0.00  0.00      PROA
ATOM   1071  CA  HSD P  69       1.600 -12.123 -32.625  0.00  0.00      PROA
ATOM   1072  HA  HSD P  69       1.971 -12.083 -33.639  0.00  0.00      PROA
ATOM   1073  CB  HSD P  69       2.281 -10.973 -31.725  0.00  0.00      PROA
ATOM   1074  HB1 HSD P  69       3.389 -11.027 -31.796  0.00  0.00      PROA
ATOM   1075  HB2 HSD P  69       2.088 -11.165 -30.648  0.00  0.00      PROA
ATOM   1076  ND1 HSD P  69       0.816  -8.891 -31.520  0.00  0.00      PROA
ATOM   1077  HD1 HSD P  69       0.199  -8.991 -30.740  0.00  0.00      PROA
ATOM   1078  CG  HSD P  69       1.744  -9.677 -32.145  0.00  0.00      PROA
ATOM   1079  CE1 HSD P  69       0.756  -7.753 -32.267  0.00  0.00      PROA
ATOM   1080  HE1 HSD P  69      -0.016  -6.990 -32.158  0.00  0.00      PROA
ATOM   1081  NE2 HSD P  69       1.631  -7.662 -33.191  0.00  0.00      PROA
ATOM   1082  CD2 HSD P  69       2.264  -8.896 -33.139  0.00  0.00      PROA
ATOM   1083  HD2 HSD P  69       2.942  -9.170 -33.938  0.00  0.00      PROA
ATOM   1084  C   HSD P  69       1.894 -13.545 -32.107  0.00  0.00      PROA
ATOM   1085  O   HSD P  69       0.974 -14.273 -31.753  0.00  0.00      PROA
ATOM   1086  N   ARG P  70       3.119 -14.026 -32.317  0.00  0.00      PROA
ATOM   1087  HN  ARG P  70       3.801 -13.368 -32.625  0.00  0.00      PROA
ATOM   1088  CA  ARG P  70       3.571 -15.405 -32.034  0.00  0.00      PROA
ATOM   1089  HA  ARG P  70       2.804 -16.057 -32.424  0.00  0.00      PROA
ATOM   1090  CB  ARG P  70       4.877 -15.740 -32.852  0.00  0.00      PROA
ATOM   1091  HB1 ARG P  70       5.212 -16.790 -32.716  0.00  0.00      PROA
ATOM   1092  HB2 ARG P  70       5.657 -15.007 -32.552  0.00  0.00      PROA
ATOM   1093  CG  ARG P  70       4.659 -15.648 -34.435  0.00  0.00      PROA
ATOM   1094  HG1 ARG P  70       3.652 -15.240 -34.668  0.00  0.00      PROA
ATOM   1095  HG2 ARG P  70       4.599 -16.697 -34.797  0.00  0.00      PROA
```

| | | | | | | | | | | | |
|---|---|---|---|---|---|---|---|---|---|---|---|
| ATOM | 1096 | CD | ARG | P | 70 | 5.691 | -14.814 | -35.143 | 0.00 | 0.00 | PROA |
| ATOM | 1097 | HD1 | ARG | P | 70 | 6.653 | -15.366 | -35.070 | 0.00 | 0.00 | PROA |
| ATOM | 1098 | HD2 | ARG | P | 70 | 5.829 | -13.819 | -34.670 | 0.00 | 0.00 | PROA |
| ATOM | 1099 | NE | ARG | P | 70 | 5.408 | -14.696 | -36.582 | 0.00 | 0.00 | PROA |
| ATOM | 1100 | HE | ARG | P | 70 | 5.102 | -13.801 | -36.906 | 0.00 | 0.00 | PROA |
| ATOM | 1101 | CZ | ARG | P | 70 | 5.148 | -15.619 | -37.465 | 0.00 | 0.00 | PROA |
| ATOM | 1102 | NH1 | ARG | P | 70 | 5.460 | -16.889 | -37.268 | 0.00 | 0.00 | PROA |
| ATOM | 1103 | HH11 | ARG | P | 70 | 6.111 | -17.190 | -36.571 | 0.00 | 0.00 | PROA |
| ATOM | 1104 | HH12 | ARG | P | 70 | 5.331 | -17.534 | -38.021 | 0.00 | 0.00 | PROA |
| ATOM | 1105 | NH2 | ARG | P | 70 | 4.464 | -15.206 | -38.534 | 0.00 | 0.00 | PROA |
| ATOM | 1106 | HH21 | ARG | P | 70 | 4.071 | -14.287 | -38.529 | 0.00 | 0.00 | PROA |
| ATOM | 1107 | HH22 | ARG | P | 70 | 4.763 | -15.611 | -39.398 | 0.00 | 0.00 | PROA |
| ATOM | 1108 | C | ARG | P | 70 | 3.821 | -15.628 | -30.512 | 0.00 | 0.00 | PROA |
| ATOM | 1109 | O | ARG | P | 70 | 4.240 | -14.677 | -29.840 | 0.00 | 0.00 | PROA |
| ATOM | 1110 | N | VAL | P | 71 | 3.598 | -16.780 | -29.931 | 0.00 | 0.00 | PROA |
| ATOM | 1111 | HN | VAL | P | 71 | 3.074 | -17.430 | -30.477 | 0.00 | 0.00 | PROA |
| ATOM | 1112 | CA | VAL | P | 71 | 3.871 | -17.233 | -28.574 | 0.00 | 0.00 | PROA |
| ATOM | 1113 | HA | VAL | P | 71 | 4.188 | -16.416 | -27.943 | 0.00 | 0.00 | PROA |
| ATOM | 1114 | CB | VAL | P | 71 | 2.691 | -17.654 | -27.734 | 0.00 | 0.00 | PROA |
| ATOM | 1115 | HB | VAL | P | 71 | 2.256 | -18.550 | -28.225 | 0.00 | 0.00 | PROA |
| ATOM | 1116 | CG1 | VAL | P | 71 | 3.096 | -18.069 | -26.343 | 0.00 | 0.00 | PROA |
| ATOM | 1117 | HG11 | VAL | P | 71 | 3.645 | -19.035 | -26.334 | 0.00 | 0.00 | PROA |
| ATOM | 1118 | HG12 | VAL | P | 71 | 2.211 | -18.104 | -25.673 | 0.00 | 0.00 | PROA |
| ATOM | 1119 | HG13 | VAL | P | 71 | 3.741 | -17.292 | -25.881 | 0.00 | 0.00 | PROA |
| ATOM | 1120 | CG2 | VAL | P | 71 | 1.721 | -16.467 | -27.766 | 0.00 | 0.00 | PROA |
| ATOM | 1121 | HG21 | VAL | P | 71 | 1.271 | -16.353 | -28.775 | 0.00 | 0.00 | PROA |
| ATOM | 1122 | HG22 | VAL | P | 71 | 2.238 | -15.629 | -27.250 | 0.00 | 0.00 | PROA |
| ATOM | 1123 | HG23 | VAL | P | 71 | 0.872 | -16.799 | -27.131 | 0.00 | 0.00 | PROA |
| ATOM | 1124 | C | VAL | P | 71 | 5.045 | -18.251 | -28.451 | 0.00 | 0.00 | PROA |
| ATOM | 1125 | O | VAL | P | 71 | 5.001 | -19.312 | -29.032 | 0.00 | 0.00 | PROA |
| ATOM | 1126 | N | LYS | P | 72 | 6.058 | -17.915 | -27.667 | 0.00 | 0.00 | PROA |
| ATOM | 1127 | HN | LYS | P | 72 | 6.213 | -17.007 | -27.285 | 0.00 | 0.00 | PROA |
| ATOM | 1128 | CA | LYS | P | 72 | 7.055 | -18.846 | -27.290 | 0.00 | 0.00 | PROA |
| ATOM | 1129 | HA | LYS | P | 72 | 6.698 | -19.858 | -27.408 | 0.00 | 0.00 | PROA |
| ATOM | 1130 | CB | LYS | P | 72 | 8.340 | -18.662 | -28.084 | 0.00 | 0.00 | PROA |
| ATOM | 1131 | HB1 | LYS | P | 72 | 8.040 | -18.864 | -29.135 | 0.00 | 0.00 | PROA |
| ATOM | 1132 | HB2 | LYS | P | 72 | 9.014 | -19.508 | -27.829 | 0.00 | 0.00 | PROA |
| ATOM | 1133 | CG | LYS | P | 72 | 9.113 | -17.307 | -28.070 | 0.00 | 0.00 | PROA |
| ATOM | 1134 | HG1 | LYS | P | 72 | 9.363 | -16.905 | -27.065 | 0.00 | 0.00 | PROA |
| ATOM | 1135 | HG2 | LYS | P | 72 | 8.469 | -16.558 | -28.578 | 0.00 | 0.00 | PROA |
| ATOM | 1136 | CD | LYS | P | 72 | 10.462 | -17.393 | -28.903 | 0.00 | 0.00 | PROA |
| ATOM | 1137 | HD1 | LYS | P | 72 | 10.393 | -18.105 | -29.753 | 0.00 | 0.00 | PROA |
| ATOM | 1138 | HD2 | LYS | P | 72 | 11.325 | -17.647 | -28.250 | 0.00 | 0.00 | PROA |
| ATOM | 1139 | CE | LYS | P | 72 | 10.844 | -15.989 | -29.389 | 0.00 | 0.00 | PROA |
| ATOM | 1140 | HE1 | LYS | P | 72 | 10.655 | -15.391 | -28.472 | 0.00 | 0.00 | PROA |
| ATOM | 1141 | HE2 | LYS | P | 72 | 10.218 | -15.587 | -30.214 | 0.00 | 0.00 | PROA |
| ATOM | 1142 | NZ | LYS | P | 72 | 12.225 | -15.919 | -29.918 | 0.00 | 0.00 | PROA |
| ATOM | 1143 | HZ1 | LYS | P | 72 | 12.888 | -16.435 | -29.305 | 0.00 | 0.00 | PROA |
| ATOM | 1144 | HZ2 | LYS | P | 72 | 12.482 | -14.911 | -29.931 | 0.00 | 0.00 | PROA |
| ATOM | 1145 | HZ3 | LYS | P | 72 | 12.249 | -16.252 | -30.903 | 0.00 | 0.00 | PROA |
| ATOM | 1146 | C | LYS | P | 72 | 7.220 | -18.761 | -25.806 | 0.00 | 0.00 | PROA |
| ATOM | 1147 | O | LYS | P | 72 | 7.160 | -17.739 | -25.123 | 0.00 | 0.00 | PROA |
| ATOM | 1148 | N | VAL | P | 73 | 7.358 | -19.944 | -25.208 | 0.00 | 0.00 | PROA |
| ATOM | 1149 | HN | VAL | P | 73 | 7.356 | -20.768 | -25.769 | 0.00 | 0.00 | PROA |
| ATOM | 1150 | CA | VAL | P | 73 | 7.549 | -20.155 | -23.795 | 0.00 | 0.00 | PROA |
| ATOM | 1151 | HA | VAL | P | 73 | 7.375 | -19.232 | -23.262 | 0.00 | 0.00 | PROA |
| ATOM | 1152 | CB | VAL | P | 73 | 6.516 | -21.199 | -23.165 | 0.00 | 0.00 | PROA |
| ATOM | 1153 | HB | VAL | P | 73 | 6.562 | -22.240 | -23.551 | 0.00 | 0.00 | PROA |
| ATOM | 1154 | CG1 | VAL | P | 73 | 6.826 | -21.130 | -21.595 | 0.00 | 0.00 | PROA |
| ATOM | 1155 | HG11 | VAL | P | 73 | 7.832 | -21.503 | -21.309 | 0.00 | 0.00 | PROA |
| ATOM | 1156 | HG12 | VAL | P | 73 | 6.098 | -21.777 | -21.058 | 0.00 | 0.00 | PROA |
| ATOM | 1157 | HG13 | VAL | P | 73 | 6.843 | -20.063 | -21.287 | 0.00 | 0.00 | PROA |
| ATOM | 1158 | CG2 | VAL | P | 73 | 5.091 | -20.823 | -23.440 | 0.00 | 0.00 | PROA |
| ATOM | 1159 | HG21 | VAL | P | 73 | 4.461 | -21.643 | -23.034 | 0.00 | 0.00 | PROA |
| ATOM | 1160 | HG22 | VAL | P | 73 | 4.942 | -20.606 | -24.519 | 0.00 | 0.00 | PROA |
| ATOM | 1161 | HG23 | VAL | P | 73 | 4.845 | -19.894 | -22.881 | 0.00 | 0.00 | PROA |
| ATOM | 1162 | C | VAL | P | 73 | 8.993 | -20.576 | -23.732 | 0.00 | 0.00 | PROA |
| ATOM | 1163 | O | VAL | P | 73 | 9.448 | -21.670 | -24.142 | 0.00 | 0.00 | PROA |
| ATOM | 1164 | N | GLU | P | 74 | 9.730 | -19.816 | -22.967 | 0.00 | 0.00 | PROA |
| ATOM | 1165 | HN | GLU | P | 74 | 9.245 | -19.196 | -22.354 | 0.00 | 0.00 | PROA |
| ATOM | 1166 | CA | GLU | P | 74 | 11.142 | -20.006 | -22.649 | 0.00 | 0.00 | PROA |
| ATOM | 1167 | HA | GLU | P | 74 | 11.623 | -20.547 | -23.452 | 0.00 | 0.00 | PROA |
| ATOM | 1168 | CB | GLU | P | 74 | 11.883 | -18.666 | -22.475 | 0.00 | 0.00 | PROA |

```
ATOM   1169  HB1 GLU P  74      11.293 -18.166 -21.677  0.00  0.00      PROA
ATOM   1170  HB2 GLU P  74      11.714 -17.997 -23.345  0.00  0.00      PROA
ATOM   1171  CG  GLU P  74      13.403 -18.668 -22.146  0.00  0.00      PROA
ATOM   1172  HG1 GLU P  74      14.037 -19.318 -22.786  0.00  0.00      PROA
ATOM   1173  HG2 GLU P  74      13.576 -19.000 -21.100  0.00  0.00      PROA
ATOM   1174  CD  GLU P  74      13.983 -17.245 -22.185  0.00  0.00      PROA
ATOM   1175  OE1 GLU P  74      14.795 -16.881 -23.043  0.00  0.00      PROA
ATOM   1176  OE2 GLU P  74      13.454 -16.531 -21.306  0.00  0.00      PROA
ATOM   1177  C   GLU P  74      11.283 -20.715 -21.251  0.00  0.00      PROA
ATOM   1178  O   GLU P  74      11.096 -20.158 -20.207  0.00  0.00      PROA
ATOM   1179  N   LEU P  75      11.697 -22.026 -21.249  0.00  0.00      PROA
ATOM   1180  HN  LEU P  75      12.131 -22.462 -22.033  0.00  0.00      PROA
ATOM   1181  CA  LEU P  75      11.806 -22.739 -20.020  0.00  0.00      PROA
ATOM   1182  HA  LEU P  75      11.139 -22.302 -19.292  0.00  0.00      PROA
ATOM   1183  CB  LEU P  75      11.565 -24.257 -20.213  0.00  0.00      PROA
ATOM   1184  HB1 LEU P  75      11.395 -24.814 -19.267  0.00  0.00      PROA
ATOM   1185  HB2 LEU P  75      12.362 -24.779 -20.785  0.00  0.00      PROA
ATOM   1186  CG  LEU P  75      10.267 -24.660 -20.959  0.00  0.00      PROA
ATOM   1187  HG  LEU P  75      10.165 -24.115 -21.922  0.00  0.00      PROA
ATOM   1188  CD1 LEU P  75      10.170 -26.201 -21.181  0.00  0.00      PROA
ATOM   1189 HD11 LEU P  75       9.770 -26.737 -20.293  0.00  0.00      PROA
ATOM   1190 HD12 LEU P  75      11.165 -26.517 -21.562  0.00  0.00      PROA
ATOM   1191 HD13 LEU P  75       9.393 -26.430 -21.941  0.00  0.00      PROA
ATOM   1192  CD2 LEU P  75       9.059 -24.111 -20.161  0.00  0.00      PROA
ATOM   1193 HD21 LEU P  75       8.162 -24.190 -20.812  0.00  0.00      PROA
ATOM   1194 HD22 LEU P  75       9.093 -23.012 -20.000  0.00  0.00      PROA
ATOM   1195 HD23 LEU P  75       8.833 -24.624 -19.202  0.00  0.00      PROA
ATOM   1196  C   LEU P  75      13.218 -22.603 -19.419  0.00  0.00      PROA
ATOM   1197  O   LEU P  75      14.156 -22.219 -20.094  0.00  0.00      PROA
ATOM   1198  N   LYS P  76      13.275 -22.867 -18.117  0.00  0.00      PROA
ATOM   1199  HN  LYS P  76      12.555 -23.345 -17.619  0.00  0.00      PROA
ATOM   1200  CA  LYS P  76      14.479 -22.605 -17.358  0.00  0.00      PROA
ATOM   1201  HA  LYS P  76      15.073 -21.877 -17.892  0.00  0.00      PROA
ATOM   1202  CB  LYS P  76      14.036 -22.051 -15.956  0.00  0.00      PROA
ATOM   1203  HB1 LYS P  76      14.975 -22.067 -15.363  0.00  0.00      PROA
ATOM   1204  HB2 LYS P  76      13.317 -22.811 -15.580  0.00  0.00      PROA
ATOM   1205  CG  LYS P  76      13.573 -20.586 -15.950  0.00  0.00      PROA
ATOM   1206  HG1 LYS P  76      13.453 -20.231 -14.904  0.00  0.00      PROA
ATOM   1207  HG2 LYS P  76      12.547 -20.650 -16.370  0.00  0.00      PROA
ATOM   1208  CD  LYS P  76      14.317 -19.602 -16.871  0.00  0.00      PROA
ATOM   1209  HD1 LYS P  76      13.720 -18.670 -16.773  0.00  0.00      PROA
ATOM   1210  HD2 LYS P  76      14.095 -19.955 -17.901  0.00  0.00      PROA
ATOM   1211  CE  LYS P  76      15.813 -19.454 -16.602  0.00  0.00      PROA
ATOM   1212  HE1 LYS P  76      16.274 -20.443 -16.389  0.00  0.00      PROA
ATOM   1213  HE2 LYS P  76      15.961 -18.820 -15.702  0.00  0.00      PROA
ATOM   1214  NZ  LYS P  76      16.465 -18.651 -17.645  0.00  0.00      PROA
ATOM   1215  HZ1 LYS P  76      16.668 -19.192 -18.509  0.00  0.00      PROA
ATOM   1216  HZ2 LYS P  76      17.356 -18.158 -17.431  0.00  0.00      PROA
ATOM   1217  HZ3 LYS P  76      15.865 -17.953 -18.130  0.00  0.00      PROA
ATOM   1218  C   LYS P  76      15.445 -23.801 -17.240  0.00  0.00      PROA
ATOM   1219  O   LYS P  76      16.677 -23.684 -17.112  0.00  0.00      PROA
ATOM   1220  N   ASN P  77      14.811 -25.028 -17.408  0.00  0.00      PROA
ATOM   1221  HN  ASN P  77      13.837 -25.192 -17.539  0.00  0.00      PROA
ATOM   1222  CA  ASN P  77      15.472 -26.242 -17.810  0.00  0.00      PROA
ATOM   1223  HA  ASN P  77      16.483 -26.003 -18.106  0.00  0.00      PROA
ATOM   1224  CB  ASN P  77      15.390 -27.293 -16.694  0.00  0.00      PROA
ATOM   1225  HB1 ASN P  77      14.323 -27.532 -16.496  0.00  0.00      PROA
ATOM   1226  HB2 ASN P  77      15.834 -26.876 -15.766  0.00  0.00      PROA
ATOM   1227  CG  ASN P  77      16.258 -28.501 -16.913  0.00  0.00      PROA
ATOM   1228  OD1 ASN P  77      17.203 -28.460 -17.735  0.00  0.00      PROA
ATOM   1229  ND2 ASN P  77      16.037 -29.584 -16.163  0.00  0.00      PROA
ATOM   1230 HD21 ASN P  77      16.727 -30.307 -16.113  0.00  0.00      PROA
ATOM   1231 HD22 ASN P  77      15.383 -29.622 -15.407  0.00  0.00      PROA
ATOM   1232  C   ASN P  77      14.661 -26.744 -19.023  0.00  0.00      PROA
ATOM   1233  O   ASN P  77      13.443 -26.600 -19.123  0.00  0.00      PROA
ATOM   1234  N   GLY P  78      15.290 -27.266 -20.067  0.00  0.00      PROA
ATOM   1235  HN  GLY P  78      16.286 -27.284 -20.089  0.00  0.00      PROA
ATOM   1236  CA  GLY P  78      14.665 -27.633 -21.262  0.00  0.00      PROA
ATOM   1237  HA1 GLY P  78      13.628 -27.934 -21.237  0.00  0.00      PROA
ATOM   1238  HA2 GLY P  78      15.186 -28.525 -21.575  0.00  0.00      PROA
ATOM   1239  C   GLY P  78      14.771 -26.476 -22.278  0.00  0.00      PROA
ATOM   1240  O   GLY P  78      15.265 -25.398 -21.936  0.00  0.00      PROA
ATOM   1241  N   ALA P  79      14.352 -26.717 -23.517  0.00  0.00      PROA
```

```
ATOM   1242  HN  ALA P  79      13.996 -27.623 -23.731  0.00  0.00      PROA
ATOM   1243  CA  ALA P  79      14.442 -25.777 -24.577  0.00  0.00      PROA
ATOM   1244  HA  ALA P  79      15.479 -25.475 -24.594  0.00  0.00      PROA
ATOM   1245  CB  ALA P  79      14.264 -26.637 -25.777  0.00  0.00      PROA
ATOM   1246  HB1 ALA P  79      15.076 -27.396 -25.813  0.00  0.00      PROA
ATOM   1247  HB2 ALA P  79      14.210 -25.976 -26.668  0.00  0.00      PROA
ATOM   1248  HB3 ALA P  79      13.270 -27.132 -25.748  0.00  0.00      PROA
ATOM   1249  C   ALA P  79      13.619 -24.511 -24.529  0.00  0.00      PROA
ATOM   1250  O   ALA P  79      12.841 -24.360 -23.589  0.00  0.00      PROA
ATOM   1251  N   THR P  80      13.735 -23.526 -25.404  0.00  0.00      PROA
ATOM   1252  HN  THR P  80      14.494 -23.556 -26.049  0.00  0.00      PROA
ATOM   1253  CA  THR P  80      12.717 -22.520 -25.562  0.00  0.00      PROA
ATOM   1254  HA  THR P  80      12.152 -22.430 -24.646  0.00  0.00      PROA
ATOM   1255  CB  THR P  80      13.254 -21.131 -26.056  0.00  0.00      PROA
ATOM   1256  HB  THR P  80      14.039 -21.129 -26.842  0.00  0.00      PROA
ATOM   1257  OG1 THR P  80      13.981 -20.564 -24.934  0.00  0.00      PROA
ATOM   1258  HG1 THR P  80      14.105 -21.260 -24.283  0.00  0.00      PROA
ATOM   1259  CG2 THR P  80      12.156 -20.111 -26.317  0.00  0.00      PROA
ATOM   1260 HG21 THR P  80      11.544 -19.919 -25.411  0.00  0.00      PROA
ATOM   1261 HG22 THR P  80      11.379 -20.438 -27.041  0.00  0.00      PROA
ATOM   1262 HG23 THR P  80      12.564 -19.097 -26.513  0.00  0.00      PROA
ATOM   1263  C   THR P  80      11.828 -23.039 -26.625  0.00  0.00      PROA
ATOM   1264  O   THR P  80      12.239 -23.579 -27.628  0.00  0.00      PROA
ATOM   1265  N   TYR P  81      10.479 -22.981 -26.401  0.00  0.00      PROA
ATOM   1266  HN  TYR P  81      10.122 -22.317 -25.749  0.00  0.00      PROA
ATOM   1267  CA  TYR P  81       9.460 -23.714 -27.115  0.00  0.00      PROA
ATOM   1268  HA  TYR P  81       9.814 -24.281 -27.964  0.00  0.00      PROA
ATOM   1269  CB  TYR P  81       8.504 -24.484 -26.134  0.00  0.00      PROA
ATOM   1270  HB1 TYR P  81       7.516 -24.758 -26.562  0.00  0.00      PROA
ATOM   1271  HB2 TYR P  81       8.497 -23.944 -25.163  0.00  0.00      PROA
ATOM   1272  CG  TYR P  81       9.288 -25.799 -25.943  0.00  0.00      PROA
ATOM   1273  CD1 TYR P  81      10.121 -25.782 -24.842  0.00  0.00      PROA
ATOM   1274  HD1 TYR P  81      10.180 -24.902 -24.220  0.00  0.00      PROA
ATOM   1275  CE1 TYR P  81      10.955 -26.953 -24.612  0.00  0.00      PROA
ATOM   1276  HE1 TYR P  81      11.536 -26.954 -23.701  0.00  0.00      PROA
ATOM   1277  CZ  TYR P  81      10.852 -28.035 -25.440  0.00  0.00      PROA
ATOM   1278  OH  TYR P  81      11.726 -29.088 -25.170  0.00  0.00      PROA
ATOM   1279  HH  TYR P  81      11.373 -29.737 -25.784  0.00  0.00      PROA
ATOM   1280  CD2 TYR P  81       9.280 -26.823 -26.873  0.00  0.00      PROA
ATOM   1281  HD2 TYR P  81       8.704 -26.544 -27.743  0.00  0.00      PROA
ATOM   1282  CE2 TYR P  81      10.069 -27.991 -26.634  0.00  0.00      PROA
ATOM   1283  HE2 TYR P  81      10.166 -28.794 -27.350  0.00  0.00      PROA
ATOM   1284  C   TYR P  81       8.442 -22.727 -27.665  0.00  0.00      PROA
ATOM   1285  O   TYR P  81       7.732 -22.132 -26.882  0.00  0.00      PROA
ATOM   1286  N   GLU P  82       8.279 -22.682 -29.009  0.00  0.00      PROA
ATOM   1287  HN  GLU P  82       8.857 -23.239 -29.600  0.00  0.00      PROA
ATOM   1288  CA  GLU P  82       7.192 -22.030 -29.650  0.00  0.00      PROA
ATOM   1289  HA  GLU P  82       7.078 -21.040 -29.233  0.00  0.00      PROA
ATOM   1290  CB  GLU P  82       7.360 -21.805 -31.163  0.00  0.00      PROA
ATOM   1291  HB1 GLU P  82       6.350 -21.720 -31.619  0.00  0.00      PROA
ATOM   1292  HB2 GLU P  82       7.746 -22.724 -31.653  0.00  0.00      PROA
ATOM   1293  CG  GLU P  82       8.179 -20.504 -31.542  0.00  0.00      PROA
ATOM   1294  HG1 GLU P  82       9.178 -20.667 -31.083  0.00  0.00      PROA
ATOM   1295  HG2 GLU P  82       7.748 -19.570 -31.123  0.00  0.00      PROA
ATOM   1296  CD  GLU P  82       8.306 -20.335 -33.010  0.00  0.00      PROA
ATOM   1297  OE1 GLU P  82       9.424 -20.108 -33.521  0.00  0.00      PROA
ATOM   1298  OE2 GLU P  82       7.251 -20.383 -33.734  0.00  0.00      PROA
ATOM   1299  C   GLU P  82       5.896 -22.729 -29.370  0.00  0.00      PROA
ATOM   1300  O   GLU P  82       5.825 -23.983 -29.423  0.00  0.00      PROA
ATOM   1301  N   ALA P  83       4.807 -22.051 -28.984  0.00  0.00      PROA
ATOM   1302  HN  ALA P  83       4.817 -21.054 -29.016  0.00  0.00      PROA
ATOM   1303  CA  ALA P  83       3.647 -22.671 -28.420  0.00  0.00      PROA
ATOM   1304  HA  ALA P  83       3.751 -23.741 -28.523  0.00  0.00      PROA
ATOM   1305  CB  ALA P  83       3.591 -22.366 -26.906  0.00  0.00      PROA
ATOM   1306  HB1 ALA P  83       2.711 -22.812 -26.395  0.00  0.00      PROA
ATOM   1307  HB2 ALA P  83       3.642 -21.271 -26.726  0.00  0.00      PROA
ATOM   1308  HB3 ALA P  83       4.547 -22.711 -26.458  0.00  0.00      PROA
ATOM   1309  C   ALA P  83       2.287 -22.288 -28.931  0.00  0.00      PROA
ATOM   1310  O   ALA P  83       2.098 -21.277 -29.568  0.00  0.00      PROA
ATOM   1311  N   LYS P  84       1.211 -23.066 -28.550  0.00  0.00      PROA
ATOM   1312  HN  LYS P  84       1.343 -23.724 -27.813  0.00  0.00      PROA
ATOM   1313  CA  LYS P  84      -0.183 -22.725 -28.810  0.00  0.00      PROA
ATOM   1314  HA  LYS P  84      -0.293 -22.217 -29.757  0.00  0.00      PROA
```

```
ATOM   1315  CB  LYS P  84      -0.942 -24.004 -29.252  0.00  0.00      PROA
ATOM   1316  HB1 LYS P  84      -0.608 -24.721 -28.473  0.00  0.00      PROA
ATOM   1317  HB2 LYS P  84      -0.563 -24.345 -30.239  0.00  0.00      PROA
ATOM   1318  CG  LYS P  84      -2.471 -23.992 -29.451  0.00  0.00      PROA
ATOM   1319  HG1 LYS P  84      -2.850 -23.687 -28.452  0.00  0.00      PROA
ATOM   1320  HG2 LYS P  84      -2.959 -24.964 -29.678  0.00  0.00      PROA
ATOM   1321  CD  LYS P  84      -2.871 -22.992 -30.529  0.00  0.00      PROA
ATOM   1322  HD1 LYS P  84      -2.282 -23.135 -31.460  0.00  0.00      PROA
ATOM   1323  HD2 LYS P  84      -2.782 -21.941 -30.182  0.00  0.00      PROA
ATOM   1324  CE  LYS P  84      -4.332 -23.289 -31.090  0.00  0.00      PROA
ATOM   1325  HE1 LYS P  84      -4.469 -24.216 -31.688  0.00  0.00      PROA
ATOM   1326  HE2 LYS P  84      -4.524 -22.514 -31.862  0.00  0.00      PROA
ATOM   1327  NZ  LYS P  84      -5.296 -23.206 -30.013  0.00  0.00      PROA
ATOM   1328  HZ1 LYS P  84      -4.968 -22.708 -29.161  0.00  0.00      PROA
ATOM   1329  HZ2 LYS P  84      -5.456 -24.207 -29.779  0.00  0.00      PROA
ATOM   1330  HZ3 LYS P  84      -6.130 -22.677 -30.338  0.00  0.00      PROA
ATOM   1331  C   LYS P  84      -0.889 -22.139 -27.566  0.00  0.00      PROA
ATOM   1332  O   LYS P  84      -0.786 -22.699 -26.441  0.00  0.00      PROA
ATOM   1333  N   ILE P  85      -1.599 -21.001 -27.760  0.00  0.00      PROA
ATOM   1334  HN  ILE P  85      -1.462 -20.521 -28.623  0.00  0.00      PROA
ATOM   1335  CA  ILE P  85      -2.663 -20.470 -26.895  0.00  0.00      PROA
ATOM   1336  HA  ILE P  85      -2.306 -20.414 -25.878  0.00  0.00      PROA
ATOM   1337  CB  ILE P  85      -3.159 -19.045 -27.312  0.00  0.00      PROA
ATOM   1338  HB  ILE P  85      -3.732 -19.136 -28.260  0.00  0.00      PROA
ATOM   1339  CG2 ILE P  85      -4.039 -18.485 -26.194  0.00  0.00      PROA
ATOM   1340 HG21 ILE P  85      -5.037 -18.954 -26.057  0.00  0.00      PROA
ATOM   1341 HG22 ILE P  85      -4.209 -17.387 -26.227  0.00  0.00      PROA
ATOM   1342 HG23 ILE P  85      -3.527 -18.632 -25.219  0.00  0.00      PROA
ATOM   1343  CG1 ILE P  85      -1.951 -18.083 -27.414  0.00  0.00      PROA
ATOM   1344 HG11 ILE P  85      -1.099 -18.491 -27.998  0.00  0.00      PROA
ATOM   1345 HG12 ILE P  85      -1.548 -18.012 -26.381  0.00  0.00      PROA
ATOM   1346  CD  ILE P  85      -2.226 -16.662 -28.016  0.00  0.00      PROA
ATOM   1347  HD1 ILE P  85      -2.876 -16.709 -28.916  0.00  0.00      PROA
ATOM   1348  HD2 ILE P  85      -1.297 -16.175 -28.383  0.00  0.00      PROA
ATOM   1349  HD3 ILE P  85      -2.699 -16.000 -27.260  0.00  0.00      PROA
ATOM   1350  C   ILE P  85      -3.888 -21.394 -26.842  0.00  0.00      PROA
ATOM   1351  O   ILE P  85      -4.374 -21.797 -27.856  0.00  0.00      PROA
ATOM   1352  N   LYS P  86      -4.357 -21.612 -25.605  0.00  0.00      PROA
ATOM   1353  HN  LYS P  86      -3.818 -21.214 -24.868  0.00  0.00      PROA
ATOM   1354  CA  LYS P  86      -5.446 -22.516 -25.281  0.00  0.00      PROA
ATOM   1355  HA  LYS P  86      -5.755 -22.993 -26.199  0.00  0.00      PROA
ATOM   1356  CB  LYS P  86      -4.964 -23.544 -24.120  0.00  0.00      PROA
ATOM   1357  HB1 LYS P  86      -5.806 -24.200 -23.810  0.00  0.00      PROA
ATOM   1358  HB2 LYS P  86      -4.819 -22.855 -23.260  0.00  0.00      PROA
ATOM   1359  CG  LYS P  86      -3.728 -24.428 -24.358  0.00  0.00      PROA
ATOM   1360  HG1 LYS P  86      -3.673 -24.986 -23.399  0.00  0.00      PROA
ATOM   1361  HG2 LYS P  86      -2.850 -23.795 -24.608  0.00  0.00      PROA
ATOM   1362  CD  LYS P  86      -4.048 -25.398 -25.509  0.00  0.00      PROA
ATOM   1363  HD1 LYS P  86      -3.144 -25.878 -25.938  0.00  0.00      PROA
ATOM   1364  HD2 LYS P  86      -4.410 -24.863 -26.413  0.00  0.00      PROA
ATOM   1365  CE  LYS P  86      -4.934 -26.617 -25.062  0.00  0.00      PROA
ATOM   1366  HE1 LYS P  86      -5.952 -26.286 -24.763  0.00  0.00      PROA
ATOM   1367  HE2 LYS P  86      -4.527 -27.114 -24.156  0.00  0.00      PROA
ATOM   1368  NZ  LYS P  86      -5.331 -27.585 -26.033  0.00  0.00      PROA
ATOM   1369  HZ1 LYS P  86      -4.551 -28.082 -26.509  0.00  0.00      PROA
ATOM   1370  HZ2 LYS P  86      -5.882 -27.210 -26.831  0.00  0.00      PROA
ATOM   1371  HZ3 LYS P  86      -5.908 -28.280 -25.518  0.00  0.00      PROA
ATOM   1372  C   LYS P  86      -6.526 -21.562 -24.748  0.00  0.00      PROA
ATOM   1373  O   LYS P  86      -6.609 -20.429 -25.324  0.00  0.00      PROA
ATOM   1374  N   ASP P  87      -7.362 -21.949 -23.692  0.00  0.00      PROA
ATOM   1375  HN  ASP P  87      -7.146 -22.896 -23.466  0.00  0.00      PROA
ATOM   1376  CA  ASP P  87      -8.409 -21.144 -23.117  0.00  0.00      PROA
ATOM   1377  HA  ASP P  87      -9.154 -20.972 -23.880  0.00  0.00      PROA
ATOM   1378  CB  ASP P  87      -9.420 -21.910 -22.222  0.00  0.00      PROA
ATOM   1379  HB1 ASP P  87     -10.294 -21.334 -21.849  0.00  0.00      PROA
ATOM   1380  HB2 ASP P  87      -8.822 -22.359 -21.400  0.00  0.00      PROA
ATOM   1381  CG  ASP P  87      -9.917 -23.106 -23.024  0.00  0.00      PROA
ATOM   1382  OD1 ASP P  87     -10.147 -24.159 -22.441  0.00  0.00      PROA
ATOM   1383  OD2 ASP P  87     -10.091 -22.973 -24.261  0.00  0.00      PROA
ATOM   1384  C   ASP P  87      -7.946 -19.842 -22.441  0.00  0.00      PROA
ATOM   1385  O   ASP P  87      -6.848 -19.896 -21.928  0.00  0.00      PROA
ATOM   1386  N   VAL P  88      -8.749 -18.807 -22.419  0.00  0.00      PROA
ATOM   1387  HN  VAL P  88      -9.619 -18.985 -22.872  0.00  0.00      PROA
```

| | | | | | | | | | | | |
|---|---|---|---|---|---|---|---|---|---|---|---|
| ATOM | 1388 | CA | VAL | P | 88 | -8.486 | -17.461 | -21.883 | 0.00 | 0.00 | PROA |
| ATOM | 1389 | HA | VAL | P | 88 | -7.598 | -17.424 | -21.268 | 0.00 | 0.00 | PROA |
| ATOM | 1390 | CB | VAL | P | 88 | -8.295 | -16.410 | -23.033 | 0.00 | 0.00 | PROA |
| ATOM | 1391 | HB | VAL | P | 88 | -9.270 | -16.127 | -23.484 | 0.00 | 0.00 | PROA |
| ATOM | 1392 | CG1 | VAL | P | 88 | -7.746 | -15.029 | -22.481 | 0.00 | 0.00 | PROA |
| ATOM | 1393 | HG11 | VAL | P | 88 | -8.618 | -14.402 | -22.197 | 0.00 | 0.00 | PROA |
| ATOM | 1394 | HG12 | VAL | P | 88 | -7.123 | -14.498 | -23.232 | 0.00 | 0.00 | PROA |
| ATOM | 1395 | HG13 | VAL | P | 88 | -7.152 | -15.094 | -21.544 | 0.00 | 0.00 | PROA |
| ATOM | 1396 | CG2 | VAL | P | 88 | -7.201 | -16.926 | -23.944 | 0.00 | 0.00 | PROA |
| ATOM | 1397 | HG21 | VAL | P | 88 | -7.477 | -17.831 | -24.527 | 0.00 | 0.00 | PROA |
| ATOM | 1398 | HG22 | VAL | P | 88 | -6.321 | -17.132 | -23.298 | 0.00 | 0.00 | PROA |
| ATOM | 1399 | HG23 | VAL | P | 88 | -6.833 | -16.238 | -24.735 | 0.00 | 0.00 | PROA |
| ATOM | 1400 | C | VAL | P | 88 | -9.763 | -17.062 | -21.139 | 0.00 | 0.00 | PROA |
| ATOM | 1401 | O | VAL | P | 88 | -10.904 | -17.332 | -21.606 | 0.00 | 0.00 | PROA |
| ATOM | 1402 | N | ASP | P | 89 | -9.574 | -16.327 | -20.023 | 0.00 | 0.00 | PROA |
| ATOM | 1403 | HN | ASP | P | 89 | -8.732 | -16.315 | -19.489 | 0.00 | 0.00 | PROA |
| ATOM | 1404 | CA | ASP | P | 89 | -10.755 | -15.865 | -19.264 | 0.00 | 0.00 | PROA |
| ATOM | 1405 | HA | ASP | P | 89 | -11.489 | -15.645 | -20.026 | 0.00 | 0.00 | PROA |
| ATOM | 1406 | CB | ASP | P | 89 | -11.232 | -16.854 | -18.109 | 0.00 | 0.00 | PROA |
| ATOM | 1407 | HB1 | ASP | P | 89 | -10.569 | -16.805 | -17.219 | 0.00 | 0.00 | PROA |
| ATOM | 1408 | HB2 | ASP | P | 89 | -11.168 | -17.910 | -18.449 | 0.00 | 0.00 | PROA |
| ATOM | 1409 | CG | ASP | P | 89 | -12.656 | -16.448 | -17.673 | 0.00 | 0.00 | PROA |
| ATOM | 1410 | OD1 | ASP | P | 89 | -12.948 | -16.581 | -16.481 | 0.00 | 0.00 | PROA |
| ATOM | 1411 | OD2 | ASP | P | 89 | -13.484 | -16.020 | -18.520 | 0.00 | 0.00 | PROA |
| ATOM | 1412 | C | ASP | P | 89 | -10.408 | -14.541 | -18.894 | 0.00 | 0.00 | PROA |
| ATOM | 1413 | O | ASP | P | 89 | -9.682 | -14.333 | -17.904 | 0.00 | 0.00 | PROA |
| ATOM | 1414 | N | GLU | P | 90 | -10.833 | -13.450 | -19.594 | 0.00 | 0.00 | PROA |
| ATOM | 1415 | HN | GLU | P | 90 | -11.424 | -13.589 | -20.385 | 0.00 | 0.00 | PROA |
| ATOM | 1416 | CA | GLU | P | 90 | -10.527 | -12.091 | -19.153 | 0.00 | 0.00 | PROA |
| ATOM | 1417 | HA | GLU | P | 90 | -9.448 | -12.067 | -19.180 | 0.00 | 0.00 | PROA |
| ATOM | 1418 | CB | GLU | P | 90 | -10.954 | -11.129 | -20.247 | 0.00 | 0.00 | PROA |
| ATOM | 1419 | HB1 | GLU | P | 90 | -10.929 | -10.053 | -19.972 | 0.00 | 0.00 | PROA |
| ATOM | 1420 | HB2 | GLU | P | 90 | -11.960 | -11.511 | -20.521 | 0.00 | 0.00 | PROA |
| ATOM | 1421 | CG | GLU | P | 90 | -9.931 | -11.233 | -21.441 | 0.00 | 0.00 | PROA |
| ATOM | 1422 | HG1 | GLU | P | 90 | -10.363 | -12.062 | -22.041 | 0.00 | 0.00 | PROA |
| ATOM | 1423 | HG2 | GLU | P | 90 | -8.891 | -11.509 | -21.164 | 0.00 | 0.00 | PROA |
| ATOM | 1424 | CD | GLU | P | 90 | -9.725 | -9.882 | -22.194 | 0.00 | 0.00 | PROA |
| ATOM | 1425 | OE1 | GLU | P | 90 | -8.600 | -9.347 | -22.299 | 0.00 | 0.00 | PROA |
| ATOM | 1426 | OE2 | GLU | P | 90 | -10.718 | -9.417 | -22.821 | 0.00 | 0.00 | PROA |
| ATOM | 1427 | C | GLU | P | 90 | -11.139 | -11.615 | -17.883 | 0.00 | 0.00 | PROA |
| ATOM | 1428 | O | GLU | P | 90 | -10.513 | -10.836 | -17.241 | 0.00 | 0.00 | PROA |
| ATOM | 1429 | N | LYS | P | 91 | -12.303 | -12.113 | -17.443 | 0.00 | 0.00 | PROA |
| ATOM | 1430 | HN | LYS | P | 91 | -12.859 | -12.669 | -18.056 | 0.00 | 0.00 | PROA |
| ATOM | 1431 | CA | LYS | P | 91 | -12.854 | -11.698 | -16.207 | 0.00 | 0.00 | PROA |
| ATOM | 1432 | HA | LYS | P | 91 | -12.771 | -10.622 | -16.169 | 0.00 | 0.00 | PROA |
| ATOM | 1433 | CB | LYS | P | 91 | -14.336 | -12.138 | -16.112 | 0.00 | 0.00 | PROA |
| ATOM | 1434 | HB1 | LYS | P | 91 | -14.927 | -11.521 | -16.823 | 0.00 | 0.00 | PROA |
| ATOM | 1435 | HB2 | LYS | P | 91 | -14.700 | -11.910 | -15.088 | 0.00 | 0.00 | PROA |
| ATOM | 1436 | CG | LYS | P | 91 | -14.592 | -13.654 | -16.295 | 0.00 | 0.00 | PROA |
| ATOM | 1437 | HG1 | LYS | P | 91 | -14.004 | -14.284 | -15.593 | 0.00 | 0.00 | PROA |
| ATOM | 1438 | HG2 | LYS | P | 91 | -14.188 | -13.820 | -17.316 | 0.00 | 0.00 | PROA |
| ATOM | 1439 | CD | LYS | P | 91 | -16.085 | -13.989 | -16.302 | 0.00 | 0.00 | PROA |
| ATOM | 1440 | HD1 | LYS | P | 91 | -16.650 | -13.310 | -16.976 | 0.00 | 0.00 | PROA |
| ATOM | 1441 | HD2 | LYS | P | 91 | -16.622 | -13.843 | -15.340 | 0.00 | 0.00 | PROA |
| ATOM | 1442 | CE | LYS | P | 91 | -16.345 | -15.388 | -16.895 | 0.00 | 0.00 | PROA |
| ATOM | 1443 | HE1 | LYS | P | 91 | -15.903 | -15.448 | -17.913 | 0.00 | 0.00 | PROA |
| ATOM | 1444 | HE2 | LYS | P | 91 | -17.427 | -15.639 | -16.910 | 0.00 | 0.00 | PROA |
| ATOM | 1445 | NZ | LYS | P | 91 | -15.632 | -16.299 | -16.000 | 0.00 | 0.00 | PROA |
| ATOM | 1446 | HZ1 | LYS | P | 91 | -15.942 | -17.279 | -16.159 | 0.00 | 0.00 | PROA |
| ATOM | 1447 | HZ2 | LYS | P | 91 | -15.743 | -16.044 | -14.998 | 0.00 | 0.00 | PROA |
| ATOM | 1448 | HZ3 | LYS | P | 91 | -14.622 | -16.355 | -16.240 | 0.00 | 0.00 | PROA |
| ATOM | 1449 | C | LYS | P | 91 | -12.145 | -12.313 | -14.951 | 0.00 | 0.00 | PROA |
| ATOM | 1450 | O | LYS | P | 91 | -12.330 | -11.821 | -13.863 | 0.00 | 0.00 | PROA |
| ATOM | 1451 | N | ALA | P | 92 | -11.312 | -13.398 | -15.086 | 0.00 | 0.00 | PROA |
| ATOM | 1452 | HN | ALA | P | 92 | -11.167 | -13.904 | -15.932 | 0.00 | 0.00 | PROA |
| ATOM | 1453 | CA | ALA | P | 92 | -10.593 | -13.954 | -13.972 | 0.00 | 0.00 | PROA |
| ATOM | 1454 | HA | ALA | P | 92 | -10.898 | -13.458 | -13.063 | 0.00 | 0.00 | PROA |
| ATOM | 1455 | CB | ALA | P | 92 | -11.047 | -15.379 | -13.885 | 0.00 | 0.00 | PROA |
| ATOM | 1456 | HB1 | ALA | P | 92 | -12.150 | -15.400 | -14.018 | 0.00 | 0.00 | PROA |
| ATOM | 1457 | HB2 | ALA | P | 92 | -10.862 | -15.757 | -12.857 | 0.00 | 0.00 | PROA |
| ATOM | 1458 | HB3 | ALA | P | 92 | -10.521 | -16.086 | -14.561 | 0.00 | 0.00 | PROA |
| ATOM | 1459 | C | ALA | P | 92 | -9.105 | -13.827 | -14.116 | 0.00 | 0.00 | PROA |
| ATOM | 1460 | O | ALA | P | 92 | -8.329 | -14.520 | -13.419 | 0.00 | 0.00 | PROA |

```
ATOM   1461  N   ASP P  93      -8.641 -12.994 -15.064  0.00  0.00      PROA
ATOM   1462  HN  ASP P  93      -9.292 -12.647 -15.735  0.00  0.00      PROA
ATOM   1463  CA  ASP P  93      -7.248 -12.743 -15.417  0.00  0.00      PROA
ATOM   1464  HA  ASP P  93      -7.381 -12.381 -16.426  0.00  0.00      PROA
ATOM   1465  CB  ASP P  93      -6.696 -11.454 -14.744  0.00  0.00      PROA
ATOM   1466  HB1 ASP P  93      -6.204 -11.630 -13.764  0.00  0.00      PROA
ATOM   1467  HB2 ASP P  93      -7.633 -10.874 -14.600  0.00  0.00      PROA
ATOM   1468  CG  ASP P  93      -5.593 -10.702 -15.451  0.00  0.00      PROA
ATOM   1469  OD1 ASP P  93      -5.485 -10.748 -16.735  0.00  0.00      PROA
ATOM   1470  OD2 ASP P  93      -4.800 -10.004 -14.768  0.00  0.00      PROA
ATOM   1471  C   ASP P  93      -6.318 -13.873 -15.552  0.00  0.00      PROA
ATOM   1472  O   ASP P  93      -5.183 -13.886 -15.134  0.00  0.00      PROA
ATOM   1473  N   ILE P  94      -6.778 -14.985 -16.259  0.00  0.00      PROA
ATOM   1474  HN  ILE P  94      -7.730 -14.960 -16.556  0.00  0.00      PROA
ATOM   1475  CA  ILE P  94      -6.138 -16.336 -16.362  0.00  0.00      PROA
ATOM   1476  HA  ILE P  94      -5.122 -16.239 -16.008  0.00  0.00      PROA
ATOM   1477  CB  ILE P  94      -6.731 -17.379 -15.486  0.00  0.00      PROA
ATOM   1478  HB  ILE P  94      -6.738 -16.941 -14.465  0.00  0.00      PROA
ATOM   1479  CG2 ILE P  94      -8.263 -17.759 -15.761  0.00  0.00      PROA
ATOM   1480 HG21 ILE P  94      -8.869 -16.828 -15.776  0.00  0.00      PROA
ATOM   1481 HG22 ILE P  94      -8.759 -18.371 -14.978  0.00  0.00      PROA
ATOM   1482 HG23 ILE P  94      -8.469 -18.219 -16.752  0.00  0.00      PROA
ATOM   1483  CG1 ILE P  94      -5.883 -18.616 -15.383  0.00  0.00      PROA
ATOM   1484 HG11 ILE P  94      -4.835 -18.284 -15.224  0.00  0.00      PROA
ATOM   1485 HG12 ILE P  94      -5.817 -19.217 -16.315  0.00  0.00      PROA
ATOM   1486  CD  ILE P  94      -6.199 -19.499 -14.119  0.00  0.00      PROA
ATOM   1487  HD1 ILE P  94      -5.363 -20.202 -13.912  0.00  0.00      PROA
ATOM   1488  HD2 ILE P  94      -7.064 -20.156 -14.351  0.00  0.00      PROA
ATOM   1489  HD3 ILE P  94      -6.508 -18.882 -13.248  0.00  0.00      PROA
ATOM   1490  C   ILE P  94      -6.118 -16.755 -17.846  0.00  0.00      PROA
ATOM   1491  O   ILE P  94      -7.156 -16.773 -18.495  0.00  0.00      PROA
ATOM   1492  N   ALA P  95      -4.899 -17.217 -18.319  0.00  0.00      PROA
ATOM   1493  HN  ALA P  95      -4.016 -17.320 -17.867  0.00  0.00      PROA
ATOM   1494  CA  ALA P  95      -4.940 -17.844 -19.626  0.00  0.00      PROA
ATOM   1495  HA  ALA P  95      -5.946 -18.164 -19.854  0.00  0.00      PROA
ATOM   1496  CB  ALA P  95      -4.469 -16.901 -20.768  0.00  0.00      PROA
ATOM   1497  HB1 ALA P  95      -5.134 -16.013 -20.708  0.00  0.00      PROA
ATOM   1498  HB2 ALA P  95      -4.431 -17.352 -21.783  0.00  0.00      PROA
ATOM   1499  HB3 ALA P  95      -3.442 -16.557 -20.519  0.00  0.00      PROA
ATOM   1500  C   ALA P  95      -3.979 -19.080 -19.658  0.00  0.00      PROA
ATOM   1501  O   ALA P  95      -3.103 -19.202 -18.841  0.00  0.00      PROA
ATOM   1502  N   LEU P  96      -4.153 -19.960 -20.674  0.00  0.00      PROA
ATOM   1503  HN  LEU P  96      -4.874 -19.756 -21.333  0.00  0.00      PROA
ATOM   1504  CA  LEU P  96      -3.531 -21.248 -20.798  0.00  0.00      PROA
ATOM   1505  HA  LEU P  96      -2.767 -21.350 -20.042  0.00  0.00      PROA
ATOM   1506  CB  LEU P  96      -4.765 -22.196 -20.624  0.00  0.00      PROA
ATOM   1507  HB1 LEU P  96      -5.455 -22.062 -21.485  0.00  0.00      PROA
ATOM   1508  HB2 LEU P  96      -5.326 -21.751 -19.774  0.00  0.00      PROA
ATOM   1509  CG  LEU P  96      -4.259 -23.614 -20.406  0.00  0.00      PROA
ATOM   1510  HG  LEU P  96      -3.513 -23.900 -21.178  0.00  0.00      PROA
ATOM   1511  CD1 LEU P  96      -3.393 -23.691 -19.080  0.00  0.00      PROA
ATOM   1512 HD11 LEU P  96      -2.330 -23.441 -19.284  0.00  0.00      PROA
ATOM   1513 HD12 LEU P  96      -3.400 -24.732 -18.691  0.00  0.00      PROA
ATOM   1514 HD13 LEU P  96      -3.762 -23.006 -18.287  0.00  0.00      PROA
ATOM   1515  CD2 LEU P  96      -5.345 -24.669 -20.183  0.00  0.00      PROA
ATOM   1516 HD21 LEU P  96      -4.870 -25.672 -20.130  0.00  0.00      PROA
ATOM   1517 HD22 LEU P  96      -6.058 -24.641 -21.035  0.00  0.00      PROA
ATOM   1518 HD23 LEU P  96      -5.969 -24.459 -19.288  0.00  0.00      PROA
ATOM   1519  C   LEU P  96      -2.843 -21.299 -22.122  0.00  0.00      PROA
ATOM   1520  O   LEU P  96      -3.385 -20.781 -23.076  0.00  0.00      PROA
ATOM   1521  N   ILE P  97      -1.650 -21.805 -22.152  0.00  0.00      PROA
ATOM   1522  HN  ILE P  97      -1.269 -22.067 -21.269  0.00  0.00      PROA
ATOM   1523  CA  ILE P  97      -0.858 -22.017 -23.335  0.00  0.00      PROA
ATOM   1524  HA  ILE P  97      -1.373 -21.711 -24.233  0.00  0.00      PROA
ATOM   1525  CB  ILE P  97       0.296 -20.959 -23.311  0.00  0.00      PROA
ATOM   1526  HB  ILE P  97       0.818 -20.919 -22.331  0.00  0.00      PROA
ATOM   1527  CG2 ILE P  97       1.408 -21.306 -24.378  0.00  0.00      PROA
ATOM   1528 HG21 ILE P  97       1.814 -22.331 -24.237  0.00  0.00      PROA
ATOM   1529 HG22 ILE P  97       2.309 -20.656 -24.376  0.00  0.00      PROA
ATOM   1530 HG23 ILE P  97       1.101 -21.197 -25.440  0.00  0.00      PROA
ATOM   1531  CG1 ILE P  97      -0.231 -19.506 -23.554  0.00  0.00      PROA
ATOM   1532 HG11 ILE P  97      -1.271 -19.312 -23.215  0.00  0.00      PROA
ATOM   1533 HG12 ILE P  97      -0.233 -19.460 -24.664  0.00  0.00      PROA
```

| | | | | | | | | | | | |
|---|---|---|---|---|---|---|---|---|---|---|---|
| ATOM | 1534 | CD | ILE | P | 97 | 0.586 | -18.367 | -22.973 | 0.00 | 0.00 | PROA |
| ATOM | 1535 | HD1 | ILE | P | 97 | 0.352 | -17.364 | -23.389 | 0.00 | 0.00 | PROA |
| ATOM | 1536 | HD2 | ILE | P | 97 | 1.667 | -18.450 | -23.217 | 0.00 | 0.00 | PROA |
| ATOM | 1537 | HD3 | ILE | P | 97 | 0.495 | -18.393 | -21.866 | 0.00 | 0.00 | PROA |
| ATOM | 1538 | C | ILE | P | 97 | -0.368 | -23.431 | -23.277 | 0.00 | 0.00 | PROA |
| ATOM | 1539 | O | ILE | P | 97 | -0.018 | -23.912 | -22.222 | 0.00 | 0.00 | PROA |
| ATOM | 1540 | N | LYS | P | 98 | -0.124 | -24.179 | -24.358 | 0.00 | 0.00 | PROA |
| ATOM | 1541 | HN | LYS | P | 98 | -0.101 | -23.685 | -25.224 | 0.00 | 0.00 | PROA |
| ATOM | 1542 | CA | LYS | P | 98 | 0.355 | -25.588 | -24.342 | 0.00 | 0.00 | PROA |
| ATOM | 1543 | HA | LYS | P | 98 | 0.532 | -26.026 | -23.371 | 0.00 | 0.00 | PROA |
| ATOM | 1544 | CB | LYS | P | 98 | -0.672 | -26.539 | -25.033 | 0.00 | 0.00 | PROA |
| ATOM | 1545 | HB1 | LYS | P | 98 | -0.876 | -26.077 | -26.022 | 0.00 | 0.00 | PROA |
| ATOM | 1546 | HB2 | LYS | P | 98 | -1.646 | -26.471 | -24.503 | 0.00 | 0.00 | PROA |
| ATOM | 1547 | CG | LYS | P | 98 | -0.035 | -27.903 | -25.278 | 0.00 | 0.00 | PROA |
| ATOM | 1548 | HG1 | LYS | P | 98 | 0.738 | -27.866 | -26.075 | 0.00 | 0.00 | PROA |
| ATOM | 1549 | HG2 | LYS | P | 98 | -0.890 | -28.371 | -25.812 | 0.00 | 0.00 | PROA |
| ATOM | 1550 | CD | LYS | P | 98 | 0.226 | -28.735 | -24.016 | 0.00 | 0.00 | PROA |
| ATOM | 1551 | HD1 | LYS | P | 98 | -0.715 | -28.596 | -23.442 | 0.00 | 0.00 | PROA |
| ATOM | 1552 | HD2 | LYS | P | 98 | 1.079 | -28.309 | -23.446 | 0.00 | 0.00 | PROA |
| ATOM | 1553 | CE | LYS | P | 98 | 0.350 | -30.181 | -24.189 | 0.00 | 0.00 | PROA |
| ATOM | 1554 | HE1 | LYS | P | 98 | -0.387 | -30.745 | -24.799 | 0.00 | 0.00 | PROA |
| ATOM | 1555 | HE2 | LYS | P | 98 | 0.265 | -30.674 | -23.197 | 0.00 | 0.00 | PROA |
| ATOM | 1556 | NZ | LYS | P | 98 | 1.611 | -30.588 | -24.791 | 0.00 | 0.00 | PROA |
| ATOM | 1557 | HZ1 | LYS | P | 98 | 1.665 | -30.386 | -25.810 | 0.00 | 0.00 | PROA |
| ATOM | 1558 | HZ2 | LYS | P | 98 | 1.788 | -31.611 | -24.725 | 0.00 | 0.00 | PROA |
| ATOM | 1559 | HZ3 | LYS | P | 98 | 2.443 | -30.204 | -24.298 | 0.00 | 0.00 | PROA |
| ATOM | 1560 | C | LYS | P | 98 | 1.758 | -25.548 | -24.957 | 0.00 | 0.00 | PROA |
| ATOM | 1561 | O | LYS | P | 98 | 1.845 | -25.114 | -26.043 | 0.00 | 0.00 | PROA |
| ATOM | 1562 | N | ILE | P | 99 | 2.843 | -26.067 | -24.325 | 0.00 | 0.00 | PROA |
| ATOM | 1563 | HN | ILE | P | 99 | 2.801 | -26.462 | -23.411 | 0.00 | 0.00 | PROA |
| ATOM | 1564 | CA | ILE | P | 99 | 4.102 | -26.469 | -25.061 | 0.00 | 0.00 | PROA |
| ATOM | 1565 | HA | ILE | P | 99 | 4.095 | -25.946 | -26.006 | 0.00 | 0.00 | PROA |
| ATOM | 1566 | CB | ILE | P | 99 | 5.323 | -26.020 | -24.280 | 0.00 | 0.00 | PROA |
| ATOM | 1567 | HB | ILE | P | 99 | 6.291 | -26.148 | -24.809 | 0.00 | 0.00 | PROA |
| ATOM | 1568 | CG2 | ILE | P | 99 | 5.322 | -24.398 | -24.354 | 0.00 | 0.00 | PROA |
| ATOM | 1569 | HG21 | ILE | P | 99 | 5.896 | -24.027 | -25.230 | 0.00 | 0.00 | PROA |
| ATOM | 1570 | HG22 | ILE | P | 99 | 5.897 | -24.148 | -23.437 | 0.00 | 0.00 | PROA |
| ATOM | 1571 | HG23 | ILE | P | 99 | 4.288 | -24.002 | -24.265 | 0.00 | 0.00 | PROA |
| ATOM | 1572 | CG1 | ILE | P | 99 | 5.491 | -26.490 | -22.827 | 0.00 | 0.00 | PROA |
| ATOM | 1573 | HG11 | ILE | P | 99 | 5.011 | -27.464 | -22.590 | 0.00 | 0.00 | PROA |
| ATOM | 1574 | HG12 | ILE | P | 99 | 5.025 | -25.786 | -22.105 | 0.00 | 0.00 | PROA |
| ATOM | 1575 | CD | ILE | P | 99 | 6.975 | -26.702 | -22.538 | 0.00 | 0.00 | PROA |
| ATOM | 1576 | HD1 | ILE | P | 99 | 7.220 | -27.003 | -21.497 | 0.00 | 0.00 | PROA |
| ATOM | 1577 | HD2 | ILE | P | 99 | 7.517 | -25.761 | -22.771 | 0.00 | 0.00 | PROA |
| ATOM | 1578 | HD3 | ILE | P | 99 | 7.367 | -27.537 | -23.158 | 0.00 | 0.00 | PROA |
| ATOM | 1579 | C | ILE | P | 99 | 4.178 | -27.941 | -25.192 | 0.00 | 0.00 | PROA |
| ATOM | 1580 | O | ILE | P | 99 | 3.492 | -28.706 | -24.576 | 0.00 | 0.00 | PROA |
| ATOM | 1581 | N | ASP | P | 100 | 5.053 | -28.513 | -26.054 | 0.00 | 0.00 | PROA |
| ATOM | 1582 | HN | ASP | P | 100 | 5.569 | -27.932 | -26.679 | 0.00 | 0.00 | PROA |
| ATOM | 1583 | CA | ASP | P | 100 | 5.107 | -29.869 | -26.448 | 0.00 | 0.00 | PROA |
| ATOM | 1584 | HA | ASP | P | 100 | 4.514 | -30.411 | -25.726 | 0.00 | 0.00 | PROA |
| ATOM | 1585 | CB | ASP | P | 100 | 4.724 | -30.239 | -27.918 | 0.00 | 0.00 | PROA |
| ATOM | 1586 | HB1 | ASP | P | 100 | 4.733 | -31.295 | -28.264 | 0.00 | 0.00 | PROA |
| ATOM | 1587 | HB2 | ASP | P | 100 | 5.322 | -29.640 | -28.638 | 0.00 | 0.00 | PROA |
| ATOM | 1588 | CG | ASP | P | 100 | 3.269 | -29.862 | -28.073 | 0.00 | 0.00 | PROA |
| ATOM | 1589 | OD1 | ASP | P | 100 | 2.390 | -30.545 | -27.447 | 0.00 | 0.00 | PROA |
| ATOM | 1590 | OD2 | ASP | P | 100 | 3.030 | -29.057 | -29.033 | 0.00 | 0.00 | PROA |
| ATOM | 1591 | C | ASP | P | 100 | 6.456 | -30.236 | -26.192 | 0.00 | 0.00 | PROA |
| ATOM | 1592 | O | ASP | P | 100 | 7.345 | -29.559 | -26.668 | 0.00 | 0.00 | PROA |
| ATOM | 1593 | N | HSD | P | 101 | 6.683 | -31.301 | -25.385 | 0.00 | 0.00 | PROA |
| ATOM | 1594 | HN | HSD | P | 101 | 5.956 | -31.899 | -25.056 | 0.00 | 0.00 | PROA |
| ATOM | 1595 | CA | HSD | P | 101 | 7.975 | -31.639 | -24.706 | 0.00 | 0.00 | PROA |
| ATOM | 1596 | HA | HSD | P | 101 | 8.714 | -31.250 | -25.391 | 0.00 | 0.00 | PROA |
| ATOM | 1597 | CB | HSD | P | 101 | 8.099 | -30.859 | -23.399 | 0.00 | 0.00 | PROA |
| ATOM | 1598 | HB1 | HSD | P | 101 | 7.316 | -31.114 | -22.653 | 0.00 | 0.00 | PROA |
| ATOM | 1599 | HB2 | HSD | P | 101 | 7.602 | -29.891 | -23.625 | 0.00 | 0.00 | PROA |
| ATOM | 1600 | ND1 | HSD | P | 101 | 10.340 | -31.639 | -22.361 | 0.00 | 0.00 | PROA |
| ATOM | 1601 | HD1 | HSD | P | 101 | 10.092 | -32.607 | -22.393 | 0.00 | 0.00 | PROA |
| ATOM | 1602 | CG | HSD | P | 101 | 9.482 | -30.724 | -22.827 | 0.00 | 0.00 | PROA |
| ATOM | 1603 | CE1 | HSD | P | 101 | 11.361 | -30.972 | -21.810 | 0.00 | 0.00 | PROA |
| ATOM | 1604 | HE1 | HSD | P | 101 | 12.150 | -31.513 | -21.288 | 0.00 | 0.00 | PROA |
| ATOM | 1605 | NE2 | HSD | P | 101 | 11.249 | -29.701 | -21.823 | 0.00 | 0.00 | PROA |
| ATOM | 1606 | CD2 | HSD | P | 101 | 10.082 | -29.543 | -22.521 | 0.00 | 0.00 | PROA |

```
ATOM   1607  HD2 HSD P 101       9.627 -28.570 -22.658  0.00  0.00      PROA
ATOM   1608  C   HSD P 101       8.123 -33.093 -24.563  0.00  0.00      PROA
ATOM   1609  O   HSD P 101       7.260 -33.860 -24.067  0.00  0.00      PROA
ATOM   1610  N   GLN P 102       9.293 -33.606 -24.951  0.00  0.00      PROA
ATOM   1611  HN  GLN P 102       9.941 -33.108 -25.522  0.00  0.00      PROA
ATOM   1612  CA  GLN P 102       9.568 -34.999 -24.751  0.00  0.00      PROA
ATOM   1613  HA  GLN P 102       8.680 -35.501 -25.107  0.00  0.00      PROA
ATOM   1614  CB  GLN P 102      10.863 -35.429 -25.565  0.00  0.00      PROA
ATOM   1615  HB1 GLN P 102      11.014 -36.501 -25.314  0.00  0.00      PROA
ATOM   1616  HB2 GLN P 102      11.756 -34.848 -25.249  0.00  0.00      PROA
ATOM   1617  CG  GLN P 102      10.837 -35.202 -27.045  0.00  0.00      PROA
ATOM   1618  HG1 GLN P 102      11.639 -35.811 -27.514  0.00  0.00      PROA
ATOM   1619  HG2 GLN P 102      10.972 -34.152 -27.384  0.00  0.00      PROA
ATOM   1620  CD  GLN P 102       9.510 -35.577 -27.798  0.00  0.00      PROA
ATOM   1621  OE1 GLN P 102       8.684 -34.697 -28.128  0.00  0.00      PROA
ATOM   1622  NE2 GLN P 102       9.336 -36.838 -28.151  0.00  0.00      PROA
ATOM   1623 HE21 GLN P 102       8.476 -37.013 -28.630  0.00  0.00      PROA
ATOM   1624 HE22 GLN P 102      10.023 -37.531 -27.932  0.00  0.00      PROA
ATOM   1625  C   GLN P 102       9.633 -35.306 -23.207  0.00  0.00      PROA
ATOM   1626  O   GLN P 102      10.148 -34.523 -22.445  0.00  0.00      PROA
ATOM   1627  N   GLY P 103       8.992 -36.448 -22.785  0.00  0.00      PROA
ATOM   1628  HN  GLY P 103       8.633 -37.108 -23.440  0.00  0.00      PROA
ATOM   1629  CA  GLY P 103       8.770 -36.738 -21.398  0.00  0.00      PROA
ATOM   1630  HA1 GLY P 103       9.681 -36.836 -20.827  0.00  0.00      PROA
ATOM   1631  HA2 GLY P 103       8.085 -37.558 -21.242  0.00  0.00      PROA
ATOM   1632  C   GLY P 103       8.116 -35.749 -20.451  0.00  0.00      PROA
ATOM   1633  O   GLY P 103       8.090 -34.520 -20.653  0.00  0.00      PROA
ATOM   1634  N   LYS P 104       7.529 -36.238 -19.293  0.00  0.00      PROA
ATOM   1635  HN  LYS P 104       7.611 -37.194 -19.021  0.00  0.00      PROA
ATOM   1636  CA  LYS P 104       6.912 -35.417 -18.342  0.00  0.00      PROA
ATOM   1637  HA  LYS P 104       6.116 -34.943 -18.897  0.00  0.00      PROA
ATOM   1638  CB  LYS P 104       6.334 -36.251 -17.055  0.00  0.00      PROA
ATOM   1639  HB1 LYS P 104       5.837 -35.540 -16.361  0.00  0.00      PROA
ATOM   1640  HB2 LYS P 104       7.120 -36.873 -16.576  0.00  0.00      PROA
ATOM   1641  CG  LYS P 104       5.385 -37.413 -17.495  0.00  0.00      PROA
ATOM   1642  HG1 LYS P 104       5.193 -38.041 -16.600  0.00  0.00      PROA
ATOM   1643  HG2 LYS P 104       5.810 -38.064 -18.288  0.00  0.00      PROA
ATOM   1644  CD  LYS P 104       4.100 -36.884 -18.089  0.00  0.00      PROA
ATOM   1645  HD1 LYS P 104       4.345 -36.056 -18.787  0.00  0.00      PROA
ATOM   1646  HD2 LYS P 104       3.452 -36.431 -17.309  0.00  0.00      PROA
ATOM   1647  CE  LYS P 104       3.252 -37.986 -18.827  0.00  0.00      PROA
ATOM   1648  HE1 LYS P 104       3.252 -38.917 -18.221  0.00  0.00      PROA
ATOM   1649  HE2 LYS P 104       3.640 -38.184 -19.849  0.00  0.00      PROA
ATOM   1650  NZ  LYS P 104       1.864 -37.581 -18.979  0.00  0.00      PROA
ATOM   1651  HZ1 LYS P 104       1.586 -37.201 -18.052  0.00  0.00      PROA
ATOM   1652  HZ2 LYS P 104       1.324 -38.436 -19.219  0.00  0.00      PROA
ATOM   1653  HZ3 LYS P 104       1.763 -36.807 -19.666  0.00  0.00      PROA
ATOM   1654  C   LYS P 104       7.803 -34.211 -17.839  0.00  0.00      PROA
ATOM   1655  O   LYS P 104       8.973 -34.199 -17.926  0.00  0.00      PROA
ATOM   1656  N   LEU P 105       7.090 -33.119 -17.353  0.00  0.00      PROA
ATOM   1657  HN  LEU P 105       6.101 -33.221 -17.419  0.00  0.00      PROA
ATOM   1658  CA  LEU P 105       7.576 -31.866 -16.897  0.00  0.00      PROA
ATOM   1659  HA  LEU P 105       8.653 -31.779 -16.891  0.00  0.00      PROA
ATOM   1660  CB  LEU P 105       6.877 -30.737 -17.713  0.00  0.00      PROA
ATOM   1661  HB1 LEU P 105       6.880 -29.786 -17.137  0.00  0.00      PROA
ATOM   1662  HB2 LEU P 105       5.829 -31.003 -17.968  0.00  0.00      PROA
ATOM   1663  CG  LEU P 105       7.516 -30.569 -19.084  0.00  0.00      PROA
ATOM   1664  HG  LEU P 105       7.746 -31.564 -19.522  0.00  0.00      PROA
ATOM   1665  CD1 LEU P 105       6.666 -29.790 -20.038  0.00  0.00      PROA
ATOM   1666 HD11 LEU P 105       7.182 -29.611 -21.006  0.00  0.00      PROA
ATOM   1667 HD12 LEU P 105       6.362 -28.781 -19.687  0.00  0.00      PROA
ATOM   1668 HD13 LEU P 105       5.779 -30.397 -20.320  0.00  0.00      PROA
ATOM   1669  CD2 LEU P 105       8.877 -29.786 -19.066  0.00  0.00      PROA
ATOM   1670 HD21 LEU P 105       9.607 -30.446 -18.553  0.00  0.00      PROA
ATOM   1671 HD22 LEU P 105       8.699 -28.770 -18.654  0.00  0.00      PROA
ATOM   1672 HD23 LEU P 105       9.167 -29.554 -20.113  0.00  0.00      PROA
ATOM   1673  C   LEU P 105       7.212 -31.618 -15.398  0.00  0.00      PROA
ATOM   1674  O   LEU P 105       6.338 -32.313 -14.834  0.00  0.00      PROA
ATOM   1675  N   PRO P 106       7.870 -30.680 -14.668  0.00  0.00      PROA
ATOM   1676  CD  PRO P 106       8.907 -29.876 -15.264  0.00  0.00      PROA
ATOM   1677  HD1 PRO P 106       8.619 -29.172 -16.074  0.00  0.00      PROA
ATOM   1678  HD2 PRO P 106       9.748 -30.415 -15.750  0.00  0.00      PROA
ATOM   1679  CA  PRO P 106       7.960 -30.630 -13.201  0.00  0.00      PROA
```

| | | | | | | | | | | | |
|---|---|---|---|---|---|---|---|---|---|---|---|
| ATOM | 1680 | HA | PRO | P | 106 | 7.972 | -31.658 | -12.870 | 0.00 | 0.00 | PROA |
| ATOM | 1681 | CB | PRO | P | 106 | 9.290 | -29.927 | -12.958 | 0.00 | 0.00 | PROA |
| ATOM | 1682 | HB1 | PRO | P | 106 | 10.060 | -30.726 | -13.012 | 0.00 | 0.00 | PROA |
| ATOM | 1683 | HB2 | PRO | P | 106 | 9.388 | -29.374 | -11.999 | 0.00 | 0.00 | PROA |
| ATOM | 1684 | CG | PRO | P | 106 | 9.583 | -29.076 | -14.195 | 0.00 | 0.00 | PROA |
| ATOM | 1685 | HG1 | PRO | P | 106 | 9.208 | -28.031 | -14.164 | 0.00 | 0.00 | PROA |
| ATOM | 1686 | HG2 | PRO | P | 106 | 10.679 | -28.906 | -14.254 | 0.00 | 0.00 | PROA |
| ATOM | 1687 | C | PRO | P | 106 | 6.830 | -29.916 | -12.595 | 0.00 | 0.00 | PROA |
| ATOM | 1688 | O | PRO | P | 106 | 7.030 | -28.896 | -11.978 | 0.00 | 0.00 | PROA |
| ATOM | 1689 | N | VAL | P | 107 | 5.573 | -30.421 | -12.764 | 0.00 | 0.00 | PROA |
| ATOM | 1690 | HN | VAL | P | 107 | 5.581 | -31.289 | -13.254 | 0.00 | 0.00 | PROA |
| ATOM | 1691 | CA | VAL | P | 107 | 4.373 | -29.832 | -12.285 | 0.00 | 0.00 | PROA |
| ATOM | 1692 | HA | VAL | P | 107 | 4.208 | -29.093 | -13.055 | 0.00 | 0.00 | PROA |
| ATOM | 1693 | CB | VAL | P | 107 | 3.178 | -30.794 | -12.412 | 0.00 | 0.00 | PROA |
| ATOM | 1694 | HB | VAL | P | 107 | 3.026 | -31.069 | -13.478 | 0.00 | 0.00 | PROA |
| ATOM | 1695 | CG1 | VAL | P | 107 | 3.516 | -32.034 | -11.650 | 0.00 | 0.00 | PROA |
| ATOM | 1696 | HG11 | VAL | P | 107 | 3.613 | -31.811 | -10.566 | 0.00 | 0.00 | PROA |
| ATOM | 1697 | HG12 | VAL | P | 107 | 4.380 | -32.660 | -11.959 | 0.00 | 0.00 | PROA |
| ATOM | 1698 | HG13 | VAL | P | 107 | 2.595 | -32.652 | -11.718 | 0.00 | 0.00 | PROA |
| ATOM | 1699 | CG2 | VAL | P | 107 | 1.789 | -30.278 | -12.004 | 0.00 | 0.00 | PROA |
| ATOM | 1700 | HG21 | VAL | P | 107 | 1.421 | -29.375 | -12.537 | 0.00 | 0.00 | PROA |
| ATOM | 1701 | HG22 | VAL | P | 107 | 1.761 | -30.044 | -10.919 | 0.00 | 0.00 | PROA |
| ATOM | 1702 | HG23 | VAL | P | 107 | 1.022 | -31.067 | -12.157 | 0.00 | 0.00 | PROA |
| ATOM | 1703 | C | VAL | P | 107 | 4.342 | -29.154 | -10.874 | 0.00 | 0.00 | PROA |
| ATOM | 1704 | O | VAL | P | 107 | 4.562 | -29.814 | -9.886 | 0.00 | 0.00 | PROA |
| ATOM | 1705 | N | LEU | P | 108 | 4.005 | -27.832 | -10.818 | 0.00 | 0.00 | PROA |
| ATOM | 1706 | HN | LEU | P | 108 | 3.854 | -27.350 | -11.678 | 0.00 | 0.00 | PROA |
| ATOM | 1707 | CA | LEU | P | 108 | 3.779 | -27.160 | -9.500 | 0.00 | 0.00 | PROA |
| ATOM | 1708 | HA | LEU | P | 108 | 4.597 | -27.556 | -8.916 | 0.00 | 0.00 | PROA |
| ATOM | 1709 | CB | LEU | P | 108 | 3.876 | -25.673 | -9.474 | 0.00 | 0.00 | PROA |
| ATOM | 1710 | HB1 | LEU | P | 108 | 3.909 | -25.270 | -8.439 | 0.00 | 0.00 | PROA |
| ATOM | 1711 | HB2 | LEU | P | 108 | 2.960 | -25.205 | -9.894 | 0.00 | 0.00 | PROA |
| ATOM | 1712 | CG | LEU | P | 108 | 5.196 | -25.190 | -10.094 | 0.00 | 0.00 | PROA |
| ATOM | 1713 | HG | LEU | P | 108 | 5.225 | -25.370 | -11.190 | 0.00 | 0.00 | PROA |
| ATOM | 1714 | CD1 | LEU | P | 108 | 5.048 | -23.699 | -10.005 | 0.00 | 0.00 | PROA |
| ATOM | 1715 | HD11 | LEU | P | 108 | 5.886 | -23.113 | -10.441 | 0.00 | 0.00 | PROA |
| ATOM | 1716 | HD12 | LEU | P | 108 | 4.931 | -23.275 | -8.985 | 0.00 | 0.00 | PROA |
| ATOM | 1717 | HD13 | LEU | P | 108 | 4.043 | -23.507 | -10.437 | 0.00 | 0.00 | PROA |
| ATOM | 1718 | CD2 | LEU | P | 108 | 6.401 | -25.595 | -9.243 | 0.00 | 0.00 | PROA |
| ATOM | 1719 | HD21 | LEU | P | 108 | 6.563 | -26.671 | -9.470 | 0.00 | 0.00 | PROA |
| ATOM | 1720 | HD22 | LEU | P | 108 | 6.294 | -25.504 | -8.141 | 0.00 | 0.00 | PROA |
| ATOM | 1721 | HD23 | LEU | P | 108 | 7.322 | -25.093 | -9.609 | 0.00 | 0.00 | PROA |
| ATOM | 1722 | C | LEU | P | 108 | 2.512 | -27.638 | -8.742 | 0.00 | 0.00 | PROA |
| ATOM | 1723 | O | LEU | P | 108 | 1.494 | -28.014 | -9.431 | 0.00 | 0.00 | PROA |
| ATOM | 1724 | N | LEU | P | 109 | 2.558 | -27.731 | -7.430 | 0.00 | 0.00 | PROA |
| ATOM | 1725 | HN | LEU | P | 109 | 3.367 | -27.362 | -6.980 | 0.00 | 0.00 | PROA |
| ATOM | 1726 | CA | LEU | P | 109 | 1.518 | -28.403 | -6.716 | 0.00 | 0.00 | PROA |
| ATOM | 1727 | HA | LEU | P | 109 | 0.879 | -28.996 | -7.353 | 0.00 | 0.00 | PROA |
| ATOM | 1728 | CB | LEU | P | 109 | 1.938 | -29.421 | -5.592 | 0.00 | 0.00 | PROA |
| ATOM | 1729 | HB1 | LEU | P | 109 | 2.350 | -29.031 | -4.636 | 0.00 | 0.00 | PROA |
| ATOM | 1730 | HB2 | LEU | P | 109 | 2.709 | -30.080 | -6.046 | 0.00 | 0.00 | PROA |
| ATOM | 1731 | CG | LEU | P | 109 | 0.754 | -30.399 | -5.205 | 0.00 | 0.00 | PROA |
| ATOM | 1732 | HG | LEU | P | 109 | -0.099 | -30.322 | -5.912 | 0.00 | 0.00 | PROA |
| ATOM | 1733 | CD1 | LEU | P | 109 | 1.095 | -31.894 | -5.207 | 0.00 | 0.00 | PROA |
| ATOM | 1734 | HD11 | LEU | P | 109 | 0.348 | -32.448 | -4.601 | 0.00 | 0.00 | PROA |
| ATOM | 1735 | HD12 | LEU | P | 109 | 2.054 | -32.102 | -4.687 | 0.00 | 0.00 | PROA |
| ATOM | 1736 | HD13 | LEU | P | 109 | 1.232 | -32.249 | -6.251 | 0.00 | 0.00 | PROA |
| ATOM | 1737 | CD2 | LEU | P | 109 | 0.266 | -29.940 | -3.814 | 0.00 | 0.00 | PROA |
| ATOM | 1738 | HD21 | LEU | P | 109 | 1.124 | -29.945 | -3.109 | 0.00 | 0.00 | PROA |
| ATOM | 1739 | HD22 | LEU | P | 109 | -0.578 | -30.550 | -3.427 | 0.00 | 0.00 | PROA |
| ATOM | 1740 | HD23 | LEU | P | 109 | -0.119 | -28.901 | -3.894 | 0.00 | 0.00 | PROA |
| ATOM | 1741 | C | LEU | P | 109 | 0.664 | -27.311 | -6.209 | 0.00 | 0.00 | PROA |
| ATOM | 1742 | O | LEU | P | 109 | 1.099 | -26.299 | -5.647 | 0.00 | 0.00 | PROA |
| ATOM | 1743 | N | LEU | P | 110 | -0.674 | -27.387 | -6.619 | 0.00 | 0.00 | PROA |
| ATOM | 1744 | HN | LEU | P | 110 | -1.072 | -28.225 | -6.985 | 0.00 | 0.00 | PROA |
| ATOM | 1745 | CA | LEU | P | 110 | -1.620 | -26.382 | -6.156 | 0.00 | 0.00 | PROA |
| ATOM | 1746 | HA | LEU | P | 110 | -1.152 | -25.409 | -6.160 | 0.00 | 0.00 | PROA |
| ATOM | 1747 | CB | LEU | P | 110 | -2.883 | -26.406 | -7.046 | 0.00 | 0.00 | PROA |
| ATOM | 1748 | HB1 | LEU | P | 110 | -3.663 | -25.679 | -6.736 | 0.00 | 0.00 | PROA |
| ATOM | 1749 | HB2 | LEU | P | 110 | -3.358 | -27.403 | -6.933 | 0.00 | 0.00 | PROA |
| ATOM | 1750 | CG | LEU | P | 110 | -2.532 | -26.218 | -8.564 | 0.00 | 0.00 | PROA |
| ATOM | 1751 | HG | LEU | P | 110 | -1.778 | -26.960 | -8.904 | 0.00 | 0.00 | PROA |
| ATOM | 1752 | CD1 | LEU | P | 110 | -3.853 | -26.287 | -9.340 | 0.00 | 0.00 | PROA |

| | | | | | | | | | | | |
|---|---|---|---|---|---|---|---|---|---|---|---|
| ATOM | 1753 | HD11 | LEU | P | 110 | -4.516 | -25.523 | -8.880 | 0.00 | 0.00 | PROA |
| ATOM | 1754 | HD12 | LEU | P | 110 | -4.360 | -27.231 | -9.047 | 0.00 | 0.00 | PROA |
| ATOM | 1755 | HD13 | LEU | P | 110 | -3.739 | -26.153 | -10.437 | 0.00 | 0.00 | PROA |
| ATOM | 1756 | CD2 | LEU | P | 110 | -1.960 | -24.759 | -8.821 | 0.00 | 0.00 | PROA |
| ATOM | 1757 | HD21 | LEU | P | 110 | -0.863 | -24.666 | -8.672 | 0.00 | 0.00 | PROA |
| ATOM | 1758 | HD22 | LEU | P | 110 | -2.600 | -24.058 | -8.243 | 0.00 | 0.00 | PROA |
| ATOM | 1759 | HD23 | LEU | P | 110 | -1.990 | -24.705 | -9.930 | 0.00 | 0.00 | PROA |
| ATOM | 1760 | C | LEU | P | 110 | -2.140 | -26.583 | -4.779 | 0.00 | 0.00 | PROA |
| ATOM | 1761 | O | LEU | P | 110 | -2.812 | -27.501 | -4.454 | 0.00 | 0.00 | PROA |
| ATOM | 1762 | N | GLY | P | 111 | -1.823 | -25.639 | -3.877 | 0.00 | 0.00 | PROA |
| ATOM | 1763 | HN | GLY | P | 111 | -1.335 | -24.835 | -4.208 | 0.00 | 0.00 | PROA |
| ATOM | 1764 | CA | GLY | P | 111 | -2.158 | -25.662 | -2.469 | 0.00 | 0.00 | PROA |
| ATOM | 1765 | HA1 | GLY | P | 111 | -1.469 | -24.971 | -2.005 | 0.00 | 0.00 | PROA |
| ATOM | 1766 | HA2 | GLY | P | 111 | -2.015 | -26.704 | -2.221 | 0.00 | 0.00 | PROA |
| ATOM | 1767 | C | GLY | P | 111 | -3.470 | -25.219 | -2.223 | 0.00 | 0.00 | PROA |
| ATOM | 1768 | O | GLY | P | 111 | -4.139 | -24.706 | -3.124 | 0.00 | 0.00 | PROA |
| ATOM | 1769 | N | ARG | P | 112 | -3.958 | -25.373 | -1.004 | 0.00 | 0.00 | PROA |
| ATOM | 1770 | HN | ARG | P | 112 | -3.343 | -25.526 | -0.235 | 0.00 | 0.00 | PROA |
| ATOM | 1771 | CA | ARG | P | 112 | -5.337 | -25.039 | -0.679 | 0.00 | 0.00 | PROA |
| ATOM | 1772 | HA | ARG | P | 112 | -5.954 | -25.195 | -1.551 | 0.00 | 0.00 | PROA |
| ATOM | 1773 | CB | ARG | P | 112 | -5.972 | -26.024 | 0.317 | 0.00 | 0.00 | PROA |
| ATOM | 1774 | HB1 | ARG | P | 112 | -6.975 | -25.688 | 0.658 | 0.00 | 0.00 | PROA |
| ATOM | 1775 | HB2 | ARG | P | 112 | -5.340 | -26.061 | 1.230 | 0.00 | 0.00 | PROA |
| ATOM | 1776 | CG | ARG | P | 112 | -6.275 | -27.461 | -0.284 | 0.00 | 0.00 | PROA |
| ATOM | 1777 | HG1 | ARG | P | 112 | -6.830 | -27.338 | -1.238 | 0.00 | 0.00 | PROA |
| ATOM | 1778 | HG2 | ARG | P | 112 | -7.007 | -27.929 | 0.409 | 0.00 | 0.00 | PROA |
| ATOM | 1779 | CD | ARG | P | 112 | -5.004 | -28.298 | -0.601 | 0.00 | 0.00 | PROA |
| ATOM | 1780 | HD1 | ARG | P | 112 | -4.463 | -27.804 | -1.436 | 0.00 | 0.00 | PROA |
| ATOM | 1781 | HD2 | ARG | P | 112 | -5.324 | -29.323 | -0.884 | 0.00 | 0.00 | PROA |
| ATOM | 1782 | NE | ARG | P | 112 | -4.077 | -28.462 | 0.616 | 0.00 | 0.00 | PROA |
| ATOM | 1783 | HE | ARG | P | 112 | -4.416 | -28.110 | 1.489 | 0.00 | 0.00 | PROA |
| ATOM | 1784 | CZ | ARG | P | 112 | -2.736 | -28.368 | 0.571 | 0.00 | 0.00 | PROA |
| ATOM | 1785 | NH1 | ARG | P | 112 | -2.043 | -28.809 | -0.477 | 0.00 | 0.00 | PROA |
| ATOM | 1786 | HH11 | ARG | P | 112 | -2.601 | -29.152 | -1.233 | 0.00 | 0.00 | PROA |
| ATOM | 1787 | HH12 | ARG | P | 112 | -1.055 | -28.658 | -0.471 | 0.00 | 0.00 | PROA |
| ATOM | 1788 | NH2 | ARG | P | 112 | -2.046 | -27.916 | 1.587 | 0.00 | 0.00 | PROA |
| ATOM | 1789 | HH21 | ARG | P | 112 | -2.509 | -27.983 | 2.471 | 0.00 | 0.00 | PROA |
| ATOM | 1790 | HH22 | ARG | P | 112 | -1.063 | -27.759 | 1.489 | 0.00 | 0.00 | PROA |
| ATOM | 1791 | C | ARG | P | 112 | -5.412 | -23.621 | -0.207 | 0.00 | 0.00 | PROA |
| ATOM | 1792 | O | ARG | P | 112 | -4.998 | -23.254 | 0.886 | 0.00 | 0.00 | PROA |
| ATOM | 1793 | N | SER | P | 113 | -5.907 | -22.717 | -1.070 | 0.00 | 0.00 | PROA |
| ATOM | 1794 | HN | SER | P | 113 | -6.316 | -22.878 | -1.965 | 0.00 | 0.00 | PROA |
| ATOM | 1795 | CA | SER | P | 113 | -5.939 | -21.312 | -0.795 | 0.00 | 0.00 | PROA |
| ATOM | 1796 | HA | SER | P | 113 | -5.117 | -20.981 | -0.177 | 0.00 | 0.00 | PROA |
| ATOM | 1797 | CB | SER | P | 113 | -5.874 | -20.556 | -2.075 | 0.00 | 0.00 | PROA |
| ATOM | 1798 | HB1 | SER | P | 113 | -4.968 | -20.818 | -2.662 | 0.00 | 0.00 | PROA |
| ATOM | 1799 | HB2 | SER | P | 113 | -5.799 | -19.448 | -2.040 | 0.00 | 0.00 | PROA |
| ATOM | 1800 | OG | SER | P | 113 | -7.012 | -20.797 | -2.948 | 0.00 | 0.00 | PROA |
| ATOM | 1801 | HG1 | SER | P | 113 | -7.050 | -21.748 | -3.074 | 0.00 | 0.00 | PROA |
| ATOM | 1802 | C | SER | P | 113 | -7.289 | -20.957 | -0.100 | 0.00 | 0.00 | PROA |
| ATOM | 1803 | O | SER | P | 113 | -7.503 | -19.820 | 0.255 | 0.00 | 0.00 | PROA |
| ATOM | 1804 | N | SER | P | 114 | -8.198 | -21.934 | 0.161 | 0.00 | 0.00 | PROA |
| ATOM | 1805 | HN | SER | P | 114 | -8.191 | -22.759 | -0.399 | 0.00 | 0.00 | PROA |
| ATOM | 1806 | CA | SER | P | 114 | -9.291 | -21.631 | 1.052 | 0.00 | 0.00 | PROA |
| ATOM | 1807 | HA | SER | P | 114 | -9.544 | -20.587 | 0.941 | 0.00 | 0.00 | PROA |
| ATOM | 1808 | CB | SER | P | 114 | -10.573 | -22.372 | 0.726 | 0.00 | 0.00 | PROA |
| ATOM | 1809 | HB1 | SER | P | 114 | -11.053 | -21.923 | -0.170 | 0.00 | 0.00 | PROA |
| ATOM | 1810 | HB2 | SER | P | 114 | -11.371 | -22.248 | 1.490 | 0.00 | 0.00 | PROA |
| ATOM | 1811 | OG | SER | P | 114 | -10.360 | -23.786 | 0.689 | 0.00 | 0.00 | PROA |
| ATOM | 1812 | HG1 | SER | P | 114 | -9.632 | -24.071 | 0.132 | 0.00 | 0.00 | PROA |
| ATOM | 1813 | C | SER | P | 114 | -8.990 | -21.841 | 2.508 | 0.00 | 0.00 | PROA |
| ATOM | 1814 | O | SER | P | 114 | -9.677 | -21.261 | 3.292 | 0.00 | 0.00 | PROA |
| ATOM | 1815 | N | GLU | P | 115 | -8.093 | -22.742 | 2.810 | 0.00 | 0.00 | PROA |
| ATOM | 1816 | HN | GLU | P | 115 | -7.791 | -23.470 | 2.200 | 0.00 | 0.00 | PROA |
| ATOM | 1817 | CA | GLU | P | 115 | -7.694 | -22.948 | 4.162 | 0.00 | 0.00 | PROA |
| ATOM | 1818 | HA | GLU | P | 115 | -8.538 | -22.896 | 4.834 | 0.00 | 0.00 | PROA |
| ATOM | 1819 | CB | GLU | P | 115 | -7.117 | -24.345 | 4.210 | 0.00 | 0.00 | PROA |
| ATOM | 1820 | HB1 | GLU | P | 115 | -6.580 | -24.470 | 5.174 | 0.00 | 0.00 | PROA |
| ATOM | 1821 | HB2 | GLU | P | 115 | -6.308 | -24.531 | 3.471 | 0.00 | 0.00 | PROA |
| ATOM | 1822 | CG | GLU | P | 115 | -8.257 | -25.388 | 4.215 | 0.00 | 0.00 | PROA |
| ATOM | 1823 | HG1 | GLU | P | 115 | -8.883 | -25.413 | 3.298 | 0.00 | 0.00 | PROA |
| ATOM | 1824 | HG2 | GLU | P | 115 | -8.931 | -25.246 | 5.087 | 0.00 | 0.00 | PROA |
| ATOM | 1825 | CD | GLU | P | 115 | -7.657 | -26.737 | 4.336 | 0.00 | 0.00 | PROA |

| | | | | | | | | | | | |
|---|---|---|---|---|---|---|---|---|---|---|---|
| ATOM | 1826 | OE1 | GLU | P | 115 | -8.108 | -27.549 | 5.198 | 0.00 | 0.00 | PROA |
| ATOM | 1827 | OE2 | GLU | P | 115 | -6.698 | -27.109 | 3.582 | 0.00 | 0.00 | PROA |
| ATOM | 1828 | C | GLU | P | 115 | -6.628 | -21.888 | 4.569 | 0.00 | 0.00 | PROA |
| ATOM | 1829 | O | GLU | P | 115 | -6.339 | -21.792 | 5.763 | 0.00 | 0.00 | PROA |
| ATOM | 1830 | N | LEU | P | 116 | -6.255 | -20.990 | 3.655 | 0.00 | 0.00 | PROA |
| ATOM | 1831 | HN | LEU | P | 116 | -6.605 | -21.014 | 2.722 | 0.00 | 0.00 | PROA |
| ATOM | 1832 | CA | LEU | P | 116 | -5.389 | -19.868 | 3.903 | 0.00 | 0.00 | PROA |
| ATOM | 1833 | HA | LEU | P | 116 | -4.584 | -20.201 | 4.542 | 0.00 | 0.00 | PROA |
| ATOM | 1834 | CB | LEU | P | 116 | -4.939 | -19.280 | 2.498 | 0.00 | 0.00 | PROA |
| ATOM | 1835 | HB1 | LEU | P | 116 | -5.852 | -19.072 | 1.902 | 0.00 | 0.00 | PROA |
| ATOM | 1836 | HB2 | LEU | P | 116 | -4.419 | -20.083 | 1.933 | 0.00 | 0.00 | PROA |
| ATOM | 1837 | CG | LEU | P | 116 | -3.904 | -18.114 | 2.411 | 0.00 | 0.00 | PROA |
| ATOM | 1838 | HG | LEU | P | 116 | -4.245 | -17.203 | 2.946 | 0.00 | 0.00 | PROA |
| ATOM | 1839 | CD1 | LEU | P | 116 | -2.598 | -18.374 | 3.270 | 0.00 | 0.00 | PROA |
| ATOM | 1840 | HD11 | LEU | P | 116 | -2.027 | -17.434 | 3.113 | 0.00 | 0.00 | PROA |
| ATOM | 1841 | HD12 | LEU | P | 116 | -1.970 | -19.196 | 2.865 | 0.00 | 0.00 | PROA |
| ATOM | 1842 | HD13 | LEU | P | 116 | -2.904 | -18.510 | 4.330 | 0.00 | 0.00 | PROA |
| ATOM | 1843 | CD2 | LEU | P | 116 | -3.498 | -17.825 | 0.967 | 0.00 | 0.00 | PROA |
| ATOM | 1844 | HD21 | LEU | P | 116 | -3.106 | -18.825 | 0.682 | 0.00 | 0.00 | PROA |
| ATOM | 1845 | HD22 | LEU | P | 116 | -2.736 | -17.023 | 1.070 | 0.00 | 0.00 | PROA |
| ATOM | 1846 | HD23 | LEU | P | 116 | -4.317 | -17.483 | 0.300 | 0.00 | 0.00 | PROA |
| ATOM | 1847 | C | LEU | P | 116 | -6.216 | -18.870 | 4.690 | 0.00 | 0.00 | PROA |
| ATOM | 1848 | O | LEU | P | 116 | -7.425 | -18.730 | 4.562 | 0.00 | 0.00 | PROA |
| ATOM | 1849 | N | GLN | P | 117 | -5.543 | -18.011 | 5.556 | 0.00 | 0.00 | PROA |
| ATOM | 1850 | HN | GLN | P | 117 | -4.552 | -18.016 | 5.661 | 0.00 | 0.00 | PROA |
| ATOM | 1851 | CA | GLN | P | 117 | -6.221 | -17.125 | 6.526 | 0.00 | 0.00 | PROA |
| ATOM | 1852 | HA | GLN | P | 117 | -7.055 | -16.667 | 6.017 | 0.00 | 0.00 | PROA |
| ATOM | 1853 | CB | GLN | P | 117 | -6.384 | -17.804 | 7.926 | 0.00 | 0.00 | PROA |
| ATOM | 1854 | HB1 | GLN | P | 117 | -6.540 | -17.091 | 8.764 | 0.00 | 0.00 | PROA |
| ATOM | 1855 | HB2 | GLN | P | 117 | -5.400 | -18.227 | 8.220 | 0.00 | 0.00 | PROA |
| ATOM | 1856 | CG | GLN | P | 117 | -7.511 | -18.891 | 8.187 | 0.00 | 0.00 | PROA |
| ATOM | 1857 | HG1 | GLN | P | 117 | -7.681 | -19.122 | 9.260 | 0.00 | 0.00 | PROA |
| ATOM | 1858 | HG2 | GLN | P | 117 | -7.261 | -19.917 | 7.842 | 0.00 | 0.00 | PROA |
| ATOM | 1859 | CD | GLN | P | 117 | -8.924 | -18.535 | 7.668 | 0.00 | 0.00 | PROA |
| ATOM | 1860 | OE1 | GLN | P | 117 | -9.740 | -19.327 | 7.110 | 0.00 | 0.00 | PROA |
| ATOM | 1861 | NE2 | GLN | P | 117 | -9.301 | -17.245 | 7.811 | 0.00 | 0.00 | PROA |
| ATOM | 1862 | HE21 | GLN | P | 117 | -8.719 | -16.652 | 8.367 | 0.00 | 0.00 | PROA |
| ATOM | 1863 | HE22 | GLN | P | 117 | -10.279 | -17.055 | 7.729 | 0.00 | 0.00 | PROA |
| ATOM | 1864 | C | GLN | P | 117 | -5.183 | -15.976 | 6.753 | 0.00 | 0.00 | PROA |
| ATOM | 1865 | O | GLN | P | 117 | -4.003 | -16.276 | 6.597 | 0.00 | 0.00 | PROA |
| ATOM | 1866 | N | PRO | P | 118 | -5.559 | -14.690 | 7.049 | 0.00 | 0.00 | PROA |
| ATOM | 1867 | CD | PRO | P | 118 | -6.888 | -14.169 | 6.801 | 0.00 | 0.00 | PROA |
| ATOM | 1868 | HD1 | PRO | P | 118 | -7.618 | -14.686 | 7.459 | 0.00 | 0.00 | PROA |
| ATOM | 1869 | HD2 | PRO | P | 118 | -7.103 | -14.204 | 5.711 | 0.00 | 0.00 | PROA |
| ATOM | 1870 | CA | PRO | P | 118 | -4.645 | -13.676 | 7.482 | 0.00 | 0.00 | PROA |
| ATOM | 1871 | HA | PRO | P | 118 | -4.137 | -13.449 | 6.557 | 0.00 | 0.00 | PROA |
| ATOM | 1872 | CB | PRO | P | 118 | -5.512 | -12.546 | 8.072 | 0.00 | 0.00 | PROA |
| ATOM | 1873 | HB1 | PRO | P | 118 | -5.064 | -11.542 | 7.913 | 0.00 | 0.00 | PROA |
| ATOM | 1874 | HB2 | PRO | P | 118 | -5.837 | -12.770 | 9.110 | 0.00 | 0.00 | PROA |
| ATOM | 1875 | CG | PRO | P | 118 | -6.776 | -12.651 | 7.190 | 0.00 | 0.00 | PROA |
| ATOM | 1876 | HG1 | PRO | P | 118 | -7.606 | -12.252 | 7.811 | 0.00 | 0.00 | PROA |
| ATOM | 1877 | HG2 | PRO | P | 118 | -6.788 | -12.055 | 6.252 | 0.00 | 0.00 | PROA |
| ATOM | 1878 | C | PRO | P | 118 | -3.446 | -14.035 | 8.429 | 0.00 | 0.00 | PROA |
| ATOM | 1879 | O | PRO | P | 118 | -3.658 | -14.813 | 9.391 | 0.00 | 0.00 | PROA |
| ATOM | 1880 | N | GLY | P | 119 | -2.255 | -13.428 | 8.259 | 0.00 | 0.00 | PROA |
| ATOM | 1881 | HN | GLY | P | 119 | -2.166 | -12.869 | 7.439 | 0.00 | 0.00 | PROA |
| ATOM | 1882 | CA | GLY | P | 119 | -1.160 | -13.514 | 9.196 | 0.00 | 0.00 | PROA |
| ATOM | 1883 | HA1 | GLY | P | 119 | -1.521 | -13.628 | 10.208 | 0.00 | 0.00 | PROA |
| ATOM | 1884 | HA2 | GLY | P | 119 | -0.608 | -12.606 | 8.999 | 0.00 | 0.00 | PROA |
| ATOM | 1885 | C | GLY | P | 119 | -0.264 | -14.657 | 8.874 | 0.00 | 0.00 | PROA |
| ATOM | 1886 | O | GLY | P | 119 | 0.299 | -15.243 | 9.750 | 0.00 | 0.00 | PROA |
| ATOM | 1887 | N | GLU | P | 120 | -0.089 | -14.914 | 7.540 | 0.00 | 0.00 | PROA |
| ATOM | 1888 | HN | GLU | P | 120 | -0.700 | -14.410 | 6.934 | 0.00 | 0.00 | PROA |
| ATOM | 1889 | CA | GLU | P | 120 | 0.579 | -16.002 | 6.943 | 0.00 | 0.00 | PROA |
| ATOM | 1890 | HA | GLU | P | 120 | 1.174 | -16.573 | 7.640 | 0.00 | 0.00 | PROA |
| ATOM | 1891 | CB | GLU | P | 120 | -0.342 | -17.018 | 6.308 | 0.00 | 0.00 | PROA |
| ATOM | 1892 | HB1 | GLU | P | 120 | -0.970 | -16.498 | 5.554 | 0.00 | 0.00 | PROA |
| ATOM | 1893 | HB2 | GLU | P | 120 | -1.055 | -17.339 | 7.098 | 0.00 | 0.00 | PROA |
| ATOM | 1894 | CG | GLU | P | 120 | 0.301 | -18.311 | 5.761 | 0.00 | 0.00 | PROA |
| ATOM | 1895 | HG1 | GLU | P | 120 | 0.894 | -18.135 | 4.838 | 0.00 | 0.00 | PROA |
| ATOM | 1896 | HG2 | GLU | P | 120 | -0.569 | -18.995 | 5.655 | 0.00 | 0.00 | PROA |
| ATOM | 1897 | CD | GLU | P | 120 | 1.124 | -18.986 | 6.863 | 0.00 | 0.00 | PROA |
| ATOM | 1898 | OE1 | GLU | P | 120 | 0.647 | -19.279 | 7.975 | 0.00 | 0.00 | PROA |

```
ATOM   1899  OE2 GLU P 120       2.356 -19.062   6.683  0.00  0.00      PROA
ATOM   1900  C   GLU P 120       1.505 -15.403   5.959  0.00  0.00      PROA
ATOM   1901  O   GLU P 120       0.996 -14.877   4.990  0.00  0.00      PROA
ATOM   1902  N   PHE P 121       2.807 -15.585   6.125  0.00  0.00      PROA
ATOM   1903  HN  PHE P 121       3.039 -16.074   6.962  0.00  0.00      PROA
ATOM   1904  CA  PHE P 121       3.905 -14.989   5.357  0.00  0.00      PROA
ATOM   1905  HA  PHE P 121       3.493 -14.103   4.897  0.00  0.00      PROA
ATOM   1906  CB  PHE P 121       5.030 -14.539   6.381  0.00  0.00      PROA
ATOM   1907  HB1 PHE P 121       5.466 -15.397   6.936  0.00  0.00      PROA
ATOM   1908  HB2 PHE P 121       4.487 -13.891   7.102  0.00  0.00      PROA
ATOM   1909  CG  PHE P 121       6.102 -13.769   5.722  0.00  0.00      PROA
ATOM   1910  CD1 PHE P 121       7.370 -14.388   5.543  0.00  0.00      PROA
ATOM   1911  HD1 PHE P 121       7.456 -15.457   5.673  0.00  0.00      PROA
ATOM   1912  CE1 PHE P 121       8.480 -13.640   5.074  0.00  0.00      PROA
ATOM   1913  HE1 PHE P 121       9.419 -14.144   4.895  0.00  0.00      PROA
ATOM   1914  CZ  PHE P 121       8.344 -12.219   4.902  0.00  0.00      PROA
ATOM   1915  HZ  PHE P 121       9.174 -11.698   4.448  0.00  0.00      PROA
ATOM   1916  CD2 PHE P 121       5.964 -12.345   5.544  0.00  0.00      PROA
ATOM   1917  HD2 PHE P 121       5.067 -11.837   5.866  0.00  0.00      PROA
ATOM   1918  CE2 PHE P 121       7.128 -11.620   5.177  0.00  0.00      PROA
ATOM   1919  HE2 PHE P 121       7.046 -10.576   4.912  0.00  0.00      PROA
ATOM   1920  C   PHE P 121       4.255 -15.711   4.170  0.00  0.00      PROA
ATOM   1921  O   PHE P 121       4.535 -16.923   4.242  0.00  0.00      PROA
ATOM   1922  N   VAL P 122       4.209 -15.064   3.010  0.00  0.00      PROA
ATOM   1923  HN  VAL P 122       4.065 -14.087   2.875  0.00  0.00      PROA
ATOM   1924  CA  VAL P 122       4.159 -15.828   1.795  0.00  0.00      PROA
ATOM   1925  HA  VAL P 122       4.544 -16.835   1.855  0.00  0.00      PROA
ATOM   1926  CB  VAL P 122       2.803 -15.813   1.176  0.00  0.00      PROA
ATOM   1927  HB  VAL P 122       2.862 -16.246   0.155  0.00  0.00      PROA
ATOM   1928  CG1 VAL P 122       1.799 -16.692   1.898  0.00  0.00      PROA
ATOM   1929 HG11 VAL P 122       2.169 -17.735   1.799  0.00  0.00      PROA
ATOM   1930 HG12 VAL P 122       0.774 -16.738   1.473  0.00  0.00      PROA
ATOM   1931 HG13 VAL P 122       1.765 -16.405   2.971  0.00  0.00      PROA
ATOM   1932  CG2 VAL P 122       2.334 -14.345   1.031  0.00  0.00      PROA
ATOM   1933 HG21 VAL P 122       1.415 -14.321   0.407  0.00  0.00      PROA
ATOM   1934 HG22 VAL P 122       3.082 -13.831   0.390  0.00  0.00      PROA
ATOM   1935 HG23 VAL P 122       2.091 -13.808   1.973  0.00  0.00      PROA
ATOM   1936  C   VAL P 122       5.059 -15.167   0.730  0.00  0.00      PROA
ATOM   1937  O   VAL P 122       5.567 -14.092   1.017  0.00  0.00      PROA
ATOM   1938  N   VAL P 123       5.406 -15.924  -0.275  0.00  0.00      PROA
ATOM   1939  HN  VAL P 123       4.994 -16.813  -0.457  0.00  0.00      PROA
ATOM   1940  CA  VAL P 123       6.430 -15.580  -1.235  0.00  0.00      PROA
ATOM   1941  HA  VAL P 123       6.900 -14.618  -1.095  0.00  0.00      PROA
ATOM   1942  CB  VAL P 123       7.543 -16.647  -1.282  0.00  0.00      PROA
ATOM   1943  HB  VAL P 123       7.141 -17.646  -1.554  0.00  0.00      PROA
ATOM   1944  CG1 VAL P 123       8.717 -16.305  -2.227  0.00  0.00      PROA
ATOM   1945 HG11 VAL P 123       8.451 -16.320  -3.305  0.00  0.00      PROA
ATOM   1946 HG12 VAL P 123       9.641 -16.895  -2.049  0.00  0.00      PROA
ATOM   1947 HG13 VAL P 123       9.040 -15.251  -2.092  0.00  0.00      PROA
ATOM   1948  CG2 VAL P 123       8.146 -16.731   0.177  0.00  0.00      PROA
ATOM   1949 HG21 VAL P 123       8.443 -15.698   0.459  0.00  0.00      PROA
ATOM   1950 HG22 VAL P 123       8.896 -17.549   0.218  0.00  0.00      PROA
ATOM   1951 HG23 VAL P 123       7.327 -17.040   0.861  0.00  0.00      PROA
ATOM   1952  C   VAL P 123       5.872 -15.505  -2.713  0.00  0.00      PROA
ATOM   1953  O   VAL P 123       5.443 -16.469  -3.291  0.00  0.00      PROA
ATOM   1954  N   ALA P 124       5.956 -14.321  -3.365  0.00  0.00      PROA
ATOM   1955  HN  ALA P 124       6.412 -13.549  -2.929  0.00  0.00      PROA
ATOM   1956  CA  ALA P 124       5.730 -14.256  -4.817  0.00  0.00      PROA
ATOM   1957  HA  ALA P 124       5.013 -14.998  -5.135  0.00  0.00      PROA
ATOM   1958  CB  ALA P 124       4.967 -12.979  -5.153  0.00  0.00      PROA
ATOM   1959  HB1 ALA P 124       4.582 -12.935  -6.194  0.00  0.00      PROA
ATOM   1960  HB2 ALA P 124       5.641 -12.101  -5.056  0.00  0.00      PROA
ATOM   1961  HB3 ALA P 124       4.227 -12.863  -4.332  0.00  0.00      PROA
ATOM   1962  C   ALA P 124       7.029 -14.375  -5.602  0.00  0.00      PROA
ATOM   1963  O   ALA P 124       7.932 -13.531  -5.576  0.00  0.00      PROA
ATOM   1964  N   ILE P 125       7.102 -15.407  -6.437  0.00  0.00      PROA
ATOM   1965  HN  ILE P 125       6.289 -15.971  -6.558  0.00  0.00      PROA
ATOM   1966  CA  ILE P 125       8.209 -15.720  -7.249  0.00  0.00      PROA
ATOM   1967  HA  ILE P 125       9.083 -15.235  -6.840  0.00  0.00      PROA
ATOM   1968  CB  ILE P 125       8.438 -17.223  -7.390  0.00  0.00      PROA
ATOM   1969  HB  ILE P 125       7.615 -17.709  -7.956  0.00  0.00      PROA
ATOM   1970  CG2 ILE P 125       9.661 -17.491  -8.260  0.00  0.00      PROA
ATOM   1971 HG21 ILE P 125       9.927 -18.556  -8.095  0.00  0.00      PROA
```

```
ATOM   1972 HG22 ILE P 125      10.482 -16.770  -8.056  0.00  0.00      PROA
ATOM   1973 HG23 ILE P 125       9.416 -17.406  -9.341  0.00  0.00      PROA
ATOM   1974  CG1 ILE P 125       8.502 -17.863  -5.992  0.00  0.00      PROA
ATOM   1975 HG11 ILE P 125       8.213 -18.935  -5.994  0.00  0.00      PROA
ATOM   1976 HG12 ILE P 125       7.832 -17.414  -5.228  0.00  0.00      PROA
ATOM   1977  CD  ILE P 125       9.999 -17.845  -5.425  0.00  0.00      PROA
ATOM   1978  HD1 ILE P 125       9.885 -18.401  -4.470  0.00  0.00      PROA
ATOM   1979  HD2 ILE P 125      10.289 -16.788  -5.244  0.00  0.00      PROA
ATOM   1980  HD3 ILE P 125      10.810 -18.331  -6.009  0.00  0.00      PROA
ATOM   1981  C   ILE P 125       7.875 -15.164  -8.732  0.00  0.00      PROA
ATOM   1982  O   ILE P 125       6.819 -15.507  -9.350  0.00  0.00      PROA
ATOM   1983  N   GLY P 126       8.734 -14.363  -9.359  0.00  0.00      PROA
ATOM   1984  HN  GLY P 126       9.521 -14.063  -8.826  0.00  0.00      PROA
ATOM   1985  CA  GLY P 126       8.677 -14.074 -10.764  0.00  0.00      PROA
ATOM   1986  HA1 GLY P 126       8.770 -13.022 -10.990  0.00  0.00      PROA
ATOM   1987  HA2 GLY P 126       7.735 -14.423 -11.162  0.00  0.00      PROA
ATOM   1988  C   GLY P 126       9.667 -14.899 -11.552  0.00  0.00      PROA
ATOM   1989  O   GLY P 126       9.479 -15.494 -12.615  0.00  0.00      PROA
ATOM   1990  N   SER P 127      10.860 -15.015 -10.898  0.00  0.00      PROA
ATOM   1991  HN  SER P 127      10.937 -14.513 -10.040  0.00  0.00      PROA
ATOM   1992  CA  SER P 127      11.915 -15.914 -11.336  0.00  0.00      PROA
ATOM   1993  HA  SER P 127      11.534 -16.783 -11.853  0.00  0.00      PROA
ATOM   1994  CB  SER P 127      12.920 -15.151 -12.261  0.00  0.00      PROA
ATOM   1995  HB1 SER P 127      13.221 -14.269 -11.656  0.00  0.00      PROA
ATOM   1996  HB2 SER P 127      12.409 -14.771 -13.171  0.00  0.00      PROA
ATOM   1997  OG  SER P 127      14.028 -15.975 -12.686  0.00  0.00      PROA
ATOM   1998  HG1 SER P 127      13.699 -16.572 -13.362  0.00  0.00      PROA
ATOM   1999  C   SER P 127      12.767 -16.478 -10.225  0.00  0.00      PROA
ATOM   2000  O   SER P 127      13.005 -15.675  -9.331  0.00  0.00      PROA
ATOM   2001  N   PRO P 128      13.088 -17.749 -10.057  0.00  0.00      PROA
ATOM   2002  CD  PRO P 128      12.591 -18.840 -10.916  0.00  0.00      PROA
ATOM   2003  HD1 PRO P 128      13.259 -18.974 -11.793  0.00  0.00      PROA
ATOM   2004  HD2 PRO P 128      11.554 -18.657 -11.273  0.00  0.00      PROA
ATOM   2005  CA  PRO P 128      13.804 -18.174  -8.919  0.00  0.00      PROA
ATOM   2006  HA  PRO P 128      13.379 -17.782  -8.006  0.00  0.00      PROA
ATOM   2007  CB  PRO P 128      13.695 -19.718  -8.977  0.00  0.00      PROA
ATOM   2008  HB1 PRO P 128      13.377 -20.078  -7.975  0.00  0.00      PROA
ATOM   2009  HB2 PRO P 128      14.643 -20.175  -9.331  0.00  0.00      PROA
ATOM   2010  CG  PRO P 128      12.639 -20.082 -10.036  0.00  0.00      PROA
ATOM   2011  HG1 PRO P 128      12.889 -20.908 -10.736  0.00  0.00      PROA
ATOM   2012  HG2 PRO P 128      11.671 -20.236  -9.513  0.00  0.00      PROA
ATOM   2013  C   PRO P 128      15.333 -17.760  -9.070  0.00  0.00      PROA
ATOM   2014  O   PRO P 128      16.149 -18.103  -8.216  0.00  0.00      PROA
ATOM   2015  N   PHE P 129      15.661 -17.042 -10.108  0.00  0.00      PROA
ATOM   2016  HN  PHE P 129      15.088 -16.704 -10.850  0.00  0.00      PROA
ATOM   2017  CA  PHE P 129      17.020 -16.734 -10.546  0.00  0.00      PROA
ATOM   2018  HA  PHE P 129      17.749 -17.045  -9.813  0.00  0.00      PROA
ATOM   2019  CB  PHE P 129      17.193 -17.501 -11.870  0.00  0.00      PROA
ATOM   2020  HB1 PHE P 129      18.248 -17.458 -12.215  0.00  0.00      PROA
ATOM   2021  HB2 PHE P 129      16.697 -17.123 -12.789  0.00  0.00      PROA
ATOM   2022  CG  PHE P 129      16.949 -19.011 -11.844  0.00  0.00      PROA
ATOM   2023  CD1 PHE P 129      16.029 -19.533 -12.720  0.00  0.00      PROA
ATOM   2024  HD1 PHE P 129      15.458 -19.071 -13.512  0.00  0.00      PROA
ATOM   2025  CE1 PHE P 129      15.643 -20.862 -12.469  0.00  0.00      PROA
ATOM   2026  HE1 PHE P 129      14.832 -21.229 -13.081  0.00  0.00      PROA
ATOM   2027  CZ  PHE P 129      16.275 -21.635 -11.544  0.00  0.00      PROA
ATOM   2028  HZ  PHE P 129      16.079 -22.695 -11.480  0.00  0.00      PROA
ATOM   2029  CD2 PHE P 129      17.599 -19.790 -10.809  0.00  0.00      PROA
ATOM   2030  HD2 PHE P 129      18.225 -19.256 -10.109  0.00  0.00      PROA
ATOM   2031  CE2 PHE P 129      17.196 -21.148 -10.648  0.00  0.00      PROA
ATOM   2032  HE2 PHE P 129      17.588 -21.807  -9.889  0.00  0.00      PROA
ATOM   2033  C   PHE P 129      17.192 -15.249 -10.685  0.00  0.00      PROA
ATOM   2034  O   PHE P 129      17.807 -14.591  -9.852  0.00  0.00      PROA
ATOM   2035  N   SER P 130      16.691 -14.714 -11.719  0.00  0.00      PROA
ATOM   2036  HN  SER P 130      16.274 -15.173 -12.499  0.00  0.00      PROA
ATOM   2037  CA  SER P 130      16.889 -13.293 -12.236  0.00  0.00      PROA
ATOM   2038  HA  SER P 130      17.897 -13.241 -12.619  0.00  0.00      PROA
ATOM   2039  CB  SER P 130      16.013 -12.986 -13.484  0.00  0.00      PROA
ATOM   2040  HB1 SER P 130      14.955 -13.125 -13.177  0.00  0.00      PROA
ATOM   2041  HB2 SER P 130      16.230 -13.780 -14.229  0.00  0.00      PROA
ATOM   2042  OG  SER P 130      16.166 -11.604 -13.946  0.00  0.00      PROA
ATOM   2043  HG1 SER P 130      16.435 -11.625 -14.867  0.00  0.00      PROA
ATOM   2044  C   SER P 130      16.714 -12.218 -11.170  0.00  0.00      PROA
```

| | | | | | | | | | | | |
|---|---|---|---|---|---|---|---|---|---|---|---|
| ATOM | 2045 | O | SER | P | 130 | 15.708 | -12.078 | -10.519 | 0.00 | 0.00 | PROA |
| ATOM | 2046 | N | LEU | P | 131 | 17.752 | -11.276 | -11.090 | 0.00 | 0.00 | PROA |
| ATOM | 2047 | HN | LEU | P | 131 | 18.598 | -11.477 | -11.578 | 0.00 | 0.00 | PROA |
| ATOM | 2048 | CA | LEU | P | 131 | 17.723 | -10.081 | -10.286 | 0.00 | 0.00 | PROA |
| ATOM | 2049 | HA | LEU | P | 131 | 17.142 | -10.239 | -9.390 | 0.00 | 0.00 | PROA |
| ATOM | 2050 | CB | LEU | P | 131 | 19.042 | -9.761 | -9.757 | 0.00 | 0.00 | PROA |
| ATOM | 2051 | HB1 | LEU | P | 131 | 18.943 | -9.037 | -8.920 | 0.00 | 0.00 | PROA |
| ATOM | 2052 | HB2 | LEU | P | 131 | 19.807 | -9.359 | -10.456 | 0.00 | 0.00 | PROA |
| ATOM | 2053 | CG | LEU | P | 131 | 19.799 | -10.929 | -9.214 | 0.00 | 0.00 | PROA |
| ATOM | 2054 | HG | LEU | P | 131 | 19.888 | -11.649 | -10.054 | 0.00 | 0.00 | PROA |
| ATOM | 2055 | CD1 | LEU | P | 131 | 21.258 | -10.428 | -8.793 | 0.00 | 0.00 | PROA |
| ATOM | 2056 | HD11 | LEU | P | 131 | 21.845 | -11.302 | -8.440 | 0.00 | 0.00 | PROA |
| ATOM | 2057 | HD12 | LEU | P | 131 | 21.197 | -9.718 | -7.941 | 0.00 | 0.00 | PROA |
| ATOM | 2058 | HD13 | LEU | P | 131 | 21.718 | -9.869 | -9.636 | 0.00 | 0.00 | PROA |
| ATOM | 2059 | CD2 | LEU | P | 131 | 19.028 | -11.588 | -7.964 | 0.00 | 0.00 | PROA |
| ATOM | 2060 | HD21 | LEU | P | 131 | 18.881 | -10.898 | -7.106 | 0.00 | 0.00 | PROA |
| ATOM | 2061 | HD22 | LEU | P | 131 | 19.534 | -12.538 | -7.690 | 0.00 | 0.00 | PROA |
| ATOM | 2062 | HD23 | LEU | P | 131 | 18.034 | -11.914 | -8.340 | 0.00 | 0.00 | PROA |
| ATOM | 2063 | C | LEU | P | 131 | 17.152 | -8.886 | -10.913 | 0.00 | 0.00 | PROA |
| ATOM | 2064 | O | LEU | P | 131 | 17.202 | -7.761 | -10.420 | 0.00 | 0.00 | PROA |
| ATOM | 2065 | N | GLN | P | 132 | 16.426 | -9.072 | -12.086 | 0.00 | 0.00 | PROA |
| ATOM | 2066 | HN | GLN | P | 132 | 16.504 | -9.842 | -12.715 | 0.00 | 0.00 | PROA |
| ATOM | 2067 | CA | GLN | P | 132 | 15.559 | -8.034 | -12.663 | 0.00 | 0.00 | PROA |
| ATOM | 2068 | HA | GLN | P | 132 | 15.693 | -7.072 | -12.191 | 0.00 | 0.00 | PROA |
| ATOM | 2069 | CB | GLN | P | 132 | 15.815 | -7.997 | -14.184 | 0.00 | 0.00 | PROA |
| ATOM | 2070 | HB1 | GLN | P | 132 | 15.154 | -7.192 | -14.570 | 0.00 | 0.00 | PROA |
| ATOM | 2071 | HB2 | GLN | P | 132 | 15.483 | -8.966 | -14.614 | 0.00 | 0.00 | PROA |
| ATOM | 2072 | CG | GLN | P | 132 | 17.296 | -7.674 | -14.547 | 0.00 | 0.00 | PROA |
| ATOM | 2073 | HG1 | GLN | P | 132 | 17.193 | -7.321 | -15.596 | 0.00 | 0.00 | PROA |
| ATOM | 2074 | HG2 | GLN | P | 132 | 18.000 | -8.529 | -14.463 | 0.00 | 0.00 | PROA |
| ATOM | 2075 | CD | GLN | P | 132 | 17.997 | -6.526 | -13.886 | 0.00 | 0.00 | PROA |
| ATOM | 2076 | OE1 | GLN | P | 132 | 17.504 | -5.806 | -13.067 | 0.00 | 0.00 | PROA |
| ATOM | 2077 | NE2 | GLN | P | 132 | 19.272 | -6.251 | -14.258 | 0.00 | 0.00 | PROA |
| ATOM | 2078 | HE21 | GLN | P | 132 | 19.547 | -5.439 | -13.743 | 0.00 | 0.00 | PROA |
| ATOM | 2079 | HE22 | GLN | P | 132 | 19.712 | -6.763 | -14.996 | 0.00 | 0.00 | PROA |
| ATOM | 2080 | C | GLN | P | 132 | 14.075 | -8.356 | -12.328 | 0.00 | 0.00 | PROA |
| ATOM | 2081 | O | GLN | P | 132 | 13.230 | -7.454 | -12.478 | 0.00 | 0.00 | PROA |
| ATOM | 2082 | N | ASN | P | 133 | 13.861 | -9.556 | -11.750 | 0.00 | 0.00 | PROA |
| ATOM | 2083 | HN | ASN | P | 133 | 14.599 | -10.221 | -11.671 | 0.00 | 0.00 | PROA |
| ATOM | 2084 | CA | ASN | P | 133 | 12.548 | -9.929 | -11.338 | 0.00 | 0.00 | PROA |
| ATOM | 2085 | HA | ASN | P | 133 | 11.790 | -9.168 | -11.231 | 0.00 | 0.00 | PROA |
| ATOM | 2086 | CB | ASN | P | 133 | 12.088 | -11.092 | -12.300 | 0.00 | 0.00 | PROA |
| ATOM | 2087 | HB1 | ASN | P | 133 | 11.126 | -11.571 | -12.020 | 0.00 | 0.00 | PROA |
| ATOM | 2088 | HB2 | ASN | P | 133 | 12.862 | -11.875 | -12.149 | 0.00 | 0.00 | PROA |
| ATOM | 2089 | CG | ASN | P | 133 | 12.079 | -10.523 | -13.738 | 0.00 | 0.00 | PROA |
| ATOM | 2090 | OD1 | ASN | P | 133 | 12.732 | -10.947 | -14.669 | 0.00 | 0.00 | PROA |
| ATOM | 2091 | ND2 | ASN | P | 133 | 11.262 | -9.405 | -13.893 | 0.00 | 0.00 | PROA |
| ATOM | 2092 | HD21 | ASN | P | 133 | 10.845 | -9.051 | -13.056 | 0.00 | 0.00 | PROA |
| ATOM | 2093 | HD22 | ASN | P | 133 | 10.767 | -9.276 | -14.752 | 0.00 | 0.00 | PROA |
| ATOM | 2094 | C | ASN | P | 133 | 12.735 | -10.533 | -9.877 | 0.00 | 0.00 | PROA |
| ATOM | 2095 | O | ASN | P | 133 | 12.328 | -11.639 | -9.581 | 0.00 | 0.00 | PROA |
| ATOM | 2096 | N | THR | P | 134 | 13.261 | -9.710 | -8.955 | 0.00 | 0.00 | PROA |
| ATOM | 2097 | HN | THR | P | 134 | 13.645 | -8.849 | -9.280 | 0.00 | 0.00 | PROA |
| ATOM | 2098 | CA | THR | P | 134 | 13.402 | -9.923 | -7.545 | 0.00 | 0.00 | PROA |
| ATOM | 2099 | HA | THR | P | 134 | 14.014 | -10.791 | -7.349 | 0.00 | 0.00 | PROA |
| ATOM | 2100 | CB | THR | P | 134 | 14.052 | -8.786 | -6.664 | 0.00 | 0.00 | PROA |
| ATOM | 2101 | HB | THR | P | 134 | 13.990 | -8.981 | -5.572 | 0.00 | 0.00 | PROA |
| ATOM | 2102 | OG1 | THR | P | 134 | 13.660 | -7.504 | -6.998 | 0.00 | 0.00 | PROA |
| ATOM | 2103 | HG1 | THR | P | 134 | 12.718 | -7.414 | -6.841 | 0.00 | 0.00 | PROA |
| ATOM | 2104 | CG2 | THR | P | 134 | 15.525 | -8.810 | -7.053 | 0.00 | 0.00 | PROA |
| ATOM | 2105 | HG21 | THR | P | 134 | 15.975 | -7.823 | -6.811 | 0.00 | 0.00 | PROA |
| ATOM | 2106 | HG22 | THR | P | 134 | 15.640 | -8.995 | -8.142 | 0.00 | 0.00 | PROA |
| ATOM | 2107 | HG23 | THR | P | 134 | 16.012 | -9.613 | -6.459 | 0.00 | 0.00 | PROA |
| ATOM | 2108 | C | THR | P | 134 | 11.980 | -10.135 | -6.960 | 0.00 | 0.00 | PROA |
| ATOM | 2109 | O | THR | P | 134 | 10.986 | -9.567 | -7.448 | 0.00 | 0.00 | PROA |
| ATOM | 2110 | N | VAL | P | 135 | 11.913 | -10.872 | -5.856 | 0.00 | 0.00 | PROA |
| ATOM | 2111 | HN | VAL | P | 135 | 12.762 | -11.245 | -5.490 | 0.00 | 0.00 | PROA |
| ATOM | 2112 | CA | VAL | P | 135 | 10.783 | -11.296 | -5.076 | 0.00 | 0.00 | PROA |
| ATOM | 2113 | HA | VAL | P | 135 | 10.021 | -11.644 | -5.758 | 0.00 | 0.00 | PROA |
| ATOM | 2114 | CB | VAL | P | 135 | 11.157 | -12.384 | -4.177 | 0.00 | 0.00 | PROA |
| ATOM | 2115 | HB | VAL | P | 135 | 12.074 | -12.123 | -3.607 | 0.00 | 0.00 | PROA |
| ATOM | 2116 | CG1 | VAL | P | 135 | 10.166 | -12.717 | -3.057 | 0.00 | 0.00 | PROA |
| ATOM | 2117 | HG11 | VAL | P | 135 | 9.188 | -13.024 | -3.484 | 0.00 | 0.00 | PROA |

```
ATOM   2118 HG12 VAL P 135       9.990 -11.879  -2.349  0.00  0.00      PROA
ATOM   2119 HG13 VAL P 135      10.476 -13.504  -2.336  0.00  0.00      PROA
ATOM   2120  CG2 VAL P 135      11.405 -13.679  -5.043  0.00  0.00      PROA
ATOM   2121 HG21 VAL P 135      12.276 -13.487  -5.706  0.00  0.00      PROA
ATOM   2122 HG22 VAL P 135      10.425 -13.751  -5.560  0.00  0.00      PROA
ATOM   2123 HG23 VAL P 135      11.482 -14.464  -4.260  0.00  0.00      PROA
ATOM   2124  C   VAL P 135      10.000 -10.170  -4.352  0.00  0.00      PROA
ATOM   2125  O   VAL P 135      10.534  -9.223  -3.805  0.00  0.00      PROA
ATOM   2126  N   THR P 136       8.659 -10.332  -4.258  0.00  0.00      PROA
ATOM   2127  HN  THR P 136       8.266 -10.991  -4.895  0.00  0.00      PROA
ATOM   2128  CA  THR P 136       7.632  -9.625  -3.486  0.00  0.00      PROA
ATOM   2129  HA  THR P 136       8.034  -8.713  -3.069  0.00  0.00      PROA
ATOM   2130  CB  THR P 136       6.369  -9.120  -4.171  0.00  0.00      PROA
ATOM   2131  HB  THR P 136       5.723  -9.969  -4.481  0.00  0.00      PROA
ATOM   2132  OG1 THR P 136       6.745  -8.384  -5.321  0.00  0.00      PROA
ATOM   2133  HG1 THR P 136       7.049  -8.978  -6.011  0.00  0.00      PROA
ATOM   2134  CG2 THR P 136       5.643  -8.132  -3.232  0.00  0.00      PROA
ATOM   2135 HG21 THR P 136       6.360  -7.560  -2.605  0.00  0.00      PROA
ATOM   2136 HG22 THR P 136       4.817  -8.638  -2.688  0.00  0.00      PROA
ATOM   2137 HG23 THR P 136       5.036  -7.550  -3.958  0.00  0.00      PROA
ATOM   2138  C   THR P 136       7.210 -10.539  -2.388  0.00  0.00      PROA
ATOM   2139  O   THR P 136       6.837 -11.726  -2.589  0.00  0.00      PROA
ATOM   2140  N   THR P 137       7.302 -10.048  -1.088  0.00  0.00      PROA
ATOM   2141  HN  THR P 137       7.686  -9.167  -0.823  0.00  0.00      PROA
ATOM   2142  CA  THR P 137       7.020 -10.983  -0.030  0.00  0.00      PROA
ATOM   2143  HA  THR P 137       6.164 -11.550  -0.364  0.00  0.00      PROA
ATOM   2144  CB  THR P 137       8.089 -11.981   0.403  0.00  0.00      PROA
ATOM   2145  HB  THR P 137       8.392 -12.605  -0.465  0.00  0.00      PROA
ATOM   2146  OG1 THR P 137       7.699 -12.817   1.469  0.00  0.00      PROA
ATOM   2147  HG1 THR P 137       6.812 -13.108   1.246  0.00  0.00      PROA
ATOM   2148  CG2 THR P 137       9.296 -11.200   0.956  0.00  0.00      PROA
ATOM   2149 HG21 THR P 137      10.190 -11.834   1.135  0.00  0.00      PROA
ATOM   2150 HG22 THR P 137       8.933 -10.680   1.869  0.00  0.00      PROA
ATOM   2151 HG23 THR P 137       9.466 -10.379   0.227  0.00  0.00      PROA
ATOM   2152  C   THR P 137       6.376 -10.264   1.176  0.00  0.00      PROA
ATOM   2153  O   THR P 137       6.689  -9.096   1.445  0.00  0.00      PROA
ATOM   2154  N   GLY P 138       5.348 -10.888   1.762  0.00  0.00      PROA
ATOM   2155  HN  GLY P 138       5.045 -11.793   1.473  0.00  0.00      PROA
ATOM   2156  CA  GLY P 138       4.624 -10.120   2.741  0.00  0.00      PROA
ATOM   2157  HA1 GLY P 138       4.001  -9.340   2.329  0.00  0.00      PROA
ATOM   2158  HA2 GLY P 138       5.295  -9.788   3.520  0.00  0.00      PROA
ATOM   2159  C   GLY P 138       3.680 -11.097   3.361  0.00  0.00      PROA
ATOM   2160  O   GLY P 138       3.521 -12.190   2.831  0.00  0.00      PROA
ATOM   2161  N   ILE P 139       3.023 -10.674   4.430  0.00  0.00      PROA
ATOM   2162  HN  ILE P 139       3.252  -9.779   4.804  0.00  0.00      PROA
ATOM   2163  CA  ILE P 139       1.913 -11.441   4.971  0.00  0.00      PROA
ATOM   2164  HA  ILE P 139       2.175 -12.484   5.071  0.00  0.00      PROA
ATOM   2165  CB  ILE P 139       1.530 -11.022   6.412  0.00  0.00      PROA
ATOM   2166  HB  ILE P 139       0.740 -11.672   6.845  0.00  0.00      PROA
ATOM   2167  CG2 ILE P 139       2.745 -11.334   7.301  0.00  0.00      PROA
ATOM   2168 HG21 ILE P 139       3.094 -12.383   7.187  0.00  0.00      PROA
ATOM   2169 HG22 ILE P 139       2.434 -11.152   8.352  0.00  0.00      PROA
ATOM   2170 HG23 ILE P 139       3.541 -10.584   7.105  0.00  0.00      PROA
ATOM   2171  CG1 ILE P 139       1.154  -9.562   6.605  0.00  0.00      PROA
ATOM   2172 HG11 ILE P 139       1.376  -9.068   5.635  0.00  0.00      PROA
ATOM   2173 HG12 ILE P 139       1.809  -9.089   7.366  0.00  0.00      PROA
ATOM   2174  CD  ILE P 139      -0.278  -9.433   6.969  0.00  0.00      PROA
ATOM   2175  HD1 ILE P 139      -0.890  -9.793   6.114  0.00  0.00      PROA
ATOM   2176  HD2 ILE P 139      -0.497  -8.379   7.242  0.00  0.00      PROA
ATOM   2177  HD3 ILE P 139      -0.522 -10.038   7.868  0.00  0.00      PROA
ATOM   2178  C   ILE P 139       0.589 -11.447   4.072  0.00  0.00      PROA
ATOM   2179  O   ILE P 139       0.323 -10.463   3.451  0.00  0.00      PROA
ATOM   2180  N   VAL P 140      -0.273 -12.473   4.079  0.00  0.00      PROA
ATOM   2181  HN  VAL P 140       0.091 -13.332   4.430  0.00  0.00      PROA
ATOM   2182  CA  VAL P 140      -1.589 -12.400   3.493  0.00  0.00      PROA
ATOM   2183  HA  VAL P 140      -1.511 -11.814   2.589  0.00  0.00      PROA
ATOM   2184  CB  VAL P 140      -2.139 -13.822   3.246  0.00  0.00      PROA
ATOM   2185  HB  VAL P 140      -1.962 -14.451   4.145  0.00  0.00      PROA
ATOM   2186  CG1 VAL P 140      -3.603 -13.769   2.690  0.00  0.00      PROA
ATOM   2187 HG11 VAL P 140      -3.816 -14.687   2.102  0.00  0.00      PROA
ATOM   2188 HG12 VAL P 140      -3.716 -12.928   1.973  0.00  0.00      PROA
ATOM   2189 HG13 VAL P 140      -4.406 -13.689   3.454  0.00  0.00      PROA
ATOM   2190  CG2 VAL P 140      -1.176 -14.484   2.182  0.00  0.00      PROA
```

```
ATOM   2191 HG21 VAL P 140      -1.399 -15.557   1.998  0.00  0.00      PROA
ATOM   2192 HG22 VAL P 140      -0.112 -14.446   2.499  0.00  0.00      PROA
ATOM   2193 HG23 VAL P 140      -1.274 -14.000   1.187  0.00  0.00      PROA
ATOM   2194  C   VAL P 140      -2.495 -11.676   4.430  0.00  0.00      PROA
ATOM   2195  O   VAL P 140      -2.630 -11.982   5.587  0.00  0.00      PROA
ATOM   2196  N   SER P 141      -3.100 -10.540   3.915  0.00  0.00      PROA
ATOM   2197  HN  SER P 141      -3.037 -10.247   2.964  0.00  0.00      PROA
ATOM   2198  CA  SER P 141      -4.063  -9.724   4.734  0.00  0.00      PROA
ATOM   2199  HA  SER P 141      -3.911  -9.889   5.790  0.00  0.00      PROA
ATOM   2200  CB  SER P 141      -3.759  -8.263   4.411  0.00  0.00      PROA
ATOM   2201  HB1 SER P 141      -2.666  -8.167   4.588  0.00  0.00      PROA
ATOM   2202  HB2 SER P 141      -4.303  -7.725   5.217  0.00  0.00      PROA
ATOM   2203  OG  SER P 141      -4.138  -7.874   3.122  0.00  0.00      PROA
ATOM   2204  HG1 SER P 141      -3.635  -8.246   2.394  0.00  0.00      PROA
ATOM   2205  C   SER P 141      -5.465 -10.111   4.418  0.00  0.00      PROA
ATOM   2206  O   SER P 141      -6.318 -10.030   5.331  0.00  0.00      PROA
ATOM   2207  N   THR P 142      -5.750 -10.583   3.254  0.00  0.00      PROA
ATOM   2208  HN  THR P 142      -5.072 -10.482   2.530  0.00  0.00      PROA
ATOM   2209  CA  THR P 142      -7.178 -10.959   3.014  0.00  0.00      PROA
ATOM   2210  HA  THR P 142      -7.643 -11.457   3.852  0.00  0.00      PROA
ATOM   2211  CB  THR P 142      -8.227  -9.817   2.801  0.00  0.00      PROA
ATOM   2212  HB  THR P 142      -8.203  -9.302   3.785  0.00  0.00      PROA
ATOM   2213  OG1 THR P 142      -9.552 -10.366   2.710  0.00  0.00      PROA
ATOM   2214  HG1 THR P 142     -10.150  -9.619   2.785  0.00  0.00      PROA
ATOM   2215  CG2 THR P 142      -7.978  -8.835   1.629  0.00  0.00      PROA
ATOM   2216 HG21 THR P 142      -8.702  -7.994   1.580  0.00  0.00      PROA
ATOM   2217 HG22 THR P 142      -7.859  -9.322   0.637  0.00  0.00      PROA
ATOM   2218 HG23 THR P 142      -7.023  -8.357   1.936  0.00  0.00      PROA
ATOM   2219  C   THR P 142      -7.118 -11.921   1.846  0.00  0.00      PROA
ATOM   2220  O   THR P 142      -6.271 -11.860   1.002  0.00  0.00      PROA
ATOM   2221  N   THR P 143      -8.067 -12.924   1.740  0.00  0.00      PROA
ATOM   2222  HN  THR P 143      -8.704 -13.047   2.497  0.00  0.00      PROA
ATOM   2223  CA  THR P 143      -8.214 -13.898   0.651  0.00  0.00      PROA
ATOM   2224  HA  THR P 143      -7.367 -13.764  -0.005  0.00  0.00      PROA
ATOM   2225  CB  THR P 143      -8.255 -15.336   1.120  0.00  0.00      PROA
ATOM   2226  HB  THR P 143      -8.183 -15.815   0.120  0.00  0.00      PROA
ATOM   2227  OG1 THR P 143      -9.429 -15.648   1.799  0.00  0.00      PROA
ATOM   2228  HG1 THR P 143     -10.070 -15.761   1.093  0.00  0.00      PROA
ATOM   2229  CG2 THR P 143      -6.991 -15.651   1.852  0.00  0.00      PROA
ATOM   2230 HG21 THR P 143      -7.077 -16.727   2.115  0.00  0.00      PROA
ATOM   2231 HG22 THR P 143      -6.769 -15.137   2.812  0.00  0.00      PROA
ATOM   2232 HG23 THR P 143      -6.173 -15.505   1.114  0.00  0.00      PROA
ATOM   2233  C   THR P 143      -9.326 -13.550  -0.322  0.00  0.00      PROA
ATOM   2234  O   THR P 143      -9.658 -14.317  -1.175  0.00  0.00      PROA
ATOM   2235  N   GLN P 144      -9.905 -12.294  -0.293  0.00  0.00      PROA
ATOM   2236  HN  GLN P 144      -9.458 -11.655   0.327  0.00  0.00      PROA
ATOM   2237  CA  GLN P 144     -11.118 -11.982  -1.044  0.00  0.00      PROA
ATOM   2238  HA  GLN P 144     -11.049 -12.319  -2.068  0.00  0.00      PROA
ATOM   2239  CB  GLN P 144     -12.271 -12.918  -0.510  0.00  0.00      PROA
ATOM   2240  HB1 GLN P 144     -12.101 -13.900  -1.000  0.00  0.00      PROA
ATOM   2241  HB2 GLN P 144     -13.223 -12.504  -0.906  0.00  0.00      PROA
ATOM   2242  CG  GLN P 144     -12.334 -13.153   1.011  0.00  0.00      PROA
ATOM   2243  HG1 GLN P 144     -11.363 -13.417   1.483  0.00  0.00      PROA
ATOM   2244  HG2 GLN P 144     -12.929 -14.079   1.166  0.00  0.00      PROA
ATOM   2245  CD  GLN P 144     -13.092 -11.980   1.743  0.00  0.00      PROA
ATOM   2246  OE1 GLN P 144     -14.222 -11.525   1.425  0.00  0.00      PROA
ATOM   2247  NE2 GLN P 144     -12.553 -11.556   2.874  0.00  0.00      PROA
ATOM   2248 HE21 GLN P 144     -11.767 -11.954   3.347  0.00  0.00      PROA
ATOM   2249 HE22 GLN P 144     -13.055 -10.851   3.376  0.00  0.00      PROA
ATOM   2250  C   GLN P 144     -11.408 -10.548  -1.304  0.00  0.00      PROA
ATOM   2251  O   GLN P 144     -12.391  -9.980  -0.801  0.00  0.00      PROA
ATOM   2252  N   ARG P 145     -10.518  -9.899  -2.088  0.00  0.00      PROA
ATOM   2253  HN  ARG P 145      -9.716 -10.312  -2.513  0.00  0.00      PROA
ATOM   2254  CA  ARG P 145     -10.570  -8.452  -2.304  0.00  0.00      PROA
ATOM   2255  HA  ARG P 145     -11.537  -8.028  -2.077  0.00  0.00      PROA
ATOM   2256  CB  ARG P 145      -9.520  -7.846  -1.331  0.00  0.00      PROA
ATOM   2257  HB1 ARG P 145      -8.539  -8.356  -1.437  0.00  0.00      PROA
ATOM   2258  HB2 ARG P 145      -9.942  -8.078  -0.330  0.00  0.00      PROA
ATOM   2259  CG  ARG P 145      -9.308  -6.281  -1.365  0.00  0.00      PROA
ATOM   2260  HG1 ARG P 145      -8.951  -5.964  -2.368  0.00  0.00      PROA
ATOM   2261  HG2 ARG P 145      -8.478  -6.055  -0.662  0.00  0.00      PROA
ATOM   2262  CD  ARG P 145     -10.660  -5.517  -1.166  0.00  0.00      PROA
ATOM   2263  HD1 ARG P 145     -11.202  -5.941  -0.293  0.00  0.00      PROA
```

```
ATOM   2264  HD2 ARG P 145     -11.434  -5.681  -1.946  0.00  0.00      PROA
ATOM   2265  NE  ARG P 145     -10.536  -3.973  -1.062  0.00  0.00      PROA
ATOM   2266  HE  ARG P 145      -9.657  -3.542  -1.265  0.00  0.00      PROA
ATOM   2267  CZ  ARG P 145     -11.461  -3.062  -0.879  0.00  0.00      PROA
ATOM   2268  NH1 ARG P 145     -12.752  -3.350  -0.841  0.00  0.00      PROA
ATOM   2269 HH11 ARG P 145     -12.977  -4.322  -0.775  0.00  0.00      PROA
ATOM   2270 HH12 ARG P 145     -13.360  -2.625  -0.517  0.00  0.00      PROA
ATOM   2271  NH2 ARG P 145     -11.181  -1.823  -0.619  0.00  0.00      PROA
ATOM   2272 HH21 ARG P 145     -10.195  -1.702  -0.734  0.00  0.00      PROA
ATOM   2273 HH22 ARG P 145     -11.972  -1.212  -0.650  0.00  0.00      PROA
ATOM   2274  C   ARG P 145     -10.169  -8.114  -3.756  0.00  0.00      PROA
ATOM   2275  O   ARG P 145      -9.093  -8.378  -4.323  0.00  0.00      PROA
ATOM   2276  N   GLY P 146     -11.071  -7.433  -4.484  0.00  0.00      PROA
ATOM   2277  HN  GLY P 146     -11.940  -7.255  -4.028  0.00  0.00      PROA
ATOM   2278  CA  GLY P 146     -10.723  -6.829  -5.733  0.00  0.00      PROA
ATOM   2279  HA1 GLY P 146     -11.602  -6.236  -5.934  0.00  0.00      PROA
ATOM   2280  HA2 GLY P 146     -10.405  -7.632  -6.382  0.00  0.00      PROA
ATOM   2281  C   GLY P 146      -9.624  -5.797  -5.708  0.00  0.00      PROA
ATOM   2282  O   GLY P 146      -9.356  -5.273  -4.654  0.00  0.00      PROA
ATOM   2283  N   GLY P 147      -9.030  -5.419  -6.852  0.00  0.00      PROA
ATOM   2284  HN  GLY P 147      -9.279  -5.857  -7.712  0.00  0.00      PROA
ATOM   2285  CA  GLY P 147      -7.896  -4.431  -6.787  0.00  0.00      PROA
ATOM   2286  HA1 GLY P 147      -6.949  -4.950  -6.794  0.00  0.00      PROA
ATOM   2287  HA2 GLY P 147      -8.128  -3.712  -6.015  0.00  0.00      PROA
ATOM   2288  C   GLY P 147      -7.808  -3.482  -7.915  0.00  0.00      PROA
ATOM   2289  O   GLY P 147      -6.680  -3.026  -8.187  0.00  0.00      PROA
ATOM   2290  N   LYS P 148      -8.950  -3.179  -8.562  0.00  0.00      PROA
ATOM   2291  HN  LYS P 148      -9.751  -3.654  -8.206  0.00  0.00      PROA
ATOM   2292  CA  LYS P 148      -9.244  -2.205  -9.617  0.00  0.00      PROA
ATOM   2293  HA  LYS P 148     -10.316  -2.122  -9.716  0.00  0.00      PROA
ATOM   2294  CB  LYS P 148      -8.863  -0.770  -9.106  0.00  0.00      PROA
ATOM   2295  HB1 LYS P 148      -8.883   0.005  -9.901  0.00  0.00      PROA
ATOM   2296  HB2 LYS P 148      -7.826  -0.824  -8.711  0.00  0.00      PROA
ATOM   2297  CG  LYS P 148      -9.638  -0.360  -7.836  0.00  0.00      PROA
ATOM   2298  HG1 LYS P 148      -9.115  -0.879  -7.004  0.00  0.00      PROA
ATOM   2299  HG2 LYS P 148     -10.665  -0.782  -7.881  0.00  0.00      PROA
ATOM   2300  CD  LYS P 148      -9.806   1.177  -7.626  0.00  0.00      PROA
ATOM   2301  HD1 LYS P 148     -10.416   1.247  -6.700  0.00  0.00      PROA
ATOM   2302  HD2 LYS P 148     -10.475   1.684  -8.355  0.00  0.00      PROA
ATOM   2303  CE  LYS P 148      -8.456   1.914  -7.502  0.00  0.00      PROA
ATOM   2304  HE1 LYS P 148      -8.034   1.933  -8.529  0.00  0.00      PROA
ATOM   2305  HE2 LYS P 148      -7.743   1.500  -6.757  0.00  0.00      PROA
ATOM   2306  NZ  LYS P 148      -8.689   3.291  -6.934  0.00  0.00      PROA
ATOM   2307  HZ1 LYS P 148      -9.577   3.278  -6.394  0.00  0.00      PROA
ATOM   2308  HZ2 LYS P 148      -8.774   3.940  -7.742  0.00  0.00      PROA
ATOM   2309  HZ3 LYS P 148      -7.925   3.669  -6.338  0.00  0.00      PROA
ATOM   2310  C   LYS P 148      -8.791  -2.485 -11.039  0.00  0.00      PROA
ATOM   2311  O   LYS P 148      -7.856  -3.253 -11.269  0.00  0.00      PROA
ATOM   2312  N   GLU P 149      -9.597  -2.131 -11.989  0.00  0.00      PROA
ATOM   2313  HN  GLU P 149     -10.271  -1.444 -11.727  0.00  0.00      PROA
ATOM   2314  CA  GLU P 149      -9.299  -2.302 -13.359  0.00  0.00      PROA
ATOM   2315  HA  GLU P 149      -8.385  -2.845 -13.547  0.00  0.00      PROA
ATOM   2316  CB  GLU P 149     -10.389  -3.014 -14.083  0.00  0.00      PROA
ATOM   2317  HB1 GLU P 149     -10.030  -3.181 -15.121  0.00  0.00      PROA
ATOM   2318  HB2 GLU P 149     -11.367  -2.486 -14.075  0.00  0.00      PROA
ATOM   2319  CG  GLU P 149     -10.539  -4.480 -13.605  0.00  0.00      PROA
ATOM   2320  HG1 GLU P 149     -11.136  -5.108 -14.300  0.00  0.00      PROA
ATOM   2321  HG2 GLU P 149     -11.215  -4.457 -12.723  0.00  0.00      PROA
ATOM   2322  CD  GLU P 149      -9.243  -5.249 -13.397  0.00  0.00      PROA
ATOM   2323  OE1 GLU P 149      -8.284  -5.244 -14.252  0.00  0.00      PROA
ATOM   2324  OE2 GLU P 149      -9.056  -5.933 -12.330  0.00  0.00      PROA
ATOM   2325  C   GLU P 149      -9.136  -0.954 -14.094  0.00  0.00      PROA
ATOM   2326  O   GLU P 149      -9.126   0.152 -13.475  0.00  0.00      PROA
ATOM   2327  N   LEU P 150      -8.895  -1.021 -15.410  0.00  0.00      PROA
ATOM   2328  HN  LEU P 150      -8.889  -1.826 -15.998  0.00  0.00      PROA
ATOM   2329  CA  LEU P 150      -8.512   0.073 -16.234  0.00  0.00      PROA
ATOM   2330  HA  LEU P 150      -7.698   0.631 -15.796  0.00  0.00      PROA
ATOM   2331  CB  LEU P 150      -8.134  -0.450 -17.682  0.00  0.00      PROA
ATOM   2332  HB1 LEU P 150      -8.066   0.375 -18.424  0.00  0.00      PROA
ATOM   2333  HB2 LEU P 150      -8.909  -1.101 -18.140  0.00  0.00      PROA
ATOM   2334  CG  LEU P 150      -6.746  -1.106 -17.707  0.00  0.00      PROA
ATOM   2335  HG  LEU P 150      -6.533  -1.580 -16.725  0.00  0.00      PROA
ATOM   2336  CD1 LEU P 150      -6.555  -2.101 -18.958  0.00  0.00      PROA
```

```
ATOM   2337 HD11 LEU P 150      -7.156  -3.014 -18.759  0.00  0.00      PROA
ATOM   2338 HD12 LEU P 150      -5.512  -2.431 -19.153  0.00  0.00      PROA
ATOM   2339 HD13 LEU P 150      -7.017  -1.582 -19.824  0.00  0.00      PROA
ATOM   2340  CD2 LEU P 150      -5.573  -0.018 -17.847  0.00  0.00      PROA
ATOM   2341 HD21 LEU P 150      -5.763   0.636 -18.725  0.00  0.00      PROA
ATOM   2342 HD22 LEU P 150      -4.600  -0.546 -17.944  0.00  0.00      PROA
ATOM   2343 HD23 LEU P 150      -5.565   0.695 -16.995  0.00  0.00      PROA
ATOM   2344  C   LEU P 150      -9.595   1.151 -16.343  0.00  0.00      PROA
ATOM   2345  O   LEU P 150     -10.765   0.905 -16.061  0.00  0.00      PROA
ATOM   2346  N   GLY P 151      -9.195   2.405 -16.565  0.00  0.00      PROA
ATOM   2347  HN  GLY P 151      -8.341   2.654 -17.014  0.00  0.00      PROA
ATOM   2348  CA  GLY P 151     -10.123   3.461 -16.130  0.00  0.00      PROA
ATOM   2349  HA1 GLY P 151     -11.077   3.274 -16.600  0.00  0.00      PROA
ATOM   2350  HA2 GLY P 151      -9.654   4.400 -16.384  0.00  0.00      PROA
ATOM   2351  C   GLY P 151     -10.453   3.533 -14.646  0.00  0.00      PROA
ATOM   2352  O   GLY P 151      -9.558   3.473 -13.788  0.00  0.00      PROA
ATOM   2353  N   LEU P 152     -11.717   3.871 -14.283  0.00  0.00      PROA
ATOM   2354  HN  LEU P 152     -12.385   4.170 -14.960  0.00  0.00      PROA
ATOM   2355  CA  LEU P 152     -12.139   4.000 -12.855  0.00  0.00      PROA
ATOM   2356  HA  LEU P 152     -11.311   4.008 -12.161  0.00  0.00      PROA
ATOM   2357  CB  LEU P 152     -12.819   5.347 -12.741  0.00  0.00      PROA
ATOM   2358  HB1 LEU P 152     -12.855   5.565 -11.652  0.00  0.00      PROA
ATOM   2359  HB2 LEU P 152     -13.788   5.315 -13.283  0.00  0.00      PROA
ATOM   2360  CG  LEU P 152     -11.970   6.562 -13.283  0.00  0.00      PROA
ATOM   2361  HG  LEU P 152     -11.575   6.283 -14.283  0.00  0.00      PROA
ATOM   2362  CD1 LEU P 152     -12.860   7.776 -13.313  0.00  0.00      PROA
ATOM   2363 HD11 LEU P 152     -12.306   8.675 -13.659  0.00  0.00      PROA
ATOM   2364 HD12 LEU P 152     -13.166   8.167 -12.319  0.00  0.00      PROA
ATOM   2365 HD13 LEU P 152     -13.827   7.713 -13.856  0.00  0.00      PROA
ATOM   2366  CD2 LEU P 152     -10.798   6.780 -12.336  0.00  0.00      PROA
ATOM   2367 HD21 LEU P 152     -10.240   5.821 -12.285  0.00  0.00      PROA
ATOM   2368 HD22 LEU P 152     -11.139   7.091 -11.326  0.00  0.00      PROA
ATOM   2369 HD23 LEU P 152     -10.088   7.536 -12.734  0.00  0.00      PROA
ATOM   2370  C   LEU P 152     -12.996   2.797 -12.453  0.00  0.00      PROA
ATOM   2371  O   LEU P 152     -13.818   2.967 -11.594  0.00  0.00      PROA
ATOM   2372  N   ARG P 153     -12.745   1.587 -13.026  0.00  0.00      PROA
ATOM   2373  HN  ARG P 153     -11.944   1.570 -13.619  0.00  0.00      PROA
ATOM   2374  CA  ARG P 153     -13.463   0.319 -12.745  0.00  0.00      PROA
ATOM   2375  HA  ARG P 153     -14.437   0.576 -12.356  0.00  0.00      PROA
ATOM   2376  CB  ARG P 153     -13.648  -0.426 -14.054  0.00  0.00      PROA
ATOM   2377  HB1 ARG P 153     -14.128  -1.421 -13.941  0.00  0.00      PROA
ATOM   2378  HB2 ARG P 153     -12.598  -0.707 -14.281  0.00  0.00      PROA
ATOM   2379  CG  ARG P 153     -14.345   0.234 -15.297  0.00  0.00      PROA
ATOM   2380  HG1 ARG P 153     -14.329   1.344 -15.248  0.00  0.00      PROA
ATOM   2381  HG2 ARG P 153     -15.433   0.049 -15.171  0.00  0.00      PROA
ATOM   2382  CD  ARG P 153     -13.970  -0.223 -16.700  0.00  0.00      PROA
ATOM   2383  HD1 ARG P 153     -12.894  -0.063 -16.928  0.00  0.00      PROA
ATOM   2384  HD2 ARG P 153     -14.513   0.238 -17.553  0.00  0.00      PROA
ATOM   2385  NE  ARG P 153     -14.282  -1.699 -16.699  0.00  0.00      PROA
ATOM   2386  HE  ARG P 153     -15.179  -1.976 -16.353  0.00  0.00      PROA
ATOM   2387  CZ  ARG P 153     -13.537  -2.644 -17.285  0.00  0.00      PROA
ATOM   2388  NH1 ARG P 153     -13.579  -3.894 -16.798  0.00  0.00      PROA
ATOM   2389 HH11 ARG P 153     -14.043  -4.195 -15.964  0.00  0.00      PROA
ATOM   2390 HH12 ARG P 153     -12.773  -4.435 -17.037  0.00  0.00      PROA
ATOM   2391  NH2 ARG P 153     -12.579  -2.354 -18.133  0.00  0.00      PROA
ATOM   2392 HH21 ARG P 153     -12.470  -1.415 -18.460  0.00  0.00      PROA
ATOM   2393 HH22 ARG P 153     -12.093  -3.069 -18.635  0.00  0.00      PROA
ATOM   2394  C   ARG P 153     -12.890  -0.501 -11.604  0.00  0.00      PROA
ATOM   2395  O   ARG P 153     -11.730  -0.414 -11.304  0.00  0.00      PROA
ATOM   2396  N   ASN P 154     -13.710  -1.383 -10.918  0.00  0.00      PROA
ATOM   2397  HN  ASN P 154     -14.627  -1.447 -11.306  0.00  0.00      PROA
ATOM   2398  CA  ASN P 154     -13.245  -2.387 -10.009  0.00  0.00      PROA
ATOM   2399  HA  ASN P 154     -12.263  -2.072  -9.689  0.00  0.00      PROA
ATOM   2400  CB  ASN P 154     -14.290  -2.394  -8.818  0.00  0.00      PROA
ATOM   2401  HB1 ASN P 154     -14.318  -3.392  -8.331  0.00  0.00      PROA
ATOM   2402  HB2 ASN P 154     -15.283  -2.234  -9.291  0.00  0.00      PROA
ATOM   2403  CG  ASN P 154     -14.037  -1.323  -7.756  0.00  0.00      PROA
ATOM   2404  OD1 ASN P 154     -13.506  -1.516  -6.600  0.00  0.00      PROA
ATOM   2405  ND2 ASN P 154     -14.306  -0.026  -8.103  0.00  0.00      PROA
ATOM   2406 HD21 ASN P 154     -13.940   0.676  -7.492  0.00  0.00      PROA
ATOM   2407 HD22 ASN P 154     -14.771   0.150  -8.970  0.00  0.00      PROA
ATOM   2408  C   ASN P 154     -12.993  -3.738 -10.636  0.00  0.00      PROA
ATOM   2409  O   ASN P 154     -13.492  -4.042 -11.694  0.00  0.00      PROA
```

| | | | | | | | | | | | |
|---|---|---|---|---|---|---|---|---|---|---|---|
| ATOM | 2410 | N | SER | P | 155 | -12.301 | -4.669 | -9.924 | 0.00 | 0.00 | PROA |
| ATOM | 2411 | HN | SER | P | 155 | -11.860 | -4.351 | -9.088 | 0.00 | 0.00 | PROA |
| ATOM | 2412 | CA | SER | P | 155 | -12.003 | -6.021 | -10.388 | 0.00 | 0.00 | PROA |
| ATOM | 2413 | HA | SER | P | 155 | -11.634 | -5.907 | -11.396 | 0.00 | 0.00 | PROA |
| ATOM | 2414 | CB | SER | P | 155 | -10.911 | -6.720 | -9.604 | 0.00 | 0.00 | PROA |
| ATOM | 2415 | HB1 | SER | P | 155 | -10.634 | -7.778 | -9.798 | 0.00 | 0.00 | PROA |
| ATOM | 2416 | HB2 | SER | P | 155 | -11.322 | -6.708 | -8.571 | 0.00 | 0.00 | PROA |
| ATOM | 2417 | OG | SER | P | 155 | -9.680 | -5.927 | -9.713 | 0.00 | 0.00 | PROA |
| ATOM | 2418 | HG1 | SER | P | 155 | -9.526 | -5.727 | -10.639 | 0.00 | 0.00 | PROA |
| ATOM | 2419 | C | SER | P | 155 | -13.278 | -6.908 | -10.453 | 0.00 | 0.00 | PROA |
| ATOM | 2420 | O | SER | P | 155 | -14.306 | -6.658 | -9.792 | 0.00 | 0.00 | PROA |
| ATOM | 2421 | N | ASP | P | 156 | -13.268 | -7.999 | -11.320 | 0.00 | 0.00 | PROA |
| ATOM | 2422 | HN | ASP | P | 156 | -12.407 | -8.070 | -11.819 | 0.00 | 0.00 | PROA |
| ATOM | 2423 | CA | ASP | P | 156 | -14.326 | -8.850 | -11.578 | 0.00 | 0.00 | PROA |
| ATOM | 2424 | HA | ASP | P | 156 | -15.191 | -8.482 | -11.046 | 0.00 | 0.00 | PROA |
| ATOM | 2425 | CB | ASP | P | 156 | -14.668 | -8.959 | -13.128 | 0.00 | 0.00 | PROA |
| ATOM | 2426 | HB1 | ASP | P | 156 | -15.363 | -9.780 | -13.404 | 0.00 | 0.00 | PROA |
| ATOM | 2427 | HB2 | ASP | P | 156 | -13.763 | -8.994 | -13.772 | 0.00 | 0.00 | PROA |
| ATOM | 2428 | CG | ASP | P | 156 | -15.342 | -7.654 | -13.580 | 0.00 | 0.00 | PROA |
| ATOM | 2429 | OD1 | ASP | P | 156 | -15.420 | -7.403 | -14.789 | 0.00 | 0.00 | PROA |
| ATOM | 2430 | OD2 | ASP | P | 156 | -16.006 | -6.997 | -12.728 | 0.00 | 0.00 | PROA |
| ATOM | 2431 | C | ASP | P | 156 | -14.059 | -10.250 | -11.054 | 0.00 | 0.00 | PROA |
| ATOM | 2432 | O | ASP | P | 156 | -14.911 | -11.150 | -11.207 | 0.00 | 0.00 | PROA |
| ATOM | 2433 | N | MET | P | 157 | -12.904 | -10.430 | -10.360 | 0.00 | 0.00 | PROA |
| ATOM | 2434 | HN | MET | P | 157 | -12.217 | -9.737 | -10.158 | 0.00 | 0.00 | PROA |
| ATOM | 2435 | CA | MET | P | 157 | -12.622 | -11.699 | -9.734 | 0.00 | 0.00 | PROA |
| ATOM | 2436 | HA | MET | P | 157 | -13.541 | -12.208 | -9.484 | 0.00 | 0.00 | PROA |
| ATOM | 2437 | CB | MET | P | 157 | -11.725 | -12.544 | -10.538 | 0.00 | 0.00 | PROA |
| ATOM | 2438 | HB1 | MET | P | 157 | -10.663 | -12.217 | -10.559 | 0.00 | 0.00 | PROA |
| ATOM | 2439 | HB2 | MET | P | 157 | -11.998 | -12.656 | -11.609 | 0.00 | 0.00 | PROA |
| ATOM | 2440 | CG | MET | P | 157 | -11.522 | -14.005 | -10.004 | 0.00 | 0.00 | PROA |
| ATOM | 2441 | HG1 | MET | P | 157 | -11.316 | -14.603 | -10.917 | 0.00 | 0.00 | PROA |
| ATOM | 2442 | HG2 | MET | P | 157 | -12.581 | -14.241 | -9.762 | 0.00 | 0.00 | PROA |
| ATOM | 2443 | SD | MET | P | 157 | -10.380 | -14.462 | -8.661 | 0.00 | 0.00 | PROA |
| ATOM | 2444 | CE | MET | P | 157 | -11.135 | -16.032 | -8.268 | 0.00 | 0.00 | PROA |
| ATOM | 2445 | HE1 | MET | P | 157 | -10.662 | -16.796 | -8.921 | 0.00 | 0.00 | PROA |
| ATOM | 2446 | HE2 | MET | P | 157 | -12.244 | -16.086 | -8.309 | 0.00 | 0.00 | PROA |
| ATOM | 2447 | HE3 | MET | P | 157 | -10.887 | -16.251 | -7.207 | 0.00 | 0.00 | PROA |
| ATOM | 2448 | C | MET | P | 157 | -11.981 | -11.256 | -8.348 | 0.00 | 0.00 | PROA |
| ATOM | 2449 | O | MET | P | 157 | -11.209 | -10.293 | -8.221 | 0.00 | 0.00 | PROA |
| ATOM | 2450 | N | ASP | P | 158 | -12.267 | -12.030 | -7.357 | 0.00 | 0.00 | PROA |
| ATOM | 2451 | HN | ASP | P | 158 | -12.840 | -12.818 | -7.569 | 0.00 | 0.00 | PROA |
| ATOM | 2452 | CA | ASP | P | 158 | -11.827 | -11.801 | -5.969 | 0.00 | 0.00 | PROA |
| ATOM | 2453 | HA | ASP | P | 158 | -11.701 | -10.730 | -5.908 | 0.00 | 0.00 | PROA |
| ATOM | 2454 | CB | ASP | P | 158 | -12.871 | -12.358 | -4.900 | 0.00 | 0.00 | PROA |
| ATOM | 2455 | HB1 | ASP | P | 158 | -12.328 | -12.526 | -3.946 | 0.00 | 0.00 | PROA |
| ATOM | 2456 | HB2 | ASP | P | 158 | -13.085 | -13.359 | -5.332 | 0.00 | 0.00 | PROA |
| ATOM | 2457 | CG | ASP | P | 158 | -14.148 | -11.471 | -4.897 | 0.00 | 0.00 | PROA |
| ATOM | 2458 | OD1 | ASP | P | 158 | -15.143 | -11.984 | -5.423 | 0.00 | 0.00 | PROA |
| ATOM | 2459 | OD2 | ASP | P | 158 | -14.016 | -10.345 | -4.423 | 0.00 | 0.00 | PROA |
| ATOM | 2460 | C | ASP | P | 158 | -10.461 | -12.399 | -5.582 | 0.00 | 0.00 | PROA |
| ATOM | 2461 | O | ASP | P | 158 | -10.259 | -13.583 | -5.436 | 0.00 | 0.00 | PROA |
| ATOM | 2462 | N | TYR | P | 159 | -9.435 | -11.569 | -5.337 | 0.00 | 0.00 | PROA |
| ATOM | 2463 | HN | TYR | P | 159 | -9.715 | -10.613 | -5.378 | 0.00 | 0.00 | PROA |
| ATOM | 2464 | CA | TYR | P | 159 | -8.090 | -11.853 | -5.239 | 0.00 | 0.00 | PROA |
| ATOM | 2465 | HA | TYR | P | 159 | -7.828 | -12.801 | -5.687 | 0.00 | 0.00 | PROA |
| ATOM | 2466 | CB | TYR | P | 159 | -7.221 | -10.760 | -5.883 | 0.00 | 0.00 | PROA |
| ATOM | 2467 | HB1 | TYR | P | 159 | -6.223 | -11.245 | -5.931 | 0.00 | 0.00 | PROA |
| ATOM | 2468 | HB2 | TYR | P | 159 | -7.318 | -9.839 | -5.269 | 0.00 | 0.00 | PROA |
| ATOM | 2469 | CG | TYR | P | 159 | -7.506 | -10.391 | -7.253 | 0.00 | 0.00 | PROA |
| ATOM | 2470 | CD1 | TYR | P | 159 | -7.476 | -9.063 | -7.624 | 0.00 | 0.00 | PROA |
| ATOM | 2471 | HD1 | TYR | P | 159 | -7.345 | -8.322 | -6.850 | 0.00 | 0.00 | PROA |
| ATOM | 2472 | CE1 | TYR | P | 159 | -7.776 | -8.692 | -8.936 | 0.00 | 0.00 | PROA |
| ATOM | 2473 | HE1 | TYR | P | 159 | -7.782 | -7.657 | -9.246 | 0.00 | 0.00 | PROA |
| ATOM | 2474 | CZ | TYR | P | 159 | -8.181 | -9.600 | -9.894 | 0.00 | 0.00 | PROA |
| ATOM | 2475 | OH | TYR | P | 159 | -8.451 | -9.209 | -11.271 | 0.00 | 0.00 | PROA |
| ATOM | 2476 | HH | TYR | P | 159 | -8.995 | -9.882 | -11.687 | 0.00 | 0.00 | PROA |
| ATOM | 2477 | CD2 | TYR | P | 159 | -7.855 | -11.394 | -8.222 | 0.00 | 0.00 | PROA |
| ATOM | 2478 | HD2 | TYR | P | 159 | -7.800 | -12.428 | -7.915 | 0.00 | 0.00 | PROA |
| ATOM | 2479 | CE2 | TYR | P | 159 | -8.272 | -10.959 | -9.513 | 0.00 | 0.00 | PROA |
| ATOM | 2480 | HE2 | TYR | P | 159 | -8.574 | -11.654 | -10.283 | 0.00 | 0.00 | PROA |
| ATOM | 2481 | C | TYR | P | 159 | -7.639 | -12.020 | -3.796 | 0.00 | 0.00 | PROA |
| ATOM | 2482 | O | TYR | P | 159 | -8.253 | -11.678 | -2.778 | 0.00 | 0.00 | PROA |

| | | | | | | | | | | | |
|---|---|---|---|---|---|---|---|---|---|---|---|
| ATOM | 2483 | N | ILE | P | 160 | -6.458 | -12.685 | -3.659 | 0.00 | 0.00 | PROA |
| ATOM | 2484 | HN | ILE | P | 160 | -6.041 | -12.995 | -4.510 | 0.00 | 0.00 | PROA |
| ATOM | 2485 | CA | ILE | P | 160 | -5.752 | -12.880 | -2.377 | 0.00 | 0.00 | PROA |
| ATOM | 2486 | HA | ILE | P | 160 | -6.433 | -12.893 | -1.539 | 0.00 | 0.00 | PROA |
| ATOM | 2487 | CB | ILE | P | 160 | -4.910 | -14.113 | -2.348 | 0.00 | 0.00 | PROA |
| ATOM | 2488 | HB | ILE | P | 160 | -4.386 | -14.283 | -3.313 | 0.00 | 0.00 | PROA |
| ATOM | 2489 | CG2 | ILE | P | 160 | -4.007 | -14.287 | -1.061 | 0.00 | 0.00 | PROA |
| ATOM | 2490 | HG21 | ILE | P | 160 | -4.615 | -14.383 | -0.136 | 0.00 | 0.00 | PROA |
| ATOM | 2491 | HG22 | ILE | P | 160 | -3.157 | -13.575 | -0.992 | 0.00 | 0.00 | PROA |
| ATOM | 2492 | HG23 | ILE | P | 160 | -3.416 | -15.219 | -1.187 | 0.00 | 0.00 | PROA |
| ATOM | 2493 | CG1 | ILE | P | 160 | -5.859 | -15.307 | -2.544 | 0.00 | 0.00 | PROA |
| ATOM | 2494 | HG11 | ILE | P | 160 | -6.652 | -15.221 | -3.316 | 0.00 | 0.00 | PROA |
| ATOM | 2495 | HG12 | ILE | P | 160 | -6.459 | -15.665 | -1.680 | 0.00 | 0.00 | PROA |
| ATOM | 2496 | CD | ILE | P | 160 | -5.114 | -16.518 | -3.171 | 0.00 | 0.00 | PROA |
| ATOM | 2497 | HD1 | ILE | P | 160 | -4.856 | -16.312 | -4.232 | 0.00 | 0.00 | PROA |
| ATOM | 2498 | HD2 | ILE | P | 160 | -5.749 | -17.429 | -3.127 | 0.00 | 0.00 | PROA |
| ATOM | 2499 | HD3 | ILE | P | 160 | -4.168 | -16.781 | -2.652 | 0.00 | 0.00 | PROA |
| ATOM | 2500 | C | ILE | P | 160 | -4.821 | -11.675 | -2.241 | 0.00 | 0.00 | PROA |
| ATOM | 2501 | O | ILE | P | 160 | -4.219 | -11.249 | -3.227 | 0.00 | 0.00 | PROA |
| ATOM | 2502 | N | GLN | P | 161 | -4.683 | -11.010 | -1.108 | 0.00 | 0.00 | PROA |
| ATOM | 2503 | HN | GLN | P | 161 | -5.277 | -11.210 | -0.333 | 0.00 | 0.00 | PROA |
| ATOM | 2504 | CA | GLN | P | 161 | -4.117 | -9.726 | -1.067 | 0.00 | 0.00 | PROA |
| ATOM | 2505 | HA | GLN | P | 161 | -3.667 | -9.427 | -2.002 | 0.00 | 0.00 | PROA |
| ATOM | 2506 | CB | GLN | P | 161 | -5.016 | -8.548 | -0.532 | 0.00 | 0.00 | PROA |
| ATOM | 2507 | HB1 | GLN | P | 161 | -5.402 | -8.767 | 0.486 | 0.00 | 0.00 | PROA |
| ATOM | 2508 | HB2 | GLN | P | 161 | -5.817 | -8.456 | -1.296 | 0.00 | 0.00 | PROA |
| ATOM | 2509 | CG | GLN | P | 161 | -4.401 | -7.117 | -0.383 | 0.00 | 0.00 | PROA |
| ATOM | 2510 | HG1 | GLN | P | 161 | -3.697 | -6.931 | -1.223 | 0.00 | 0.00 | PROA |
| ATOM | 2511 | HG2 | GLN | P | 161 | -3.692 | -7.039 | 0.469 | 0.00 | 0.00 | PROA |
| ATOM | 2512 | CD | GLN | P | 161 | -5.384 | -5.934 | -0.306 | 0.00 | 0.00 | PROA |
| ATOM | 2513 | OE1 | GLN | P | 161 | -6.263 | -6.029 | 0.571 | 0.00 | 0.00 | PROA |
| ATOM | 2514 | NE2 | GLN | P | 161 | -5.193 | -4.927 | -1.155 | 0.00 | 0.00 | PROA |
| ATOM | 2515 | HE21 | GLN | P | 161 | -5.721 | -4.085 | -1.050 | 0.00 | 0.00 | PROA |
| ATOM | 2516 | HE22 | GLN | P | 161 | -4.454 | -4.974 | -1.827 | 0.00 | 0.00 | PROA |
| ATOM | 2517 | C | GLN | P | 161 | -2.967 | -9.809 | -0.036 | 0.00 | 0.00 | PROA |
| ATOM | 2518 | O | GLN | P | 161 | -3.245 | -9.851 | 1.173 | 0.00 | 0.00 | PROA |
| ATOM | 2519 | N | THR | P | 162 | -1.712 | -9.679 | -0.472 | 0.00 | 0.00 | PROA |
| ATOM | 2520 | HN | THR | P | 162 | -1.618 | -9.561 | -1.458 | 0.00 | 0.00 | PROA |
| ATOM | 2521 | CA | THR | P | 162 | -0.531 | -9.870 | 0.382 | 0.00 | 0.00 | PROA |
| ATOM | 2522 | HA | THR | P | 162 | -0.800 | -10.321 | 1.326 | 0.00 | 0.00 | PROA |
| ATOM | 2523 | CB | THR | P | 162 | 0.633 | -10.607 | -0.337 | 0.00 | 0.00 | PROA |
| ATOM | 2524 | HB | THR | P | 162 | 0.845 | -10.152 | -1.329 | 0.00 | 0.00 | PROA |
| ATOM | 2525 | OG1 | THR | P | 162 | 0.241 | -11.990 | -0.350 | 0.00 | 0.00 | PROA |
| ATOM | 2526 | HG1 | THR | P | 162 | -0.244 | -12.148 | -1.164 | 0.00 | 0.00 | PROA |
| ATOM | 2527 | CG2 | THR | P | 162 | 1.924 | -10.496 | 0.502 | 0.00 | 0.00 | PROA |
| ATOM | 2528 | HG21 | THR | P | 162 | 1.702 | -10.816 | 1.543 | 0.00 | 0.00 | PROA |
| ATOM | 2529 | HG22 | THR | P | 162 | 2.512 | -9.556 | 0.444 | 0.00 | 0.00 | PROA |
| ATOM | 2530 | HG23 | THR | P | 162 | 2.782 | -11.108 | 0.152 | 0.00 | 0.00 | PROA |
| ATOM | 2531 | C | THR | P | 162 | -0.053 | -8.466 | 0.589 | 0.00 | 0.00 | PROA |
| ATOM | 2532 | O | THR | P | 162 | 0.172 | -7.695 | -0.325 | 0.00 | 0.00 | PROA |
| ATOM | 2533 | N | ASP | P | 163 | 0.257 | -8.128 | 1.791 | 0.00 | 0.00 | PROA |
| ATOM | 2534 | HN | ASP | P | 163 | 0.248 | -8.759 | 2.563 | 0.00 | 0.00 | PROA |
| ATOM | 2535 | CA | ASP | P | 163 | 0.601 | -6.774 | 2.194 | 0.00 | 0.00 | PROA |
| ATOM | 2536 | HA | ASP | P | 163 | -0.117 | -6.165 | 1.665 | 0.00 | 0.00 | PROA |
| ATOM | 2537 | CB | ASP | P | 163 | 0.159 | -6.570 | 3.626 | 0.00 | 0.00 | PROA |
| ATOM | 2538 | HB1 | ASP | P | 163 | 0.812 | -7.217 | 4.251 | 0.00 | 0.00 | PROA |
| ATOM | 2539 | HB2 | ASP | P | 163 | -0.884 | -6.926 | 3.765 | 0.00 | 0.00 | PROA |
| ATOM | 2540 | CG | ASP | P | 163 | 0.115 | -5.115 | 4.042 | 0.00 | 0.00 | PROA |
| ATOM | 2541 | OD1 | ASP | P | 163 | 0.018 | -4.961 | 5.250 | 0.00 | 0.00 | PROA |
| ATOM | 2542 | OD2 | ASP | P | 163 | 0.265 | -4.188 | 3.202 | 0.00 | 0.00 | PROA |
| ATOM | 2543 | C | ASP | P | 163 | 1.994 | -6.351 | 1.860 | 0.00 | 0.00 | PROA |
| ATOM | 2544 | O | ASP | P | 163 | 2.900 | -6.148 | 2.737 | 0.00 | 0.00 | PROA |
| ATOM | 2545 | N | ALA | P | 164 | 2.204 | -6.155 | 0.537 | 0.00 | 0.00 | PROA |
| ATOM | 2546 | HN | ALA | P | 164 | 1.498 | -6.630 | 0.018 | 0.00 | 0.00 | PROA |
| ATOM | 2547 | CA | ALA | P | 164 | 3.480 | -5.780 | 0.046 | 0.00 | 0.00 | PROA |
| ATOM | 2548 | HA | ALA | P | 164 | 3.956 | -5.178 | 0.805 | 0.00 | 0.00 | PROA |
| ATOM | 2549 | CB | ALA | P | 164 | 4.449 | -6.957 | -0.102 | 0.00 | 0.00 | PROA |
| ATOM | 2550 | HB1 | ALA | P | 164 | 4.065 | -7.672 | -0.861 | 0.00 | 0.00 | PROA |
| ATOM | 2551 | HB2 | ALA | P | 164 | 4.516 | -7.541 | 0.841 | 0.00 | 0.00 | PROA |
| ATOM | 2552 | HB3 | ALA | P | 164 | 5.422 | -6.537 | -0.434 | 0.00 | 0.00 | PROA |
| ATOM | 2553 | C | ALA | P | 164 | 3.306 | -5.049 | -1.309 | 0.00 | 0.00 | PROA |
| ATOM | 2554 | O | ALA | P | 164 | 2.343 | -5.263 | -2.000 | 0.00 | 0.00 | PROA |
| ATOM | 2555 | N | ILE | P | 165 | 4.194 | -4.107 | -1.639 | 0.00 | 0.00 | PROA |

| | | | | | | | | | | | |
|---|---|---|---|---|---|---|---|---|---|---|---|
| ATOM | 2556 | HN | ILE | P | 165 | 4.848 | -3.838 | -0.936 | 0.00 | 0.00 | PROA |
| ATOM | 2557 | CA | ILE | P | 165 | 4.250 | -3.375 | -2.909 | 0.00 | 0.00 | PROA |
| ATOM | 2558 | HA | ILE | P | 165 | 3.226 | -3.207 | -3.208 | 0.00 | 0.00 | PROA |
| ATOM | 2559 | CB | ILE | P | 165 | 4.951 | -1.997 | -2.804 | 0.00 | 0.00 | PROA |
| ATOM | 2560 | HB | ILE | P | 165 | 5.955 | -2.012 | -2.328 | 0.00 | 0.00 | PROA |
| ATOM | 2561 | CG2 | ILE | P | 165 | 4.984 | -1.168 | -4.133 | 0.00 | 0.00 | PROA |
| ATOM | 2562 | HG21 | ILE | P | 165 | 5.780 | -1.628 | -4.758 | 0.00 | 0.00 | PROA |
| ATOM | 2563 | HG22 | ILE | P | 165 | 5.149 | -0.108 | -3.844 | 0.00 | 0.00 | PROA |
| ATOM | 2564 | HG23 | ILE | P | 165 | 4.010 | -1.297 | -4.651 | 0.00 | 0.00 | PROA |
| ATOM | 2565 | CG1 | ILE | P | 165 | 4.155 | -1.134 | -1.746 | 0.00 | 0.00 | PROA |
| ATOM | 2566 | HG11 | ILE | P | 165 | 4.684 | -0.158 | -1.694 | 0.00 | 0.00 | PROA |
| ATOM | 2567 | HG12 | ILE | P | 165 | 4.118 | -1.662 | -0.769 | 0.00 | 0.00 | PROA |
| ATOM | 2568 | CD | ILE | P | 165 | 2.711 | -0.902 | -2.090 | 0.00 | 0.00 | PROA |
| ATOM | 2569 | HD1 | ILE | P | 165 | 2.117 | -1.793 | -2.386 | 0.00 | 0.00 | PROA |
| ATOM | 2570 | HD2 | ILE | P | 165 | 2.657 | -0.113 | -2.872 | 0.00 | 0.00 | PROA |
| ATOM | 2571 | HD3 | ILE | P | 165 | 2.117 | -0.394 | -1.301 | 0.00 | 0.00 | PROA |
| ATOM | 2572 | C | ILE | P | 165 | 5.025 | -4.163 | -3.992 | 0.00 | 0.00 | PROA |
| ATOM | 2573 | O | ILE | P | 165 | 6.157 | -4.605 | -3.885 | 0.00 | 0.00 | PROA |
| ATOM | 2574 | N | ILE | P | 166 | 4.444 | -4.373 | -5.184 | 0.00 | 0.00 | PROA |
| ATOM | 2575 | HN | ILE | P | 166 | 3.473 | -4.158 | -5.263 | 0.00 | 0.00 | PROA |
| ATOM | 2576 | CA | ILE | P | 166 | 5.031 | -5.169 | -6.294 | 0.00 | 0.00 | PROA |
| ATOM | 2577 | HA | ILE | P | 166 | 5.176 | -6.145 | -5.854 | 0.00 | 0.00 | PROA |
| ATOM | 2578 | CB | ILE | P | 166 | 4.018 | -5.280 | -7.457 | 0.00 | 0.00 | PROA |
| ATOM | 2579 | HB | ILE | P | 166 | 4.341 | -5.981 | -8.257 | 0.00 | 0.00 | PROA |
| ATOM | 2580 | CG2 | ILE | P | 166 | 2.626 | -5.771 | -6.919 | 0.00 | 0.00 | PROA |
| ATOM | 2581 | HG21 | ILE | P | 166 | 2.093 | -5.030 | -6.285 | 0.00 | 0.00 | PROA |
| ATOM | 2582 | HG22 | ILE | P | 166 | 2.727 | -6.744 | -6.392 | 0.00 | 0.00 | PROA |
| ATOM | 2583 | HG23 | ILE | P | 166 | 1.941 | -5.987 | -7.766 | 0.00 | 0.00 | PROA |
| ATOM | 2584 | CG1 | ILE | P | 166 | 3.782 | -3.907 | -8.124 | 0.00 | 0.00 | PROA |
| ATOM | 2585 | HG11 | ILE | P | 166 | 4.784 | -3.496 | -8.370 | 0.00 | 0.00 | PROA |
| ATOM | 2586 | HG12 | ILE | P | 166 | 3.159 | -3.172 | -7.570 | 0.00 | 0.00 | PROA |
| ATOM | 2587 | CD | ILE | P | 166 | 3.191 | -4.179 | -9.515 | 0.00 | 0.00 | PROA |
| ATOM | 2588 | HD1 | ILE | P | 166 | 3.127 | -3.234 | -10.096 | 0.00 | 0.00 | PROA |
| ATOM | 2589 | HD2 | ILE | P | 166 | 2.229 | -4.723 | -9.398 | 0.00 | 0.00 | PROA |
| ATOM | 2590 | HD3 | ILE | P | 166 | 3.815 | -4.934 | -10.040 | 0.00 | 0.00 | PROA |
| ATOM | 2591 | C | ILE | P | 166 | 6.434 | -4.885 | -6.845 | 0.00 | 0.00 | PROA |
| ATOM | 2592 | O | ILE | P | 166 | 6.988 | -3.767 | -6.816 | 0.00 | 0.00 | PROA |
| ATOM | 2593 | N | ASN | P | 167 | 7.143 | -6.001 | -7.234 | 0.00 | 0.00 | PROA |
| ATOM | 2594 | HN | ASN | P | 167 | 6.781 | -6.862 | -6.886 | 0.00 | 0.00 | PROA |
| ATOM | 2595 | CA | ASN | P | 167 | 8.347 | -6.069 | -8.004 | 0.00 | 0.00 | PROA |
| ATOM | 2596 | HA | ASN | P | 167 | 8.855 | -5.120 | -7.909 | 0.00 | 0.00 | PROA |
| ATOM | 2597 | CB | ASN | P | 167 | 9.330 | -7.160 | -7.457 | 0.00 | 0.00 | PROA |
| ATOM | 2598 | HB1 | ASN | P | 167 | 10.192 | -7.278 | -8.148 | 0.00 | 0.00 | PROA |
| ATOM | 2599 | HB2 | ASN | P | 167 | 8.940 | -8.178 | -7.243 | 0.00 | 0.00 | PROA |
| ATOM | 2600 | CG | ASN | P | 167 | 9.979 | -6.679 | -6.210 | 0.00 | 0.00 | PROA |
| ATOM | 2601 | OD1 | ASN | P | 167 | 11.193 | -6.359 | -6.163 | 0.00 | 0.00 | PROA |
| ATOM | 2602 | ND2 | ASN | P | 167 | 9.181 | -6.622 | -5.133 | 0.00 | 0.00 | PROA |
| ATOM | 2603 | HD21 | ASN | P | 167 | 8.305 | -7.105 | -5.127 | 0.00 | 0.00 | PROA |
| ATOM | 2604 | HD22 | ASN | P | 167 | 9.538 | -6.418 | -4.221 | 0.00 | 0.00 | PROA |
| ATOM | 2605 | C | ASN | P | 167 | 8.067 | -6.442 | -9.428 | 0.00 | 0.00 | PROA |
| ATOM | 2606 | O | ASN | P | 167 | 7.175 | -7.259 | -9.673 | 0.00 | 0.00 | PROA |
| ATOM | 2607 | N | TYR | P | 168 | 8.862 | -5.931 | -10.394 | 0.00 | 0.00 | PROA |
| ATOM | 2608 | HN | TYR | P | 168 | 9.606 | -5.294 | -10.206 | 0.00 | 0.00 | PROA |
| ATOM | 2609 | CA | TYR | P | 168 | 8.652 | -6.038 | -11.847 | 0.00 | 0.00 | PROA |
| ATOM | 2610 | HA | TYR | P | 168 | 7.664 | -5.680 | -12.094 | 0.00 | 0.00 | PROA |
| ATOM | 2611 | CB | TYR | P | 168 | 9.719 | -5.227 | -12.609 | 0.00 | 0.00 | PROA |
| ATOM | 2612 | HB1 | TYR | P | 168 | 10.798 | -5.428 | -12.437 | 0.00 | 0.00 | PROA |
| ATOM | 2613 | HB2 | TYR | P | 168 | 9.412 | -4.237 | -12.209 | 0.00 | 0.00 | PROA |
| ATOM | 2614 | CG | TYR | P | 168 | 9.601 | -5.137 | -14.115 | 0.00 | 0.00 | PROA |
| ATOM | 2615 | CD1 | TYR | P | 168 | 10.323 | -6.057 | -14.853 | 0.00 | 0.00 | PROA |
| ATOM | 2616 | HD1 | TYR | P | 168 | 10.952 | -6.756 | -14.323 | 0.00 | 0.00 | PROA |
| ATOM | 2617 | CE1 | TYR | P | 168 | 10.293 | -6.009 | -16.277 | 0.00 | 0.00 | PROA |
| ATOM | 2618 | HE1 | TYR | P | 168 | 10.916 | -6.716 | -16.803 | 0.00 | 0.00 | PROA |
| ATOM | 2619 | CZ | TYR | P | 168 | 9.623 | -4.992 | -16.904 | 0.00 | 0.00 | PROA |
| ATOM | 2620 | OH | TYR | P | 168 | 9.643 | -4.900 | -18.404 | 0.00 | 0.00 | PROA |
| ATOM | 2621 | HH | TYR | P | 168 | 9.878 | -5.770 | -18.735 | 0.00 | 0.00 | PROA |
| ATOM | 2622 | CD2 | TYR | P | 168 | 8.804 | -4.159 | -14.746 | 0.00 | 0.00 | PROA |
| ATOM | 2623 | HD2 | TYR | P | 168 | 8.176 | -3.465 | -14.207 | 0.00 | 0.00 | PROA |
| ATOM | 2624 | CE2 | TYR | P | 168 | 8.823 | -4.125 | -16.130 | 0.00 | 0.00 | PROA |
| ATOM | 2625 | HE2 | TYR | P | 168 | 8.342 | -3.229 | -16.493 | 0.00 | 0.00 | PROA |
| ATOM | 2626 | C | TYR | P | 168 | 8.683 | -7.477 | -12.302 | 0.00 | 0.00 | PROA |
| ATOM | 2627 | O | TYR | P | 168 | 9.559 | -8.269 | -11.922 | 0.00 | 0.00 | PROA |
| ATOM | 2628 | N | GLY | P | 169 | 7.746 | -7.929 | -13.140 | 0.00 | 0.00 | PROA |

```
ATOM   2629  HN  GLY P 169       6.991  -7.357 -13.452  0.00  0.00      PROA
ATOM   2630  CA  GLY P 169       7.809  -9.298 -13.745  0.00  0.00      PROA
ATOM   2631  HA1 GLY P 169       8.732  -9.442 -14.288  0.00  0.00      PROA
ATOM   2632  HA2 GLY P 169       7.003  -9.322 -14.463  0.00  0.00      PROA
ATOM   2633  C   GLY P 169       7.468 -10.400 -12.809  0.00  0.00      PROA
ATOM   2634  O   GLY P 169       7.771 -11.577 -12.955  0.00  0.00      PROA
ATOM   2635  N   ASN P 170       6.771 -10.091 -11.749  0.00  0.00      PROA
ATOM   2636  HN  ASN P 170       6.389  -9.172 -11.692  0.00  0.00      PROA
ATOM   2637  CA  ASN P 170       6.238 -11.104 -10.903  0.00  0.00      PROA
ATOM   2638  HA  ASN P 170       6.920 -11.927 -10.755  0.00  0.00      PROA
ATOM   2639  CB  ASN P 170       6.125 -10.445  -9.466  0.00  0.00      PROA
ATOM   2640  HB1 ASN P 170       5.411 -11.040  -8.859  0.00  0.00      PROA
ATOM   2641  HB2 ASN P 170       5.595  -9.478  -9.602  0.00  0.00      PROA
ATOM   2642  CG  ASN P 170       7.410 -10.281  -8.579  0.00  0.00      PROA
ATOM   2643  OD1 ASN P 170       7.314 -10.159  -7.329  0.00  0.00      PROA
ATOM   2644  ND2 ASN P 170       8.659 -10.467  -9.102  0.00  0.00      PROA
ATOM   2645 HD21 ASN P 170       8.592 -10.427 -10.099  0.00  0.00      PROA
ATOM   2646 HD22 ASN P 170       9.420 -10.301  -8.476  0.00  0.00      PROA
ATOM   2647  C   ASN P 170       4.920 -11.649 -11.362  0.00  0.00      PROA
ATOM   2648  O   ASN P 170       4.561 -12.807 -11.153  0.00  0.00      PROA
ATOM   2649  N   SER P 171       4.109 -10.863 -12.105  0.00  0.00      PROA
ATOM   2650  HN  SER P 171       4.319  -9.891 -12.174  0.00  0.00      PROA
ATOM   2651  CA  SER P 171       2.746 -11.213 -12.598  0.00  0.00      PROA
ATOM   2652  HA  SER P 171       2.152 -11.445 -11.726  0.00  0.00      PROA
ATOM   2653  CB  SER P 171       2.043 -10.021 -13.231  0.00  0.00      PROA
ATOM   2654  HB1 SER P 171       2.520  -9.794 -14.208  0.00  0.00      PROA
ATOM   2655  HB2 SER P 171       2.240  -9.169 -12.546  0.00  0.00      PROA
ATOM   2656  OG  SER P 171       0.691 -10.279 -13.421  0.00  0.00      PROA
ATOM   2657  HG1 SER P 171       0.589 -10.984 -14.064  0.00  0.00      PROA
ATOM   2658  C   SER P 171       2.817 -12.424 -13.616  0.00  0.00      PROA
ATOM   2659  O   SER P 171       3.725 -12.587 -14.466  0.00  0.00      PROA
ATOM   2660  N   GLY P 172       1.793 -13.315 -13.505  0.00  0.00      PROA
ATOM   2661  HN  GLY P 172       0.947 -13.135 -13.009  0.00  0.00      PROA
ATOM   2662  CA  GLY P 172       1.864 -14.592 -14.250  0.00  0.00      PROA
ATOM   2663  HA1 GLY P 172       2.494 -14.554 -15.126  0.00  0.00      PROA
ATOM   2664  HA2 GLY P 172       0.839 -14.777 -14.535  0.00  0.00      PROA
ATOM   2665  C   GLY P 172       2.386 -15.731 -13.393  0.00  0.00      PROA
ATOM   2666  O   GLY P 172       1.896 -16.875 -13.367  0.00  0.00      PROA
ATOM   2667  N   GLY P 173       3.424 -15.466 -12.555  0.00  0.00      PROA
ATOM   2668  HN  GLY P 173       3.896 -14.590 -12.617  0.00  0.00      PROA
ATOM   2669  CA  GLY P 173       4.030 -16.494 -11.673  0.00  0.00      PROA
ATOM   2670  HA1 GLY P 173       4.883 -15.962 -11.280  0.00  0.00      PROA
ATOM   2671  HA2 GLY P 173       4.149 -17.465 -12.130  0.00  0.00      PROA
ATOM   2672  C   GLY P 173       3.226 -16.799 -10.448  0.00  0.00      PROA
ATOM   2673  O   GLY P 173       2.167 -16.257 -10.222  0.00  0.00      PROA
ATOM   2674  N   PRO P 174       3.738 -17.770  -9.731  0.00  0.00      PROA
ATOM   2675  CD  PRO P 174       5.006 -18.480  -9.935  0.00  0.00      PROA
ATOM   2676  HD1 PRO P 174       5.899 -17.830  -9.810  0.00  0.00      PROA
ATOM   2677  HD2 PRO P 174       5.042 -19.059 -10.882  0.00  0.00      PROA
ATOM   2678  CA  PRO P 174       3.072 -18.180  -8.520  0.00  0.00      PROA
ATOM   2679  HA  PRO P 174       2.025 -18.261  -8.769  0.00  0.00      PROA
ATOM   2680  CB  PRO P 174       3.662 -19.597  -8.329  0.00  0.00      PROA
ATOM   2681  HB1 PRO P 174       3.305 -20.302  -9.109  0.00  0.00      PROA
ATOM   2682  HB2 PRO P 174       3.642 -19.960  -7.279  0.00  0.00      PROA
ATOM   2683  CG  PRO P 174       5.108 -19.478  -8.743  0.00  0.00      PROA
ATOM   2684  HG1 PRO P 174       5.712 -18.907  -8.006  0.00  0.00      PROA
ATOM   2685  HG2 PRO P 174       5.552 -20.444  -9.067  0.00  0.00      PROA
ATOM   2686  C   PRO P 174       3.295 -17.387  -7.220  0.00  0.00      PROA
ATOM   2687  O   PRO P 174       4.378 -16.911  -7.061  0.00  0.00      PROA
ATOM   2688  N   LEU P 175       2.275 -17.408  -6.381  0.00  0.00      PROA
ATOM   2689  HN  LEU P 175       1.409 -17.831  -6.636  0.00  0.00      PROA
ATOM   2690  CA  LEU P 175       2.320 -17.066  -5.032  0.00  0.00      PROA
ATOM   2691  HA  LEU P 175       3.155 -16.406  -4.850  0.00  0.00      PROA
ATOM   2692  CB  LEU P 175       0.999 -16.329  -4.463  0.00  0.00      PROA
ATOM   2693  HB1 LEU P 175       0.121 -16.981  -4.660  0.00  0.00      PROA
ATOM   2694  HB2 LEU P 175       0.864 -15.491  -5.180  0.00  0.00      PROA
ATOM   2695  CG  LEU P 175       0.980 -15.851  -2.990  0.00  0.00      PROA
ATOM   2696  HG  LEU P 175       1.335 -16.729  -2.411  0.00  0.00      PROA
ATOM   2697  CD1 LEU P 175       1.907 -14.672  -2.779  0.00  0.00      PROA
ATOM   2698 HD11 LEU P 175       2.288 -14.557  -1.742  0.00  0.00      PROA
ATOM   2699 HD12 LEU P 175       1.365 -13.753  -3.088  0.00  0.00      PROA
ATOM   2700 HD13 LEU P 175       2.696 -14.707  -3.561  0.00  0.00      PROA
ATOM   2701  CD2 LEU P 175      -0.437 -15.572  -2.406  0.00  0.00      PROA
```

| | | | | | | | | | | | |
|---|---|---|---|---|---|---|---|---|---|---|---|
| ATOM | 2702 | HD21 | LEU | P | 175 | -1.128 | -16.290 | -2.897 | 0.00 | 0.00 | PROA |
| ATOM | 2703 | HD22 | LEU | P | 175 | -0.687 | -14.562 | -2.798 | 0.00 | 0.00 | PROA |
| ATOM | 2704 | HD23 | LEU | P | 175 | -0.581 | -15.543 | -1.305 | 0.00 | 0.00 | PROA |
| ATOM | 2705 | C | LEU | P | 175 | 2.448 | -18.330 | -4.224 | 0.00 | 0.00 | PROA |
| ATOM | 2706 | O | LEU | P | 175 | 1.710 | -19.291 | -4.361 | 0.00 | 0.00 | PROA |
| ATOM | 2707 | N | VAL | P | 176 | 3.509 | -18.415 | -3.369 | 0.00 | 0.00 | PROA |
| ATOM | 2708 | HN | VAL | P | 176 | 4.165 | -17.682 | -3.207 | 0.00 | 0.00 | PROA |
| ATOM | 2709 | CA | VAL | P | 176 | 3.894 | -19.718 | -2.830 | 0.00 | 0.00 | PROA |
| ATOM | 2710 | HA | VAL | P | 176 | 3.231 | -20.493 | -3.186 | 0.00 | 0.00 | PROA |
| ATOM | 2711 | CB | VAL | P | 176 | 5.297 | -20.104 | -3.341 | 0.00 | 0.00 | PROA |
| ATOM | 2712 | HB | VAL | P | 176 | 6.128 | -19.452 | -2.996 | 0.00 | 0.00 | PROA |
| ATOM | 2713 | CG1 | VAL | P | 176 | 5.637 | -21.599 | -3.040 | 0.00 | 0.00 | PROA |
| ATOM | 2714 | HG11 | VAL | P | 176 | 6.531 | -21.927 | -3.612 | 0.00 | 0.00 | PROA |
| ATOM | 2715 | HG12 | VAL | P | 176 | 4.771 | -22.269 | -3.228 | 0.00 | 0.00 | PROA |
| ATOM | 2716 | HG13 | VAL | P | 176 | 5.746 | -21.599 | -1.935 | 0.00 | 0.00 | PROA |
| ATOM | 2717 | CG2 | VAL | P | 176 | 5.328 | -19.806 | -4.913 | 0.00 | 0.00 | PROA |
| ATOM | 2718 | HG21 | VAL | P | 176 | 4.324 | -20.134 | -5.257 | 0.00 | 0.00 | PROA |
| ATOM | 2719 | HG22 | VAL | P | 176 | 6.106 | -20.433 | -5.399 | 0.00 | 0.00 | PROA |
| ATOM | 2720 | HG23 | VAL | P | 176 | 5.351 | -18.741 | -5.230 | 0.00 | 0.00 | PROA |
| ATOM | 2721 | C | VAL | P | 176 | 3.911 | -19.596 | -1.302 | 0.00 | 0.00 | PROA |
| ATOM | 2722 | O | VAL | P | 176 | 4.362 | -18.599 | -0.721 | 0.00 | 0.00 | PROA |
| ATOM | 2723 | N | ASN | P | 177 | 3.433 | -20.584 | -0.593 | 0.00 | 0.00 | PROA |
| ATOM | 2724 | HN | ASN | P | 177 | 3.049 | -21.380 | -1.054 | 0.00 | 0.00 | PROA |
| ATOM | 2725 | CA | ASN | P | 177 | 3.540 | -20.503 | 0.837 | 0.00 | 0.00 | PROA |
| ATOM | 2726 | HA | ASN | P | 177 | 3.397 | -19.489 | 1.181 | 0.00 | 0.00 | PROA |
| ATOM | 2727 | CB | ASN | P | 177 | 2.381 | -21.291 | 1.576 | 0.00 | 0.00 | PROA |
| ATOM | 2728 | HB1 | ASN | P | 177 | 1.475 | -20.858 | 1.102 | 0.00 | 0.00 | PROA |
| ATOM | 2729 | HB2 | ASN | P | 177 | 2.363 | -21.168 | 2.680 | 0.00 | 0.00 | PROA |
| ATOM | 2730 | CG | ASN | P | 177 | 2.395 | -22.776 | 1.235 | 0.00 | 0.00 | PROA |
| ATOM | 2731 | OD1 | ASN | P | 177 | 3.191 | -23.359 | 0.543 | 0.00 | 0.00 | PROA |
| ATOM | 2732 | ND2 | ASN | P | 177 | 1.371 | -23.486 | 1.690 | 0.00 | 0.00 | PROA |
| ATOM | 2733 | HD21 | ASN | P | 177 | 1.374 | -24.465 | 1.485 | 0.00 | 0.00 | PROA |
| ATOM | 2734 | HD22 | ASN | P | 177 | 0.636 | -22.988 | 2.149 | 0.00 | 0.00 | PROA |
| ATOM | 2735 | C | ASN | P | 177 | 4.909 | -21.067 | 1.281 | 0.00 | 0.00 | PROA |
| ATOM | 2736 | O | ASN | P | 177 | 5.683 | -21.506 | 0.443 | 0.00 | 0.00 | PROA |
| ATOM | 2737 | N | LEU | P | 178 | 5.286 | -21.024 | 2.575 | 0.00 | 0.00 | PROA |
| ATOM | 2738 | HN | LEU | P | 178 | 4.858 | -20.650 | 3.395 | 0.00 | 0.00 | PROA |
| ATOM | 2739 | CA | LEU | P | 178 | 6.590 | -21.497 | 2.937 | 0.00 | 0.00 | PROA |
| ATOM | 2740 | HA | LEU | P | 178 | 7.330 | -21.198 | 2.209 | 0.00 | 0.00 | PROA |
| ATOM | 2741 | CB | LEU | P | 178 | 6.918 | -20.730 | 4.262 | 0.00 | 0.00 | PROA |
| ATOM | 2742 | HB1 | LEU | P | 178 | 7.847 | -21.135 | 4.717 | 0.00 | 0.00 | PROA |
| ATOM | 2743 | HB2 | LEU | P | 178 | 6.089 | -20.833 | 4.995 | 0.00 | 0.00 | PROA |
| ATOM | 2744 | CG | LEU | P | 178 | 7.296 | -19.238 | 4.039 | 0.00 | 0.00 | PROA |
| ATOM | 2745 | HG | LEU | P | 178 | 6.498 | -18.700 | 3.485 | 0.00 | 0.00 | PROA |
| ATOM | 2746 | CD1 | LEU | P | 178 | 7.561 | -18.563 | 5.396 | 0.00 | 0.00 | PROA |
| ATOM | 2747 | HD11 | LEU | P | 178 | 8.372 | -17.811 | 5.495 | 0.00 | 0.00 | PROA |
| ATOM | 2748 | HD12 | LEU | P | 178 | 7.849 | -19.354 | 6.121 | 0.00 | 0.00 | PROA |
| ATOM | 2749 | HD13 | LEU | P | 178 | 6.609 | -18.061 | 5.675 | 0.00 | 0.00 | PROA |
| ATOM | 2750 | CD2 | LEU | P | 178 | 8.595 | -19.010 | 3.219 | 0.00 | 0.00 | PROA |
| ATOM | 2751 | HD21 | LEU | P | 178 | 8.965 | -17.990 | 3.461 | 0.00 | 0.00 | PROA |
| ATOM | 2752 | HD22 | LEU | P | 178 | 8.516 | -19.091 | 2.113 | 0.00 | 0.00 | PROA |
| ATOM | 2753 | HD23 | LEU | P | 178 | 9.391 | -19.706 | 3.561 | 0.00 | 0.00 | PROA |
| ATOM | 2754 | C | LEU | P | 178 | 6.659 | -22.997 | 3.205 | 0.00 | 0.00 | PROA |
| ATOM | 2755 | O | LEU | P | 178 | 7.633 | -23.565 | 3.804 | 0.00 | 0.00 | PROA |
| ATOM | 2756 | N | ASP | P | 179 | 5.611 | -23.721 | 2.785 | 0.00 | 0.00 | PROA |
| ATOM | 2757 | HN | ASP | P | 179 | 4.872 | -23.150 | 2.435 | 0.00 | 0.00 | PROA |
| ATOM | 2758 | CA | ASP | P | 179 | 5.673 | -25.118 | 2.679 | 0.00 | 0.00 | PROA |
| ATOM | 2759 | HA | ASP | P | 179 | 6.296 | -25.585 | 3.428 | 0.00 | 0.00 | PROA |
| ATOM | 2760 | CB | ASP | P | 179 | 4.247 | -25.735 | 2.893 | 0.00 | 0.00 | PROA |
| ATOM | 2761 | HB1 | ASP | P | 179 | 4.189 | -26.795 | 2.563 | 0.00 | 0.00 | PROA |
| ATOM | 2762 | HB2 | ASP | P | 179 | 3.625 | -25.121 | 2.207 | 0.00 | 0.00 | PROA |
| ATOM | 2763 | CG | ASP | P | 179 | 3.867 | -25.558 | 4.324 | 0.00 | 0.00 | PROA |
| ATOM | 2764 | OD1 | ASP | P | 179 | 2.653 | -25.666 | 4.727 | 0.00 | 0.00 | PROA |
| ATOM | 2765 | OD2 | ASP | P | 179 | 4.737 | -25.311 | 5.239 | 0.00 | 0.00 | PROA |
| ATOM | 2766 | C | ASP | P | 179 | 6.174 | -25.446 | 1.257 | 0.00 | 0.00 | PROA |
| ATOM | 2767 | O | ASP | P | 179 | 6.974 | -26.351 | 1.166 | 0.00 | 0.00 | PROA |
| ATOM | 2768 | N | GLY | P | 180 | 5.751 | -24.727 | 0.166 | 0.00 | 0.00 | PROA |
| ATOM | 2769 | HN | GLY | P | 180 | 5.073 | -24.020 | 0.354 | 0.00 | 0.00 | PROA |
| ATOM | 2770 | CA | GLY | P | 180 | 6.289 | -24.809 | -1.222 | 0.00 | 0.00 | PROA |
| ATOM | 2771 | HA1 | GLY | P | 180 | 6.991 | -25.627 | -1.289 | 0.00 | 0.00 | PROA |
| ATOM | 2772 | HA2 | GLY | P | 180 | 6.659 | -23.820 | -1.446 | 0.00 | 0.00 | PROA |
| ATOM | 2773 | C | GLY | P | 180 | 5.127 | -25.035 | -2.148 | 0.00 | 0.00 | PROA |
| ATOM | 2774 | O | GLY | P | 180 | 5.255 | -25.400 | -3.294 | 0.00 | 0.00 | PROA |

```
ATOM   2775  N   GLU P 181       3.913 -24.821  -1.730  0.00  0.00      PROA
ATOM   2776  HN  GLU P 181       3.698 -24.504  -0.810  0.00  0.00      PROA
ATOM   2777  CA  GLU P 181       2.707 -25.126  -2.442  0.00  0.00      PROA
ATOM   2778  HA  GLU P 181       2.875 -25.934  -3.139  0.00  0.00      PROA
ATOM   2779  CB  GLU P 181       1.654 -25.652  -1.452  0.00  0.00      PROA
ATOM   2780  HB1 GLU P 181       0.726 -25.942  -1.989  0.00  0.00      PROA
ATOM   2781  HB2 GLU P 181       1.372 -24.747  -0.872  0.00  0.00      PROA
ATOM   2782  CG  GLU P 181       2.119 -26.761  -0.512  0.00  0.00      PROA
ATOM   2783  HG1 GLU P 181       2.931 -26.286   0.079  0.00  0.00      PROA
ATOM   2784  HG2 GLU P 181       2.520 -27.672  -1.005  0.00  0.00      PROA
ATOM   2785  CD  GLU P 181       1.019 -27.178   0.382  0.00  0.00      PROA
ATOM   2786  OE1 GLU P 181       0.509 -28.253   0.109  0.00  0.00      PROA
ATOM   2787  OE2 GLU P 181       0.687 -26.402   1.276  0.00  0.00      PROA
ATOM   2788  C   GLU P 181       2.234 -23.802  -3.025  0.00  0.00      PROA
ATOM   2789  O   GLU P 181       2.205 -22.677  -2.434  0.00  0.00      PROA
ATOM   2790  N   VAL P 182       1.825 -23.856  -4.304  0.00  0.00      PROA
ATOM   2791  HN  VAL P 182       1.881 -24.781  -4.672  0.00  0.00      PROA
ATOM   2792  CA  VAL P 182       1.368 -22.760  -5.099  0.00  0.00      PROA
ATOM   2793  HA  VAL P 182       1.976 -21.896  -4.874  0.00  0.00      PROA
ATOM   2794  CB  VAL P 182       1.415 -23.008  -6.556  0.00  0.00      PROA
ATOM   2795  HB  VAL P 182       1.143 -24.067  -6.750  0.00  0.00      PROA
ATOM   2796  CG1 VAL P 182       0.678 -22.057  -7.432  0.00  0.00      PROA
ATOM   2797 HG11 VAL P 182      -0.389 -21.823  -7.225  0.00  0.00      PROA
ATOM   2798 HG12 VAL P 182       0.739 -22.255  -8.523  0.00  0.00      PROA
ATOM   2799 HG13 VAL P 182       1.090 -21.027  -7.377  0.00  0.00      PROA
ATOM   2800  CG2 VAL P 182       2.913 -22.978  -6.976  0.00  0.00      PROA
ATOM   2801 HG21 VAL P 182       3.405 -22.063  -6.582  0.00  0.00      PROA
ATOM   2802 HG22 VAL P 182       3.077 -23.231  -8.045  0.00  0.00      PROA
ATOM   2803 HG23 VAL P 182       3.472 -23.762  -6.423  0.00  0.00      PROA
ATOM   2804  C   VAL P 182      -0.038 -22.431  -4.593  0.00  0.00      PROA
ATOM   2805  O   VAL P 182      -0.976 -23.134  -4.882  0.00  0.00      PROA
ATOM   2806  N   ILE P 183      -0.264 -21.240  -4.005  0.00  0.00      PROA
ATOM   2807  HN  ILE P 183       0.533 -20.804  -3.594  0.00  0.00      PROA
ATOM   2808  CA  ILE P 183      -1.503 -20.817  -3.332  0.00  0.00      PROA
ATOM   2809  HA  ILE P 183      -2.173 -21.663  -3.281  0.00  0.00      PROA
ATOM   2810  CB  ILE P 183      -1.268 -20.468  -1.853  0.00  0.00      PROA
ATOM   2811  HB  ILE P 183      -2.231 -20.046  -1.495  0.00  0.00      PROA
ATOM   2812  CG2 ILE P 183      -1.216 -21.794  -1.027  0.00  0.00      PROA
ATOM   2813 HG21 ILE P 183      -0.452 -22.527  -1.363  0.00  0.00      PROA
ATOM   2814 HG22 ILE P 183      -2.207 -22.296  -1.043  0.00  0.00      PROA
ATOM   2815 HG23 ILE P 183      -0.982 -21.703   0.055  0.00  0.00      PROA
ATOM   2816  CG1 ILE P 183      -0.020 -19.582  -1.663  0.00  0.00      PROA
ATOM   2817 HG11 ILE P 183       0.306 -19.037  -2.575  0.00  0.00      PROA
ATOM   2818 HG12 ILE P 183       0.886 -20.214  -1.540  0.00  0.00      PROA
ATOM   2819  CD  ILE P 183      -0.141 -18.698  -0.467  0.00  0.00      PROA
ATOM   2820  HD1 ILE P 183       0.818 -18.249  -0.133  0.00  0.00      PROA
ATOM   2821  HD2 ILE P 183      -0.536 -19.185   0.450  0.00  0.00      PROA
ATOM   2822  HD3 ILE P 183      -0.823 -17.868  -0.750  0.00  0.00      PROA
ATOM   2823  C   ILE P 183      -2.142 -19.696  -4.137  0.00  0.00      PROA
ATOM   2824  O   ILE P 183      -3.240 -19.311  -3.865  0.00  0.00      PROA
ATOM   2825  N   GLY P 184      -1.494 -19.212  -5.205  0.00  0.00      PROA
ATOM   2826  HN  GLY P 184      -0.609 -19.648  -5.347  0.00  0.00      PROA
ATOM   2827  CA  GLY P 184      -2.124 -18.294  -6.105  0.00  0.00      PROA
ATOM   2828  HA1 GLY P 184      -2.126 -17.301  -5.678  0.00  0.00      PROA
ATOM   2829  HA2 GLY P 184      -2.988 -18.807  -6.499  0.00  0.00      PROA
ATOM   2830  C   GLY P 184      -1.224 -18.157  -7.227  0.00  0.00      PROA
ATOM   2831  O   GLY P 184      -0.147 -18.693  -7.355  0.00  0.00      PROA
ATOM   2832  N   ILE P 185      -1.695 -17.431  -8.227  0.00  0.00      PROA
ATOM   2833  HN  ILE P 185      -2.570 -16.971  -8.099  0.00  0.00      PROA
ATOM   2834  CA  ILE P 185      -1.092 -16.940  -9.424  0.00  0.00      PROA
ATOM   2835  HA  ILE P 185      -0.044 -17.176  -9.313  0.00  0.00      PROA
ATOM   2836  CB  ILE P 185      -1.698 -17.597 -10.695  0.00  0.00      PROA
ATOM   2837  HB  ILE P 185      -1.455 -18.681 -10.662  0.00  0.00      PROA
ATOM   2838  CG2 ILE P 185      -3.184 -17.475 -10.661  0.00  0.00      PROA
ATOM   2839 HG21 ILE P 185      -3.691 -18.115 -11.415  0.00  0.00      PROA
ATOM   2840 HG22 ILE P 185      -3.446 -16.417 -10.876  0.00  0.00      PROA
ATOM   2841 HG23 ILE P 185      -3.716 -17.874  -9.771  0.00  0.00      PROA
ATOM   2842  CG1 ILE P 185      -1.180 -17.121 -12.063  0.00  0.00      PROA
ATOM   2843 HG11 ILE P 185      -0.083 -17.254 -11.948  0.00  0.00      PROA
ATOM   2844 HG12 ILE P 185      -1.397 -16.040 -12.204  0.00  0.00      PROA
ATOM   2845  CD  ILE P 185      -1.675 -17.933 -13.208  0.00  0.00      PROA
ATOM   2846  HD1 ILE P 185      -0.961 -17.803 -14.050  0.00  0.00      PROA
ATOM   2847  HD2 ILE P 185      -2.729 -17.831 -13.543  0.00  0.00      PROA
```

| | | | | | | | | | | | |
|---|---|---|---|---|---|---|---|---|---|---|---|
| ATOM | 2848 | HD3 | ILE | P | 185 | -1.624 | -19.005 | -12.921 | 0.00 | 0.00 | PROA |
| ATOM | 2849 | C | ILE | P | 185 | -1.173 | -15.390 | -9.433 | 0.00 | 0.00 | PROA |
| ATOM | 2850 | O | ILE | P | 185 | -2.197 | -14.866 | -9.144 | 0.00 | 0.00 | PROA |
| ATOM | 2851 | N | ASN | P | 186 | -0.038 | -14.710 | -9.645 | 0.00 | 0.00 | PROA |
| ATOM | 2852 | HN | ASN | P | 186 | 0.807 | -15.206 | -9.827 | 0.00 | 0.00 | PROA |
| ATOM | 2853 | CA | ASN | P | 186 | 0.008 | -13.271 | -9.436 | 0.00 | 0.00 | PROA |
| ATOM | 2854 | HA | ASN | P | 186 | -0.488 | -13.026 | -8.508 | 0.00 | 0.00 | PROA |
| ATOM | 2855 | CB | ASN | P | 186 | 1.458 | -12.769 | -9.270 | 0.00 | 0.00 | PROA |
| ATOM | 2856 | HB1 | ASN | P | 186 | 1.449 | -11.684 | -9.032 | 0.00 | 0.00 | PROA |
| ATOM | 2857 | HB2 | ASN | P | 186 | 1.996 | -12.996 | -10.215 | 0.00 | 0.00 | PROA |
| ATOM | 2858 | CG | ASN | P | 186 | 2.201 | -13.519 | -8.187 | 0.00 | 0.00 | PROA |
| ATOM | 2859 | OD1 | ASN | P | 186 | 1.526 | -13.696 | -7.144 | 0.00 | 0.00 | PROA |
| ATOM | 2860 | ND2 | ASN | P | 186 | 3.462 | -13.734 | -8.317 | 0.00 | 0.00 | PROA |
| ATOM | 2861 | HD21 | ASN | P | 186 | 4.054 | -14.160 | -7.633 | 0.00 | 0.00 | PROA |
| ATOM | 2862 | HD22 | ASN | P | 186 | 3.964 | -13.472 | -9.142 | 0.00 | 0.00 | PROA |
| ATOM | 2863 | C | ASN | P | 186 | -0.846 | -12.501 | -10.474 | 0.00 | 0.00 | PROA |
| ATOM | 2864 | O | ASN | P | 186 | -0.756 | -12.842 | -11.635 | 0.00 | 0.00 | PROA |
| ATOM | 2865 | N | THR | P | 187 | -1.554 | -11.404 | -10.121 | 0.00 | 0.00 | PROA |
| ATOM | 2866 | HN | THR | P | 187 | -1.575 | -11.048 | -9.190 | 0.00 | 0.00 | PROA |
| ATOM | 2867 | CA | THR | P | 187 | -2.069 | -10.509 | -11.181 | 0.00 | 0.00 | PROA |
| ATOM | 2868 | HA | THR | P | 187 | -1.690 | -10.841 | -12.136 | 0.00 | 0.00 | PROA |
| ATOM | 2869 | CB | THR | P | 187 | -3.588 | -10.537 | -11.408 | 0.00 | 0.00 | PROA |
| ATOM | 2870 | HB | THR | P | 187 | -3.821 | -11.491 | -11.928 | 0.00 | 0.00 | PROA |
| ATOM | 2871 | OG1 | THR | P | 187 | -4.082 | -9.465 | -12.209 | 0.00 | 0.00 | PROA |
| ATOM | 2872 | HG1 | THR | P | 187 | -4.412 | -9.917 | -12.989 | 0.00 | 0.00 | PROA |
| ATOM | 2873 | CG2 | THR | P | 187 | -4.299 | -10.511 | -10.074 | 0.00 | 0.00 | PROA |
| ATOM | 2874 | HG21 | THR | P | 187 | -3.926 | -9.697 | -9.417 | 0.00 | 0.00 | PROA |
| ATOM | 2875 | HG22 | THR | P | 187 | -4.170 | -11.472 | -9.531 | 0.00 | 0.00 | PROA |
| ATOM | 2876 | HG23 | THR | P | 187 | -5.365 | -10.381 | -10.358 | 0.00 | 0.00 | PROA |
| ATOM | 2877 | C | THR | P | 187 | -1.549 | -9.127 | -11.049 | 0.00 | 0.00 | PROA |
| ATOM | 2878 | O | THR | P | 187 | -1.537 | -8.577 | -9.989 | 0.00 | 0.00 | PROA |
| ATOM | 2879 | N | LEU | P | 188 | -0.993 | -8.481 | -12.118 | 0.00 | 0.00 | PROA |
| ATOM | 2880 | HN | LEU | P | 188 | -0.793 | -9.070 | -12.897 | 0.00 | 0.00 | PROA |
| ATOM | 2881 | CA | LEU | P | 188 | -0.656 | -7.044 | -12.219 | 0.00 | 0.00 | PROA |
| ATOM | 2882 | HA | LEU | P | 188 | -0.210 | -6.805 | -11.265 | 0.00 | 0.00 | PROA |
| ATOM | 2883 | CB | LEU | P | 188 | 0.316 | -6.805 | -13.335 | 0.00 | 0.00 | PROA |
| ATOM | 2884 | HB1 | LEU | P | 188 | -0.023 | -7.341 | -14.247 | 0.00 | 0.00 | PROA |
| ATOM | 2885 | HB2 | LEU | P | 188 | 1.252 | -7.381 | -13.170 | 0.00 | 0.00 | PROA |
| ATOM | 2886 | CG | LEU | P | 188 | 0.795 | -5.360 | -13.629 | 0.00 | 0.00 | PROA |
| ATOM | 2887 | HG | LEU | P | 188 | 0.635 | -4.808 | -12.678 | 0.00 | 0.00 | PROA |
| ATOM | 2888 | CD1 | LEU | P | 188 | 2.329 | -5.375 | -13.756 | 0.00 | 0.00 | PROA |
| ATOM | 2889 | HD11 | LEU | P | 188 | 2.744 | -4.429 | -14.165 | 0.00 | 0.00 | PROA |
| ATOM | 2890 | HD12 | LEU | P | 188 | 2.692 | -6.163 | -14.450 | 0.00 | 0.00 | PROA |
| ATOM | 2891 | HD13 | LEU | P | 188 | 2.824 | -5.521 | -12.772 | 0.00 | 0.00 | PROA |
| ATOM | 2892 | CD2 | LEU | P | 188 | 0.039 | -4.686 | -14.706 | 0.00 | 0.00 | PROA |
| ATOM | 2893 | HD21 | LEU | P | 188 | 0.085 | -5.353 | -15.593 | 0.00 | 0.00 | PROA |
| ATOM | 2894 | HD22 | LEU | P | 188 | 0.515 | -3.716 | -14.962 | 0.00 | 0.00 | PROA |
| ATOM | 2895 | HD23 | LEU | P | 188 | -1.020 | -4.448 | -14.470 | 0.00 | 0.00 | PROA |
| ATOM | 2896 | C | LEU | P | 188 | -1.788 | -6.126 | -12.352 | 0.00 | 0.00 | PROA |
| ATOM | 2897 | O | LEU | P | 188 | -2.688 | -6.144 | -13.211 | 0.00 | 0.00 | PROA |
| ATOM | 2898 | N | LYS | P | 189 | -2.023 | -5.262 | -11.305 | 0.00 | 0.00 | PROA |
| ATOM | 2899 | HN | LYS | P | 189 | -1.373 | -5.117 | -10.563 | 0.00 | 0.00 | PROA |
| ATOM | 2900 | CA | LYS | P | 189 | -3.099 | -4.310 | -11.373 | 0.00 | 0.00 | PROA |
| ATOM | 2901 | HA | LYS | P | 189 | -3.870 | -4.575 | -12.081 | 0.00 | 0.00 | PROA |
| ATOM | 2902 | CB | LYS | P | 189 | -3.777 | -4.213 | -10.021 | 0.00 | 0.00 | PROA |
| ATOM | 2903 | HB1 | LYS | P | 189 | -4.660 | -3.540 | -10.083 | 0.00 | 0.00 | PROA |
| ATOM | 2904 | HB2 | LYS | P | 189 | -3.043 | -3.808 | -9.292 | 0.00 | 0.00 | PROA |
| ATOM | 2905 | CG | LYS | P | 189 | -4.303 | -5.627 | -9.448 | 0.00 | 0.00 | PROA |
| ATOM | 2906 | HG1 | LYS | P | 189 | -4.874 | -5.359 | -8.534 | 0.00 | 0.00 | PROA |
| ATOM | 2907 | HG2 | LYS | P | 189 | -3.406 | -6.137 | -9.034 | 0.00 | 0.00 | PROA |
| ATOM | 2908 | CD | LYS | P | 189 | -4.982 | -6.613 | -10.394 | 0.00 | 0.00 | PROA |
| ATOM | 2909 | HD1 | LYS | P | 189 | -5.351 | -7.425 | -9.732 | 0.00 | 0.00 | PROA |
| ATOM | 2910 | HD2 | LYS | P | 189 | -4.226 | -6.950 | -11.135 | 0.00 | 0.00 | PROA |
| ATOM | 2911 | CE | LYS | P | 189 | -6.220 | -6.092 | -11.130 | 0.00 | 0.00 | PROA |
| ATOM | 2912 | HE1 | LYS | P | 189 | -6.080 | -5.075 | -11.555 | 0.00 | 0.00 | PROA |
| ATOM | 2913 | HE2 | LYS | P | 189 | -6.954 | -6.007 | -10.301 | 0.00 | 0.00 | PROA |
| ATOM | 2914 | NZ | LYS | P | 189 | -6.672 | -6.945 | -12.236 | 0.00 | 0.00 | PROA |
| ATOM | 2915 | HZ1 | LYS | P | 189 | -6.897 | -7.943 | -12.046 | 0.00 | 0.00 | PROA |
| ATOM | 2916 | HZ2 | LYS | P | 189 | -6.060 | -6.972 | -13.076 | 0.00 | 0.00 | PROA |
| ATOM | 2917 | HZ3 | LYS | P | 189 | -7.603 | -6.619 | -12.565 | 0.00 | 0.00 | PROA |
| ATOM | 2918 | C | LYS | P | 189 | -2.444 | -2.967 | -11.712 | 0.00 | 0.00 | PROA |
| ATOM | 2919 | O | LYS | P | 189 | -1.332 | -2.625 | -11.249 | 0.00 | 0.00 | PROA |
| ATOM | 2920 | N | VAL | P | 190 | -3.181 | -2.183 | -12.546 | 0.00 | 0.00 | PROA |

| | | | | | | | | | | | |
|---|---|---|---|---|---|---|---|---|---|---|---|
| ATOM | 2921 | HN | VAL | P | 190 | -4.030 | -2.582 | -12.884 | 0.00 | 0.00 | PROA |
| ATOM | 2922 | CA | VAL | P | 190 | -2.667 | -0.979 | -13.134 | 0.00 | 0.00 | PROA |
| ATOM | 2923 | HA | VAL | P | 190 | -1.747 | -1.292 | -13.604 | 0.00 | 0.00 | PROA |
| ATOM | 2924 | CB | VAL | P | 190 | -3.674 | -0.485 | -14.194 | 0.00 | 0.00 | PROA |
| ATOM | 2925 | HB | VAL | P | 190 | -3.681 | -1.280 | -14.970 | 0.00 | 0.00 | PROA |
| ATOM | 2926 | CG1 | VAL | P | 190 | -5.146 | -0.252 | -13.684 | 0.00 | 0.00 | PROA |
| ATOM | 2927 | HG11 | VAL | P | 190 | -5.717 | 0.343 | -14.428 | 0.00 | 0.00 | PROA |
| ATOM | 2928 | HG12 | VAL | P | 190 | -5.192 | 0.355 | -12.754 | 0.00 | 0.00 | PROA |
| ATOM | 2929 | HG13 | VAL | P | 190 | -5.674 | -1.214 | -13.510 | 0.00 | 0.00 | PROA |
| ATOM | 2930 | CG2 | VAL | P | 190 | -3.178 | 0.855 | -14.792 | 0.00 | 0.00 | PROA |
| ATOM | 2931 | HG21 | VAL | P | 190 | -3.539 | 1.709 | -14.180 | 0.00 | 0.00 | PROA |
| ATOM | 2932 | HG22 | VAL | P | 190 | -3.591 | 1.019 | -15.811 | 0.00 | 0.00 | PROA |
| ATOM | 2933 | HG23 | VAL | P | 190 | -2.068 | 0.880 | -14.824 | 0.00 | 0.00 | PROA |
| ATOM | 2934 | C | VAL | P | 190 | -2.357 | 0.057 | -12.119 | 0.00 | 0.00 | PROA |
| ATOM | 2935 | O | VAL | P | 190 | -1.247 | 0.626 | -12.049 | 0.00 | 0.00 | PROA |
| ATOM | 2936 | N | THR | P | 191 | -3.293 | 0.404 | -11.253 | 0.00 | 0.00 | PROA |
| ATOM | 2937 | HN | THR | P | 191 | -4.173 | -0.062 | -11.194 | 0.00 | 0.00 | PROA |
| ATOM | 2938 | CA | THR | P | 191 | -3.191 | 1.641 | -10.423 | 0.00 | 0.00 | PROA |
| ATOM | 2939 | HA | THR | P | 191 | -2.275 | 2.146 | -10.693 | 0.00 | 0.00 | PROA |
| ATOM | 2940 | CB | THR | P | 191 | -4.457 | 2.592 | -10.550 | 0.00 | 0.00 | PROA |
| ATOM | 2941 | HB | THR | P | 191 | -4.607 | 2.728 | -11.643 | 0.00 | 0.00 | PROA |
| ATOM | 2942 | OG1 | THR | P | 191 | -4.318 | 3.872 | -9.889 | 0.00 | 0.00 | PROA |
| ATOM | 2943 | HG1 | THR | P | 191 | -3.507 | 4.298 | -10.176 | 0.00 | 0.00 | PROA |
| ATOM | 2944 | CG2 | THR | P | 191 | -5.659 | 1.936 | -9.960 | 0.00 | 0.00 | PROA |
| ATOM | 2945 | HG21 | THR | P | 191 | -6.585 | 2.509 | -10.182 | 0.00 | 0.00 | PROA |
| ATOM | 2946 | HG22 | THR | P | 191 | -5.523 | 1.764 | -8.871 | 0.00 | 0.00 | PROA |
| ATOM | 2947 | HG23 | THR | P | 191 | -5.826 | 0.928 | -10.398 | 0.00 | 0.00 | PROA |
| ATOM | 2948 | C | THR | P | 191 | -3.053 | 1.269 | -8.983 | 0.00 | 0.00 | PROA |
| ATOM | 2949 | O | THR | P | 191 | -2.820 | 2.174 | -8.166 | 0.00 | 0.00 | PROA |
| ATOM | 2950 | N | ALA | P | 192 | -3.102 | -0.012 | -8.652 | 0.00 | 0.00 | PROA |
| ATOM | 2951 | HN | ALA | P | 192 | -3.146 | -0.731 | -9.341 | 0.00 | 0.00 | PROA |
| ATOM | 2952 | CA | ALA | P | 192 | -2.955 | -0.498 | -7.276 | 0.00 | 0.00 | PROA |
| ATOM | 2953 | HA | ALA | P | 192 | -2.952 | 0.355 | -6.614 | 0.00 | 0.00 | PROA |
| ATOM | 2954 | CB | ALA | P | 192 | -4.145 | -1.462 | -6.986 | 0.00 | 0.00 | PROA |
| ATOM | 2955 | HB1 | ALA | P | 192 | -5.118 | -0.927 | -6.946 | 0.00 | 0.00 | PROA |
| ATOM | 2956 | HB2 | ALA | P | 192 | -4.035 | -1.818 | -5.939 | 0.00 | 0.00 | PROA |
| ATOM | 2957 | HB3 | ALA | P | 192 | -4.125 | -2.343 | -7.663 | 0.00 | 0.00 | PROA |
| ATOM | 2958 | C | ALA | P | 192 | -1.610 | -1.191 | -7.044 | 0.00 | 0.00 | PROA |
| ATOM | 2959 | O | ALA | P | 192 | -1.301 | -2.126 | -7.712 | 0.00 | 0.00 | PROA |
| ATOM | 2960 | N | GLY | P | 193 | -0.728 | -0.661 | -6.189 | 0.00 | 0.00 | PROA |
| ATOM | 2961 | HN | GLY | P | 193 | -1.271 | -0.052 | -5.617 | 0.00 | 0.00 | PROA |
| ATOM | 2962 | CA | GLY | P | 193 | 0.681 | -1.002 | -6.032 | 0.00 | 0.00 | PROA |
| ATOM | 2963 | HA1 | GLY | P | 193 | 1.213 | -0.224 | -5.505 | 0.00 | 0.00 | PROA |
| ATOM | 2964 | HA2 | GLY | P | 193 | 1.006 | -1.171 | -7.048 | 0.00 | 0.00 | PROA |
| ATOM | 2965 | C | GLY | P | 193 | 0.784 | -2.226 | -5.261 | 0.00 | 0.00 | PROA |
| ATOM | 2966 | O | GLY | P | 193 | 1.829 | -2.935 | -5.353 | 0.00 | 0.00 | PROA |
| ATOM | 2967 | N | ILE | P | 194 | -0.198 | -2.422 | -4.342 | 0.00 | 0.00 | PROA |
| ATOM | 2968 | HN | ILE | P | 194 | -0.914 | -1.731 | -4.276 | 0.00 | 0.00 | PROA |
| ATOM | 2969 | CA | ILE | P | 194 | -0.226 | -3.606 | -3.574 | 0.00 | 0.00 | PROA |
| ATOM | 2970 | HA | ILE | P | 194 | 0.673 | -3.694 | -2.981 | 0.00 | 0.00 | PROA |
| ATOM | 2971 | CB | ILE | P | 194 | -1.290 | -3.626 | -2.497 | 0.00 | 0.00 | PROA |
| ATOM | 2972 | HB | ILE | P | 194 | -1.273 | -2.693 | -1.893 | 0.00 | 0.00 | PROA |
| ATOM | 2973 | CG2 | ILE | P | 194 | -2.757 | -3.640 | -3.103 | 0.00 | 0.00 | PROA |
| ATOM | 2974 | HG21 | ILE | P | 194 | -2.678 | -3.071 | -4.054 | 0.00 | 0.00 | PROA |
| ATOM | 2975 | HG22 | ILE | P | 194 | -3.484 | -3.179 | -2.401 | 0.00 | 0.00 | PROA |
| ATOM | 2976 | HG23 | ILE | P | 194 | -3.063 | -4.699 | -3.241 | 0.00 | 0.00 | PROA |
| ATOM | 2977 | CG1 | ILE | P | 194 | -1.199 | -4.648 | -1.365 | 0.00 | 0.00 | PROA |
| ATOM | 2978 | HG11 | ILE | P | 194 | -0.096 | -4.760 | -1.289 | 0.00 | 0.00 | PROA |
| ATOM | 2979 | HG12 | ILE | P | 194 | -1.634 | -5.611 | -1.709 | 0.00 | 0.00 | PROA |
| ATOM | 2980 | CD | ILE | P | 194 | -1.813 | -4.225 | -0.024 | 0.00 | 0.00 | PROA |
| ATOM | 2981 | HD1 | ILE | P | 194 | -2.903 | -4.043 | -0.146 | 0.00 | 0.00 | PROA |
| ATOM | 2982 | HD2 | ILE | P | 194 | -1.401 | -3.291 | 0.414 | 0.00 | 0.00 | PROA |
| ATOM | 2983 | HD3 | ILE | P | 194 | -1.650 | -5.091 | 0.652 | 0.00 | 0.00 | PROA |
| ATOM | 2984 | C | ILE | P | 194 | -0.386 | -4.940 | -4.433 | 0.00 | 0.00 | PROA |
| ATOM | 2985 | O | ILE | P | 194 | -0.941 | -4.994 | -5.520 | 0.00 | 0.00 | PROA |
| ATOM | 2986 | N | SER | P | 195 | 0.230 | -6.058 | -3.884 | 0.00 | 0.00 | PROA |
| ATOM | 2987 | HN | SER | P | 195 | 0.810 | -5.859 | -3.098 | 0.00 | 0.00 | PROA |
| ATOM | 2988 | CA | SER | P | 195 | 0.389 | -7.410 | -4.446 | 0.00 | 0.00 | PROA |
| ATOM | 2989 | HA | SER | P | 195 | 0.634 | -7.259 | -5.487 | 0.00 | 0.00 | PROA |
| ATOM | 2990 | CB | SER | P | 195 | 1.583 | -8.163 | -3.953 | 0.00 | 0.00 | PROA |
| ATOM | 2991 | HB1 | SER | P | 195 | 1.371 | -8.541 | -2.930 | 0.00 | 0.00 | PROA |
| ATOM | 2992 | HB2 | SER | P | 195 | 2.351 | -7.360 | -3.966 | 0.00 | 0.00 | PROA |
| ATOM | 2993 | OG | SER | P | 195 | 2.024 | -9.105 | -4.918 | 0.00 | 0.00 | PROA |

```
ATOM   2994  HG1 SER P 195       1.400  -9.833  -4.966  0.00  0.00      PROA
ATOM   2995  C   SER P 195      -0.874  -8.241  -4.276  0.00  0.00      PROA
ATOM   2996  O   SER P 195      -1.262  -8.683  -3.251  0.00  0.00      PROA
ATOM   2997  N   PHE P 196      -1.518  -8.485  -5.434  0.00  0.00      PROA
ATOM   2998  HN  PHE P 196      -1.198  -8.114  -6.302  0.00  0.00      PROA
ATOM   2999  CA  PHE P 196      -2.560  -9.424  -5.594  0.00  0.00      PROA
ATOM   3000  HA  PHE P 196      -3.032  -9.595  -4.638  0.00  0.00      PROA
ATOM   3001  CB  PHE P 196      -3.822  -8.767  -6.321  0.00  0.00      PROA
ATOM   3002  HB1 PHE P 196      -4.621  -9.527  -6.459  0.00  0.00      PROA
ATOM   3003  HB2 PHE P 196      -3.398  -8.328  -7.249  0.00  0.00      PROA
ATOM   3004  CG  PHE P 196      -4.397  -7.712  -5.487  0.00  0.00      PROA
ATOM   3005  CD1 PHE P 196      -5.301  -8.051  -4.484  0.00  0.00      PROA
ATOM   3006  HD1 PHE P 196      -5.463  -9.075  -4.180  0.00  0.00      PROA
ATOM   3007  CE1 PHE P 196      -5.976  -7.028  -3.814  0.00  0.00      PROA
ATOM   3008  HE1 PHE P 196      -6.632  -7.253  -2.987  0.00  0.00      PROA
ATOM   3009  CZ  PHE P 196      -5.910  -5.727  -4.213  0.00  0.00      PROA
ATOM   3010  HZ  PHE P 196      -6.565  -4.941  -3.869  0.00  0.00      PROA
ATOM   3011  CD2 PHE P 196      -4.155  -6.313  -5.785  0.00  0.00      PROA
ATOM   3012  HD2 PHE P 196      -3.516  -6.013  -6.603  0.00  0.00      PROA
ATOM   3013  CE2 PHE P 196      -4.978  -5.337  -5.185  0.00  0.00      PROA
ATOM   3014  HE2 PHE P 196      -4.779  -4.302  -5.420  0.00  0.00      PROA
ATOM   3015  C   PHE P 196      -2.320 -10.773  -6.277  0.00  0.00      PROA
ATOM   3016  O   PHE P 196      -1.658 -10.896  -7.341  0.00  0.00      PROA
ATOM   3017  N   ALA P 197      -2.877 -11.890  -5.738  0.00  0.00      PROA
ATOM   3018  HN  ALA P 197      -3.510 -11.702  -4.991  0.00  0.00      PROA
ATOM   3019  CA  ALA P 197      -2.766 -13.240  -6.255  0.00  0.00      PROA
ATOM   3020  HA  ALA P 197      -2.367 -13.162  -7.256  0.00  0.00      PROA
ATOM   3021  CB  ALA P 197      -1.944 -14.120  -5.389  0.00  0.00      PROA
ATOM   3022  HB1 ALA P 197      -0.959 -13.634  -5.220  0.00  0.00      PROA
ATOM   3023  HB2 ALA P 197      -1.606 -15.104  -5.779  0.00  0.00      PROA
ATOM   3024  HB3 ALA P 197      -2.334 -14.333  -4.370  0.00  0.00      PROA
ATOM   3025  C   ALA P 197      -4.138 -13.739  -6.497  0.00  0.00      PROA
ATOM   3026  O   ALA P 197      -5.081 -13.518  -5.753  0.00  0.00      PROA
ATOM   3027  N   ILE P 198      -4.480 -14.390  -7.680  0.00  0.00      PROA
ATOM   3028  HN  ILE P 198      -3.740 -14.474  -8.343  0.00  0.00      PROA
ATOM   3029  CA  ILE P 198      -5.672 -15.239  -7.929  0.00  0.00      PROA
ATOM   3030  HA  ILE P 198      -6.507 -14.633  -7.609  0.00  0.00      PROA
ATOM   3031  CB  ILE P 198      -5.873 -15.442  -9.428  0.00  0.00      PROA
ATOM   3032  HB  ILE P 198      -4.838 -15.639  -9.781  0.00  0.00      PROA
ATOM   3033  CG2 ILE P 198      -6.906 -16.553  -9.754  0.00  0.00      PROA
ATOM   3034 HG21 ILE P 198      -7.876 -16.272  -9.291  0.00  0.00      PROA
ATOM   3035 HG22 ILE P 198      -6.773 -17.613  -9.447  0.00  0.00      PROA
ATOM   3036 HG23 ILE P 198      -7.071 -16.540 -10.853  0.00  0.00      PROA
ATOM   3037  CG1 ILE P 198      -6.310 -14.095 -10.092  0.00  0.00      PROA
ATOM   3038 HG11 ILE P 198      -5.941 -13.267  -9.449  0.00  0.00      PROA
ATOM   3039 HG12 ILE P 198      -7.393 -13.874  -9.983  0.00  0.00      PROA
ATOM   3040  CD  ILE P 198      -5.774 -13.810 -11.488  0.00  0.00      PROA
ATOM   3041  HD1 ILE P 198      -4.666 -13.841 -11.571  0.00  0.00      PROA
ATOM   3042  HD2 ILE P 198      -6.235 -12.854 -11.815  0.00  0.00      PROA
ATOM   3043  HD3 ILE P 198      -6.164 -14.643 -12.111  0.00  0.00      PROA
ATOM   3044  C   ILE P 198      -5.575 -16.499  -7.175  0.00  0.00      PROA
ATOM   3045  O   ILE P 198      -4.446 -16.962  -7.179  0.00  0.00      PROA
ATOM   3046  N   PRO P 199      -6.478 -17.090  -6.531  0.00  0.00      PROA
ATOM   3047  CD  PRO P 199      -7.611 -16.285  -6.030  0.00  0.00      PROA
ATOM   3048  HD1 PRO P 199      -8.237 -16.032  -6.912  0.00  0.00      PROA
ATOM   3049  HD2 PRO P 199      -7.365 -15.380  -5.434  0.00  0.00      PROA
ATOM   3050  CA  PRO P 199      -6.486 -18.419  -5.956  0.00  0.00      PROA
ATOM   3051  HA  PRO P 199      -5.781 -18.417  -5.138  0.00  0.00      PROA
ATOM   3052  CB  PRO P 199      -7.980 -18.651  -5.437  0.00  0.00      PROA
ATOM   3053  HB1 PRO P 199      -7.971 -19.326  -4.554  0.00  0.00      PROA
ATOM   3054  HB2 PRO P 199      -8.628 -19.111  -6.213  0.00  0.00      PROA
ATOM   3055  CG  PRO P 199      -8.469 -17.258  -5.152  0.00  0.00      PROA
ATOM   3056  HG1 PRO P 199      -9.509 -17.130  -5.519  0.00  0.00      PROA
ATOM   3057  HG2 PRO P 199      -8.411 -16.919  -4.096  0.00  0.00      PROA
ATOM   3058  C   PRO P 199      -6.067 -19.524  -6.827  0.00  0.00      PROA
ATOM   3059  O   PRO P 199      -6.526 -19.712  -7.934  0.00  0.00      PROA
ATOM   3060  N   SER P 200      -5.226 -20.448  -6.317  0.00  0.00      PROA
ATOM   3061  HN  SER P 200      -4.782 -20.252  -5.446  0.00  0.00      PROA
ATOM   3062  CA  SER P 200      -4.902 -21.730  -6.906  0.00  0.00      PROA
ATOM   3063  HA  SER P 200      -4.348 -21.544  -7.814  0.00  0.00      PROA
ATOM   3064  CB  SER P 200      -3.903 -22.480  -5.920  0.00  0.00      PROA
ATOM   3065  HB1 SER P 200      -2.897 -22.025  -5.795  0.00  0.00      PROA
ATOM   3066  HB2 SER P 200      -3.610 -23.443  -6.389  0.00  0.00      PROA
```

| | | | | | | | | | | | |
|---|---|---|---|---|---|---|---|---|---|---|---|
| ATOM | 3067 | OG | SER | P | 200 | -4.412 | -22.536 | -4.621 | 0.00 | 0.00 | PROA |
| ATOM | 3068 | HG1 | SER | P | 200 | -4.524 | -23.434 | -4.301 | 0.00 | 0.00 | PROA |
| ATOM | 3069 | C | SER | P | 200 | -6.098 | -22.574 | -7.154 | 0.00 | 0.00 | PROA |
| ATOM | 3070 | O | SER | P | 200 | -6.130 | -23.290 | -8.184 | 0.00 | 0.00 | PROA |
| ATOM | 3071 | N | ASP | P | 201 | -7.066 | -22.519 | -6.244 | 0.00 | 0.00 | PROA |
| ATOM | 3072 | HN | ASP | P | 201 | -6.752 | -22.330 | -5.317 | 0.00 | 0.00 | PROA |
| ATOM | 3073 | CA | ASP | P | 201 | -8.379 | -23.131 | -6.369 | 0.00 | 0.00 | PROA |
| ATOM | 3074 | HA | ASP | P | 201 | -8.360 | -24.208 | -6.286 | 0.00 | 0.00 | PROA |
| ATOM | 3075 | CB | ASP | P | 201 | -9.361 | -22.574 | -5.334 | 0.00 | 0.00 | PROA |
| ATOM | 3076 | HB1 | ASP | P | 201 | -10.368 | -23.023 | -5.470 | 0.00 | 0.00 | PROA |
| ATOM | 3077 | HB2 | ASP | P | 201 | -9.530 | -21.503 | -5.573 | 0.00 | 0.00 | PROA |
| ATOM | 3078 | CG | ASP | P | 201 | -8.919 | -22.749 | -3.942 | 0.00 | 0.00 | PROA |
| ATOM | 3079 | OD1 | ASP | P | 201 | -9.692 | -22.374 | -2.988 | 0.00 | 0.00 | PROA |
| ATOM | 3080 | OD2 | ASP | P | 201 | -7.763 | -23.288 | -3.676 | 0.00 | 0.00 | PROA |
| ATOM | 3081 | C | ASP | P | 201 | -9.053 | -22.867 | -7.770 | 0.00 | 0.00 | PROA |
| ATOM | 3082 | O | ASP | P | 201 | -9.566 | -23.758 | -8.368 | 0.00 | 0.00 | PROA |
| ATOM | 3083 | N | LYS | P | 202 | -8.994 | -21.620 | -8.256 | 0.00 | 0.00 | PROA |
| ATOM | 3084 | HN | LYS | P | 202 | -8.408 | -20.942 | -7.817 | 0.00 | 0.00 | PROA |
| ATOM | 3085 | CA | LYS | P | 202 | -9.644 | -21.110 | -9.496 | 0.00 | 0.00 | PROA |
| ATOM | 3086 | HA | LYS | P | 202 | -10.638 | -21.525 | -9.573 | 0.00 | 0.00 | PROA |
| ATOM | 3087 | CB | LYS | P | 202 | -9.707 | -19.554 | -9.558 | 0.00 | 0.00 | PROA |
| ATOM | 3088 | HB1 | LYS | P | 202 | -8.792 | -19.137 | -9.085 | 0.00 | 0.00 | PROA |
| ATOM | 3089 | HB2 | LYS | P | 202 | -10.506 | -19.223 | -8.861 | 0.00 | 0.00 | PROA |
| ATOM | 3090 | CG | LYS | P | 202 | -9.715 | -18.921 | -11.002 | 0.00 | 0.00 | PROA |
| ATOM | 3091 | HG1 | LYS | P | 202 | -8.788 | -19.171 | -11.562 | 0.00 | 0.00 | PROA |
| ATOM | 3092 | HG2 | LYS | P | 202 | -9.634 | -17.819 | -10.885 | 0.00 | 0.00 | PROA |
| ATOM | 3093 | CD | LYS | P | 202 | -10.910 | -19.199 | -11.871 | 0.00 | 0.00 | PROA |
| ATOM | 3094 | HD1 | LYS | P | 202 | -10.978 | -20.162 | -12.420 | 0.00 | 0.00 | PROA |
| ATOM | 3095 | HD2 | LYS | P | 202 | -10.861 | -18.492 | -12.727 | 0.00 | 0.00 | PROA |
| ATOM | 3096 | CE | LYS | P | 202 | -12.242 | -19.110 | -11.137 | 0.00 | 0.00 | PROA |
| ATOM | 3097 | HE1 | LYS | P | 202 | -12.342 | -18.132 | -10.619 | 0.00 | 0.00 | PROA |
| ATOM | 3098 | HE2 | LYS | P | 202 | -12.360 | -19.956 | -10.426 | 0.00 | 0.00 | PROA |
| ATOM | 3099 | NZ | LYS | P | 202 | -13.425 | -19.266 | -12.035 | 0.00 | 0.00 | PROA |
| ATOM | 3100 | HZ1 | LYS | P | 202 | -13.443 | -18.741 | -12.932 | 0.00 | 0.00 | PROA |
| ATOM | 3101 | HZ2 | LYS | P | 202 | -14.274 | -19.039 | -11.478 | 0.00 | 0.00 | PROA |
| ATOM | 3102 | HZ3 | LYS | P | 202 | -13.514 | -20.282 | -12.239 | 0.00 | 0.00 | PROA |
| ATOM | 3103 | C | LYS | P | 202 | -8.899 | -21.700 | -10.727 | 0.00 | 0.00 | PROA |
| ATOM | 3104 | O | LYS | P | 202 | -9.495 | -22.238 | -11.648 | 0.00 | 0.00 | PROA |
| ATOM | 3105 | N | ILE | P | 203 | -7.536 | -21.723 | -10.662 | 0.00 | 0.00 | PROA |
| ATOM | 3106 | HN | ILE | P | 203 | -7.086 | -21.359 | -9.850 | 0.00 | 0.00 | PROA |
| ATOM | 3107 | CA | ILE | P | 203 | -6.695 | -22.423 | -11.577 | 0.00 | 0.00 | PROA |
| ATOM | 3108 | HA | ILE | P | 203 | -6.837 | -21.938 | -12.532 | 0.00 | 0.00 | PROA |
| ATOM | 3109 | CB | ILE | P | 203 | -5.236 | -22.384 | -11.230 | 0.00 | 0.00 | PROA |
| ATOM | 3110 | HB | ILE | P | 203 | -4.982 | -23.048 | -10.376 | 0.00 | 0.00 | PROA |
| ATOM | 3111 | CG2 | ILE | P | 203 | -4.520 | -22.911 | -12.554 | 0.00 | 0.00 | PROA |
| ATOM | 3112 | HG21 | ILE | P | 203 | -4.481 | -24.014 | -12.688 | 0.00 | 0.00 | PROA |
| ATOM | 3113 | HG22 | ILE | P | 203 | -3.425 | -22.723 | -12.539 | 0.00 | 0.00 | PROA |
| ATOM | 3114 | HG23 | ILE | P | 203 | -5.030 | -22.425 | -13.413 | 0.00 | 0.00 | PROA |
| ATOM | 3115 | CG1 | ILE | P | 203 | -4.860 | -21.060 | -10.732 | 0.00 | 0.00 | PROA |
| ATOM | 3116 | HG11 | ILE | P | 203 | -5.392 | -20.808 | -9.790 | 0.00 | 0.00 | PROA |
| ATOM | 3117 | HG12 | ILE | P | 203 | -5.060 | -20.189 | -11.392 | 0.00 | 0.00 | PROA |
| ATOM | 3118 | CD | ILE | P | 203 | -3.419 | -21.102 | -10.257 | 0.00 | 0.00 | PROA |
| ATOM | 3119 | HD1 | ILE | P | 203 | -3.167 | -22.129 | -9.918 | 0.00 | 0.00 | PROA |
| ATOM | 3120 | HD2 | ILE | P | 203 | -3.119 | -20.351 | -9.496 | 0.00 | 0.00 | PROA |
| ATOM | 3121 | HD3 | ILE | P | 203 | -2.851 | -20.786 | -11.158 | 0.00 | 0.00 | PROA |
| ATOM | 3122 | C | ILE | P | 203 | -7.063 | -23.906 | -11.699 | 0.00 | 0.00 | PROA |
| ATOM | 3123 | O | ILE | P | 203 | -7.260 | -24.378 | -12.791 | 0.00 | 0.00 | PROA |
| ATOM | 3124 | N | LYS | P | 204 | -7.238 | -24.609 | -10.573 | 0.00 | 0.00 | PROA |
| ATOM | 3125 | HN | LYS | P | 204 | -7.030 | -24.066 | -9.762 | 0.00 | 0.00 | PROA |
| ATOM | 3126 | CA | LYS | P | 204 | -7.567 | -25.944 | -10.399 | 0.00 | 0.00 | PROA |
| ATOM | 3127 | HA | LYS | P | 204 | -6.826 | -26.465 | -10.987 | 0.00 | 0.00 | PROA |
| ATOM | 3128 | CB | LYS | P | 204 | -7.620 | -26.356 | -8.923 | 0.00 | 0.00 | PROA |
| ATOM | 3129 | HB1 | LYS | P | 204 | -8.233 | -25.705 | -8.263 | 0.00 | 0.00 | PROA |
| ATOM | 3130 | HB2 | LYS | P | 204 | -6.559 | -26.422 | -8.599 | 0.00 | 0.00 | PROA |
| ATOM | 3131 | CG | LYS | P | 204 | -8.099 | -27.750 | -8.670 | 0.00 | 0.00 | PROA |
| ATOM | 3132 | HG1 | LYS | P | 204 | -7.520 | -28.483 | -9.271 | 0.00 | 0.00 | PROA |
| ATOM | 3133 | HG2 | LYS | P | 204 | -9.168 | -27.929 | -8.916 | 0.00 | 0.00 | PROA |
| ATOM | 3134 | CD | LYS | P | 204 | -7.932 | -28.268 | -7.247 | 0.00 | 0.00 | PROA |
| ATOM | 3135 | HD1 | LYS | P | 204 | -7.842 | -27.460 | -6.489 | 0.00 | 0.00 | PROA |
| ATOM | 3136 | HD2 | LYS | P | 204 | -6.877 | -28.618 | -7.229 | 0.00 | 0.00 | PROA |
| ATOM | 3137 | CE | LYS | P | 204 | -8.858 | -29.401 | -6.994 | 0.00 | 0.00 | PROA |
| ATOM | 3138 | HE1 | LYS | P | 204 | -8.844 | -30.189 | -7.776 | 0.00 | 0.00 | PROA |
| ATOM | 3139 | HE2 | LYS | P | 204 | -9.864 | -28.962 | -6.819 | 0.00 | 0.00 | PROA |

| | | | | | | | | | | | |
|---|---|---|---|---|---|---|---|---|---|---|---|
| ATOM | 3140 | NZ | LYS | P | 204 | -8.568 | -30.125 | -5.708 | 0.00 | 0.00 | PROA |
| ATOM | 3141 | HZ1 | LYS | P | 204 | -9.252 | -30.862 | -5.442 | 0.00 | 0.00 | PROA |
| ATOM | 3142 | HZ2 | LYS | P | 204 | -8.601 | -29.436 | -4.929 | 0.00 | 0.00 | PROA |
| ATOM | 3143 | HZ3 | LYS | P | 204 | -7.591 | -30.460 | -5.829 | 0.00 | 0.00 | PROA |
| ATOM | 3144 | C | LYS | P | 204 | -8.954 | -26.219 | -11.026 | 0.00 | 0.00 | PROA |
| ATOM | 3145 | O | LYS | P | 204 | -9.140 | -27.221 | -11.788 | 0.00 | 0.00 | PROA |
| ATOM | 3146 | N | LYS | P | 205 | -9.969 | -25.392 | -10.777 | 0.00 | 0.00 | PROA |
| ATOM | 3147 | HN | LYS | P | 205 | -9.774 | -24.591 | -10.216 | 0.00 | 0.00 | PROA |
| ATOM | 3148 | CA | LYS | P | 205 | -11.246 | -25.483 | -11.479 | 0.00 | 0.00 | PROA |
| ATOM | 3149 | HA | LYS | P | 205 | -11.613 | -26.472 | -11.247 | 0.00 | 0.00 | PROA |
| ATOM | 3150 | CB | LYS | P | 205 | -12.279 | -24.476 | -10.890 | 0.00 | 0.00 | PROA |
| ATOM | 3151 | HB1 | LYS | P | 205 | -11.962 | -23.430 | -11.092 | 0.00 | 0.00 | PROA |
| ATOM | 3152 | HB2 | LYS | P | 205 | -12.166 | -24.593 | -9.791 | 0.00 | 0.00 | PROA |
| ATOM | 3153 | CG | LYS | P | 205 | -13.798 | -24.664 | -11.306 | 0.00 | 0.00 | PROA |
| ATOM | 3154 | HG1 | LYS | P | 205 | -13.892 | -24.590 | -12.410 | 0.00 | 0.00 | PROA |
| ATOM | 3155 | HG2 | LYS | P | 205 | -14.335 | -23.800 | -10.857 | 0.00 | 0.00 | PROA |
| ATOM | 3156 | CD | LYS | P | 205 | -14.509 | -25.908 | -10.769 | 0.00 | 0.00 | PROA |
| ATOM | 3157 | HD1 | LYS | P | 205 | -14.326 | -26.142 | -9.698 | 0.00 | 0.00 | PROA |
| ATOM | 3158 | HD2 | LYS | P | 205 | -14.127 | -26.804 | -11.303 | 0.00 | 0.00 | PROA |
| ATOM | 3159 | CE | LYS | P | 205 | -16.083 | -25.843 | -10.904 | 0.00 | 0.00 | PROA |
| ATOM | 3160 | HE1 | LYS | P | 205 | -16.551 | -24.912 | -10.522 | 0.00 | 0.00 | PROA |
| ATOM | 3161 | HE2 | LYS | P | 205 | -16.406 | -26.729 | -10.317 | 0.00 | 0.00 | PROA |
| ATOM | 3162 | NZ | LYS | P | 205 | -16.527 | -26.111 | -12.269 | 0.00 | 0.00 | PROA |
| ATOM | 3163 | HZ1 | LYS | P | 205 | -17.532 | -26.374 | -12.300 | 0.00 | 0.00 | PROA |
| ATOM | 3164 | HZ2 | LYS | P | 205 | -15.855 | -26.833 | -12.600 | 0.00 | 0.00 | PROA |
| ATOM | 3165 | HZ3 | LYS | P | 205 | -16.329 | -25.287 | -12.872 | 0.00 | 0.00 | PROA |
| ATOM | 3166 | C | LYS | P | 205 | -11.105 | -25.240 | -12.976 | 0.00 | 0.00 | PROA |
| ATOM | 3167 | O | LYS | P | 205 | -11.592 | -26.027 | -13.794 | 0.00 | 0.00 | PROA |
| ATOM | 3168 | N | PHE | P | 206 | -10.312 | -24.273 | -13.445 | 0.00 | 0.00 | PROA |
| ATOM | 3169 | HN | PHE | P | 206 | -9.908 | -23.705 | -12.732 | 0.00 | 0.00 | PROA |
| ATOM | 3170 | CA | PHE | P | 206 | -10.010 | -23.888 | -14.828 | 0.00 | 0.00 | PROA |
| ATOM | 3171 | HA | PHE | P | 206 | -10.987 | -23.728 | -15.261 | 0.00 | 0.00 | PROA |
| ATOM | 3172 | CB | PHE | P | 206 | -9.225 | -22.504 | -14.780 | 0.00 | 0.00 | PROA |
| ATOM | 3173 | HB1 | PHE | P | 206 | -8.294 | -22.548 | -14.176 | 0.00 | 0.00 | PROA |
| ATOM | 3174 | HB2 | PHE | P | 206 | -9.819 | -21.752 | -14.220 | 0.00 | 0.00 | PROA |
| ATOM | 3175 | CG | PHE | P | 206 | -8.852 | -22.004 | -16.092 | 0.00 | 0.00 | PROA |
| ATOM | 3176 | CD1 | PHE | P | 206 | -9.469 | -20.885 | -16.679 | 0.00 | 0.00 | PROA |
| ATOM | 3177 | HD1 | PHE | P | 206 | -10.234 | -20.336 | -16.150 | 0.00 | 0.00 | PROA |
| ATOM | 3178 | CE1 | PHE | P | 206 | -9.039 | -20.462 | -17.923 | 0.00 | 0.00 | PROA |
| ATOM | 3179 | HE1 | PHE | P | 206 | -9.470 | -19.647 | -18.486 | 0.00 | 0.00 | PROA |
| ATOM | 3180 | CZ | PHE | P | 206 | -7.958 | -21.153 | -18.597 | 0.00 | 0.00 | PROA |
| ATOM | 3181 | HZ | PHE | P | 206 | -7.763 | -20.974 | -19.644 | 0.00 | 0.00 | PROA |
| ATOM | 3182 | CD2 | PHE | P | 206 | -7.691 | -22.631 | -16.644 | 0.00 | 0.00 | PROA |
| ATOM | 3183 | HD2 | PHE | P | 206 | -7.076 | -23.368 | -16.149 | 0.00 | 0.00 | PROA |
| ATOM | 3184 | CE2 | PHE | P | 206 | -7.332 | -22.198 | -17.908 | 0.00 | 0.00 | PROA |
| ATOM | 3185 | HE2 | PHE | P | 206 | -6.575 | -22.826 | -18.353 | 0.00 | 0.00 | PROA |
| ATOM | 3186 | C | PHE | P | 206 | -9.377 | -25.040 | -15.571 | 0.00 | 0.00 | PROA |
| ATOM | 3187 | O | PHE | P | 206 | -9.758 | -25.279 | -16.686 | 0.00 | 0.00 | PROA |
| ATOM | 3188 | N | LEU | P | 207 | -8.491 | -25.728 | -14.934 | 0.00 | 0.00 | PROA |
| ATOM | 3189 | HN | LEU | P | 207 | -8.081 | -25.319 | -14.122 | 0.00 | 0.00 | PROA |
| ATOM | 3190 | CA | LEU | P | 207 | -7.895 | -26.991 | -15.335 | 0.00 | 0.00 | PROA |
| ATOM | 3191 | HA | LEU | P | 207 | -7.553 | -26.939 | -16.358 | 0.00 | 0.00 | PROA |
| ATOM | 3192 | CB | LEU | P | 207 | -6.628 | -27.427 | -14.512 | 0.00 | 0.00 | PROA |
| ATOM | 3193 | HB1 | LEU | P | 207 | -6.358 | -28.487 | -14.708 | 0.00 | 0.00 | PROA |
| ATOM | 3194 | HB2 | LEU | P | 207 | -6.984 | -27.403 | -13.459 | 0.00 | 0.00 | PROA |
| ATOM | 3195 | CG | LEU | P | 207 | -5.457 | -26.489 | -14.704 | 0.00 | 0.00 | PROA |
| ATOM | 3196 | HG | LEU | P | 207 | -5.795 | -25.430 | -14.712 | 0.00 | 0.00 | PROA |
| ATOM | 3197 | CD1 | LEU | P | 207 | -4.601 | -26.593 | -13.450 | 0.00 | 0.00 | PROA |
| ATOM | 3198 | HD11 | LEU | P | 207 | -4.092 | -27.580 | -13.494 | 0.00 | 0.00 | PROA |
| ATOM | 3199 | HD12 | LEU | P | 207 | -5.220 | -26.696 | -12.533 | 0.00 | 0.00 | PROA |
| ATOM | 3200 | HD13 | LEU | P | 207 | -3.806 | -25.833 | -13.605 | 0.00 | 0.00 | PROA |
| ATOM | 3201 | CD2 | LEU | P | 207 | -4.693 | -26.878 | -15.960 | 0.00 | 0.00 | PROA |
| ATOM | 3202 | HD21 | LEU | P | 207 | -5.439 | -26.891 | -16.782 | 0.00 | 0.00 | PROA |
| ATOM | 3203 | HD22 | LEU | P | 207 | -4.250 | -27.888 | -15.828 | 0.00 | 0.00 | PROA |
| ATOM | 3204 | HD23 | LEU | P | 207 | -4.026 | -26.031 | -16.228 | 0.00 | 0.00 | PROA |
| ATOM | 3205 | C | LEU | P | 207 | -8.822 | -28.067 | -15.493 | 0.00 | 0.00 | PROA |
| ATOM | 3206 | O | LEU | P | 207 | -8.786 | -28.785 | -16.548 | 0.00 | 0.00 | PROA |
| ATOM | 3207 | N | THR | P | 208 | -9.660 | -28.301 | -14.439 | 0.00 | 0.00 | PROA |
| ATOM | 3208 | HN | THR | P | 208 | -9.461 | -27.740 | -13.639 | 0.00 | 0.00 | PROA |
| ATOM | 3209 | CA | THR | P | 208 | -10.569 | -29.427 | -14.461 | 0.00 | 0.00 | PROA |
| ATOM | 3210 | HA | THR | P | 208 | -10.008 | -30.304 | -14.750 | 0.00 | 0.00 | PROA |
| ATOM | 3211 | CB | THR | P | 208 | -11.294 | -29.641 | -13.096 | 0.00 | 0.00 | PROA |
| ATOM | 3212 | HB | THR | P | 208 | -11.849 | -28.699 | -12.897 | 0.00 | 0.00 | PROA |

| | | | | | | | | | | | |
|---|---|---|---|---|---|---|---|---|---|---|---|
| ATOM | 3213 | OG1 | THR | P | 208 | -10.254 | -29.838 | -12.167 | 0.00 | 0.00 | PROA |
| ATOM | 3214 | HG1 | THR | P | 208 | -9.798 | -28.994 | -12.134 | 0.00 | 0.00 | PROA |
| ATOM | 3215 | CG2 | THR | P | 208 | -12.262 | -30.890 | -13.026 | 0.00 | 0.00 | PROA |
| ATOM | 3216 | HG21 | THR | P | 208 | -13.230 | -30.829 | -13.570 | 0.00 | 0.00 | PROA |
| ATOM | 3217 | HG22 | THR | P | 208 | -12.561 | -31.102 | -11.977 | 0.00 | 0.00 | PROA |
| ATOM | 3218 | HG23 | THR | P | 208 | -11.774 | -31.836 | -13.342 | 0.00 | 0.00 | PROA |
| ATOM | 3219 | C | THR | P | 208 | -11.603 | -29.327 | -15.508 | 0.00 | 0.00 | PROA |
| ATOM | 3220 | O | THR | P | 208 | -11.803 | -30.365 | -16.226 | 0.00 | 0.00 | PROA |
| ATOM | 3221 | N | GLU | P | 209 | -12.105 | -28.091 | -15.662 | 0.00 | 0.00 | PROA |
| ATOM | 3222 | HN | GLU | P | 209 | -11.755 | -27.384 | -15.053 | 0.00 | 0.00 | PROA |
| ATOM | 3223 | CA | GLU | P | 209 | -13.085 | -27.672 | -16.684 | 0.00 | 0.00 | PROA |
| ATOM | 3224 | HA | GLU | P | 209 | -13.972 | -28.274 | -16.557 | 0.00 | 0.00 | PROA |
| ATOM | 3225 | CB | GLU | P | 209 | -13.616 | -26.189 | -16.448 | 0.00 | 0.00 | PROA |
| ATOM | 3226 | HB1 | GLU | P | 209 | -14.316 | -26.086 | -17.305 | 0.00 | 0.00 | PROA |
| ATOM | 3227 | HB2 | GLU | P | 209 | -12.776 | -25.463 | -16.482 | 0.00 | 0.00 | PROA |
| ATOM | 3228 | CG | GLU | P | 209 | -14.526 | -25.968 | -15.164 | 0.00 | 0.00 | PROA |
| ATOM | 3229 | HG1 | GLU | P | 209 | -13.948 | -26.307 | -14.278 | 0.00 | 0.00 | PROA |
| ATOM | 3230 | HG2 | GLU | P | 209 | -15.454 | -26.554 | -15.332 | 0.00 | 0.00 | PROA |
| ATOM | 3231 | CD | GLU | P | 209 | -14.920 | -24.531 | -14.849 | 0.00 | 0.00 | PROA |
| ATOM | 3232 | OE1 | GLU | P | 209 | -14.224 | -23.513 | -15.097 | 0.00 | 0.00 | PROA |
| ATOM | 3233 | OE2 | GLU | P | 209 | -16.098 | -24.420 | -14.337 | 0.00 | 0.00 | PROA |
| ATOM | 3234 | C | GLU | P | 209 | -12.540 | -27.811 | -18.140 | 0.00 | 0.00 | PROA |
| ATOM | 3235 | O | GLU | P | 209 | -13.095 | -28.397 | -19.081 | 0.00 | 0.00 | PROA |
| ATOM | 3236 | N | SER | P | 210 | -11.315 | -27.337 | -18.296 | 0.00 | 0.00 | PROA |
| ATOM | 3237 | HN | SER | P | 210 | -10.901 | -26.774 | -17.584 | 0.00 | 0.00 | PROA |
| ATOM | 3238 | CA | SER | P | 210 | -10.545 | -27.451 | -19.549 | 0.00 | 0.00 | PROA |
| ATOM | 3239 | HA | SER | P | 210 | -11.065 | -26.948 | -20.351 | 0.00 | 0.00 | PROA |
| ATOM | 3240 | CB | SER | P | 210 | -9.085 | -26.793 | -19.461 | 0.00 | 0.00 | PROA |
| ATOM | 3241 | HB1 | SER | P | 210 | -8.614 | -27.095 | -18.501 | 0.00 | 0.00 | PROA |
| ATOM | 3242 | HB2 | SER | P | 210 | -9.232 | -25.694 | -19.525 | 0.00 | 0.00 | PROA |
| ATOM | 3243 | OG | SER | P | 210 | -8.177 | -26.990 | -20.542 | 0.00 | 0.00 | PROA |
| ATOM | 3244 | HG1 | SER | P | 210 | -7.574 | -27.673 | -20.241 | 0.00 | 0.00 | PROA |
| ATOM | 3245 | C | SER | P | 210 | -10.418 | -28.898 | -20.016 | 0.00 | 0.00 | PROA |
| ATOM | 3246 | O | SER | P | 210 | -10.783 | -29.248 | -21.118 | 0.00 | 0.00 | PROA |
| ATOM | 3247 | N | HSD | P | 211 | -10.002 | -29.819 | -19.100 | 0.00 | 0.00 | PROA |
| ATOM | 3248 | HN | HSD | P | 211 | -9.672 | -29.542 | -18.201 | 0.00 | 0.00 | PROA |
| ATOM | 3249 | CA | HSD | P | 211 | -9.930 | -31.303 | -19.322 | 0.00 | 0.00 | PROA |
| ATOM | 3250 | HA | HSD | P | 211 | -9.225 | -31.508 | -20.114 | 0.00 | 0.00 | PROA |
| ATOM | 3251 | CB | HSD | P | 211 | -9.226 | -31.889 | -18.060 | 0.00 | 0.00 | PROA |
| ATOM | 3252 | HB1 | HSD | P | 211 | -9.465 | -32.973 | -17.999 | 0.00 | 0.00 | PROA |
| ATOM | 3253 | HB2 | HSD | P | 211 | -9.671 | -31.419 | -17.158 | 0.00 | 0.00 | PROA |
| ATOM | 3254 | ND1 | HSD | P | 211 | -6.978 | -32.101 | -19.243 | 0.00 | 0.00 | PROA |
| ATOM | 3255 | HD1 | HSD | P | 211 | -7.369 | -32.663 | -19.972 | 0.00 | 0.00 | PROA |
| ATOM | 3256 | CG | HSD | P | 211 | -7.699 | -31.568 | -18.198 | 0.00 | 0.00 | PROA |
| ATOM | 3257 | CE1 | HSD | P | 211 | -5.697 | -31.908 | -19.015 | 0.00 | 0.00 | PROA |
| ATOM | 3258 | HE1 | HSD | P | 211 | -4.954 | -32.330 | -19.692 | 0.00 | 0.00 | PROA |
| ATOM | 3259 | NE2 | HSD | P | 211 | -5.528 | -31.238 | -17.887 | 0.00 | 0.00 | PROA |
| ATOM | 3260 | CD2 | HSD | P | 211 | -6.799 | -31.033 | -17.357 | 0.00 | 0.00 | PROA |
| ATOM | 3261 | HD2 | HSD | P | 211 | -7.090 | -30.594 | -16.411 | 0.00 | 0.00 | PROA |
| ATOM | 3262 | C | HSD | P | 211 | -11.239 | -31.927 | -19.804 | 0.00 | 0.00 | PROA |
| ATOM | 3263 | O | HSD | P | 211 | -11.183 | -32.822 | -20.638 | 0.00 | 0.00 | PROA |
| ATOM | 3264 | N | ASP | P | 212 | -12.340 | -31.503 | -19.214 | 0.00 | 0.00 | PROA |
| ATOM | 3265 | HN | ASP | P | 212 | -12.418 | -30.650 | -18.703 | 0.00 | 0.00 | PROA |
| ATOM | 3266 | CA | ASP | P | 212 | -13.706 | -31.992 | -19.595 | 0.00 | 0.00 | PROA |
| ATOM | 3267 | HA | ASP | P | 212 | -13.665 | -33.066 | -19.696 | 0.00 | 0.00 | PROA |
| ATOM | 3268 | CB | ASP | P | 212 | -14.735 | -31.277 | -18.700 | 0.00 | 0.00 | PROA |
| ATOM | 3269 | HB1 | ASP | P | 212 | -14.824 | -30.209 | -18.991 | 0.00 | 0.00 | PROA |
| ATOM | 3270 | HB2 | ASP | P | 212 | -14.474 | -31.392 | -17.626 | 0.00 | 0.00 | PROA |
| ATOM | 3271 | CG | ASP | P | 212 | -16.149 | -31.946 | -18.825 | 0.00 | 0.00 | PROA |
| ATOM | 3272 | OD1 | ASP | P | 212 | -16.278 | -33.197 | -19.044 | 0.00 | 0.00 | PROA |
| ATOM | 3273 | OD2 | ASP | P | 212 | -17.146 | -31.109 | -18.848 | 0.00 | 0.00 | PROA |
| ATOM | 3274 | C | ASP | P | 212 | -13.996 | -31.620 | -21.091 | 0.00 | 0.00 | PROA |
| ATOM | 3275 | O | ASP | P | 212 | -14.567 | -32.361 | -21.816 | 0.00 | 0.00 | PROA |
| ATOM | 3276 | N | ARG | P | 213 | -13.529 | -30.418 | -21.461 | 0.00 | 0.00 | PROA |
| ATOM | 3277 | HN | ARG | P | 213 | -13.064 | -29.861 | -20.777 | 0.00 | 0.00 | PROA |
| ATOM | 3278 | CA | ARG | P | 213 | -13.707 | -29.914 | -22.874 | 0.00 | 0.00 | PROA |
| ATOM | 3279 | HA | ARG | P | 213 | -14.652 | -30.343 | -23.174 | 0.00 | 0.00 | PROA |
| ATOM | 3280 | CB | ARG | P | 213 | -13.492 | -28.394 | -23.030 | 0.00 | 0.00 | PROA |
| ATOM | 3281 | HB1 | ARG | P | 213 | -13.529 | -28.173 | -24.118 | 0.00 | 0.00 | PROA |
| ATOM | 3282 | HB2 | ARG | P | 213 | -12.482 | -28.133 | -22.648 | 0.00 | 0.00 | PROA |
| ATOM | 3283 | CG | ARG | P | 213 | -14.608 | -27.663 | -22.292 | 0.00 | 0.00 | PROA |
| ATOM | 3284 | HG1 | ARG | P | 213 | -14.676 | -27.985 | -21.231 | 0.00 | 0.00 | PROA |
| ATOM | 3285 | HG2 | ARG | P | 213 | -15.433 | -28.171 | -22.835 | 0.00 | 0.00 | PROA |

```
ATOM   3286  CD  ARG P 213     -14.756 -26.132 -22.528  0.00  0.00      PROA
ATOM   3287  HD1 ARG P 213     -15.680 -25.769 -22.029  0.00  0.00      PROA
ATOM   3288  HD2 ARG P 213     -14.910 -25.933 -23.610  0.00  0.00      PROA
ATOM   3289  NE  ARG P 213     -13.538 -25.482 -22.019  0.00  0.00      PROA
ATOM   3290  HE  ARG P 213     -12.819 -25.258 -22.676  0.00  0.00      PROA
ATOM   3291  CZ  ARG P 213     -13.303 -24.965 -20.832  0.00  0.00      PROA
ATOM   3292  NH1 ARG P 213     -14.191 -24.894 -19.893  0.00  0.00      PROA
ATOM   3293 HH11 ARG P 213     -15.062 -25.355 -20.062  0.00  0.00      PROA
ATOM   3294 HH12 ARG P 213     -14.029 -24.390 -19.044  0.00  0.00      PROA
ATOM   3295  NH2 ARG P 213     -12.169 -24.442 -20.566  0.00  0.00      PROA
ATOM   3296 HH21 ARG P 213     -11.443 -24.381 -21.251  0.00  0.00      PROA
ATOM   3297 HH22 ARG P 213     -12.048 -23.994 -19.680  0.00  0.00      PROA
ATOM   3298  C   ARG P 213     -12.797 -30.623 -23.912  0.00  0.00      PROA
ATOM   3299  O   ARG P 213     -13.258 -30.843 -25.054  0.00  0.00      PROA
ATOM   3300  N   GLN P 214     -11.599 -31.103 -23.512  0.00  0.00      PROA
ATOM   3301  HN  GLN P 214     -11.259 -31.042 -22.577  0.00  0.00      PROA
ATOM   3302  CA  GLN P 214     -10.717 -31.754 -24.466  0.00  0.00      PROA
ATOM   3303  HA  GLN P 214     -10.850 -31.460 -25.496  0.00  0.00      PROA
ATOM   3304  CB  GLN P 214      -9.238 -31.443 -24.225  0.00  0.00      PROA
ATOM   3305  HB1 GLN P 214      -8.671 -32.019 -24.987  0.00  0.00      PROA
ATOM   3306  HB2 GLN P 214      -8.965 -31.766 -23.198  0.00  0.00      PROA
ATOM   3307  CG  GLN P 214      -9.104 -29.916 -24.426  0.00  0.00      PROA
ATOM   3308  HG1 GLN P 214      -9.876 -29.288 -23.933  0.00  0.00      PROA
ATOM   3309  HG2 GLN P 214      -9.164 -29.671 -25.508  0.00  0.00      PROA
ATOM   3310  CD  GLN P 214      -7.762 -29.407 -23.733  0.00  0.00      PROA
ATOM   3311  OE1 GLN P 214      -6.757 -29.029 -24.344  0.00  0.00      PROA
ATOM   3312  NE2 GLN P 214      -7.899 -29.274 -22.394  0.00  0.00      PROA
ATOM   3313 HE21 GLN P 214      -7.095 -29.072 -21.835  0.00  0.00      PROA
ATOM   3314 HE22 GLN P 214      -8.698 -29.648 -21.923  0.00  0.00      PROA
ATOM   3315  C   GLN P 214     -10.912 -33.290 -24.462  0.00  0.00      PROA
ATOM   3316  O   GLN P 214     -10.640 -33.946 -25.446  0.00  0.00      PROA
ATOM   3317  N   ALA P 215     -11.408 -33.851 -23.307  0.00  0.00      PROA
ATOM   3318  HN  ALA P 215     -11.713 -33.279 -22.550  0.00  0.00      PROA
ATOM   3319  CA  ALA P 215     -11.439 -35.281 -23.172  0.00  0.00      PROA
ATOM   3320  HA  ALA P 215     -10.935 -35.671 -24.044  0.00  0.00      PROA
ATOM   3321  CB  ALA P 215     -10.563 -35.758 -21.984  0.00  0.00      PROA
ATOM   3322  HB1 ALA P 215     -10.288 -36.834 -21.992  0.00  0.00      PROA
ATOM   3323  HB2 ALA P 215     -10.939 -35.473 -20.978  0.00  0.00      PROA
ATOM   3324  HB3 ALA P 215      -9.609 -35.189 -21.996  0.00  0.00      PROA
ATOM   3325  C   ALA P 215     -12.812 -35.815 -23.010  0.00  0.00      PROA
ATOM   3326  O   ALA P 215     -13.764 -35.068 -22.700  0.00  0.00      PROA
ATOM   3327  N   LYS P 216     -13.008 -37.132 -23.176  0.00  0.00      PROA
ATOM   3328  HN  LYS P 216     -12.258 -37.668 -23.556  0.00  0.00      PROA
ATOM   3329  CA  LYS P 216     -14.247 -37.864 -22.847  0.00  0.00      PROA
ATOM   3330  HA  LYS P 216     -15.117 -37.263 -22.626  0.00  0.00      PROA
ATOM   3331  CB  LYS P 216     -14.568 -38.917 -23.978  0.00  0.00      PROA
ATOM   3332  HB1 LYS P 216     -15.506 -39.477 -23.775  0.00  0.00      PROA
ATOM   3333  HB2 LYS P 216     -13.747 -39.664 -24.027  0.00  0.00      PROA
ATOM   3334  CG  LYS P 216     -14.735 -38.224 -25.370  0.00  0.00      PROA
ATOM   3335  HG1 LYS P 216     -15.025 -39.017 -26.093  0.00  0.00      PROA
ATOM   3336  HG2 LYS P 216     -13.794 -37.750 -25.721  0.00  0.00      PROA
ATOM   3337  CD  LYS P 216     -15.743 -37.064 -25.430  0.00  0.00      PROA
ATOM   3338  HD1 LYS P 216     -15.594 -36.535 -26.396  0.00  0.00      PROA
ATOM   3339  HD2 LYS P 216     -15.416 -36.298 -24.695  0.00  0.00      PROA
ATOM   3340  CE  LYS P 216     -17.260 -37.323 -25.148  0.00  0.00      PROA
ATOM   3341  HE1 LYS P 216     -17.830 -36.375 -25.254  0.00  0.00      PROA
ATOM   3342  HE2 LYS P 216     -17.600 -37.808 -24.208  0.00  0.00      PROA
ATOM   3343  NZ  LYS P 216     -17.747 -38.231 -26.175  0.00  0.00      PROA
ATOM   3344  HZ1 LYS P 216     -17.274 -39.146 -26.032  0.00  0.00      PROA
ATOM   3345  HZ2 LYS P 216     -17.543 -37.902 -27.140  0.00  0.00      PROA
ATOM   3346  HZ3 LYS P 216     -18.775 -38.392 -26.205  0.00  0.00      PROA
ATOM   3347  C   LYS P 216     -14.040 -38.598 -21.555  0.00  0.00      PROA
ATOM   3348  OT1 LYS P 216     -12.859 -38.828 -21.161  0.00  0.00      PROA
ATOM   3349  OT2 LYS P 216     -15.065 -38.790 -20.799  0.00  0.00      PROA
ATOM   3350  CAY LYS P 217      14.788 -15.591  -6.202  0.00  0.00      PROB
ATOM   3351  HY1 LYS P 217      14.320 -16.574  -5.978  0.00  0.00      PROB
ATOM   3352  HY2 LYS P 217      15.673 -15.978  -6.750  0.00  0.00      PROB
ATOM   3353  HY3 LYS P 217      14.156 -14.875  -6.769  0.00  0.00      PROB
ATOM   3354  CY  LYS P 217      15.272 -14.840  -4.938  0.00  0.00      PROB
ATOM   3355  OY  LYS P 217      15.143 -15.297  -3.827  0.00  0.00      PROB
ATOM   3356  N   LYS P 217      15.930 -13.682  -5.071  0.00  0.00      PROB
ATOM   3357  HN  LYS P 217      16.035 -13.423  -6.028  0.00  0.00      PROB
ATOM   3358  CA  LYS P 217      16.255 -12.723  -4.052  0.00  0.00      PROB
```

```
ATOM   3359  HA  LYS P 217      16.415 -13.315  -3.163  0.00  0.00      PROB
ATOM   3360  CB  LYS P 217      17.575 -12.027  -4.242  0.00  0.00      PROB
ATOM   3361  HB1 LYS P 217      17.601 -11.297  -3.405  0.00  0.00      PROB
ATOM   3362  HB2 LYS P 217      17.574 -11.386  -5.150  0.00  0.00      PROB
ATOM   3363  CG  LYS P 217      18.838 -12.904  -4.251  0.00  0.00      PROB
ATOM   3364  HG1 LYS P 217      18.844 -13.651  -5.073  0.00  0.00      PROB
ATOM   3365  HG2 LYS P 217      18.778 -13.449  -3.284  0.00  0.00      PROB
ATOM   3366  CD  LYS P 217      20.142 -12.066  -4.403  0.00  0.00      PROB
ATOM   3367  HD1 LYS P 217      19.979 -11.117  -3.848  0.00  0.00      PROB
ATOM   3368  HD2 LYS P 217      20.214 -11.783  -5.475  0.00  0.00      PROB
ATOM   3369  CE  LYS P 217      21.335 -12.838  -3.898  0.00  0.00      PROB
ATOM   3370  HE1 LYS P 217      21.141 -13.917  -4.079  0.00  0.00      PROB
ATOM   3371  HE2 LYS P 217      21.492 -12.745  -2.803  0.00  0.00      PROB
ATOM   3372  NZ  LYS P 217      22.616 -12.454  -4.606  0.00  0.00      PROB
ATOM   3373  HZ1 LYS P 217      22.858 -11.480  -4.333  0.00  0.00      PROB
ATOM   3374  HZ2 LYS P 217      22.512 -12.528  -5.638  0.00  0.00      PROB
ATOM   3375  HZ3 LYS P 217      23.361 -13.079  -4.237  0.00  0.00      PROB
ATOM   3376  C   LYS P 217      15.139 -11.688  -3.809  0.00  0.00      PROB
ATOM   3377  O   LYS P 217      14.433 -11.274  -4.732  0.00  0.00      PROB
ATOM   3378  N   VAL P 218      15.008 -11.197  -2.576  0.00  0.00      PROB
ATOM   3379  HN  VAL P 218      15.604 -11.642  -1.912  0.00  0.00      PROB
ATOM   3380  CA  VAL P 218      14.007 -10.247  -2.168  0.00  0.00      PROB
ATOM   3381  HA  VAL P 218      13.123 -10.367  -2.777  0.00  0.00      PROB
ATOM   3382  CB  VAL P 218      13.585 -10.486  -0.631  0.00  0.00      PROB
ATOM   3383  HB  VAL P 218      14.525 -10.465  -0.039  0.00  0.00      PROB
ATOM   3384  CG1 VAL P 218      12.521  -9.584  -0.009  0.00  0.00      PROB
ATOM   3385 HG11 VAL P 218      13.021  -8.599   0.104  0.00  0.00      PROB
ATOM   3386 HG12 VAL P 218      12.069 -10.016   0.909  0.00  0.00      PROB
ATOM   3387 HG13 VAL P 218      11.819  -9.397  -0.850  0.00  0.00      PROB
ATOM   3388  CG2 VAL P 218      13.084 -11.945  -0.461  0.00  0.00      PROB
ATOM   3389 HG21 VAL P 218      12.303 -12.099  -1.236  0.00  0.00      PROB
ATOM   3390 HG22 VAL P 218      12.528 -11.963   0.500  0.00  0.00      PROB
ATOM   3391 HG23 VAL P 218      13.871 -12.721  -0.348  0.00  0.00      PROB
ATOM   3392  C   VAL P 218      14.389  -8.761  -2.336  0.00  0.00      PROB
ATOM   3393  O   VAL P 218      15.468  -8.352  -1.876  0.00  0.00      PROB
ATOM   3394  N   GLY P 219      13.561  -7.920  -2.996  0.00  0.00      PROB
ATOM   3395  HN  GLY P 219      12.731  -8.287  -3.409  0.00  0.00      PROB
ATOM   3396  CA  GLY P 219      13.813  -6.434  -3.092  0.00  0.00      PROB
ATOM   3397  HA1 GLY P 219      13.948  -5.945  -2.139  0.00  0.00      PROB
ATOM   3398  HA2 GLY P 219      12.934  -6.084  -3.613  0.00  0.00      PROB
ATOM   3399  C   GLY P 219      15.015  -6.044  -3.998  0.00  0.00      PROB
ATOM   3400  O   GLY P 219      15.768  -6.910  -4.391  0.00  0.00      PROB
ATOM   3401  N   LEU P 220      15.210  -4.753  -4.315  0.00  0.00      PROB
ATOM   3402  HN  LEU P 220      14.661  -4.052  -3.867  0.00  0.00      PROB
ATOM   3403  CA  LEU P 220      16.435  -4.384  -5.149  0.00  0.00      PROB
ATOM   3404  HA  LEU P 220      16.432  -5.060  -5.991  0.00  0.00      PROB
ATOM   3405  CB  LEU P 220      16.312  -2.938  -5.720  0.00  0.00      PROB
ATOM   3406  HB1 LEU P 220      17.011  -2.761  -6.565  0.00  0.00      PROB
ATOM   3407  HB2 LEU P 220      16.517  -2.263  -4.861  0.00  0.00      PROB
ATOM   3408  CG  LEU P 220      14.954  -2.491  -6.282  0.00  0.00      PROB
ATOM   3409  HG  LEU P 220      14.298  -2.424  -5.388  0.00  0.00      PROB
ATOM   3410  CD1 LEU P 220      15.003  -1.013  -6.858  0.00  0.00      PROB
ATOM   3411 HD11 LEU P 220      14.152  -0.955  -7.569  0.00  0.00      PROB
ATOM   3412 HD12 LEU P 220      15.866  -1.174  -7.539  0.00  0.00      PROB
ATOM   3413 HD13 LEU P 220      15.122  -0.244  -6.065  0.00  0.00      PROB
ATOM   3414  CD2 LEU P 220      14.349  -3.459  -7.333  0.00  0.00      PROB
ATOM   3415 HD21 LEU P 220      13.402  -3.005  -7.697  0.00  0.00      PROB
ATOM   3416 HD22 LEU P 220      14.199  -4.418  -6.793  0.00  0.00      PROB
ATOM   3417 HD23 LEU P 220      15.066  -3.535  -8.178  0.00  0.00      PROB
ATOM   3418  C   LEU P 220      17.690  -4.389  -4.399  0.00  0.00      PROB
ATOM   3419  O   LEU P 220      17.694  -4.434  -3.149  0.00  0.00      PROB
ATOM   3420  N   ALA P 221      18.869  -4.492  -5.105  0.00  0.00      PROB
ATOM   3421  HN  ALA P 221      18.826  -4.586  -6.097  0.00  0.00      PROB
ATOM   3422  CA  ALA P 221      20.104  -4.858  -4.463  0.00  0.00      PROB
ATOM   3423  HA  ALA P 221      19.989  -4.880  -3.389  0.00  0.00      PROB
ATOM   3424  CB  ALA P 221      20.741  -6.082  -5.089  0.00  0.00      PROB
ATOM   3425  HB1 ALA P 221      20.835  -6.023  -6.194  0.00  0.00      PROB
ATOM   3426  HB2 ALA P 221      20.146  -6.974  -4.799  0.00  0.00      PROB
ATOM   3427  HB3 ALA P 221      21.731  -6.246  -4.613  0.00  0.00      PROB
ATOM   3428  C   ALA P 221      20.968  -3.702  -4.795  0.00  0.00      PROB
ATOM   3429  O   ALA P 221      20.712  -2.949  -5.742  0.00  0.00      PROB
ATOM   3430  N   LEU P 222      22.075  -3.534  -4.048  0.00  0.00      PROB
ATOM   3431  HN  LEU P 222      22.192  -4.177  -3.295  0.00  0.00      PROB
```

```
ATOM   3432  CA  LEU P 222      22.987  -2.394  -4.169  0.00  0.00      PROB
ATOM   3433  HA  LEU P 222      22.411  -1.514  -3.923  0.00  0.00      PROB
ATOM   3434  CB  LEU P 222      24.186  -2.453  -3.192  0.00  0.00      PROB
ATOM   3435  HB1 LEU P 222      24.770  -1.512  -3.274  0.00  0.00      PROB
ATOM   3436  HB2 LEU P 222      24.829  -3.321  -3.453  0.00  0.00      PROB
ATOM   3437  CG  LEU P 222      23.772  -2.505  -1.699  0.00  0.00      PROB
ATOM   3438  HG  LEU P 222      23.234  -3.422  -1.376  0.00  0.00      PROB
ATOM   3439  CD1 LEU P 222      25.041  -2.624  -0.760  0.00  0.00      PROB
ATOM   3440 HD11 LEU P 222      24.687  -2.600   0.293  0.00  0.00      PROB
ATOM   3441 HD12 LEU P 222      25.695  -1.759  -1.001  0.00  0.00      PROB
ATOM   3442 HD13 LEU P 222      25.557  -3.582  -0.986  0.00  0.00      PROB
ATOM   3443  CD2 LEU P 222      22.901  -1.342  -1.294  0.00  0.00      PROB
ATOM   3444 HD21 LEU P 222      21.852  -1.704  -1.359  0.00  0.00      PROB
ATOM   3445 HD22 LEU P 222      22.936  -0.398  -1.878  0.00  0.00      PROB
ATOM   3446 HD23 LEU P 222      23.083  -1.060  -0.235  0.00  0.00      PROB
ATOM   3447  C   LEU P 222      23.625  -2.301  -5.586  0.00  0.00      PROB
ATOM   3448  O   LEU P 222      23.768  -3.227  -6.326  0.00  0.00      PROB
ATOM   3449  N   GLU P 223      24.032  -1.122  -6.024  0.00  0.00      PROB
ATOM   3450  HN  GLU P 223      23.884  -0.281  -5.510  0.00  0.00      PROB
ATOM   3451  CA  GLU P 223      24.259  -0.876  -7.441  0.00  0.00      PROB
ATOM   3452  HA  GLU P 223      23.634  -1.466  -8.094  0.00  0.00      PROB
ATOM   3453  CB  GLU P 223      23.906   0.600  -7.827  0.00  0.00      PROB
ATOM   3454  HB1 GLU P 223      24.324   0.882  -8.817  0.00  0.00      PROB
ATOM   3455  HB2 GLU P 223      24.463   1.202  -7.078  0.00  0.00      PROB
ATOM   3456  CG  GLU P 223      22.366   0.987  -7.849  0.00  0.00      PROB
ATOM   3457  HG1 GLU P 223      22.132   2.070  -7.766  0.00  0.00      PROB
ATOM   3458  HG2 GLU P 223      21.779   0.489  -7.048  0.00  0.00      PROB
ATOM   3459  CD  GLU P 223      21.653   0.690  -9.220  0.00  0.00      PROB
ATOM   3460  OE1 GLU P 223      21.719  -0.471  -9.686  0.00  0.00      PROB
ATOM   3461  OE2 GLU P 223      21.038   1.596  -9.812  0.00  0.00      PROB
ATOM   3462  C   GLU P 223      25.752  -1.053  -7.800  0.00  0.00      PROB
ATOM   3463  O   GLU P 223      26.626  -0.660  -7.051  0.00  0.00      PROB
ATOM   3464  N   LEU P 224      26.122  -1.621  -8.901  0.00  0.00      PROB
ATOM   3465  HN  LEU P 224      25.398  -2.021  -9.458  0.00  0.00      PROB
ATOM   3466  CA  LEU P 224      27.435  -2.243  -9.113  0.00  0.00      PROB
ATOM   3467  HA  LEU P 224      28.079  -2.131  -8.254  0.00  0.00      PROB
ATOM   3468  CB  LEU P 224      27.293  -3.745  -9.395  0.00  0.00      PROB
ATOM   3469  HB1 LEU P 224      26.935  -3.812 -10.445  0.00  0.00      PROB
ATOM   3470  HB2 LEU P 224      26.755  -4.310  -8.604  0.00  0.00      PROB
ATOM   3471  CG  LEU P 224      28.716  -4.496  -9.364  0.00  0.00      PROB
ATOM   3472  HG  LEU P 224      29.163  -4.187 -10.333  0.00  0.00      PROB
ATOM   3473  CD1 LEU P 224      29.540  -4.168  -8.134  0.00  0.00      PROB
ATOM   3474 HD11 LEU P 224      29.037  -4.484  -7.194  0.00  0.00      PROB
ATOM   3475 HD12 LEU P 224      29.792  -3.089  -8.217  0.00  0.00      PROB
ATOM   3476 HD13 LEU P 224      30.530  -4.663  -8.227  0.00  0.00      PROB
ATOM   3477  CD2 LEU P 224      28.359  -6.076  -9.449  0.00  0.00      PROB
ATOM   3478 HD21 LEU P 224      27.787  -6.404  -8.555  0.00  0.00      PROB
ATOM   3479 HD22 LEU P 224      29.188  -6.802  -9.587  0.00  0.00      PROB
ATOM   3480 HD23 LEU P 224      27.706  -6.331 -10.311  0.00  0.00      PROB
ATOM   3481  C   LEU P 224      28.190  -1.506 -10.274  0.00  0.00      PROB
ATOM   3482  O   LEU P 224      27.800  -1.572 -11.438  0.00  0.00      PROB
ATOM   3483  N   GLU P 225      29.308  -0.860  -9.885  0.00  0.00      PROB
ATOM   3484  HN  GLU P 225      29.552  -0.726  -8.928  0.00  0.00      PROB
ATOM   3485  CA  GLU P 225      30.231  -0.419 -10.908  0.00  0.00      PROB
ATOM   3486  HA  GLU P 225      29.715   0.242 -11.589  0.00  0.00      PROB
ATOM   3487  CB  GLU P 225      31.318   0.437 -10.261  0.00  0.00      PROB
ATOM   3488  HB1 GLU P 225      32.005   0.863 -11.023  0.00  0.00      PROB
ATOM   3489  HB2 GLU P 225      31.854  -0.177  -9.505  0.00  0.00      PROB
ATOM   3490  CG  GLU P 225      30.583   1.706  -9.658  0.00  0.00      PROB
ATOM   3491  HG1 GLU P 225      29.636   1.403  -9.162  0.00  0.00      PROB
ATOM   3492  HG2 GLU P 225      30.462   2.391 -10.525  0.00  0.00      PROB
ATOM   3493  CD  GLU P 225      31.421   2.337  -8.614  0.00  0.00      PROB
ATOM   3494  OE1 GLU P 225      32.476   2.923  -8.935  0.00  0.00      PROB
ATOM   3495  OE2 GLU P 225      30.965   2.261  -7.442  0.00  0.00      PROB
ATOM   3496  C   GLU P 225      30.869  -1.434 -11.726  0.00  0.00      PROB
ATOM   3497  O   GLU P 225      31.213  -2.535 -11.326  0.00  0.00      PROB
ATOM   3498  N   ALA P 226      31.078  -1.088 -12.971  0.00  0.00      PROB
ATOM   3499  HN  ALA P 226      30.649  -0.312 -13.426  0.00  0.00      PROB
ATOM   3500  CA  ALA P 226      31.820  -1.802 -14.004  0.00  0.00      PROB
ATOM   3501  HA  ALA P 226      31.613  -2.860 -13.929  0.00  0.00      PROB
ATOM   3502  CB  ALA P 226      31.399  -1.258 -15.323  0.00  0.00      PROB
ATOM   3503  HB1 ALA P 226      31.802  -0.240 -15.511  0.00  0.00      PROB
ATOM   3504  HB2 ALA P 226      30.299  -1.117 -15.262  0.00  0.00      PROB
```

```
ATOM   3505  HB3 ALA P 226      31.681  -1.795 -16.254  0.00  0.00      PROB
ATOM   3506  C   ALA P 226      33.359  -1.528 -13.829  0.00  0.00      PROB
ATOM   3507  OT1 ALA P 226      33.798  -0.411 -14.004  0.00  0.00      PROB
ATOM   3508  OT2 ALA P 226      34.171  -2.487 -13.702  0.00  0.00      PROB
END
```
