# Supplementary material for: Rational correction of pathogenic conformational defects in HTRA1
Source: Nat Commun. 2024 Jul 16;15:5944. doi: 10.1038/s41467-024-49982-8 (PMC11252331; doi:10.1038/s41467-024-49982-8)
Supplement: Supplementary file 17 — Reporting Summary [file 41467_2024_49982_MOESM17_ESM.pdf]

nature portfolio

Corresponding author(s): Michael Ehrmann and Martin Dichgans

Last updated by author(s): Nov 21, 2023

Reporting Summary

Nature Portfolio wishes to improve the reproducibility of the work that we publish. This form provides structure for consistency and transparency in reporting. For further information on Nature Portfolio policies, see our Editorial Policies and the Editorial Policy Checklist.

Statistics

For all statistical analyses, confirm that the following items are present in the figure legend, table legend, main text, or Methods section.

n/a	Confirmed	
[ ]	[x]	The exact sample size (*n*) for each experimental group/condition, given as a discrete number and unit of measurement
[ ]	[x]	A statement on whether measurements were taken from distinct samples or whether the same sample was measured repeatedly
[ ]	[x]	The statistical test(s) used AND whether they are one- or two-sided<br>*Only common tests should be described solely by name; describe more complex techniques in the Methods section.*
[x]	[ ]	A description of all covariates tested
[ ]	[x]	A description of any assumptions or corrections, such as tests of normality and adjustment for multiple comparisons
[ ]	[x]	A full description of the statistical parameters including central tendency (e.g. means) or other basic estimates (e.g. regression coefficient) AND variation (e.g. standard deviation) or associated estimates of uncertainty (e.g. confidence intervals)
[ ]	[x]	For null hypothesis testing, the test statistic (e.g. *F*, *t*, *r*) with confidence intervals, effect sizes, degrees of freedom and *P* value noted<br>*Give P values as exact values whenever suitable.*
[x]	[ ]	For Bayesian analysis, information on the choice of priors and Markov chain Monte Carlo settings
[x]	[ ]	For hierarchical and complex designs, identification of the appropriate level for tests and full reporting of outcomes
[x]	[ ]	Estimates of effect sizes (e.g. Cohen's *d*, Pearson's *r*), indicating how they were calculated

Our web collection on statistics for biologists contains articles on many of the points above.

Software and code

Policy information about availability of computer code

Data collection	For enzyme kinetics: Molecular Devices Spectramax 5e reader integrated software. For SEC(-MALS): Astra 8 software. For AUC: Optima XL-I (Beckman Inc.) integrated software. For nMS: Exactive Plus EMR Orbitrap mass spectrometer (ThermoFisher) integrated software. For NMR: Topspin 3.5 (Bruker). For MS: Q-Exactive HF mass spectrometer (Thermo Fisher Scientific) integrated software. For fluorescence microscopy: Axio Observer Z1 and Zen 2 blue edition interface (Zeiss). For RT-qPCR: LightCycler 480 II (Roche) integrated software. See Methods for additional information.
Data analysis	For X-Ray crystallography, SLS synchrotron data sets were integrated using XDS or DIALS and scaled with XSCALE. Structures were solved via molecular replacement with PHASER (CCP4 suite). Refinements were done with REFMAC (CCP4 suite) and PHENIX and model building with COOT. The r.m.s.d values were calculated with PYMOL. VMD 1.9.4 to setup simulation files and the analysis of MD trajectories, ParseFEP 1.9 for analysis of FEP calculations, gnuplot 5.2 to plot contact distance map. For SEC(-MALS): Astra 8 software, Origin software. For AUC data: SEDFIT software package. For nMS data: UniDec software (version 5.0.2). For NMR experiments: spectra were processed with Topspin 3.5 (Bruker) and integrated with the built-in T1T2 Dynamics module. The resulting DOSY signal intensities were plotted and fitted with GraphPad Prism 5.0. Docking simulations were performed using CABSDock. For analysis of MS data and statistics: Maxquant (version 1.5.5.1), Perseus (version 1.6.2.1) and R (version 4.0.2). For image processing and quantification: TotalLab Quant and ImageJ/Fiji (version 1.52p). For statistics and figure representation: GraphPad (8.3.1), Adobe Illustrator (2017), Excel (2016). See Methods for additional information.

For manuscripts utilizing custom algorithms or software that are central to the research but not yet described in published literature, software must be made available to editors and reviewers. We strongly encourage code deposition in a community repository (e.g. GitHub). See the Nature Portfolio guidelines for submitting code & software for further information.

Data

Policy information about availability of data

All manuscripts must include a data availability statement. This statement should provide the following information, where applicable:

- Accession codes, unique identifiers, or web links for publicly available datasets
- A description of any restrictions on data availability
- For clinical datasets or third party data, please ensure that the statement adheres to our policy

The structures of HTRA1 R274Q and D174R R274Q have been deposited in the Protein Data Bank (PDB) with the accession codes 6Z0E [https://www.rcsb.org/structure/6z0e], 6Z0X [https://www.rcsb.org/structure/6Z0X] and 6Z0Y [https://www.rcsb.org/structure/6Z0Y]. Mouse brain vessel proteomic data have been deposited to the ProteomeXchange Consortium via the PRIDE60 partner repository with the dataset identifier PXD024683 [https://www.ebi.ac.uk/pride/archive/projects/PXD024683]. The processed mouse brain vessel proteomic data are provided in Supplementary Data 1, 2 and 3. Uniprot is publicly available at https://www.uniprot.org. Source data underlying Fig.1, 2, 3, 4, 5, 6, Supplementary Fig. 1, 2, 5, 6, 7, 8, 9, 10, 11, 12, 13, 14, 15 and Supplementary Table 1 are provided as a Source Data file. Source data are provided with this paper.

Research involving human participants, their data, or biological material

Policy information about studies with human participants or human data. See also policy information about sex, gender (identity/presentation), and sexual orientation and race, ethnicity and racism.

Reporting on sex and gender	not applicable
Reporting on race, ethnicity, or other socially relevant groupings	not applicable
Population characteristics	not applicable
Recruitment	not applicable
Ethics oversight	not applicable

Note that full information on the approval of the study protocol must also be provided in the manuscript.

Field-specific reporting

Please select the one below that is the best fit for your research. If you are not sure, read the appropriate sections before making your selection.

[x] Life sciences [ ] Behavioural & social sciences [ ] Ecological, evolutionary & environmental sciences

For a reference copy of the document with all sections, see nature.com/documents/nr-reporting-summary-flat.pdf

Life sciences study design

All studies must disclose on these points even when the disclosure is negative.

Sample size	Sample sizes were determined based on results obtained in previous studies published by the authors (e.g. see Zellner et al. Acta Neuroptahologica 2018; Beaufort et al. Proc Natl. Acad Sci 2014; Rey et al., Proc Natl. Acad Sci 2022).
Data exclusions	For animal-based experiments, no data were excluded. For in vitro assays, outlier datapoints (marked by asterisks in Source Data) were excluded.
Replication	Animal-based experiments included 3-5 mice per genotype (as described in Figure Legends). There was no further replication. We show all data that have been generated and there were no data excluded.<br>In vitro experiments were replicated as described in the figure legends. Replication was successful, no dataset was excluded.
Randomization	For immunoblot analysis, immunohistochemistry, and mass spectrometry analyses of mouse vasculature, mice (and mouse-derived samples) were randomly selected after genotyping. There was no experimental intervention that would have required randomization. Animals were matched for age and sex and we did not control for any additional covariates as we do not see any covariates that would be meaningful to control for. No randomization was performed for in vitro studies.
Blinding	Blinding was applied to immunohistochemical analysis.<br>Mouse brain vessel proteomic and immunoblot analyses as well as in vitro studies were performed unblinded. The operator has limited influence on the results (eg, mass spectrometry, NMR-DOSY) and/or the raw data used for quantification (eg Coomassie gels and immunoblots) are provided as Source Data.

Reporting for specific materials, systems and methods

We require information from authors about some types of materials, experimental systems and methods used in many studies. Here, indicate whether each material, system or method listed is relevant to your study. If you are not sure if a list item applies to your research, read the appropriate section before selecting a response.

Materials & experimental systems

n/a	Involved in the study
☐	☒ Antibodies
☐	☒ Eukaryotic cell lines
☒	☐ Palaeontology and archaeology
☐	☒ Animals and other organisms
☒	☐ Clinical data
☒	☐ Dual use research of concern
☒	☐ Plants

Methods

n/a	Involved in the study
☒	☐ ChIP-seq
☒	☐ Flow cytometry
☒	☐ MRI-based neuroimaging

Antibodies

Antibodies used

Primary antibodies:
Anti Strep-tag Abs (Qiagen; #34850), IB dilution 1/1,000
Anti His-tag Abs (Qiagen; #34670), IB dilution 1/2,500
Anti-HTRA1 Abs (R&D Systems; #MAB2916), IB dilution 1/5,000
Anti-Ltbp4 Abs (R&D Systems; #AF2885); IB, dilution 1/1000; IHC: dilution 1/100
Anti-laminin Abs (Dako; #L9393), IHC dilution 1/50
Anti-actin Abs (Sigma-Aldrich; #A2066), IB dilution 1/500

Secondary antibodies:
Goat-Anti-Mouse-AP (Sigma-Aldrich #A1418), IB dilution 1/20,000
Rabbit Anti-Goat Immunoglobulins/HRP (Dako; #P0449), IB dilution 1/10,000
Goat Anti-Mouse Immunoglobulins/HRP (Dako; #P0447), IB dilution 1/10,000
Goat Anti-Rabbit Immunoglobulins/HRP (Dako; #P0448), IB dilution 1/10,000
Donkey Anti-Rabbit Immunoglobulins/Cy3 (Jackson; #711-165-152), IHC dilution 1/400
Donkey Anti-Goat Immunoglobulins/Alexa Fluor 488 (Jackson; #705-546-147), IHC dilution 1/400

Validation

All Abs are commercially available and were used according to the manufacturer´s instructions.

Anti Strep-tag Abs (Qiagen; #34850), used in the present study to detect Strep-tag by IB. Abs were validated by the supplier for detection of Strep-tag by IB. https://www.qiagen.com/us/products/discovery-and-translational-research/protein-purification/tagged-protein-expression-purification-detection/strep-tag-antibody

Anti His-tag Abs (Qiagen; #34670), used in the present study to detect His-tag by IB. Abs were validated by the supplier for detection of His-tag by IB. https://www.qiagen.com/us/products/discovery-and-translational-research/protein-purification/tagged-protein-expression-purification-detection/anti-his-antibodies-bsa-free?catno=34670

Anti-HTRA1 Abs (R&D Systems; #MAB2916) used in the present study to detect recombinant human HTRA1 by IB. Abs were validated by the supplier for detection of human HTRA1 by IB. https://www.rndsystems.com/products/human-htra1-prss11-antibody-275615_mab2916

Anti-Ltbp4 Abs (R&D Systems; #AF2885), used in the present study to detect mouse Ltbp4 by IB and IHC. Abs are sold by the supplier for detection of mouse Ltbp4 by Western blot. https://www.rndsystems.com/products/mouse-latent-tgf-beta-bp4-antibody_af2885. In addition, the reactivity of these Abs was verified for both IB and IHC analyses using tissue from Ltbp4+/+ compared to Ltpb4-/- mice (Bultmann-Mellin et al., Dis Model Mech, 2015).

Anti-laminin Abs (Dako; #L9393), used in the present study to detect mouse laminins by IHC. Abs were validated by the supplier for detection of mouse laminins by IHC. https://www.sigmaaldrich.com/DE/en/product/sigma/l9393?gclid=EAIaIQobChMI25vuuP6nggMV8JJoCR0Ufwo6EAAYASAAEgKTJPD_BwE.
Laminin signal was used as a control vessel-wall marker. Staining was restricted to blood vessels, as expected.

Anti-actin Abs (Sigma-Aldrich; #A2066), used in the present study to detect mouse actin by IB. Abs were validated by the supplier for detection of mouse actin by IB. https://www.sigmaaldrich.com/DE/de/product/sigma/a2066?gclid=EAIaIQobChMIjl_a0v2nggMVRIpoCR2NSQ64EAAYASAAEgKTGvD_BwE

Eukaryotic cell lines

Policy information about cell lines and Sex and Gender in Research

Cell line source(s)

HEK-293T: ATCC CRL-3216.

Authentication

The cell line used was not authenticated.

Mycoplasma contamination	The cell line was tested negative for mycoplasma contamination.
Commonly misidentified lines (See ICLAC register)	No commonly misidentified cell line was used in this study.

Animals and other research organisms

Policy information about studies involving animals; ARRIVE guidelines recommended for reporting animal research, and Sex and Gender in Research

Laboratory animals	Mus musculus, C57BL/6J, mixed males and females, 6 month-old.<br>Animals were kept under standard conditions in a specific pathogen-free facility at 20-24°C and 45-65% humidity on a 12-h light/dark cycle and had access to food and water ad libitum.
Wild animals	The study did not involve wild animals.
Reporting on sex	Mouse-based analyses were restricted to 3-5 sex-matched mice (mixed males and females) per genotype. The study was not designed for sex-based analyses and is not suitable for this purpose due to low sample size.
Field-collected samples	The study did not involve samples collected from the field.
Ethics oversight	All mouse-based experiments were performed in accordance with the German Animal Welfare Law and in compliance with the Government of Upper Bavaria. Following euthanasia, brain was collected for scientific purposes, which does not require ethical approval (TierSchG. § 4).

Note that full information on the approval of the study protocol must also be provided in the manuscript.

Plants

Seed stocks	not applicable
Novel plant genotypes	not applicable
Authentication	not applicable
